# Network-based anomaly detection algorithm reveals proteins with major roles in human tissues

**DOI:** 10.1093/gigascience/giaf034

**Published:** 2025-04-08

**Authors:** Dima Kagan, Juman Jubran, Esti Yeger-Lotem, Michael Fire

**Affiliations:** Department of Software and Information Systems Engineering, Ben-Gurion University of the Negev, Beer Sheva 84105, Israel; Department of Clinical Biochemistry and Pharmacology, Ben-Gurion University of the Negev, Beer Sheva 84105, Israel; Department of Clinical Biochemistry and Pharmacology, Ben-Gurion University of the Negev, Beer Sheva 84105, Israel; The National Institute for Biotechnology in the Negev, Ben-Gurion University of the Negev, Beer Sheva 84105, Israel; Department of Software and Information Systems Engineering, Ben-Gurion University of the Negev, Beer Sheva 84105, Israel

**Keywords:** protein–protein interaction (PPI) networks, anomaly detection, weighted graphs, machine learning

## Abstract

**Background:**

Proteins act through physical interactions with other molecules to maintain organismal health. Protein–protein interaction (PPI) networks have proved to be a powerful framework for obtaining insight into protein functions, cellular organization, response to signals, and disease states. In multicellular organisms, protein content varies between tissues, influencing tissue morphology and function. Weighted PPI networks, reflecting the likelihood of interactions in specific tissues, offer insights into tissue-specific processes and disease mechanisms. We hypothesized that detecting anomalous nodes in these networks could reveal proteins with key tissue-specific functions.

**Results:**

Here, we introduce Weighted Graph Anomalous Node Detection (WGAND), a novel machine-learning algorithm to identify anomalous nodes in weighted graphs. WGAND estimates expected edge weights and uses deviations to generate anomaly detection features, which are then used to score network nodes. We applied WGAND to weighted PPI networks of 17 human tissues. High-ranking anomalous nodes were enriched for proteins associated with tissue-specific diseases and tissue-specific biological processes, such as neuron signaling in the brain and spermatogenesis in the testis. WGAND outperformed other methods in terms of area under the ROC curve and precision at *K*, highlighting its effectiveness in uncovering biologically meaningful anomalies.

**Conclusions:**

Our findings demonstrate WGAND’s potential as a powerful tool for detecting anomalous proteins with significant biological roles. By identifying proteins involved in critical tissue-specific processes and diseases, WGAND offers valuable insights for discovering novel biomarkers and therapeutic targets. Its versatile algorithm is suitable for any weighted graph and is broadly applicable across various fields. The WGAND algorithm is available as an open-source Python library at https://github.com/data4goodlab/wgand.

## Introduction

Proteins are the prime molecules in living cells, driving and mediating all biological processes through interactions with other molecules. In multitissue organisms, protein content differs between tissues because proteins can either be absent from certain tissues or expressed at different levels [1]. These differences affect tissues’ morphology and function and susceptibility to aberrations [2]. Protein–protein interaction (PPI) networks, representing the interactions between proteins within a cell, provide a valuable framework for studying protein functions and cellular information transfer [3]. Weighted PPI networks, in which the edge weight reflects the likelihood of an interaction, offer a refined view of context-specific interactions [4]. For example, weighted PPI networks representing different human tissues helped understand tissue-specific protein functions, biological processes, and differences in disease susceptibilities [5–7].

In studying such complex networks, detecting anomalies could offer a unique perspective. Detecting anomalies is a critical problem in diverse domains, from cybersecurity to social network analysis. Anomalies refer to elements or objects “that appear to deviate markedly from other members of the sample in which it occurs” [8]. The study of anomalies has fascinated scientists for centuries, as anomalies often hold unique insights into the dynamics of complex systems [9].

Anomaly detection is an invaluable tool for various disciplines, from aviation to medicine, to uncover insights not easily gained through traditional methods [9]. For instance, in aviation, an anomaly found in airplane sensors may indicate a fault, while in medicine, it could signify a rare disease [10]. In biomedicine, many advances can be attributed to identifying unusual proteins and genes [11].

In recent years, anomaly detection in graphs (i.e., networks) has gained increasing attention due to its applicability in various domains such as cybersecurity, social networks, and biological networks [10]. The structure and interconnected nature of graphs can provide rich information for anomaly detection. However, the graph’s complex nature, including its size, diversity, and noise, makes this task challenging [10]. Over the years, many studies have addressed anomaly detection, especially in graphs. However, only a few studies focused on anomaly detection in weighted graphs [12]. For example, Akoglu et al. [13] introduced OddBall, an algorithm that detects anomalous nodes in weighted graphs. OddbBall focuses on neighborhoods around each node and extracts features from these neighborhoods. Then, these features are transformed into a score to pinpoint outliers. The following year, Davis et al. [14] presented Yagada, an algorithm that searches for structural and numerical anomalies in labeled graphs. In 2018, Kagan et al. [15] introduced an unsupervised classifier based on network topology to detect anomalous nodes. Their algorithm operates on the assumption that nodes with many improbable links are more likely to be anomalous. They successfully applied this method to networks of varying scales, demonstrating its ability to identify abnormal nodes effectively. More recently, Lee et al. [12] introduced GAWD, a method for detecting outliers in weighted graphs. GAWD iterates through the graph, searching for the ”best” substructures, which generate the largest compression when replaced with a supernode. The anomaly score is generated based on how well it was compressed.

In this study, we hypothesized that proteins involved in key biological roles within a specific tissue would appear as anomalous nodes in the PPI network representing that tissue. Among these proteins are those involved in main tissue processes, such as neuron signaling in the brain and spermatogenesis in the testis, and those that participate in tissue-specific diseases, such as BRCA1, aberrations of which increase the risk for breast and ovarian cancers. Previous methods aiming to reveal proteins with key tissue-specific roles have focused on the preferential expression of proteins in specific tissues [16], their network features [6], or both [7,17]. For example, using the DiffNet method for scoring protein interactions in tissue contexts [18], it was shown that subnetworks composed of the top 1% differential interaction were enriched for tissue-associated disease proteins. Likewise, by using the ProAct method for scoring biological processes [19], it was shown that tissue-associated disease proteins participated in processes that were highly active in the respective tissue [20]. Lastly, FUGUE predicted tissue-relevant genes by applying supervised machine-learning methods to transcriptional and network features of genes [7]. FUGUE distinguished tissue-specific or cell-type-specific genes better than conventional methods that used tissue-specific expression alone [7].

Here, we combined expression and network features with the anomaly concept. To identify anomalous proteins, we developed Weighted Graph Anomalous Node Detection (WGAND), a novel generic anomaly detection machine-learning-based algorithm for weighted graphs. WGAND is constructed based on the assumption that edge weights of anomalous nodes would deviate from their expected norm. Thus, the model estimated the expected edge weight for all edges in the network and then used the difference between the actual and the expected weight to generate anomaly detection features. These features were then used to train a model that generates anomaly scores for all the network nodes. To test our hypothesis, we applied the WGAND algorithm to weighted PPI networks of 17 different human tissues, where each network was composed of 13,523 nodes (proteins) and 134,223 edges (PPIs) with the same topology but with different edge weights. When applied to identify disease-tissue-associated proteins, we found that in most tissues, WGAND obtained a higher area under the ROC curve (AUC), a higher area under the precision-recall curve (PR-AUC), and a higher precision at K (P@K) than other methods. Moreover, high-ranking anomalous proteins were more likely to be associated with diseases than other proteins, were enriched for proteins involved in tissue-specific processes, and tended to be involved in processes that were preferentially active in the respective tissues. Notably, WGAND outperformed three other methods for identifying tissue-relevant or disease-associated proteins. Whereas our study used weighted PPI networks as a case study, WGAND is a generic method that could be applied to any weighted graph. WGAND is an open Python library [21].

## Results

**Figure 1:**
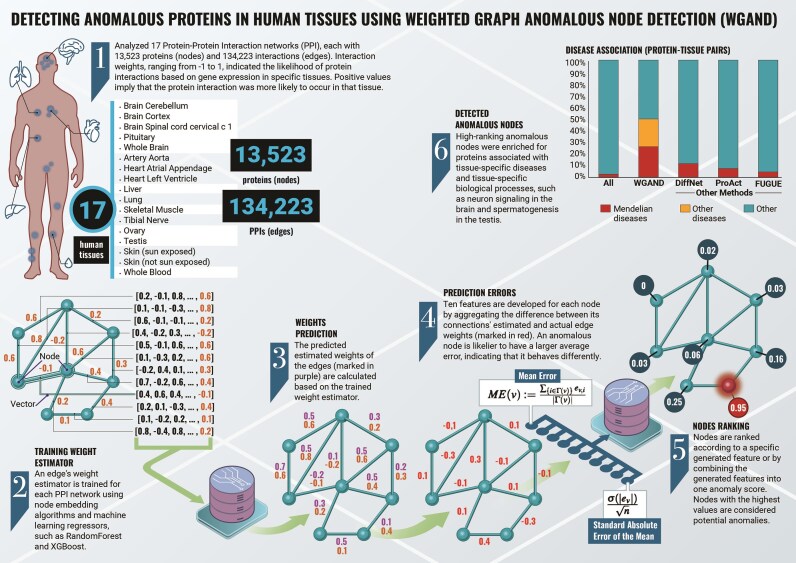
Methodology overview.

To create an anomaly detection model based on weighted graphs, we used 17 tissue-specific differential PPI networks (Methods, [18]). Each network consisted of 13,523 nodes (proteins) and 134,223 edges (PPIs). These networks had identical topologies and differed from each other only in their edge weights, ranging from −1 to 1 (Fig. 1, step 1).

The anomaly detection method assumes that the edge weights of anomalous nodes are likely to deviate from their expected norm. Hence, we constructed an anomaly detection model:

1. We trained an edge weight estimator to predict edge weights (Fig. 1, step 2).
2. We constructed meta-features based on the error of the edge weight prediction (Fig. 1, steps 3 and 4).
3. We trained a model on the meta-features to predict anomalous nodes (Fig. 1, steps 5 and 6).

### Using edge weight estimation errors to identify anomalous nodes

To build an edge weight estimator, we used node-embedding methods [22] to generate features representing the nodes, allowing us to predict the edge weight. We tested and evaluated five different node-embedding models and assessed their performance in detecting anomalous nodes based on AUC, PR-AUC, P@K, and runtime (Table 1). On average, the RandNE [23] embedding method showed the highest performance by all metrics, including the fastest average runtimes in generating the embeddings (Table 1). Although its AUC is slightly higher than other models, its P@K and PR-AUC show considerable gains compared with the rest of the models.

**Table 1:** Evaluation of different node-embedding models. Numbers in bold indicate the highest value obtained for each metric

Embedding model	AUC	PR-AUC	P@1	P@3	P@10	P@20	Embedding runtime (s)
DeepWalk	0.6629	0.0528	0.4118	0.2353	0.1941	0.1588	96
GLEE	0.6699	0.0417	0.3529	0.2549	0.1765	0.1412	4
Node2Vec	0.6658	0.0565	0.4118	0.2745	0.2412	0.1824	2,912
NodeSketch	0.6700	0.0569	0.4118	0.3137	0.2471	0.1941	229
RandNE	**0.6701**	**0.06155**	**0.5294**	**0.3725**	**0.2529**	**0.2147**	**1.6**

Next, we used the node-embedding features generated by RandNE and constructed three different edge weight estimators using different regression algorithms (Methods, Fig. 1, step 2). First, we evaluated their performances just on the edge-weight estimation task. The model created by LightGBM presented the best performance in terms of mean squared error (MSE) (Table 2). Second, we evaluated the performance of the generated anomaly detection models (Table 3). We found that, on average, when using RandomForest [24] as an edge weight estimator to generate the meta-features, the anomaly detection model achieved the highest score in all anomaly detection metrics. Therefore, we chose the combination of RandNE embedding and the RandomForest edge weight estimator, which presented the best results for the anomaly detection model construction.

**Table 2:** Edge weight estimator performance based on different models

Weight estimator	MSE
LightGBM	**0.0016**
RandomForest	0.0029
XGBoost	0.0020

**Table 3:** Average metrics for different weight estimators base models when used to detect anomalous nodes. Numbers in bold indicate the highest value obtained for each metric

Weight Estimator	AUC	PR-AUC	P@1	P@3	P@10	P@20
LightGBM	0.64	0.03	0.18	0.14	0.12	0.08
RandomForest	**0.67**	**0.06**	**0.53**	**0.37**	**0.25**	**0.21**
XGBoost	0.60	0.01	0.00	0.00	0.01	0.02

We turned to check whether the edge weight estimator could provide biological insights (Methods). For each tissue network, we created a PPI subnetwork that only contained edges with a high difference between predicted and actual values (Fig. 1, step 3). Within each subnetwork, we labeled the tissue-associated disease proteins, namely proteins associated with Mendelian diseases that manifest in that tissue. We hypothesized that proteins that appear on paths between tissue-associated disease proteins are likely to have central roles in the tissue. Hence, we counted how many times each protein appeared on a path connecting two tissue-associated disease proteins. We focused on the top-10 proteins per network that participated in the largest number of such paths.

**Figure 2:**
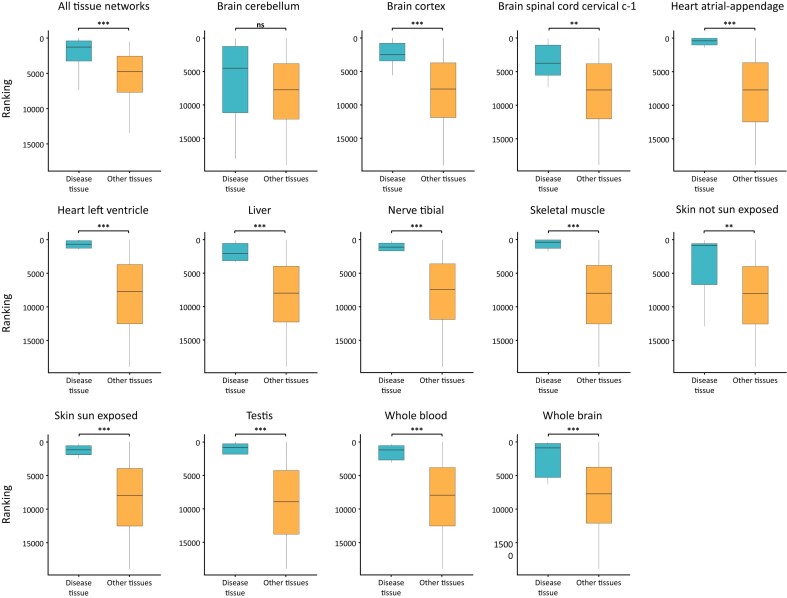
Using edge weight estimation errors to identify tissue-associated disease proteins. The 10 proteins that were most frequently on paths connecting tissue-associated disease proteins were significantly more likely to be tissue-associated disease proteins in that tissue (blue) than in other tissues (orange; $p-value=7.57e-6$, Wilcoxon test). Top-10 proteins were collated from 13 tissue networks. Adjusted *P*-values for cerebellum, cortex, spinal cord, heart atrial appendage, heart left ventricle, liver, nerve tibial, skeletal muscle, skin not sun-exposed, skin sun-exposed, testis, whole blood, and whole brain in respective order: 0.13, 8.14 × 10^−4^, 8.12 × 10^−3^, 2.75 × 10^−6^, 2.46 × 10^−6^, 9.21 × 10^−4^, 3.85 × 10^−5^, 1.85 × 10^−3^, 9.83 × 10^−5^, 3.11 × 10^−5^, 1.16 × 10^−5^, and 8.6 × 10^−4^ (one-sided Mann–Whitney test, Benjamini–Hochberg correction).

We used the TRACE tool [17] to rank these proteins by their likelihood of being tissue-associated disease proteins in the given tissue and in other tissues. The top-10 proteins were ranked significantly higher in the given tissue relative to their median ranking in other tissues (*P* = 7.57 × 10^−6^; Wilcoxon test; Fig. 2). In addition, except for brain cerebellum tissue, the top-10 proteins ranked significantly higher relative to other proteins of the same tissue (adjusted *P* ≤ 8.12 × 10^−3^, Mann–Whitney test, Benjamini–Hochberg correction; Fig. 2). Taken together, these results show that the top-10 proteins were not randomly picked but could potentially have tissue-specific roles. Overall, this analysis suggests that we could identify anomalous nodes based on the error in predicting their edge weight.

### The ensemble anomaly detection method outperforms other methods in detecting anomalous nodes

**Figure 3:**
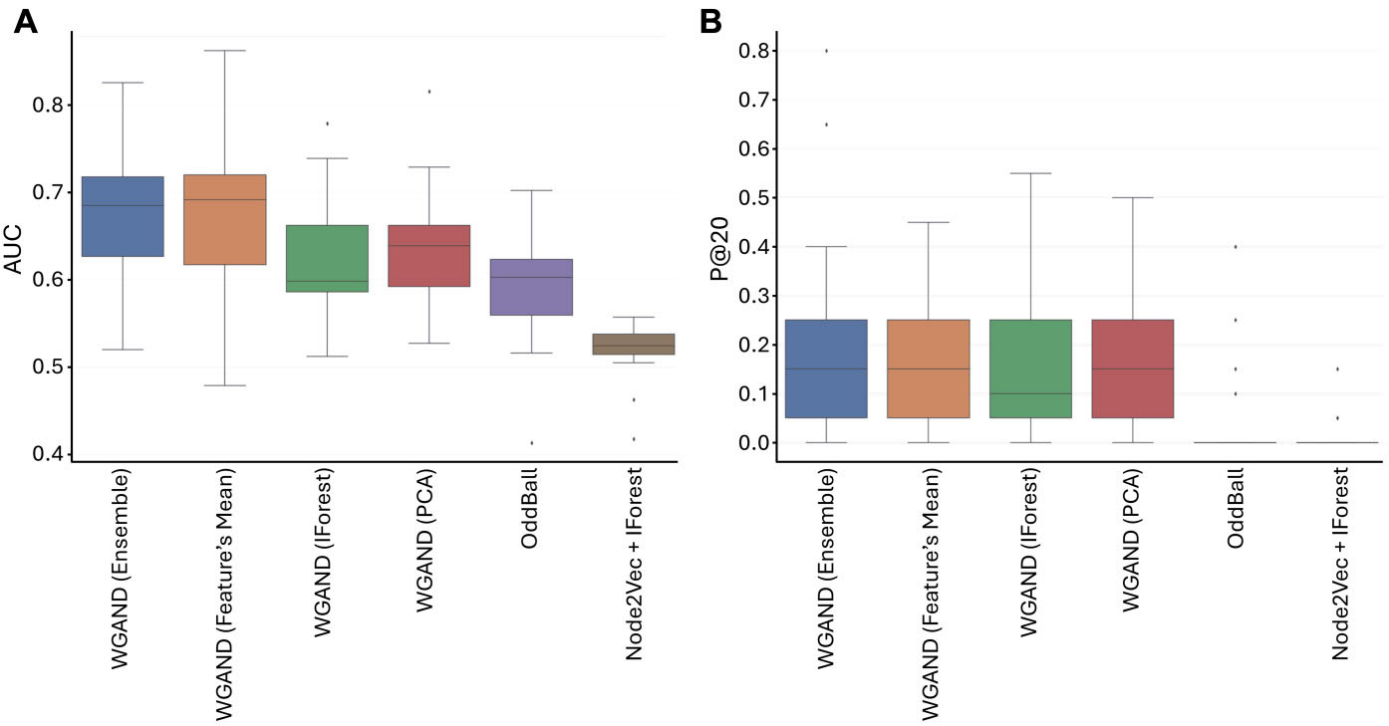
Evaluation of different classifiers using AUC (A) and P@20 (B) measures to predict anomalous nodes based on the meta-features.

**Figure 4:**
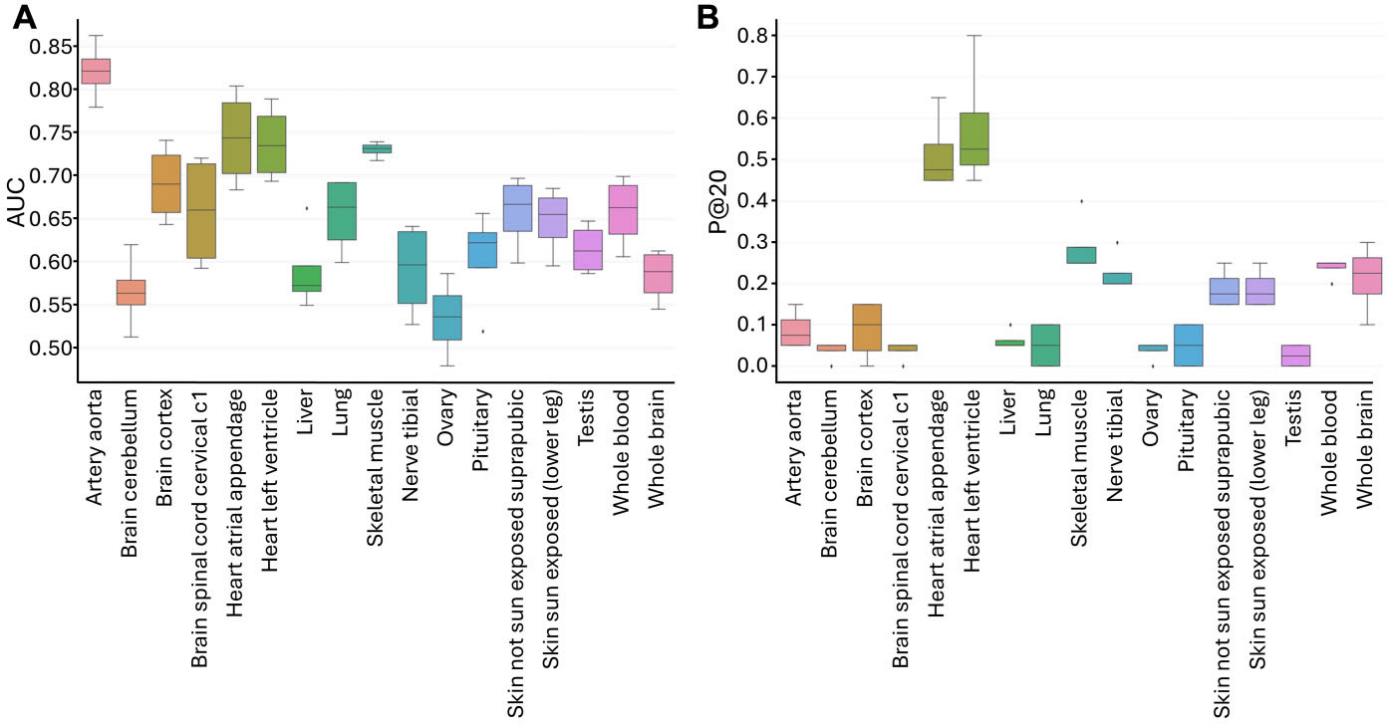
Evaluation of the variance of the four methods for predicting anomalous nodes for each tissue using AUC (A) and P@20 (B). Most tissues exhibit high variance in performance between different methods.

Next, we aimed to construct an unsupervised machine-learning-based anomaly detector model to detect anomalous nodes. For each tissue PPI network, we assigned nodes with 10 meta-features constructed based on the edge weight prediction error (Methods, Fig. 1, step 4). Then, using the edges’ actual weights, we evaluated the performance of four suggested anomaly detector models and compared them to two baseline models (Methods). On average, all four anomaly detector models present superior performance over the baseline models in all metrics with respect to AUC and P@K (Fig. 3). In terms of AUC, the ensemble and the feature’s mean presented the best performance with a marginal difference. In terms of P@K, we saw similar performance between the ensemble, PCA, and the feature’s mean with a slight advantage for the ensemble. In addition, we evaluated the performance of the ensemble method against the baseline models of each tissue network separately. The ensemble method had a higher AUC than the baselines across all tissues, higher PR-AUC in 16 out of 17 tissues, and higher P@20 in 13 out of 17 (Supplementary Table S1). Overall, the ensemble method—“WGAND (ensemble)”—performed best. For 85% of the datasets, the proposed method achieved higher performance than the baselines in terms of AUC and P@K. Fig. 4 and Supplementary Fig. S1 show significant variance between the different methods in most tissues. Furthermore, we observed some tissues, such as brain spinal cord cervical c1, where the anomaly detector achieves high AUC but relatively lower P@K values (Supplementary Table S1).

Next, to inspect which feature could explain the anomaly best and may be used as an indicator in unlabeled networks, we used the ”WGAND (ensemble)” method and constructed a model for each feature. The models were evaluated based on their P@K values (Supplementary Fig. S2). Most features had P@K higher than random in all the tissue networks (Supplementary Table S2). The features with the best performance on average were the sum of errors, error standard deviation, mean error, and mean absolute error.

### WGAND predicts anomalous proteins with major biological roles

We hypothesized that tissue-associated anomalous proteins would play major biological roles in the respective tissue. Examples of major biological roles include involvement in a tissue-associated disease (e.g., Duchenne muscular dystrophy in skeletal muscle) or in a biological process that is highly active specifically in that tissue (e.g., neuron signaling in brain tissue). Notably, we do not assume that an anomalous node necessarily causes a tissue-associated disease or a certain biological process, only that it is associated with them and thus appears different in the networks. We tested this hypothesis for the top-10 most likely anomalous proteins for each tissue network according to the best-performing combination “WGAND (ensemble).”

**Figure 5:**
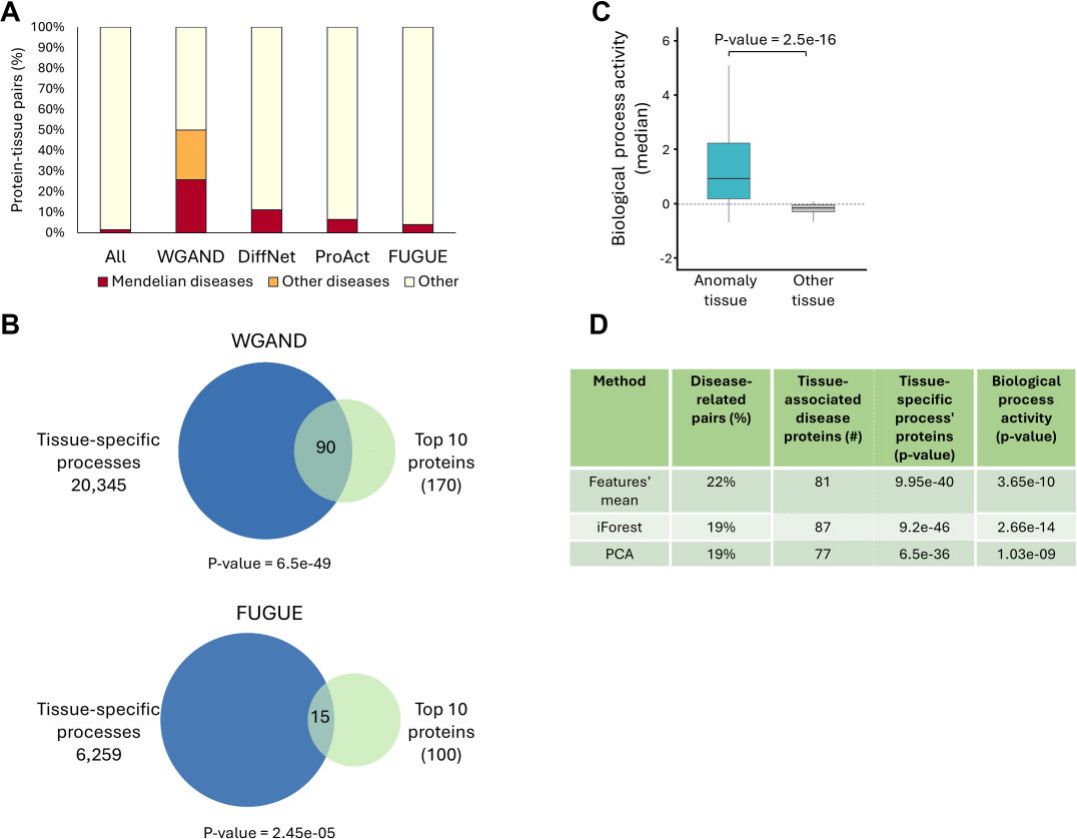
Top-10 anomalous proteins were enriched for tissue-specific diseases and cellular processes. (A) The percentage of protein–tissue pairs where the protein was associated with a Mendelian disease affecting that tissue (red). The percentage of such pairs out of all possible pairs was 1.5% (All), which was lower than their percentage among the top-10 pairs ranked by WGAND (ensemble) (26%), DiffNet (11%), ProAct (6%), and FUGUE (4%). The top-10 pairs ranked by WGAND were also enriched for proteins involved in other tissue-selective diseases and phenotypes (24%, orange). (B) The overlap between the top-10 protein–tissue pairs and pairs where the protein was associated with a biological process specific to that tissue. The number of such pairs appears in parentheses. Pairs ranked by WGAND were more significantly enriched than pairs ranked by FUGUE (Fisher’s exact test). (C) Top-10 proteins per tissue were more likely associated with cellular processes that were preferentially active in that tissue (blue) relative to other tissues (grey; *P* = 2.5 × 10^−16^, Mann–Whitney). (D) Analyses in panels (A)–(C) were applied to the top-10 anomalous proteins detected by three other WGAND methods, including the features’ mean, IForest, and PCA. The results were significant yet weaker than the ensemble method.

To test for involvement in tissue-associated diseases, we first assessed the association of the top-10 anomalous proteins with tissue-selective Mendelian diseases affecting the relevant tissue [25] (Methods). We found that 26% of the top-10 anomalous proteins per tissue (a total of 170 proteins) were indeed tissue-associated diseases proteins (Fig. 5A). To assess whether this percentage is expected by chance, we assessed it for all proteins in our networks. We found that only 1.5% of all proteins were tissue-associated disease proteins, a 17-fold decrease relative to the top-10 anomalous proteins. To go beyond Mendelian diseases, we manually mined the literature and disease-related databases for additional associations between the top-10 proteins per tissue and non-Mendelian diseases and phenotypes affecting the same tissue. This revealed that an additional 24% of the top-10 proteins were associated with diseases and phenotypes of that tissue (Fig. 5A and Supplementary Table S3). Hence, the top-10 anomalous proteins per tissue were highly enriched for proteins involved in diseases and phenotypes affecting the respective anomaly-relevant tissue.

Next, we compared WGAND to three other methods, including DiffNet [18], ProACT [19], and FUGUE [7] (Methods). All three methods had been tested previously for their ability to detect tissue-associated disease proteins. Hence, for each tissue, we ranked proteins according to each method. Then, we checked how many of the top-10 highly ranked proteins were tissue-associated disease proteins. According to DiffNet, ProAct, and FUGUE, only 11%, 6%, and 4%, respectively, were tissue-associated diseases proteins in the respective tissue, compared with 24% for WGAND (Fig. 5A). Hence, WGAND outperformed all three other methods.

To test the involvement of anomalous proteins in tissue-associated biological processes, we checked how many of the top-10 anomalous proteins were annotated as participating in a biological process that was specific to that tissue (Methods). WGAND had a larger and more significant overlap (Fig. 5B). We extended this analysis to include proteins that were involved in processes that were not necessarily tissue specific but rather preferentially active in the respective tissue by using ProAct scores [19]. Notably, the top-10 anomalous proteins, according to WGAND, were also more likely involved in such processes relative to their score in other tissues (Fig. 5C; *P* = 2.5 × 10^−16^, Mann–Whitney test). Finally, we compared “WGAND ensemble” to three other WGAND methods, including the feature’s mean, IForest, and PCA. WGAND ensemble performed best in all tests (Fig. 5D). We also checked whether the results were specific to the top-10 proteins by checking for the top-5, top-15, and top-20 proteins. In all cases, WGAND performed better that all other methods (Supplementary Fig. S4).

To demonstrate the value of our method, we describe tissue-relevant proteins that were indeed ranked as anomalous by ”WGAND (ensemble)” in the respective tissue, supporting our hypothesis. Notably, these proteins were not part of the positive set used to train WGAND. TNNT2 and TNNT3 are members of the troponin protein complex that initiates the process of muscle contraction. Both proteins encode the troponin T subunit of the complex. TNNT2 encodes the cardiac type of troponin T and was indeed ranked by WGAND as the seventh and sixth most anomalous node in the heart atrial appendage and left ventricle tissues, respectively. TNNT3 encodes the fast skeletal muscle type of troponin T and was indeed ranked by WGAND as the topmost anomalous node in skeletal muscle. Additionally, each of these proteins is associated with a disease affecting the anomaly-relevant tissue: TNNT2 is involved in cardiomyopathy (OMIM #191045, [26]), whereas TNNT3 is involved in Sheldon–Hall syndrome, a disorder that affects muscle and skeletal development before birth [27, 28]. Another example is the BGN protein, ranked third by WGAND in artery aorta tissue. BGN is associated with a rare genetic disorder called Meester–Loeys syndrome that is characterized by early-onset aortic aneurysm and dissection, leading to compromised structural integrity of the aorta and other large arteries [29]. Thus, the predicted anomalous state could indicate proteins involved in diseases and biological processes affecting the respective tissues.

## Discussion

We presented a novel machine-learning method for detecting abnormal nodes in weighted networks with positive and negative edge weights, which has important implications for various domains. Here, we demonstrated the effectiveness of our approach by applying it to PPI networks of different human tissues to identify anomalous proteins. By anomalous proteins, we refer not only to proteins associated with human diseases that manifest in specific tissues but also to proteins with major tissue-specific biological roles. Knowledge of such proteins could illuminate the molecular basis of human diseases, tissue phenotypes, and tissue-specific physiological processes. These goals were partially met by previous methods that relied on tissue transcriptomes [19], tissue-relevant networks [18], or methods that combine both, often using machine learning [7, 17]. Yet, the concept of network anomaly has not been used for this purpose.

We first demonstrated the relevance of network anomaly by showing that estimation errors of edge weights could be used to identify tissue-associated disease proteins (Fig. 2). Next, we took this approach further by devising a machine-learning method, WGAND, which was trained on features derived from estimation errors to predict anomalous proteins per tissue. Lastly, we used the predicted anomaly scores to rank anomalous proteins. We showed that the top-10 anomalous proteins overlapped significantly with proteins associated with diseases or phenotypes that affect the anomaly-relevant tissues, or with proteins with major tissue-specific roles. Moreover, WGAND outperformed other methods in detecting tissue-associated disease proteins and tissue-relevant proteins (Fig. 5). One caveat in our analyses is that the training data of disease- or tissue-associated proteins is incomplete. This implies that performance could be improved once more training data are available. It also implies that anomaly prediction scores could be used to pinpoint promising candidates for further analysis. Another potential bias in our analysis is that disease-associated proteins were more likely to be extensively studied than other proteins. Hence, their node degrees in our networks tend to be higher, contributing to their anomaly scores [30]. We controlled for this bias in two ways. Firstly, all tissue networks had the same topology and differed only in edge weights derived from unbiased transcriptomic data. Since anomaly scores were tissue specific, they were not simply affected by differences in protein degrees between disease-associated proteins and other proteins. Secondly, we showed that proteins with major tissue-specific roles beyond disease association were more likely to be an anomaly in the relevant tissue network. Specifically, we applied WGAND to predict tissue-relevant proteins participating in tissue-specific processes. These were derived either via manual curation or via an unbiased analysis of processes that were preferentially active in specific tissues based on transcriptomic data. Anomaly scores were assessed for each category. We found that high-ranking anomalous proteins were enriched not only for disease-associated proteins (Fig. 5A), but also for proteins involved in tissue-specific processes (Fig. 5B) and tissue preferentially active processes (Fig. 5C). Finally, we compared our results to FUGUE, which had the same goal as WGAND of predicting tissue-relevant proteins going beyond disease-associated proteins. We found that WGAND performed better than FUGUE (Fig. 5A,B).

We evaluated WGAND on weighted networks where edge weights can be positive and negative. WGAND can also be applied to other types of weighted networks, such as those with only positive or negative values. Moreover, WGAND can also be used for unweighted networks by converting the networks’ weights into binary values, where the existing edge will have a weight of 1 and the non-existing edge will have a weight of 0. WGAND could generate anomaly scores for all of these cases. We hope to evaluate this in future studies by analyzing additional types of networks.

We evaluate WGAND’s performance using AUC, P@K, and PR-AUC. When dealing with imbalanced data, P@K is critical, as a model with higher P@K can help experts narrow down the search space when inspecting anomalies. While the differences between various node embedding models are not highly significant, it is possible that the performance differences could be more pronounced in detecting anomalous nodes in different types of networks, such as social networks or computer networks. Additionally, the performance of the method may be improved by using a more systematic way to select the best node-embedding algorithm for a given task. Moreover, using hyperparameter optimization tuning and using frameworks such as Optuna [31] can also improve the algorithm’s performance. We plan to investigate this further in future studies by analyzing additional node embedding models and networks.

We found that “WGAND ensemble” performs significantly better than the baseline models in most tissues. OddBall outperformed WGAND in only four brain-related networks in terms of P@K and one network in terms of PR-AUC, but performed worse in terms of AUC (Supplementary Table S1). These results suggest that biological differences between brain tissues and other tissues may be a contributing factor to the observed performance differences. In future studies, one could explore the commonalities and differences between brain-related tissues further. We also noted that Node2Vec with IForest could not achieve good results with these data in all the tissues, suggesting they were not suited for the task.

We showed that a single meta-feature can be a good measure for node anomaly detection. On average, the sum of errors is the best predictive single feature across all metrics. Surprisingly, the absolute sum of errors is the worst feature across all metrics, despite our expectation that the error direction should not matter and that a higher total error should be a good indication of an anomaly. This result suggests that error direction is an important factor in node anomaly detection and that the difference between regular and absolute meta-features is inconsistent. In a future study, we would like to evaluate WGAND on different use cases, such as malicious user detection in online social networks. We would like to explore if the same type of anomaly detection features would continue to give the best performance in different domains.

Lastly, we investigated different methods for generating anomaly scores and extracting anomalies from the generated anomaly features and found that some methods have similar performance. Among the four proposed methods, the IForest-based solution performs the worst in terms of AUC, while Feature’s Mean has the best AUC. In terms of P@K, the differences are smaller, but different methods have advantages in different tissue networks. For example, the ensemble-based method has a significant advantage in the liver network, while the PCA-based and Feature’s Mean methods have a significant advantage in the lung network. Also, some tissues, such as skin not sun exposed suprapubic, heart left ventricle, and nerve tibial, had higher P@K when learning from a single feature than all of the features. While it is difficult to conclude which method is the best, a good rule of thumb would be first to try using the ensemble-based WGAND, which should combine the advantages of all other methods.

Currently, our search for anomalous nodes is limited to proteins with known PPIs. Future applications could extend the search for other types of genes, for example, by using coexpression networks [32] and to additional contexts, such as cell types [33].

## Methods and Experiments

In this section, we present the components of our generic anomaly detection framework and detail how its performance was tested and evaluated. We then describe the dataset of weighted, tissue-specific PPI networks utilized in this study. Following this, we explain the process of annotating tissue-associated disease proteins and tissue-specific biological processes. Next, we outline how the framework was employed to evaluate edge weight predictions based on their ability to identify tissue-associated disease proteins and how it was applied to detect anomalous nodes with tissue-specific roles. Lastly, we conclude the section by comparing the performance of WGAND (RRID:SCR_026407) in identifying tissue-associated disease proteins proteins with tissue-specific impacts to other tissue-specific ranking methods.

### Anomaly detection generic framework

Precisely defining what constitutes an anomaly is challenging [34] because definitions can vary across different domains and disciplines. Generally, anomalies are considered unusual observations that deviate significantly from the general population to the extent that they suggest a different mechanism may have created them [13]. In this study, we treated an anomaly as a node that behaves differently from most of the nodes in the network and interacts differently with its neighbors.

To formalize this idea, let us consider a weighted graph $G=(V,E,W)$, where *V* is the set of vertices, *E* is the set of edges, and *W* is the set of weights. Given a vertex *v*, we define its neighborhood $\Gamma (v)=\lbrace (v,u) \mid (v,u) \in E\rbrace$ as the set of vertices that are connected to *v* by an edge, and their weights as $W_v = \lbrace W_{v,u} \mid u \in \Gamma (v)\rbrace$, where $W_a$ and $W_n$ represent the edge weights between a suspected anomalous node *a* with its neighbors and a normal node *n* with its neighbors, respectively.

Extending our recent work [15], we hypothesized that $W_a$ should deviate from $W_n$ if *a* is truly anomalous. In other words, the average edge weights for *a* should differ from those of *n*. To test this hypothesis, we assumed that a general predictive function $P_f(v,u)$ exists, which can estimate the weight of an edge $(v,u)$ with high accuracy. Given this assumption, we expect that $|\hat{W}_{a,v} - W_{a,v}| > |\hat{W}_{n,v} - W_{n,v}|$ if *v* is a neighbor of *a* and *a* is genuinely anomalous. This is because the predicted weight $\hat{W}_{a,v}$ for the edge $(a,v)$ should deviate considerably from the actual weight $W_{a,v}$ if *a* is anomalous. In contrast, the predicted weight $\hat{W}_{n,v}$ for the edge $(n,v)$ should be close to the actual weight $W_{n,v}$ if *n* is normal.

Since most nodes and their edges are normal, it should be possible to train a model that learns to predict the values of normal interactions. A model that will learn to generalize well should successfully predict the normal values and fail to predict abnormal values, resulting in higher errors for these cases.

### Development of an edge weight estimator

We trained a model that estimates edge weights to create the predictive function $P_f(v,u)$. We used node embedding to generate features representing nodes *v* and *u* to train $P_f(v,u)$. Node embedding is a vector representation of nodes in a graph designed to capture the relationships between nodes [35]. Unlike structure-based feature sets that may be specific to certain types of networks, node embedding is a more general solution that can produce near-state-of-the-art performance without requiring additional handcrafted features.

We treated the network as an unweighted graph to generate the embedding vectors. (Some node embedding algorithms, such as node2vec [36], use the edge weights when building the embedding. Using embedding created from a weighted graph to train an edge weight estimator creates a data leakage that will affect the whole system’s performance.) In this study, to select the node-embedding method, we evaluated five types of embeddings: node2vec [36], RandNE [23], GLEE [37], NodeSketch [38], and DeepWalk [39, 40] based on the implementation by Rozemberczki et al. [40].

Using the extracted embeddings, we can train a weight estimator by using various regression algorithms and measure their performances using a variety of metrics, such as mean absolute error (MAE), root mean square error (RMSE), and MSE. In the study, we utilized three regressors: Random Forest [24], XGBoost (RRID:SCR_021361) [41], and LightGBM (RRID:SCR_021697) [42]. We measured the performance of the weight estimators using MSE. In addition, it is essential to note that during the training of the weight estimator, it is crucial to avoid considerable overfit. In this study, to detect anomalous proteins, we tuned the models’ parameters by limiting each tree’s maximum depth to reduce overfitting. Suppose the model overfits and “memorizes” the edge values like an infinitely large decision tree. In that case, it will be unable to identify nodes that deviate from the norm because there will be no deviations in the predictions.

### Construction of anomaly detection features

Anomaly detection features are used to identify nodes in the network that behave differently from the norm. Features are constructed to represent a node by aggregating the difference between the estimated and the actual edge weight of its edges. An anomalous node is likelier to have a larger average error, indicating that it behaves differently. The accumulation suggests that the deviation is not a result of the model error but something systematic.

Deviation from the norm can be measured in a variety of ways. Similarly to Kagan et al. [15], we have generated anomaly detection features based on basic statistics such as standard deviation, mean, etc. To create the anomaly detection features, we defined the error of an edge $(u,v)$ as $e_{u,v} = \hat{W}_{u,v} - W_{u,v}$ and the error of a node *v* as $e_v=\lbrace e_{u,v}\mid u \in \Gamma (v)\rbrace$. Based on these scores, we calculated 10 meta-features for each node in the network:

1. **Mean error:** The average error of the predicted weights of the interactions between node *v* and its neighbors: $ME(v):={\frac{{\sum _{\lbrace i\in \Gamma (v)\rbrace }}e_{v,i}}{\mid \Gamma (v) \mid }}$.
2. **Error standard deviation (error SD):** The dispersion of the predicted weights of the interactions between node *v* and its neighbors: $\sigma (e_v)$.
3. **Median error:** The median error of the predicted weights of the interactions between node *v* and its neighbors: $median(e_v)$.
4. **Sum of errors:** - The total sum of errors of the predicted weights of the interactions between node *v* and its neighbors: ${\sum _{\lbrace i\in \Gamma (v)\rbrace }}e_{i}$.
5. **Standard error of the mean (SEM):** Measures the average amount of error variation of the predicted weights of the interactions between node *v* and its neighbors:$\frac{\sigma (e_v)}{\sqrt{n}}$.
6. **Mean absolute error**: The average absolute error of the predicted weights of the interactions between node *v* and its neighbors: $MAE(v):={\frac{{\sum _{\lbrace i\in \Gamma (v)\rbrace }}\left|e_{v,i}\right|}{\mid \Gamma (v) \mid }}$.
7. **Absolute error standard deviation**: The dispersion of the absolute errors of the predicted weights of the interactions between node *v* and its neighbors $\sigma (\mid e_v \mid )$.
8. **Median absolute error:** The absolute median error of the predicted weights of the interactions between node *v* and its neighbors: $median(\mid e_v \mid )$.
9. **Sum of absolute errors:** The sum of absolute errors of the predicted weights of the interactions between node *v* and its neighbors: $\sum _{i \in \Gamma (v)}\mid e_{i}\mid$.
10. **Standard absolute error of the mean:** Measures the average amount of absolute error variation of the predicted weights of the interactions between node *v* and its neighbors: $\frac{\sigma (\mid e_v \mid )}{\sqrt{n}}$.

Using these features, we generate a 10-dimension vector for each node. In this study, to inspect the potential of the proposed features in predicting anomalies in PPI networks, we extracted the meta-features of the differential networks and treated them as a 10-dimension vector. Then, we used principal component analysis (PCA) to reduce the meta-features to a 2-dimensional vector. The PCA analysis revealed that the tissue-associated disease proteins are grouped together. This result indicates that the meta-features successfully identified proteins with major biological roles in a specific tissue and these proteins behave as anomalies (Supplementary Fig. S3).

### Detection of anomalous nodes

The main approach to search for anomalies is by ranking the nodes according to a specific anomaly detection feature, where nodes with the highest values are considered potential anomalies. However, determining the most suitable feature among the presented anomaly detection features can be challenging. To address this, we propose a variety of methods can be four methods combining the generated anomaly detection features into one anomaly score.

1. **Feature’s Mean**—uses a sigmoid function to normalize the values of each anomaly detection feature, then assigns each node with the mean normalized values of the features. This process produces a single value that can be used as the anomaly score.
2. **PCA**—reduces the dimensionality of the anomaly detection features. The resulting component value can be used as the anomaly score [43].
3. **Isolation Forest (IForest)**—uses anomaly detection features as the IForest [44] model input to generate an anomaly score.
4. **Ensemble**—combines the previous three methods. Here, the raw Feature’s Mean, PCA values, and also their values after a sigmoid function were inserted into an IForest model to generate the anomaly score. Then, we select the maximum value from this new anomaly score and the scores produced from the first two methods.

### Evaluation scheme

To assess the effectiveness of our proposed method and evaluate anomaly detection performance, a variety of metrics can be used, such as the models’ AUC [45], PR-AUC [46], and P@K, where K refers to the number of top-ranked records (nodes) to consider. AUC and PR-AUC are commonly used metrics to estimate the overall performance of a classifier [45, 46]. P@K is particularly useful in anomaly detection tasks, where the goal is to discover new items and recommend and prioritize a subset of predictions with the highest confidence scores.

To evaluate our method in identifying abnormal nodes in PPI networks, in this study, we utilized the AUC, PR-AUC, and P@K metrics. Based on these metrics, we first compared the performance of five different node-embedding models on weighted networks. Secondly, using the best-performing embedding model, we compared the performance of three edge weight estimator models, the performance of which was also measured by MSE. Thirdly, we used the best-performing combination of node-embedding and edge weight estimator models and evaluated the performance of four different anomaly detection methods.

To evaluate the performance of the proposed method on the PPI networks, and compare it to other node anomaly detection methods, we used two baseline methods:

1. **Node2Vec + IForest:** Similarly to Lee et al. [12], we used node2vec combined with the IForest model. Here, we generated the embeddings based on the weights of the graph edges via node2vec. Then, we utilized these embeddings as the features for the IForest model.
2. **OddBall**: We used the OddBall algorithm [13] for anomaly detection in weighted graphs. To the best of our knowledge, OddBall is the only available algorithm with an implementation (https://github.com/gloooryyt/oddball_py3) that was specifically designed for anomaly detection in weighted graphs.

### Dataset of weighted tissue-specific PPI networks

In this study, we tested and evaluated our method on data of 17 PPIs networks downloaded from Basha et al. [47] (Table 4). Each network was composed of 13,523 proteins (nodes) and 134,223 PPIs (edges). The weight of an interaction relied on the expression of the interacting genes in the given tissue relative to their expression in other tissues [47]. Weights ranged between $[-1,1]$, where positive scores imply that the protein interaction was more likely to occur in that tissue relative to other tissues, and negative scores imply that it is less likely to occur.

**Table 4:** PPI network datasets

Tissue	No. of tissue-associated disease proteins nodes (%)
Artery aorta	23 (0.17)
Brain cerebellum	65 (0.48)
Brain cortex	77 (0.57)
Brain spinal cord cervical c1	48 (0.35)
Heart atrial appendage	133 (0.98)
Heart left ventricle	138 (1.02)
Liver	53 (0.39)
Lung	274 (2.03)
Muscle skeletal	121 (0.89)
Nerve tibial	77 (0.57)
Ovary	41 (0.3)
Pituitary	23 (0.17)
Skin not sun exposed suprapubic	125 (0.92)
Skin sun exposed lower leg	125 (0.92)
Testis	101 (0.75)
Whole blood	442 (3.3)
Whole brain	564 (4.2)

### Annotation of tissue-associated disease proteins and tissue-specific biological processes

Data of Mendelian diseases, disease proteins, and disease-affected tissues were obtained from ODiseA [25]. Nodes of a given tissue PPI network were labeled as tissue-associated disease proteins if the corresponding protein was associated with a Mendelian disease that affected that tissue. As shown in Table 4, up to 4.2% of the nodes per network were labeled as tissue-associated disease proteins. Data of proteins involved in additional tissue-selective diseases and phenotypes were obtained from PubMed [48], GeneCards [49], and HPO [50] databases via manual curation. Data of proteins participating in tissue-specific biological processes were obtained from Basha et al. [47].

### Protein anomaly detection model

We tested whether the anomaly detection algorithm could be used to identify proteins with major biological roles in the tissue where they appear anomalous. We considered a protein to have major biological roles in the tissue if it was (i) designated as tissue-associated disease protein in that tissue according to ODiseA [25]; (ii) was involved in an additional tissue-selective disease or phenotype, according to manual search; (iii) participated in a biological process that was specific to that tissue according to Basha et al. [47]; or (iv) participated in a biological process that was more active in that tissue relative to other tissues, as estimated by ProAct [19]. We applied WGAND to each tissue-specific PPI network and focused on the top-10 anomalous nodes per network. To test for (i), we compared the fraction of tissue-associated disease proteins among the top-10 anomalous nodes relative to that fraction among all other nodes. To test for (ii), we performed a manual search. To test for (iii), we computed the fraction of top-10 proteins that participated in a tissue-specific biological process out of the total number of proteins participating in such processes in the given tissue and assessed for enrichment using Fisher’s exact test. To test for (iv), we compared the estimated biological process activities of the top-10 anomalous proteins in the given tissue to their estimated activities in all other tissues using the Mann–Whitney test.

### Comparison of the WGAND anomaly detection method to other methods

We compared the performance of WGAND in revealing tissue-associated disease proteins that affect a specific tissue to three other tissue-specific ranking schemes. Firstly, we compared WGAND with a ranking of proteins by DiffNet [18]. Specifically, for each tissue network, we associated each protein with the median differential score of its interactions and then considered the top-10 proteins with the highest score. Secondly, we compared WGAND with a ranking of proteins by ProAct [19]. Specifically, for each tissue, we associated each protein with median ProAct score of the processes in which it participates, and then considered the top-10 proteins with the highest score. Thirdly, we compared WGAND with the top-10 tissue-relevant proteins according to FUGUE [7]. We downloaded FUGUE scores from Somepalli et al. [7]. Scores were limited to proteins and tissues that WGAND also scored, resulting in 10 tissues that were common to both methods. For each method, we computed for each tissue the fraction of tissue-associated disease proteins out of the top-10 proteins that were associated with a Mendelian disease that affected that tissue. For FUGUE, we also tested whether the top-10 tissue-relevant proteins per tissue participated in a biological process specific to that tissue, as described in the preceding subsection. All tests were repeated for the top 5, 15, and 20 proteins per tissue (Supplementary Fig. S4).

## Availability of Source Code and Requirements

- Project name: WGAND (RRID:SCR_026406)
- Project homepage: https://github.com/data4goodlab/wgand
- Operating system(s): platform independent
- Programming language: Python 3.6 or higher
- Other requirements: pandas, combo, tqdm, pyod, scikit-learn, numpy, networkx, karateclub
- License: GPL 3.0
- Reference: Version 1 [21]

## Supplementary Material

giaf034_Supplemental_File

giaf034_GIGA-D-24-00363_Original_Submission

giaf034_GIGA-D-24-00363_Revision_1

giaf034_GIGA-D-24-00363_Revision_2

giaf034_Response_to_Reviewer_Comments_Original_Submission

giaf034_Response_to_Reviewer_Comments_Revision_1

giaf034_Reviewer_1_Report_Original_Submission Yong Zhang -- 10/16/2024

giaf034_Reviewer_2_Report_Original_Submission Dan Shao -- 10/22/2024

## Data Availability

The following datasets and algorithms were utilized in this study: DiffNet [18], ProAct [19], TRACE [17], ODiseA [25], and FUGUE [7]. Supporting data for this study can be found in the GigaScience GigaDB [51].

## Additional Files

**Supplementary Fig. S1:** P@K of different classifiers per each PPI tissue-specific network.

**Supplementary Fig. S2:** P@K of “WGAND ensemble” method trained with each feature separately on PPI tissue-specific networks.

**Supplementary Fig. S3:** PCA representation of the anomaly detection features. The red dots represent tissue-associated disease proteins; the blue dots represent all other proteins.

**Supplementary Fig. S4:** WGAND shows robust performance for the top-K proteins for different values of K. Top anomalous proteins show consistent enrichment for tissue-aware disease proteins, tissue-specific biological processes, and biological process activity in the anomaly-relevant tissue, as described in Fig. 5 of the main text for top-10 proteins. (a) Top-5 proteins. (b) Top-15 proteins, and (c) Top-20 proteins.

**Supplementary Table S1:** Evaluation of WGAND versus baselines on PPI dataset.

**Supplementary Table S2:** P@K of “WGAND ‘ensemble” method constructed per feature.

**Supplementary Table S3:** The top-10 genes predicted by the ensemble methods and the diseases that these genes were related to.

## Abbreviations

AUC: area under curve; IForest: Isolation Foresrt; MAE: mean absolute error; MSE: mean squared error; P@K: precision at K; PPI: protein–protein interaction; SD: standard deviation; SEM: Standard error of the mean; PCA: principal component analysis; WGAND: Weighted Graph Anomalous Node Detection.
