# Supplementary material for: Network-based anomaly detection algorithm reveals proteins with major roles in human tissues
Source: Gigascience. 2025 Apr 8;14:giaf034. doi: 10.1093/gigascience/giaf034 (PMC11976396; doi:10.1093/gigascience/giaf034)
Supplement: giaf034_Supplemental_File [file giaf034_supplemental_file.pdf]

Supplementary Materials

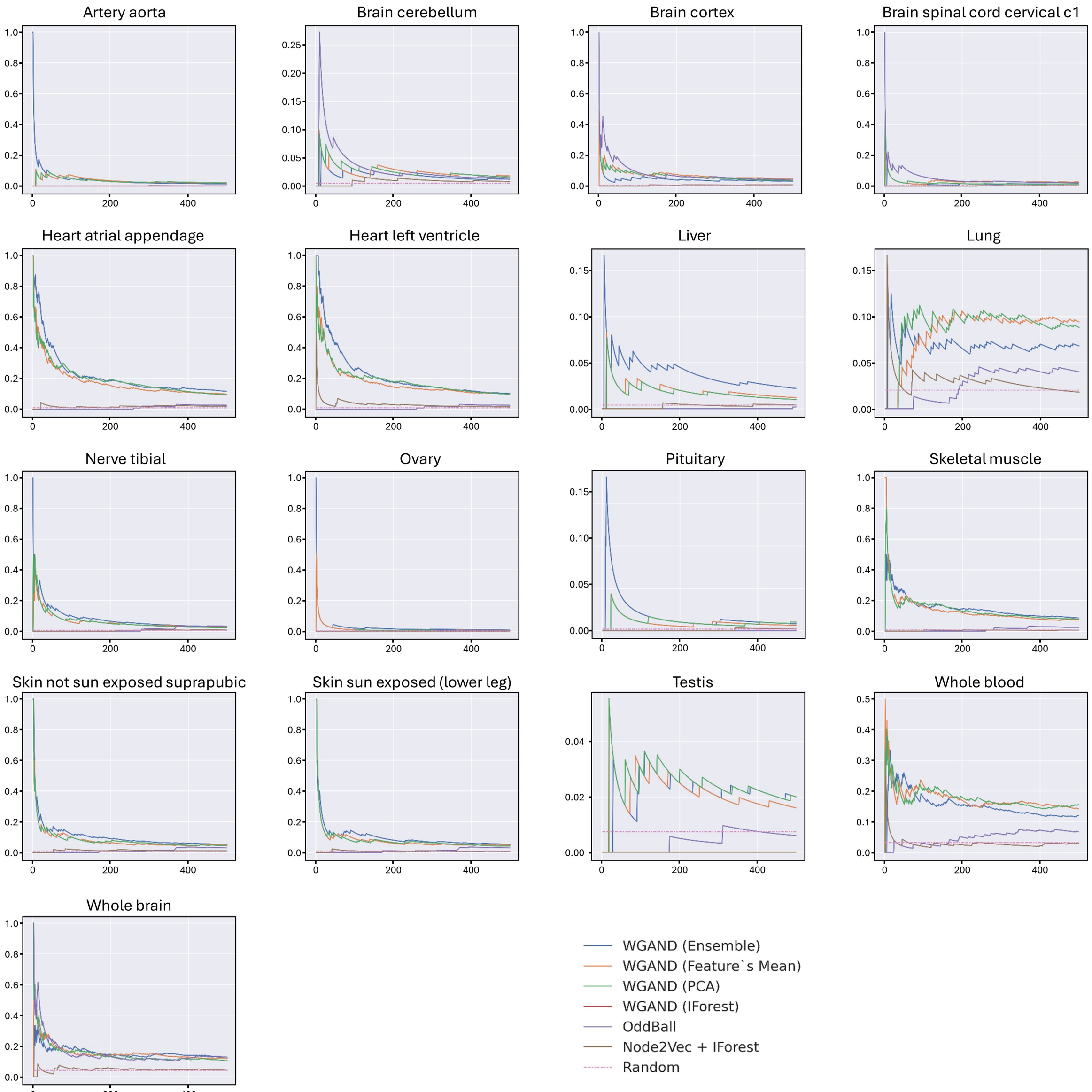

Figure S1. P@K of different classifiers per each PPI tissue-specific network.

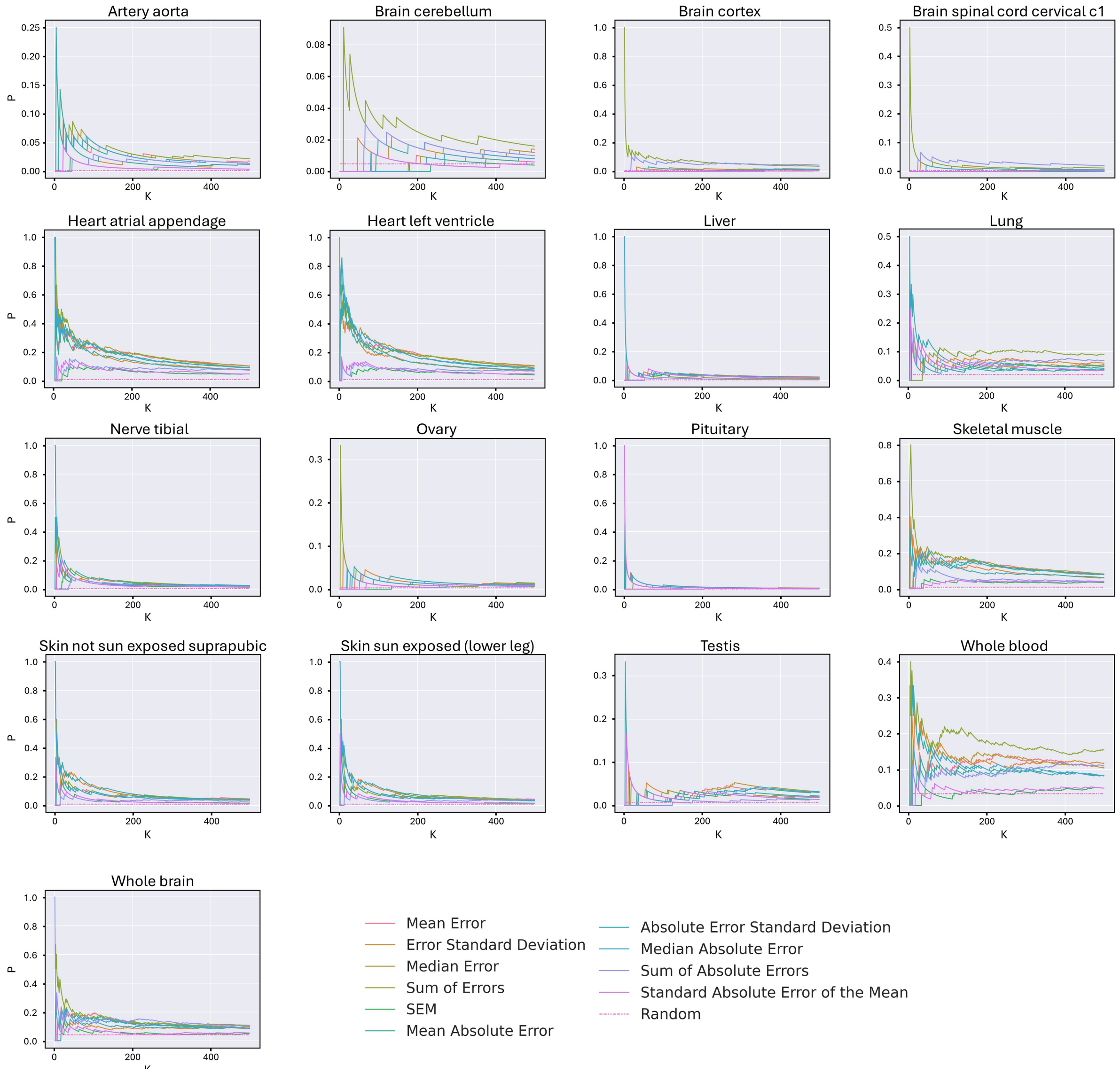

Figure S2. P@K of WGAND 'ensemble' method trained with each feature separately on PPI tissue-specific networks.

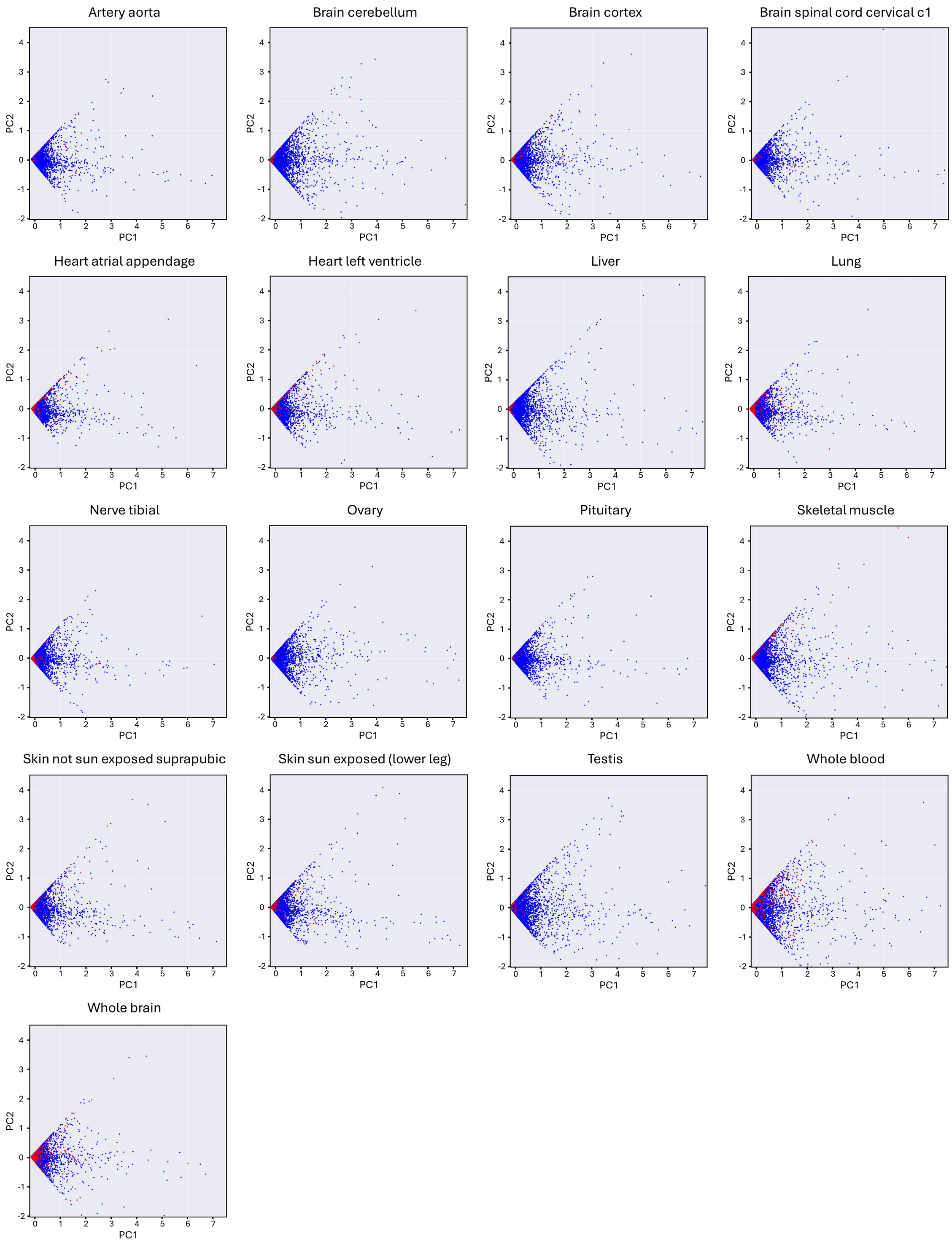

Figure S3. PCA representation of the anomaly detection features. The red dots represent tissue-associated disease proteins; the blue dots represent all other proteins.

A. Top 5 predicted genes

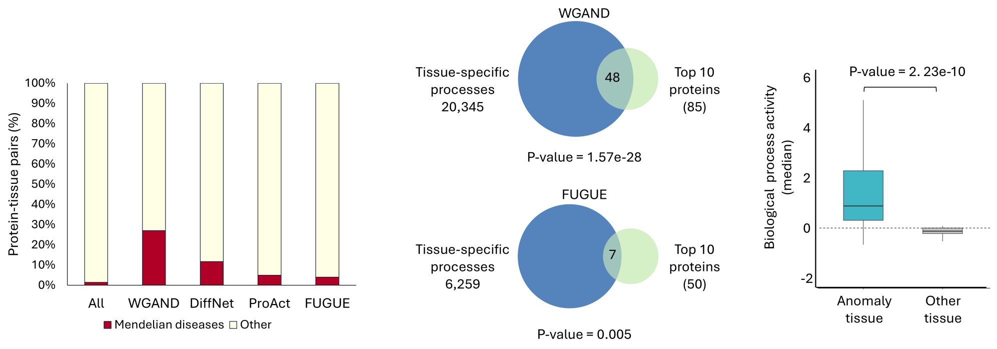

Method	Disease-related pairs (%)	Tissue-associate d disease proteins (#)	Tissue-specific process' proteins (p-value)	Biological process activity (p-value)
Features' mean	31%	46	2.56e-26	6.53e-07
iForest	22%	44	3.47e-24	3.12e-8
PCA	28%	44	3.47e-24	5.08e-08

B. Top 15 predicted genes

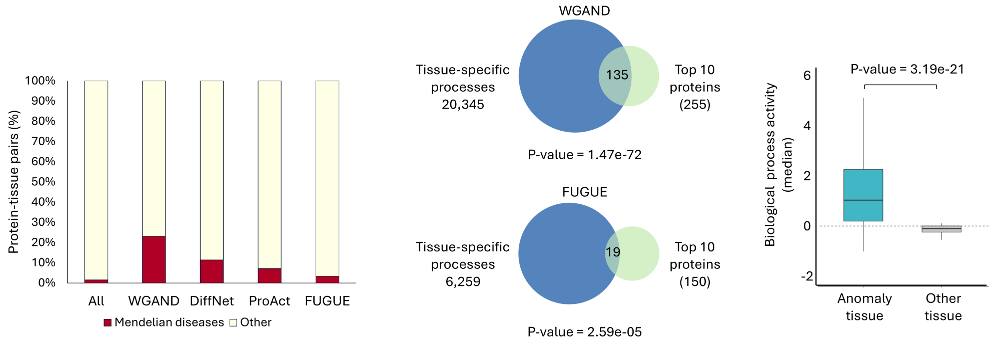

Method	Disease-related pairs (%)	Tissue-associated disease proteins (#)	Tissue-specific process' proteins (p-value)	Biological process activity (p-value)
Features' mean	19%	117	1.87e-54	2.42e-13
iForest	17%	124	3.12e-61	1.52e-20
PCA	17%	114	1.9e-51	4.05e-12

C. Top 20 predicted genes

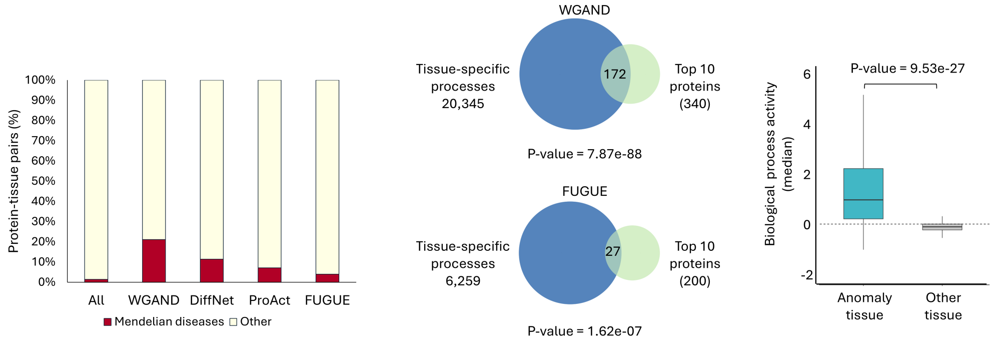

Method	Disease-related pairs (%)	Tissue-associated disease proteins (#)	Tissue-specific process' proteins (p-value)	Biological process activity (p-value)
Features' mean	15%	150	1.77e-66	1.62e-14
iForest	16%	162	8.11e-78	6.34e-26
PCA	16%	144	4.35e-61	1.11e-16

Figure S4. WGAND shows robust performance for the top-k proteins for different values of K. Top anomalous proteins show consistent enrichment for tissue-aware disease proteins, tissue-specific biological processes, and biological process activity in the anomaly-relevant tissue, as described in Fig. 5 of the main text for top-10 proteins. (a) Top-5 proteins. (b) Top-15 proteins, and (c) Top-20 proteins.

Table S1. Evaluation WGAND versus baselines on PPI dataset

Tissue Network	WGAND (Ensemble)						Node2Vec + Iforest						Oddball					
	AUC	PR-AUC	P@1	P@3	P@10	P@20	AUC	PR-AUC	P@1	P@3	P@10	P@20	AUC	PR-AUC	P@1	P@3	P@10	P@20
Artery Aorta	**0.83**	**0.11**	**1.00**	**0.67**	**0.20**	**0.15**	0.46	0.00	0.00	0.00	0.00	0.00	0.70	0.00	0.00	0.00	0.00	0.00
Brain Cerebellum	**0.56**	**0.01**	0.00	0.00	0.00	0.05	0.52	**0.01**	0.00	0.00	0.00	0.00	0.53	**0.01**	0.00	0.00	**0.20**	**0.15**
Brain Cortex	**0.72**	**0.02**	0.00	**0.33**	0.10	0.05	0.53	0.01	0.00	0.00	0.00	0.00	0.68	**0.05**	**1.00**	**0.33**	**0.40**	**0.25**
Brain Spinal cord cervical c 1	**0.71**	**0.01**	0.00	0.00	0.10	0.05	0.52	0.00	0.00	0.00	0.00	0.05	0.70	**0.04**	**1.00**	**0.33**	**0.20**	**0.10**
Heart Atrial Appendage	**0.78**	**0.18**	**1.00**	**1.00**	**0.70**	**0.65**	0.56	0.01	0.00	0.00	0.00	0.00	0.63	0.03	0.00	0.00	0.00	0.00
Heart Left Ventricle	**0.76**	**0.18**	**1.00**	**1.00**	**0.90**	**0.80**	0.54	0.02	0.00	0.00	0.00	0.00	0.62	0.03	0.00	0.00	0.00	0.00
Liver	**0.55**	**0.01**	0.00	0.00	0.00	**0.10**	0.42	0.00	0.00	0.00	0.00	0.00	0.41	0.00	0.00	0.00	0.00	0.00
Lung	**0.69**	**0.05**	**0.00**	**0.00**	**0.10**	**0.10**	0.52	0.02	0.00	0.00	0.10	0.05	0.60	0.03	0.00	0.00	0.00	0.00
Muscle Skeletal	**0.73**	**0.09**	**1.00**	**0.67**	**0.50**	**0.40**	0.54	0.01	0.00	0.00	0.00	0.00	0.56	0.02	0.00	0.00	0.00	0.00
Nerve Tibial	**0.63**	**0.05**	**1.00**	**0.33**	**0.30**	**0.30**	0.51	0.01	0.00	0.00	0.00	0.00	0.59	0.01	0.00	0.00	0.00	0.00
Ovary	**0.52**	**0.03**	**1.00**	**0.33**	**0.10**	**0.05**	0.51	0.00	0.00	0.00	0.00	0.00	0.52	0.00	0.00	0.00	0.00	0.00
Pituitary	**0.63**	**0.01**	**0.00**	**0.00**	**0.20**	**0.10**	0.52	0.00	0.00	0.00	0.00	0.00	0.53	0.00	0.00	0.00	0.00	0.00
Skin Not Sun Exposed Suprapubic	**0.69**	**0.06**	**1.00**	**0.67**	**0.30**	**0.20**	0.54	0.01	0.00	0.00	0.00	0.00	0.62	0.02	0.00	0.00	0.00	0.00
Skin Sun Exposed Lower leg	**0.67**	**0.05**	**1.00**	**0.67**	**0.30**	**0.20**	0.52	0.01	0.00	0.00	0.00	0.00	0.62	0.02	0.00	0.00	0.00	0.00
Testis	**0.63**	**0.01**	**0.00**	**0.00**	**0.00**	**0.00**	0.51	**0.01**	0.00	0.00	0.00	0.00	0.57	**0.01**	0.00	0.00	0.00	0.00
Whole Blood	**0.69**	**0.08**	**1.00**	**0.33**	**0.20**	**0.25**	0.54	0.04	0.00	0.00	0.10	0.15	0.61	0.06	0.00	0.00	0.00	0.00
Whole Brain	**0.61**	0.07	0.00	0.33	0.30	0.20	0.53	0.04	0.00	0.00	0.00	0.00	0.56	**0.08**	**1.00**	**0.67**	**0.50**	**0.40**
Average	**0.67**	**0.06**	**0.53**	**0.37**	**0.25**	**0.21**	0.52	0.01	0.00	0.00	0.01	0.01	0.59	0.02	0.18	0.08	0.08	0.05

Table S2. P@K of WGAND ‘ensemble’ method constructed per feature.

Tissue Network	Mean Error				Error Standard Deviation				Median Error			
	P@1	P@3	P@10	P@20	P@1	P@3	P@10	P@20	P@1	P@3	P@10	P@20
Artery Aorta	0.00	0.00	**0.10**	**0.10**	0.00	0.00	**0.10**	0.05	0.00	0.00	0.00	0.05
Brain Cerebellum	0.00	0.00	0.00	0.00	0.00	0.00	0.00	0.00	0.00	0.00	0.00	0.00
Brain Cortex	0.00	0.00	0.00	0.00	0.00	0.00	0.00	0.00	0.00	0.00	0.00	0.00
Brain Spinal cord cervical c 1	0.00	0.00	0.00	0.00	0.00	0.00	0.00	0.00	0.00	0.00	0.00	0.00
Heart Atrial Appendage	**1.00**	0.33	0.20	0.35	0.00	0.33	0.40	0.40	**1.00**	0.67	0.40	0.35
Heart Left Ventricle	0.00	**0.67**	0.60	0.50	0.00	0.33	0.40	0.40	0.00	**0.67**	0.70	**0.55**
Liver	**1.00**	**0.33**	**0.10**	**0.05**	0.00	0.00	0.10	**0.05**	**1.00**	**0.33**	**0.10**	0.05
Lung	0.00	**0.33**	0.10	0.05	0.00	**0.33**	0.10	0.10	0.00	**0.33**	0.10	0.05
Muscle Skeletal	0.00	0.00	0.00	0.15	0.00	0.00	0.30	0.20	0.00	0.00	0.00	0.15
Nerve Tibial	**1.00**	**0.67**	0.20	0.10	0.00	0.00	0.20	0.15	**1.00**	**0.67**	**0.20**	0.10
Ovary	0.00	0.00	0.00	0.00	0.00	0.00	**0.10**	**0.05**	0.00	0.00	0.00	0.00
Pituitary	0.00	0.00	**0.10**	**0.10**	0.00	**0.33**	**0.10**	0.05	0.00	0.00	**0.10**	**0.10**
Skin Not Sun Exposed Suprapubic	**1.00**	**0.67**	**0.30**	0.20	0.00	0.00	0.10	0.20	**1.00**	**0.67**	**0.30**	0.20
Skin Sun Exposed Lower leg	**1.00**	**0.67**	**0.40**	**0.25**	0.00	0.33	0.10	0.15	**1.00**	**0.67**	**0.40**	**0.25**
Testis	0.00	0.00	0.00	0.00	0.00	0.00	0.00	**0.05**	0.00	0.00	0.00	0.00
Whole Blood	0.00	0.00	0.10	0.05	0.00	0.00	0.20	0.20	0.00	0.00	0.10	0.10
Whole Brain	0.00	0.33	0.10	0.15	0.00	0.00	0.10	0.10	0.00	0.33	0.10	0.20
Average	0.29	0.24	0.14	0.12	0.00	0.10	0.14	0.13	0.29	0.25	0.15	0.13

Tissue Network	Sum of Errors				SEM				Mean Absolute Error			
	P@1	P@3	P@10	P@20	P@1	P@3	P@10	P@20	P@1	P@3	P@10	P@20
Artery Aorta	0.00	0.00	**0.10**	0.05	0.00	0.00	0.00	0.00	0.00	0.00	**0.10**	**0.10**
Brain Cerebellum	0.00	0.00	0.00	**0.05**	0.00	0.00	0.00	0.00	0.00	0.00	0.00	0.00
Brain Cortex	**1.00**	**0.33**	**0.10**	**0.15**	0.00	0.00	0.00	0.00	0.00	0.00	0.00	0.00
Brain Spinal cord cervical c 1	0.00	**0.33**	**0.10**	**0.05**	0.00	0.00	0.00	0.00	0.00	0.00	0.00	0.00
Heart Atrial Appendage	**1.00**	**1.00**	**0.50**	**0.45**	0.00	0.00	0.00	0.05	**1.00**	0.33	0.20	0.35
Heart Left Ventricle	**1.00**	**0.67**	0.50	0.50	0.00	0.00	0.10	0.05	0.00	**0.67**	0.60	0.50
Liver	0.00	0.00	0.00	**0.05**	0.00	0.00	0.00	**0.05**	**1.00**	**0.33**	**0.10**	**0.05**
Lung	0.00	0.00	0.00	0.00	0.00	0.00	0.10	0.10	0.00	**0.33**	0.10	0.05
Muscle Skeletal	0.00	**0.67**	**0.40**	**0.25**	0.00	0.00	0.00	0.00	0.00	0.00	0.00	0.15
Nerve Tibial	0.00	0.33	**0.30**	**0.20**	0.00	0.00	0.00	0.05	**1.00**	**0.67**	0.20	0.15
Ovary	0.00	**0.33**	**0.10**	**0.05**	0.00	0.00	0.00	0.00	0.00	0.00	0.00	0.00
Pituitary	0.00	0.00	0.00	0.00	**1.00**	**0.33**	**0.10**	0.05	0.00	0.00	**0.10**	**0.10**
Skin Not Sun Exposed Suprapubic	**1.00**	**0.67**	**0.30**	0.15	0.00	0.00	0.10	0.15	**1.00**	**0.67**	**0.30**	0.20
Skin Sun Exposed Lower leg	**1.00**	**0.67**	0.30	0.15	0.00	0.33	0.10	0.10	**1.00**	**0.67**	**0.40**	**0.25**
Testis	0.00	0.00	0.00	**0.05**	0.00	0.00	0.00	0.05	0.00	0.00	0.00	0.00
Whole Blood	0.00	**0.33**	**0.30**	**0.25**	0.00	0.00	0.00	0.00	0.00	0.00	0.10	0.05
Whole Brain	**1.00**	**0.67**	**0.40**	**0.30**	0.00	0.00	0.00	0.05	0.00	0.33	0.10	0.15
Average	**0.35**	**0.35**	**0.20**	**0.16**	0.06	0.04	0.03	0.04	0.29	0.24	0.14	0.12

Tissue Network	Absolute Error Standard Deviation				Median Absolute Error				Sum of Absolute Errors				Standard Absolute Error of the Mean			
	P@1	P@3	P@10	P@20	P@1	P@3	P@10	P@20	P@1	P@3	P@10	P@20	P@1	P@3	P@10	P@20
Artery Aorta	0.00	0.00	**0.10**	0.05	0.00	0.00	0.00	0.05	0.00	0.00	0.00	0.00	0.00	0.00	0.00	0.00
Brain Cerebellum	0.00	0.00	0.00	0.00	0.00	0.00	0.00	0.00	0.00	0.00	0.00	0.00	0.00	0.00	0.00	0.00
Brain Cortex	0.00	0.00	0.00	0.00	0.00	0.00	0.00	0.00	0.00	0.00	0.00	0.10	0.00	0.00	0.00	0.00
Brain Spinal cord cervical c 1	0.00	0.00	0.00	0.00	0.00	0.00	0.00	0.00	0.00	0.00	0.00	0.00	0.00	0.00	0.00	0.00
Heart Atrial Appendage	0.00	0.33	0.40	**0.45**	**1.00**	0.67	0.40	0.35	0.00	0.00	0.10	0.05	0.00	0.00	0.10	0.10
Heart Left Ventricle	0.00	0.33	0.50	0.40	0.00	**0.67**	**0.70**	**0.55**	0.00	0.00	0.10	0.05	0.00	0.00	0.10	0.10
Liver	0.00	0.00	**0.10**	**0.05**	**1.00**	**0.33**	**0.10**	0.05	0.00	0.00	0.00	0.00	0.00	0.00	**0.10**	**0.05**
Lung	0.00	**0.33**	**0.30**	**0.15**	0.00	**0.33**	0.10	0.05	0.00	0.00	0.10	0.10	0.00	0.00	0.10	0.10
Muscle Skeletal	0.00	0.33	0.20	0.15	0.00	0.00	0.00	0.15	0.00	0.00	0.00	0.10	0.00	0.00	0.00	0.00
Nerve Tibial	0.00	0.33	**0.30**	**0.20**	**1.00**	**0.67**	0.20	0.10	0.00	0.00	0.00	0.00	0.00	0.00	0.10	**0.20**
Ovary	0.00	0.00	0.00	0.00	0.00	0.00	0.00	**0.05**	0.00	0.00	0.00	0.00	0.00	0.00	0.00	0.00
Pituitary	0.00	**0.33**	**0.10**	0.05	0.00	0.00	**0.10**	0.05	0.00	0.00	0.00	0.00	**1.00**	**0.33**	**0.10**	0.05
Skin Not Sun Exposed Suprapubic	0.00	0.00	**0.30**	**0.30**	**1.00**	**0.67**	**0.30**	0.20	0.00	0.00	0.00	0.05	0.00	0.33	0.10	0.10
Skin Sun Exposed Lower leg	0.00	0.33	0.20	0.15	**1.00**	**0.67**	**0.40**	**0.25**	0.00	0.00	0.00	0.05	0.00	0.33	0.10	0.10
Testis	0.00	**0.33**	**0.10**	**0.05**	0.00	0.00	0.00	0.00	0.00	0.00	0.00	0.00	0.00	0.00	**0.10**	**0.05**
Whole Blood	0.00	0.00	**0.30**	0.20	0.00	0.00	0.10	0.10	0.00	0.00	0.10	0.05	0.00	0.00	0.00	0.05
Whole Brain	0.00	0.00	0.00	0.10	0.00	0.33	0.10	0.20	**1.00**	0.33	0.20	0.15	0.00	0.00	0.10	0.10
Average	0.00	0.16	0.17	0.14	0.29	0.25	0.15	0.13	0.06	0.02	0.04	0.04	0.06	0.06	0.06	0.06

Table S3. The top-ten genes predicted by the ensemble methods and the diseases that these genes were related to.

Protein	Tissue Network	Tissue-associated Disease Protein	Anomalous Protein	Diseases
ENSG00000061455	Artery Aorta	1	1	Patent Ductus Arteriosus 3
ENSG00000107796	Artery Aorta	1	1	Aortic Aneurysm, Familial Thoracic 6
ENSG00000182492	Artery Aorta	0	0	-
ENSG00000115414	Artery Aorta	0	0	-
ENSG00000159251	Artery Aorta	0	0	-
ENSG00000136999	Artery Aorta	0	0	-
ENSG00000077943	Artery Aorta	0	0	-
ENSG00000135324	Artery Aorta	0	0	-
ENSG00000154553	Artery Aorta	0	0	-
ENSG00000133026	Artery Aorta	0	0	-
ENSG00000131095	Brain Cerebellum	0	1	Alexander Disease
ENSG00000104833	Brain Cerebellum	0	1	Leukodystrophy, Hypomyelinating, 6, Dystonia 4, Torsion, Autosomal Dominant
ENSG00000089199	Brain Cerebellum	0	0	-
ENSG00000154127	Brain Cerebellum	0	0	-
ENSG00000105613	Brain Cerebellum	0	1	Mega-Corpus-Callosum Syndrome With Cerebellar Hypoplasia And Cortical Malformations, Cerebellar Hypoplasia
ENSG00000101210	Brain Cerebellum	0	0	-
ENSG00000171885	Brain Cerebellum	0	0	-
ENSG00000188827	Brain Cerebellum	0	0	-
ENSG00000130540	Brain Cerebellum	0	0	-
ENSG00000132639	Brain Cerebellum	0	0	-
ENSG00000131095	Brain Cortex	0	1	Alexander Disease
ENSG00000156475	Brain Cortex	1	0	Spinocerebellar Ataxia 12, Autosomal Dominant Cerebellar Ataxia
ENSG00000130540	Brain Cortex	0	0	-
ENSG00000171885	Brain Cortex	0	1	Brain Edema
ENSG00000129990	Brain Cortex	0	0	-
ENSG00000104833	Brain Cortex	0	0	-
ENSG00000075340	Brain Cortex	0	0	-
ENSG00000127585	Brain Cortex	0	0	-
ENSG00000064787	Brain Cortex	0	0	-
ENSG00000089169	Brain Cortex	0	0	-
ENSG00000064787	Brain Spinal cord cervical c 1	0	0	-
ENSG00000131095	Brain Spinal cord cervical c 1	0	1	Alexander Disease
ENSG00000123560	Brain Spinal cord cervical c 1	0	1	Pelizaeus-Merzbacher Disease, Spastic Paraplegia 2, X-Linked
ENSG00000197971	Brain Spinal cord cervical c 1	0	1	Secondary Progressive Multiple Sclerosis, Demyelinating Disease

Continued on next page

Table S3 – *Continued from previous page*

Protein	Tissue Network	Tissue-associated Disease Protein	Anomalous Protein	Diseases
ENSG00000171885	Brain Spinal cord cervical c 1	0	1	Neuromyelitis Optica
ENSG00000105695	Brain Spinal cord cervical c 1	0	1	Polyneuropathy
ENSG00000156475	Brain Spinal cord cervical c 1	1	1	Spinocerebellar Ataxia 12, Autosomal Dominant Cerebellar Ataxia
ENSG00000104833	Brain Spinal cord cervical c 1	0	0	-
ENSG00000160307	Brain Spinal cord cervical c 1	0	1	Syringoma, Neurofibroma
ENSG00000112280	Brain Spinal cord cervical c 1	0	0	-
ENSG00000134571	Heart Atrial Appendage	1	1	Cardiomyopathy, Familial Hypertrophic, 4
ENSG00000159251	Heart Atrial Appendage	1	1	Atrial Septal Defect 5, Cardiomyopathy, Familial Hypertrophic, 11
ENSG00000175206	Heart Atrial Appendage	1	1	Atrial Standstill 2, Atrial Fibrillation, Familial, 6
ENSG00000120937	Heart Atrial Appendage	0	0	-
ENSG00000077522	Heart Atrial Appendage	1	1	Cardiomyopathy, Dilated, 1Aa, With Or Without Left Ventricular Noncompaction, Myopathy, Distal, 6, Adult-Onset, Autosomal Dominant
ENSG00000155657	Heart Atrial Appendage	1	1	Myopathy, Myofibrillar, 9, With Early Respiratory Failure, Congenital Myopathy 5 With Cardiomyopathy
ENSG00000118194	Heart Atrial Appendage	1	1	Cardiomyopathy, Dilated, 1D, Cardiomyopathy, Familial Hypertrophic, 2
ENSG00000173991	Heart Atrial Appendage	1	1	Cardiomyopathy, Familial Hypertrophic, 25, Muscular Dystrophy, Limb-Girdle, Autosomal Recessive 7
ENSG00000104879	Heart Atrial Appendage	0	0	-
ENSG00000198523	Heart Atrial Appendage	1	1	Cardiomyopathy, Dilated, 1P, Cardiomyopathy, Familial Hypertrophic, 18
ENSG00000134571	Heart Left Ventricle	1	1	Cardiomyopathy, Familial Hypertrophic, 4, Left Ventricular Noncompaction 10
ENSG00000159251	Heart Left Ventricle	1	1	Atrial Septal Defect 5, Cardiomyopathy, Familial Hypertrophic, 11
ENSG00000092054	Heart Left Ventricle	1	1	Myopathy, Distal, 1, Congenital Myopathy 7A, Myosin Storage, Autosomal Dominant
ENSG00000077522	Heart Left Ventricle	1	1	Cardiomyopathy, Dilated, 1Aa, With Or Without Left Ventricular Noncompaction, Myopathy, Distal, 6, Adult-Onset, Autosomal Dominant

Continued on next page

Table S3 – *Continued from previous page*

Protein	Tissue Network	Tissue-associated Disease Protein	Anomalous Protein	Diseases
ENSG00000155657	Heart Left Ventricle	1	1	Myopathy, Myofibrillar, 9, With Early Respiratory Failure, Congenital Myopathy 5 With Cardiomyopathy
ENSG00000118194	Heart Left Ventricle	1	1	Cardiomyopathy, Dilated, 1D, Cardiomyopathy, Familial Hypertrophic, 2
ENSG00000129991	Heart Left Ventricle	1	1	Cardiomyopathy, Dilated, 2A, Cardiomyopathy, Familial Hypertrophic, 7
ENSG00000143632	Heart Left Ventricle	0	0	-
ENSG00000186439	Heart Left Ventricle	1	1	Cardiac Arrhythmia Syndrome, With Or Without Skeletal Muscle Weakness, Catecholaminergic Polymorphic Ventricular Tachycardia 5
ENSG00000114854	Heart Left Ventricle	1	1	Cardiomyopathy, Familial Hypertrophic, 13, Cardiomyopathy, Dilated, 1Z
ENSG00000171557	Liver	0	1	†Afibrinogenemia, Congenital
ENSG00000171564	Liver	0	1	†Afibrinogenemia, Congenital
ENSG00000163631	Liver	0	1	Analbuminemia, Hyperthyroxinemia, Familial Dysalbuminemic
ENSG00000118137	Liver	0	1	Hypoalphalipoproteinemia, Primary, 2, Hypoalphalipoproteinemia, Primary, 2, Intermediate
ENSG00000124253	Liver	0	1	Phosphoenolpyruvate Carboxykinase Deficiency, Cytosolic, Pepck 1 Deficiency
ENSG00000198650	Liver	1	1	Tyrosinemia, Type Ii, Tyrosinemia
ENSG00000145321	Liver	0	1	Hepatic Encephalopathy
ENSG00000171759	Liver	0	1	Phenylketonuria, Hyperphenylalaninemia
ENSG00000257017	Liver	0	1	Anhaptoglobinemia
ENSG00000197249	Liver	0	1	Alpha-1-Antitrypsin Deficiency
ENSG00000187908	Lung	0	0	-
ENSG00000182010	Lung	0	0	-
ENSG00000171885	Lung	0	0	-
ENSG00000175899	Lung	0	0	-
ENSG00000171345	Lung	0	0	-
ENSG00000133661	Lung	0	1	Extrinsic Allergic Alveolitis, Pulmonary Alveolar Proteinosis
ENSG00000197249	Lung	0	1	Alpha-1-Antitrypsin Deficiency, Hemorrhagic Disease Due To Alpha-1-Antitrypsin Pittsburgh Mutation
ENSG00000168484	Lung	1	1	

Continued on next page

Table S3 – *Continued from previous page*

Protein	Tissue Network	Tissue-associated Disease Protein	Anomalous Protein	Diseases
ENSG00000211896	Lung	0	0	-
ENSG00000165140	Lung	0	0	-
ENSG00000130595	Muscle Skeletal	0	1	Arthrogryposis, Distal, Type 2B2, Arthrogryposis, Distal, Type 1A
ENSG00000183091	Muscle Skeletal	1	1	Nemaline Myopathy 2, Arthrogryposis Multiplex Congenita 6
ENSG00000130957	Muscle Skeletal	0	0	-
ENSG00000105048	Muscle Skeletal	1	1	Nemaline Myopathy 5, Nemaline Myopathy
ENSG00000197893	Muscle Skeletal	0	1	Myopathy, Myofibrillar, 5, Myopathy, Myofibrillar, 4
ENSG00000069869	Muscle Skeletal	0	0	-
ENSG00000143632	Muscle Skeletal	1	1	Myopathy, Scapulohumeroperoneal, Congenital Myopathy 2A, Typical, Autosomal Dominant
ENSG00000155657	Muscle Skeletal	1	1	Myopathy, Myofibrillar, 9, With Early Respiratory Failure, Congenital Myopathy 5 With Cardiomyopathy
ENSG00000086967	Muscle Skeletal	0	1	Lethal Congenital Contracture Syndrome 4, Nemaline Myopathy 9
ENSG00000186439	Muscle Skeletal	0	0	-
ENSG00000105227	Nerve Tibial	1	1	Charcot-Marie-Tooth Disease, Demyelinating, Type 4F, Hypertrophic Neuropathy Of Dejerine-Sottas
ENSG00000102385	Nerve Tibial	0	1	Charcot-Marie-Tooth Disease, Charcot-Marie-Tooth Disease, Demyelinating, Type 4F
ENSG00000064787	Nerve Tibial	0	0	-
ENSG00000158887	Nerve Tibial	1	1	Hypertrophic Neuropathy Of Dejerine-Sottas, Charcot-Marie-Tooth Disease, Demyelinating, Type 1B
ENSG00000181092	Nerve Tibial	0	0	-
ENSG00000109099	Nerve Tibial	1	1	Charcot-Marie-Tooth Disease And Deafness, Charcot-Marie-Tooth Disease, Demyelinating, Type 1A
ENSG00000171345	Nerve Tibial	0	0	-
ENSG00000064300	Nerve Tibial	0	0	-
ENSG00000160307	Nerve Tibial	0	1	Neurofibroma
ENSG00000197971	Nerve Tibial	0	0	-
ENSG00000136931	Ovary	1	1	46,Xx Sex Reversal 4, Premature Ovarian Failure 7

Continued on next page

Table S3 – *Continued from previous page*

Protein	Tissue Network	Tissue-associated Disease Protein	Anomalous Protein	Diseases
ENSG00000180447	Ovary	0	0	-
ENSG00000156475	Ovary	0	0	-
ENSG00000185070	Ovary	0	0	-
ENSG00000107317	Ovary	0	0	-
ENSG00000198300	Ovary	0	0	-
ENSG00000143768	Ovary	0	1	Infertility
ENSG00000125398	Ovary	0	0	-
ENSG00000185559	Ovary	0	0	-
ENSG00000117425	Ovary	0	0	-
ENSG00000089199	Pituitary	0	1	Pheochromocytoma
ENSG00000069011	Pituitary	0	0	-
ENSG00000135346	Pituitary	0	0	-
ENSG00000136931	Pituitary	0	0	-
ENSG00000124253	Pituitary	0	0	-
ENSG00000129990	Pituitary	0	0	-
ENSG00000170421	Pituitary	0	0	-
ENSG00000149295	Pituitary	0	0	-
ENSG00000064835	Pituitary	1	1	Pituitary Hormone Deficiency, Combined Or Isolated, 1, Isolated Growth Hormone Deficiency, Type Ii
ENSG00000105894	Pituitary	0	0	-
ENSG00000167768	Skin Not Sun Exposed Suprapubic	1	1	Palmoplantar Keratoderma, Nonepidermolytic, Ichthyosis Hystrix, Curth-Macklin Type
ENSG00000186081	Skin Not Sun Exposed Suprapubic	1	1	Epidermolysis Bullosa Simplex 2F, With Mottled Pigmentation, Epidermolysis Bullosa Simplex 2E, With Migratory Circinate Erythema
ENSG00000186847	Skin Not Sun Exposed Suprapubic	1	1	Dermatopathia Pigmentosa Reticularis, Naegeli-Franceschetti-Jadassohn Syndrome
ENSG00000096696	Skin Not Sun Exposed Suprapubic	0	1	Skin Fragility-Woolly Hair Syndrome, Epidermolysis Bullosa, Lethal Acantholytic
ENSG00000172867	Skin Not Sun Exposed Suprapubic	0	1	Ichthyosis Bullosa Of Siemens, Epidermolytic Hyperkeratosis
ENSG00000171346	Skin Not Sun Exposed Suprapubic	0	1	Morpheaform Basal Cell Carcinoma, Infiltrative Basal Cell Carcinoma
ENSG00000161634	Skin Not Sun Exposed Suprapubic	0	0	-
ENSG00000163207	Skin Not Sun Exposed Suprapubic	0	1	Cholesteatoma Of Middle Ear, Porokeratosis

Continued on next page

Table S3 – *Continued from previous page*

Protein	Tissue Network	Tissue-associated Disease Protein	Anomalous Protein	Diseases
ENSG00000081277	Skin Not Sun Exposed Suprapubic	0	1	Ectodermal Dysplasia/Skin Fragility Syndrome, Ectodermal Dysplasia
ENSG00000178372	Skin Not Sun Exposed Suprapubic	0	0	-
ENSG00000167768	Skin Sun Exposed Lower leg	1	1	Palmoplantar Keratoderma, Nonepidermolytic, Ichthyosis Hystrix, Curth-Macklin Type
ENSG00000186081	Skin Sun Exposed Lower leg	1	1	Epidermolysis Bullosa Simplex 2F, With Mottled Pigmentation, Epidermolysis Bullosa Simplex 2E, With Migratory Circinate Erythema
ENSG00000096696	Skin Sun Exposed Lower leg	0	1	Skin Fragility-Woolly Hair Syndrome, Epidermolysis Bullosa, Lethal Acantholytic
ENSG00000172867	Skin Sun Exposed Lower leg	0	1	Ichthyosis Bullosa Of Siemens, Epidermolytic Hyperkeratosis
ENSG00000161634	Skin Sun Exposed Lower leg	0	0	-
ENSG00000186847	Skin Sun Exposed Lower leg	1	1	Dermatopathia Pigmentosa Reticularis, Naegeli-Franceschetti-Jadassohn Syndrome
ENSG00000163207	Skin Sun Exposed Lower leg	0	0	-
ENSG00000171346	Skin Sun Exposed Lower leg	0	1	Morpheaform Basal Cell Carcinoma, Infiltrative Basal Cell Carcinoma
ENSG00000081277	Skin Sun Exposed Lower leg	0	1	Ectodermal Dysplasia/Skin Fragility Syndrome, Ectodermal Dysplasia
ENSG00000149418	Skin Sun Exposed Lower leg	1	1	Ichthyosis, Congenital, Autosomal Recessive 11, Ichthyosis
ENSG00000118137	Testis	0	0	-
ENSG00000131747	Testis	0	0	-
ENSG00000198033	Testis	0	0	-
ENSG00000174015	Testis	0	0	-
ENSG00000170777	Testis	0	0	-
ENSG00000168454	Testis	0	0	-
ENSG00000183207	Testis	0	0	-
ENSG00000166851	Testis	0	0	-
ENSG00000133101	Testis	0	1	Testicular Cancer
ENSG00000117399	Testis	0	0	-
ENSG00000148346	Whole Blood	0	0	-
ENSG00000104918	Whole Blood	0	0	-
ENSG00000124731	Whole Blood	0	0	-
ENSG00000132965	Whole Blood	0	0	-
ENSG00000143546	Whole Blood	0	0	-

Continued on next page

Table S3 – *Continued from previous page*

Protein	Tissue Network	Tissue-associated Disease Protein	Anomalous Protein	Diseases
ENSG00000141480	Whole Blood	0	0	-
ENSG00000188536	Whole Blood	1	1	Hemoglobin H Disease, Alpha-Thalassemia
ENSG00000100985	Whole Blood	0	0	-
ENSG00000140368	Whole Blood	1	1	Pyogenic Sterile Arthritis, Pyoderma Gangrenosum, And Acne
ENSG00000066336	Whole Blood	0	0	-
ENSG00000131095	Whole Brain	0	1	Alexander Disease
ENSG00000171885	Whole Brain	0	1	Neuromyelitis Optica
ENSG00000156475	Whole Brain	1	1	Spinocerebellar Ataxia 12, Autosomal Dominant Cerebellar Ataxia
ENSG00000064787	Whole Brain	0	0	-
ENSG00000130540	Whole Brain	0	0	-
ENSG00000104833	Whole Brain	1	1	Leukodystrophy, Hypomyelinating, 6, Dystonia 4, Torsion, Autosomal Dominant
ENSG00000129990	Whole Brain	0	0	-
ENSG00000089169	Whole Brain	0	0	-
ENSG00000167971	Whole Brain	0	0	-
ENSG00000155980	Whole Brain	1	1	Spastic Paraplegia 10, Autosomal Dominant, Myoclonus, Intractable, Neonatal
