# Supplementary material for: Network-based anomaly detection algorithm reveals proteins with major roles in human tissues
Source: Gigascience. 2025 Apr 8;14:giaf034. doi: 10.1093/gigascience/giaf034 (PMC11976396; doi:10.1093/gigascience/giaf034)

GigaScience

Network-based anomaly detection algorithm reveals proteins with major roles in human tissues

--Manuscript Draft--

**Manuscript Number:**	GIGA-D-24-00363
**Full Title:**	Network-based anomaly detection algorithm reveals proteins with major roles in human tissues
**Article Type:**	Research
**Funding Information:**	Council for Higher Education — Dr. Michael Fire, Prof. Esti Yeger-Lotem<br>Israel Science Foundation (401/22) — Prof. Esti Yeger-Lotem

Abstract:

Background
Proteins act through physical interactions with other molecules to maintain organismal health. Protein-protein interaction (PPI) networks proved to be a powerful framework for obtaining insight into protein functions, cellular organization, response to signals, and disease states. In multicellular organisms, protein content varies between tissues, influencing tissue morphology and function. Weighted PPI networks, reflecting the likelihood of interactions in specific tissues, offer insights into tissue-specific processes and disease mechanisms. We hypothesized that detecting anomalous nodes in these networks could reveal proteins with key tissue-specific functions.

Results
Here, we introduce Weighted Graph Anomalous Node Detection (WGAND), a novel machine-learning algorithm to identify anomalous nodes in weighted graphs. WGAND estimates expected edge weights and uses deviations to generate anomaly detection features, which are then used to score network nodes. We applied WGAND to weighted PPI networks of 17 human tissues. High-ranking anomalous nodes were enriched for proteins associated with tissue-specific diseases and tissue-specific biological processes, such as neuron signaling in the brain and spermatogenesis in the testis. WGAND outperformed other methods in terms of Area Under the ROC Curve (AUC) and Precision at K (P@K), highlighting its effectiveness in uncovering biologically meaningful anomalies.

Conclusion
Our findings demonstrate WGAND's potential as a powerful tool for detecting anomalous proteins with significant biological roles. By identifying proteins involved in critical tissue-specific processes and diseases, WGAND offers valuable insights for discovering novel biomarkers and therapeutic targets. Its versatile algorithm is suitable for any weighted graph and is broadly applicable across various fields. The WGAND algorithm is available as an open-source Python library at https://github.com/data4goodlab/wgand.

**Corresponding Author:**	Michael Fire, Ph.D<br>Ben-Gurion University of the Negev<br>Beer-Sheva, Israel ISRAEL
**Corresponding Author Secondary Information:**	
**Corresponding Author's Institution:**	Ben-Gurion University of the Negev
**Corresponding Author's Secondary Institution:**	
**First Author:**	Michael Fire, Ph.D
**First Author Secondary Information:**	
**Order of Authors:**	Michael Fire, Ph.D

Powered by Editorial Manager® and ProduXion Manager® from Aries Systems Corporation

	Dima Kagan
	Juman Jubran
	Esti Yeger-Lotem
**Order of Authors Secondary Information:**	
**Additional Information:**	
**Question**	**Response**
Are you submitting this manuscript to a special series or article collection?	No
**Experimental design and statistics**<br><br>Full details of the experimental design and statistical methods used should be given in the Methods section, as detailed in our Minimum Standards Reporting Checklist. Information essential to interpreting the data presented should be made available in the figure legends.<br><br>Have you included all the information requested in your manuscript?	Yes
**Resources**<br><br>A description of all resources used, including antibodies, cell lines, animals and software tools, with enough information to allow them to be uniquely identified, should be included in the Methods section. Authors are strongly encouraged to cite Research Resource Identifiers (RRIDs) for antibodies, model organisms and tools, where possible.<br><br>Have you included the information requested as detailed in our Minimum Standards Reporting Checklist?	Yes
**Availability of data and materials**<br><br>All datasets and code on which the conclusions of the paper rely must be either included in your submission or deposited in publicly available repositories (where available and ethically	Yes

appropriate), referencing such data using a unique identifier in the references and in the “Availability of Data and Materials” section of your manuscript.<br><br>Have you have met the above requirement as detailed in our Minimum Standards Reporting Checklist?	

Powered by Editorial Manager® and ProduXion Manager® from Aries Systems Corporation

Click here to access/download;Manuscript;protiens_anomaly_detection_kaga

Network-based anomaly detection algorithm reveals proteins with major roles in human tissues

Dima Kagan[1*], Juman Jubran[2*], Esti Yeger-Lotem[2,3], and Michael Fire[1]

Abstract

Background

Proteins act through physical interactions with other molecules to maintain organismal health. Protein-protein interaction (PPI) networks proved to be a powerful framework for obtaining insight into protein functions, cellular organization, response to signals, and disease states. In multicellular organisms, protein content varies between tissues, influencing tissue morphology and function. Weighted PPI networks, reflecting the likelihood of interactions in specific tissues, offer insights into tissue-specific processes and disease mechanisms. We hypothesized that detecting anomalous nodes in these networks could reveal proteins with key tissue-specific functions.

Results

Here, we introduce Weighted Graph Anomalous Node Detection (WGAND), a novel machine-learning algorithm to identify anomalous nodes in weighted graphs. WGAND estimates expected edge weights and uses deviations to generate anomaly detection features, which are then used to score network nodes. We applied WGAND to weighted PPI networks of 17 human tissues. High-ranking anomalous nodes were enriched for proteins associated with tissue-specific diseases and tissue-specific biological processes, such as neuron signaling in the brain and spermatogenesis in the testis. WGAND outperformed other methods in terms of Area Under the ROC Curve (AUC) and Precision at K (P@K), highlighting its effectiveness in uncovering biologically meaningful anomalies.

Conclusion

Our findings demonstrate WGAND's potential as a powerful tool for detecting anomalous proteins with significant biological roles. By identifying proteins involved in critical tissue-specific processes and diseases, WGAND offers valuable insights for discovering novel biomarkers and therapeutic targets. Its versatile algorithm is suitable for any weighted graph and is broadly applicable across various fields. The WGAND algorithm is available as an open-source Python library at `https://github.com/data4goodlab/wgand`.

Keywords

Protein-Protein Interaction (PPI) Networks — Anomaly Detection — Weighted Graphs — Machine Learning

[1] *Department of Software and Information Systems Engineering, Ben-Gurion University of the Negev, Beer Sheva 84105, Israel*
[2] *Department of Clinical Biochemistry and Pharmacology, Ben-Gurion University of the Negev, Beer Sheva 84105, Israel*
[3] *The National Institute for Biotechnology in the Negev, Ben-Gurion University of the Negev, Beer Sheva 84105, Israel*
[*] Dima Kagan and Juman Jubran have contributed equally to this work.
Email: kagandi@post.bgu.ac.il, juman@post.bgu.ac.il, estiyl@bgu.ac.il, mickyfi@bgu.ac.il

Introduction

Proteins are the prime molecules in living cells, driving and mediating all biological processes through interactions with other molecules. In multi-tissue organisms, protein content differs between tissues, such that proteins could either be absent or expressed at different levels [1]. These differences affect tissues' morphology and function and susceptibility to aberrations [2]. Protein-protein interaction (PPI) networks, representing the interactions between proteins within a cell, provide a valuable framework for studying protein functions and cellular information transfer [3]. Weighted PPI networks, where edge weight reflects the likelihood of an interaction, offer a refined view of context-specific interactions [4]. For example, weighted PPI networks representing different human tissues helped understand tissue-specific protein functions, biological processes, and differences in disease susceptibilities [5, 6, 7].

In the study of such complex networks, detecting anomalies could offer a unique perspective. Detecting anomalies is a critical problem in diverse domains, from cybersecurity to social network analysis. Anomalies refer to elements or objects "that appear to deviate markedly from other members of the sample in which it occurs" [8]. The study of anomalies has fascinated scientists for centuries, as they often hold unique insights into the dynamics of complex systems [9]. Anomaly detection is an invaluable tool for various disciplines, from aviation to medicine, to uncover insights not easily gained

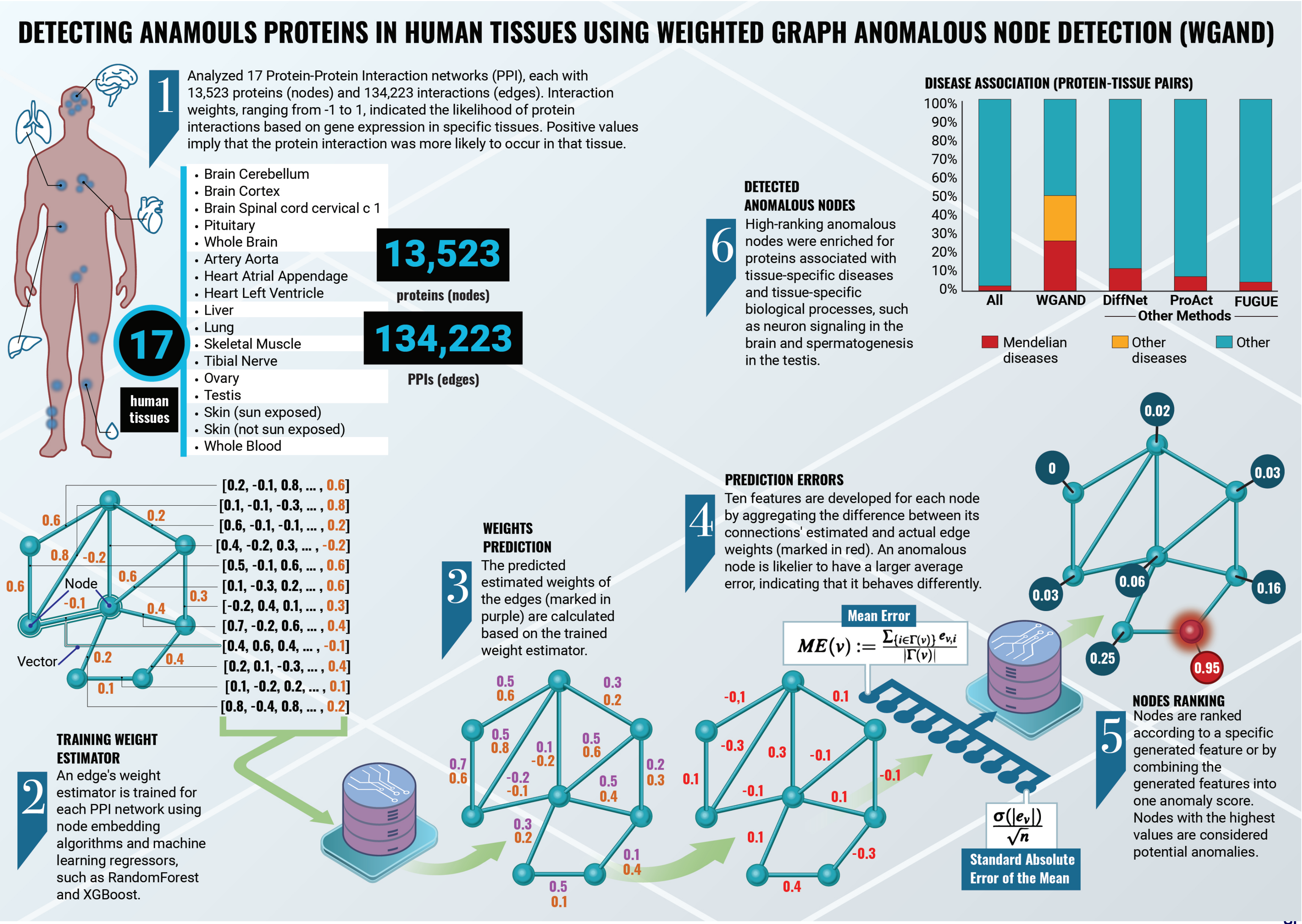

Figure 1. Methodology Overview.

through traditional methods [9]. For instance, in aviation, an anomaly found in airplane sensors may indicate a fault, while in medicine, it could signify a rare disease [10]. In biomedicine, many advances can be attributed to identifying unusual proteins and genes [11].

In recent years, anomaly detection in graphs has gained increasing attention due to its applicability in various domains such as cybersecurity, social networks, and biological networks [10]. The structure and interconnected nature of graphs can provide rich information for anomaly detection. However, the graph's complex nature, including its size, diversity, and noise, makes this task challenging [10]. Over the years, many studies have addressed anomaly detection, especially in graphs. However, only a few studies focused on anomaly detection in weighted graphs [12]. For example, Akoglu et al. [13] introduced OddBall, an algorithm that detects anomalous nodes in weighted graphs. OddbBall focuses on neighborhoods around each node and extracts features from these neighborhoods. Then, these features are transformed into a score to pinpoint outliers. The following year, Davis et al. [14] presented Yagada, an algorithm that searches for structural and numerical anomalies. More recently, Lee et al. [12] introduced GAWD, a method for detecting outliers in weighted graphs. GAWD iterates through the graph, searching for the "best" substructures, which generate the largest compression when replaced with a super-node. The anomaly score is generated based on how well it was compressed.

In this study, we hypothesized that proteins involved with key biological roles within a specific tissue would appear as anomalous nodes in the PPI network, representing that tissue. Among these proteins are those involved in main tissue processes, such as neuron signaling in the brain and spermatogenesis in the testis, and those that participate in tissue-specific diseases, such as BRCA1, whose aberrations increase the risk for breast and ovarian cancers. Previous methods aiming to reveal proteins with key tissue-specific roles have focused on the preferential expression of proteins in specific tissues [15], their network features [6], or both [7, 16]. Here, we combined the latter with the anomaly concept. To identify anomalous proteins, we developed Weighted Graph Anomalous Node Detection (WGAND), a novel generic anomaly detection machine learning-based algorithm for weighted graphs. WGAND is constructed based on the assumption that edge weights of anomalous nodes should deviate from their expected norm. Thus, the model estimated the expected edge weight for all edges in the network and then used the difference between the actual and the expected weight to generate anomaly detection features. These features were then used to train a model that generates anomaly scores for all the network nodes. To test our hypothesis, we applied the WGAND algorithm to weighted PPI networks of 17 different human tissues and to 1,541 proteins involved in tissue-specific diseases. When applied to identify disease-related proteins in most tissues, we found that WGAND obtained higher AUC, Precision-Recall Area Under the Curve (PR-AUC), and P@K, than other methods (Table S1). High-ranking anomalous proteins were more likely to be associated with diseases than other proteins, were enriched for proteins involved in tissue-specific processes, and tended to be involved in preferentially active processes in the designated tissue. Notably, WGAND outperformed three other methods for identifying proteins involved in tissue-specific processes or diseases. Whereas our study used weighted PPI networks as a case study, WGAND is a generic method that could be applied to any weighted graph. WGAND is an open Python library: `https://github.com/data4goodlab/wgand`.

Results

To create an anomaly detection model based on weighted graphs, we used 17 tissue-specific differential PPI networks (Methods). The anomaly detection method assumes that the edge weight of anomalous nodes are likely to deviate from their expected norm. Hence, we constructed an anomaly detection model (Fig. 1):

1. We trained an edge weight estimator to predict edge weights.
2. We constructed meta-features based on the error of the edge weight prediction.
3. We trained a model on the meta-features to predict anomalous nodes.

Using Edge Weight Estimation Errors to Identify Anomalous Nodes

To build an edge weight estimator, we used node embedding methods [17] to generate features representing the nodes, allowing us to predict the edge weight. We tested and evaluated five different node embedding models and assessed their performance in detecting anomalous nodes using *AUC*, *PR-AUC*, *P@K*, and *runtime* (Table 1).

On average, the RandNE [18] embedding method showed the highest performance by all metrics, including the fastest average runtimes in generating the embeddings (Table 1). Although its AUC is slightly higher than other models, its *P@K* and *PR-AUC* show considerable gains compared to the rest of the models.

Next, we used the node embedding features that were generated by RandNE and constructed three different edge weight estimators using different regression algorithms (Methods). First, we evaluated their performances just on the *edge-weight estimation task*. The model created by LightGBM presented the best performance in terms of Mean Squared Error (MSE) (Table 2). Second, we evaluated the performance of the generated anomaly detection models (Table 3). We found that, on average, when using the Random Forest [19] as an edge weight estimator to generate the meta-features, the anomaly detection model achieved the highest score in all anomaly detection metrics. Therefore, we chose the combination of

Table 1. Evaluation of different node embedding algorithms

Embedding	AUC	PR-AUC	P@1	P@3	P@10	P@20	Embedding Runtime (Sec)
DeepWalk	0.6629	0.0528	0.4118	0.2353	0.1941	0.1588	96
GLEE	0.6699	0.0417	0.3529	0.2549	0.1765	0.1412	4
Node2Vec	0.6658	0.0565	0.4118	0.2745	0.2412	0.1824	2912
NodeSketch	0.6700	0.0569	0.4118	0.3137	0.2471	0.1941	229
RandNE	**0.6701**	**0.06155**	**0.5294**	**0.3725**	**0.2529**	**0.2147**	**1.6**

RandNE embedding and Random Forest edge weight estimator, which presented the best results, for the anomaly detection model construction.

We turned to check whether the edge weight estimator could provide biological insights (Methods). Per tissue network, we created a PPI subnetwork that only contained edges with a high difference between the predicted value and the actual value. We labeled the disease-related nodes within each subnetwork. We hypothesized that nodes that appear on paths between disease-related nodes are likely to have central roles in the corresponding tissue. Hence, we counted how many times each node appeared on a path between two disease-related nodes. We focused on the top-10 proteins (nodes) per network that participated in the largest number of paths between two disease-related proteins.

Table 2. Interaction estimator performance based on different models

Weight Estimator	MSE
LightGBM	**0.0016**
RandomForest	0.0029
XGBoost	0.0020

Table 3. Average metrics for different weight estimators.

Weight Estimator	AUC	PR-AUC	P@1	P@3	P@10	P@20
LightGBM	0.64	0.03	0.18	0.14	0.12	0.08
RandomForest	**0.67**	**0.06**	**0.53**	**0.37**	**0.25**	**0.21**
XGBoost	0.60	0.01	0.00	0.00	0.01	0.02

We used the TRACE tool [16] to rank these proteins by their likelihood of being disease-related in the given tissue. The top-10 proteins were ranked significantly higher in the given tissue relative to their median ranking in other tissues ($p-value = 7.57e-6$; Wilcoxon test; Fig. 2). In addition, except for brain cerebellum tissue, the top-10 proteins ranked significantly higher relative to other proteins of the same tissue (adjusted $p-value \leq 8.12e-3$, Mann-Whitney test, Benjamini-Hochberg correction; Fig. 2). These results show that the top-10 proteins were not randomly picked but could potentially have important tissue-specific roles. Overall, this analysis suggests that based on the error in predicting the edge weight, we can identify anomalous nodes.

Ensemble Anomaly detection method outperforms other methods in detecting anomalous nodes

Next, we aimed to construct an unsupervised machine-learning-based anomaly detector model to detect anomalous nodes. Per tissue PPI network, we assigned nodes with ten meta-features constructed based on the edge weight prediction error (Methods). Then, using the ground truth labels, we evaluated the performance of four suggested anomaly detector models and compared them to two baseline models (Methods). On average, all four anomaly detector models present superior performance over the baseline models in all metrics with respect to AUC and P@K (Fig. 3). In terms of AUC, the ensemble and the feature's mean presented the best performance with a marginal difference. In terms of P@K, we saw similar performance between the ensemble, PCA, and the feature's mean with a slight advantage for the ensemble. In addition, we evaluated the performance of the ensemble method against the baseline models of each tissue network separately. The ensemble method had a higher AUC than the baselines across all tissues, higher PR-AUC in 16 out of 17 tissues, and higher P@20 in 13 out of 17 tissues (Table S1). Overall, the ensemble method – 'WGAND (ensemble)' - performed best. For 85% of the datasets, the proposed method achieved higher performance than the baselines in terms of AUC and P@K. Figures 4 and S1 show significant variance between the different methods in most tissues. Furthermore, we observed that some tissues, such as the Brain Spinal cord cervical c 1, where the anomaly detector achieves high AUC but with a relatively lower P@K values (Table S1).

Next, to inspect which feature could explain anomaly best and may be used as an indicator in unlabeled networks, we used the WGAND 'ensemble' method and constructed a model per feature. The models were evaluated based on their P@K values (Fig. S2). Most features had P@K higher than random in all the tissue networks (Table S2). The features with the best performance on average were the Sum of Errors, Error Standard Deviation, Mean Error, and Mean Absolute Error.

WGAND predicts anomalous proteins with major biological roles

We hypothesized that tissue-associated anomalous proteins would play major biological roles in that tissue, which would be evident either by their involvement in a disease that affected that tissue or in tissue-specific or tissue-preferentially active biological processes. We tested this hypothesis for

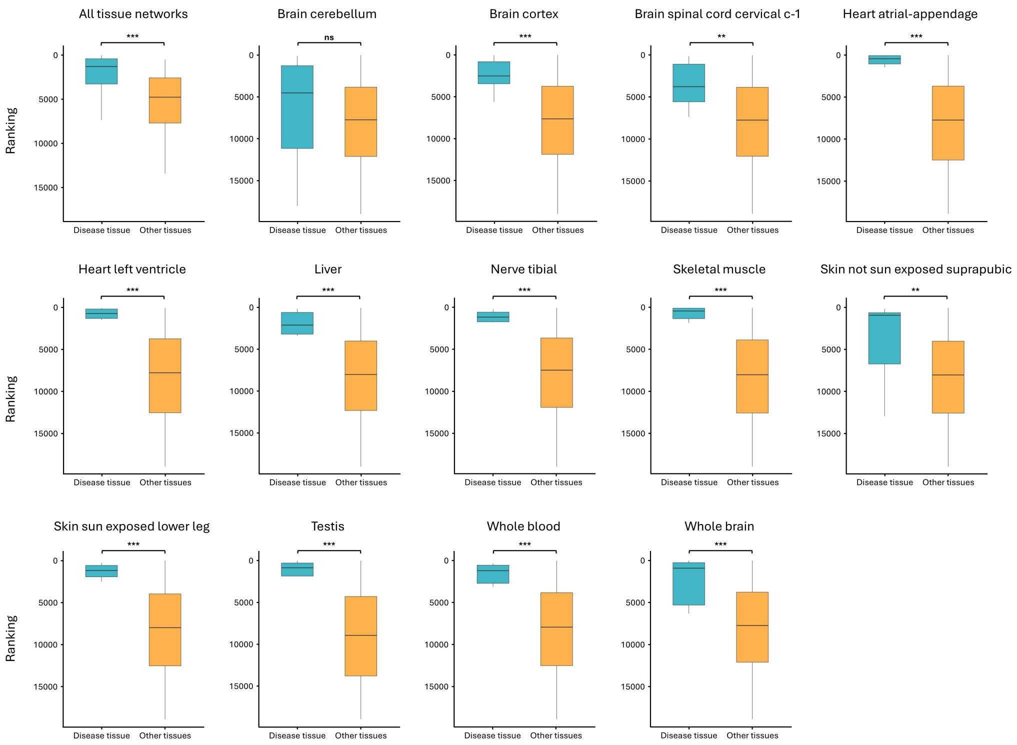

Figure 2. **Pathway analysis validation. The 10 proteins that were most prevalent on paths between disease-related proteins** were significantly more likely to be disease-related in that tissue (blue) than in other tissues (orange; $p-value = 7.57e-6$, Wilcoxon test). Top-10 proteins were collated from 13 tissue networks. Adjusted p-values for cerebellum, cortex, spinal cord, heart atrial appendage, heart left ventricle, liver, nerve tibial, skeletal muscle, skin not sun-exposed, skin sun-exposed, testis, whole blood and whole brain in respective order: $0.13, 8.14e-4, 8.12e-3, 2.75e-6, 2.46e-6, 9.21e-4, 3.85e-5, 3.03e-5, 1.85e-3, 9.83e-5, 3.11e-5, 1.16-4$, and $8.6e-4$ (one-sided Mann-Whitney test, Benjamini-Hochberg correction).

the top-10 anomalous proteins per tissue network according to the best-performing combination "WGAND (ensemble)." First, we focused on the involvement of these proteins in Mendelian diseases. We found that 26% of the top-10 anomalous proteins from all the tissues (a total of 170 proteins) were indeed related to Mendelain diseases affecting the relevant tissue [20] relative to only 1.5% when considering all proteins per tissue (Fig. 5A). An additional 24% of the top-10 proteins were associated with other tissue selective diseases and phenotypes according to literature and disease-related databases (Fig. 5A and Table S3). Hence, top-10 anomalous proteins were highly enriched for proteins involved in diseases and phenotypes affecting that tissue. In addition, we compared "WGAND (ensemble)" to three other methods that were used to detect disease-related proteins (Methods). The first, DiffNet [21], scored proteins by their median interaction weights in the given tissue. It was previously shown that the DiffNet subnetworks composed of the top 1% differential interaction were enriched with disease-related proteins that affect the corresponding tissue. The second, ProAct [22], scored proteins by the median activity score of their associated biological processes. It was previously shown that 21% of the disease-related proteins participate in highly active processes that affect the corresponding tissue. The third, FUGUE, predicted tissue-relevant genes by applying machine learning to transcriptional and network features of genes [7]. FUGUE distinguished tissue or cell type-specific genes better than conventional methods that used tissue-specific expression alone [7]. FUGUE was applicable to 10 tissues that WGAND also analyzed. Among the top-10 proteins per tissue ranked by DiffNet, ProAct, and FUGUE, only 11%, 6%, and 4%, respectively, were annotated as Mendelian disease-related in the relevant disease-tissue (Fig. 5A). WGAND, with a percentage of 24% among its top-10 proteins, outperformed the other methods.

Next, we checked whether the top-10 anomalous proteins were involved in tissue-specific or preferentially active biological processes in the corresponding tissue. We first calculated

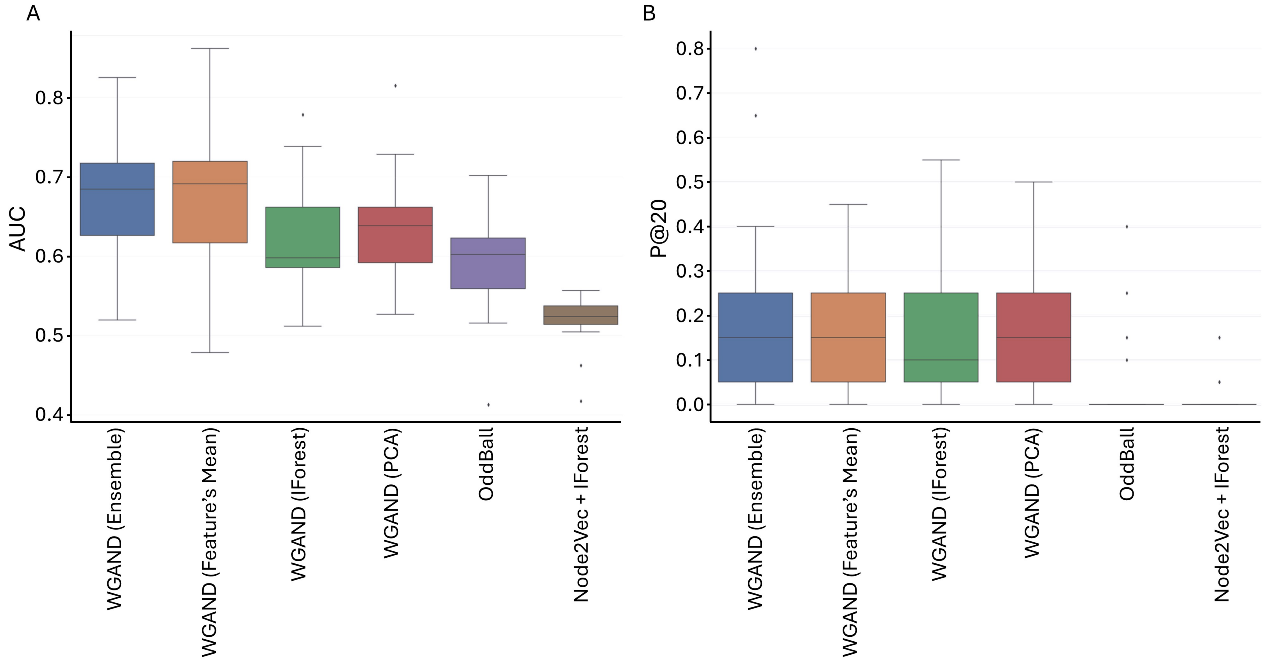

Figure 3. Evaluation of different classifiers using AUC (A) and P@20 (B) measures to predict anomalous nodes based on the meta-features.

the overlap between the top-10 anomalous proteins and proteins involved in tissue-specific biological processes. The overlap was significant (Fig 5B; $p-value = 6.5e-49$, Fisher Exact test). We repeated the same test for FUGUE (Fig. 5B). The top-10 tissue-relevant proteins predicted by FUGUE per tissue were also enriched for tissue-specific biological processes, validating the power of machine learning. Yet, the overlap according to FUGUE was less statistically significant relative to WGAND (Fig 5B; $pvalue = 2.45e-5$, Fisher Exact test).

Lastly, the top-10 anomalous proteins were also more likely involved in processes that were active preferentially in the corresponding tissue (Fig 5C; $p-value = 2.5e-16$, Mann-Whitney test). We applied similar tests to the top-10 anomalous proteins that were detected by the other three WGAND (feature's mean, IForest, and PCA). They also showed significant yet slightly weaker enrichments (Fig. 5D).

Discussion

We presented a novel machine learning-based method for detecting abnormal nodes in weighted networks with positive and negative weights, which has important implications for various domains. Here, we demonstrated the effectiveness of our approach by applying it to PPI networks of different human tissues to identify anomalous proteins. By anomalous proteins we refer not only to proteins associated with human diseases that manifest in specific tissues, but also to proteins with major biological roles in specific tissues. Knowledge of such proteins could illuminate the molecular basis of human diseases, tissue phenotypes, and tissue-specific physiological processes. These goals were partially met by previous methods that relied on tissue transcriptomes [22], tissue-relevant networks [21], or methods that combined both, often with machine learning [7, 16]. Yet, the concept of network anomaly has not been used for this purpose.

We first demonstrated the relevance of network anomaly by showing that estimation errors of edge weights could be used to identify disease-related genes (Fig. 2). Next, we took this approach further by devising a machine-learning method, WGAND, which was trained on features derived from estimation errors. Lastly, we used the predicted anomaly scores to rank anomalous proteins. We showed that the top-10 anomalous proteins overlapped significantly with proteins involved in tissue-specific or tissue- preferentially-active biological processes. Moreover, WGAND outperformed other methods in detecting disease-related and tissue-relevant proteins (Fig. 5). We evaluated WGAND on weighted networks where edge weights can be positive and negative. WGAND can also be applied to other types of weighted networks, such as those with only positive or negative values. Moreover, WGAND can also be used for unweighted networks by converting the networks' weights into binary values, where the existing edge will have a weight of 1 and the non-existing edge will have a weight of 0. WGAND should be able to generate anomaly scores for all of these cases. We hope to evaluate this in future studies by analyzing additional types of networks.

We evaluate WGAND's performance using AUC, P@K, and PR-AUC. When dealing with imbalanced data, P@K is

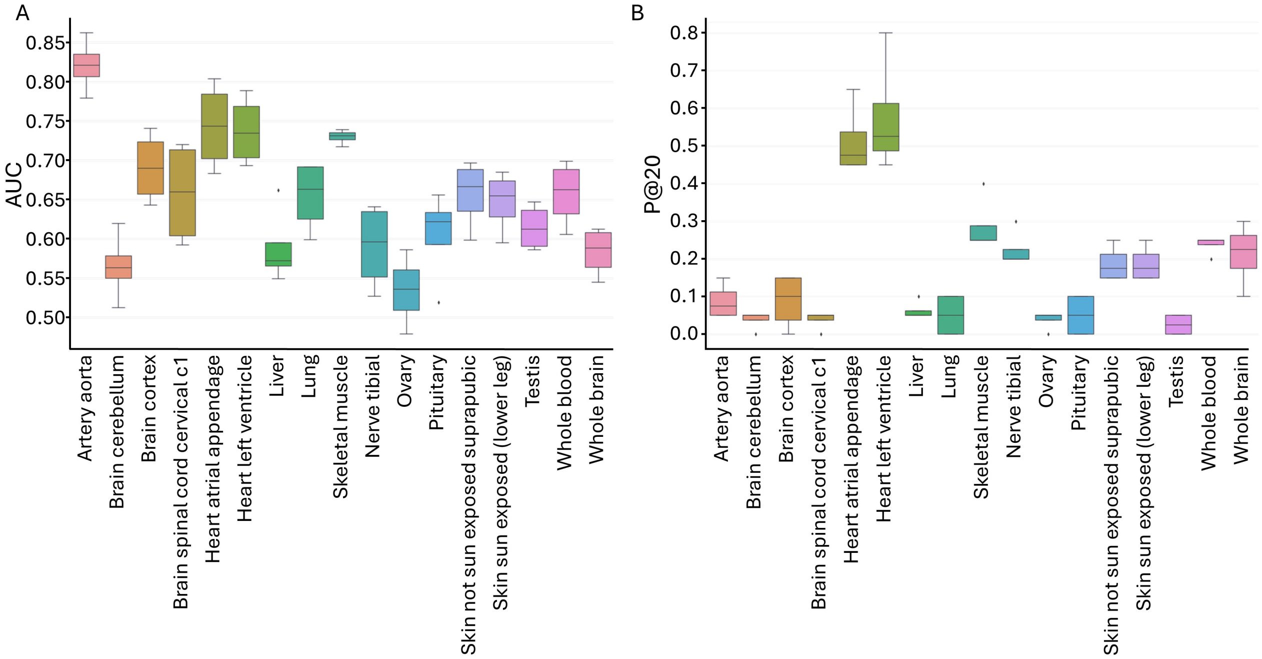

Figure 4. Evaluation of the variance of the four methods for predicting anomalous nodes for each tissue. We can observe that most tissues exhibit high variance in performance between different methods.

critical, as a model with higher P@K can help experts narrow down the search space when inspecting anomalies. While the differences between various node embedding models are not highly significant, it is possible that the performance differences could be more pronounced in detecting anomalous nodes in different networks, such as social networks or computer networks. Additionally, the performance of the method may be improved by using a more systematic way to select the best node embedding algorithm for a given task. Moreover, using hyperparameter optimization tuning, using frameworks like Optuna [23] can also improve the algorithm's performance. We plan to investigate this further in future studies by analyzing additional node embedding models and networks.

We found that the WGAND ensemble performs significantly better than the baseline models in most tissues. OddBall outperformed WGAND in only four brain-related networks in terms of P@K and one network in terms of PR-AUC, but worse in terms of AUC (Table S1). These results suggest that biological differences between brain tissues and other tissues may be a contributing factor to the observed performance differences. In future studies, one could explore the commonalities and differences between brain-related tissues further. We also noted that Node2Vec with IForest could not achieve good results with this data in all the tissues, suggesting it was not suited for the task.

We showed that a single meta-feature can be a good measure for node anomaly detection. On average, the Sum of Errors is the best predictive single feature across all metrics. Surprisingly, the Absolute Sum of Errors is the worst feature across all metrics, despite our expectation that the error direction should not matter and that a higher total error should be a good indication of an anomaly. This result suggests that error direction is an important factor in node anomaly detection and that the difference between regular and absolute meta-features is inconsistent. In a future study, we would like to evaluate the WGAND on different use cases, such as malicious user detection in online social networks. We would like to explore if the same type of anomaly detection features would continue to give the best performance in different domains.

Lastly, we investigated different methods for generating anomaly scores and extracting anomalies from the generated anomaly features and found that some methods have similar performance. Among the four proposed methods, the IForest-based solution performs the worst in terms of AUC, while Feature's Mean has the best AUC. In terms of P@K, the differences are smaller, but different methods have advantages in different tissue networks. For example, the ensemble-based method has a significant advantage in the liver network, while the PCA-based and Feature's Mean methods have a significant advantage in the lung network. Also, some tissues, such as Skin Not Sun Exposed Suprapubic, Heart Left Ventricle, and Nerve Tibial had higher P@K when learning from a single feature than all of the features. While it is difficult to conclude which method is the best, a good rule of thumb would be first to try using the ensemble-based WGAND, which should combine the advantages of all other methods.

Currently, our search for anomalous nodes is limited to proteins with known PPIs. Future applications could extend

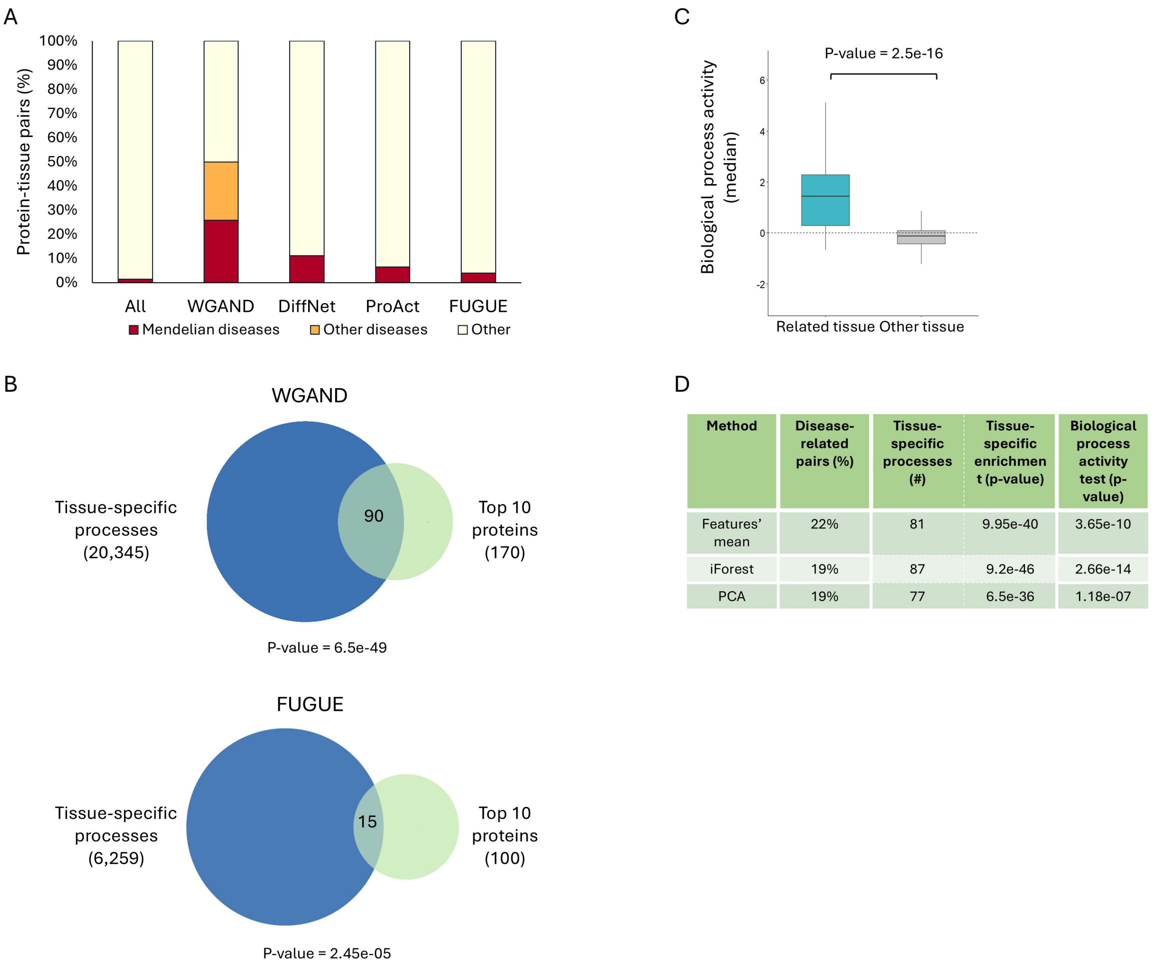

Method	Disease-related pairs (%)	Tissue-specific processes (#)	Tissue-specific enrichment (p-value)	Biological process activity test (p-value)
Features' mean	22%	81	9.95e-40	3.65e-10
iForest	19%	87	9.2e-46	2.66e-14
PCA	19%	77	6.5e-36	1.18e-07

Figure 5. Top-10 anomalous proteins were enriched for tissue-specific diseases and cellular processes. (a) The percentage of protein-tissue pairs where the protein was associated with a Mendelian disease affecting that tissue (red). The percentage of such pairs out of all possible pairs was 1.5% (All), which was lower than their percentage among the top-10 pairs ranked by WGAND (26%), DiffNet (11%), ProAct (6%), and FUGUE (4%). The top-10 pairs ranked by WGAND were also enriched for proteins involved in other tissue-selective diseases and phenotypes (24%, orange). (b) The overlap between the top-10 protein-tissue pairs and pairs where the protein was associated with a biological process specific to that tissue. The number of such pairs appears in parentheses. Pairs ranked by WGAND were more significantly enriched than pairs ranked by FUGUE (Fisher exact test). (c) Top-10 proteins per tissue were more likely associated with cellular processes that were preferentially active in that tissue (blue) relative to other tissues (grey; p-value = $2.5e-16$, Mann-Whitney). (d) Analyses in panels (a-c) were applied to the top-10 anomalous proteins detected by three methods, including features' mean, IForest, and PCA. The results were significant yet weaker than the ensemble method.

the search for other types of genes, for example, by using coexpression networks [24] and to additional contexts, such as cell types [25].

Methods

Dataset of weighted tissue-specific PPI networks

Data of 17 PPIs networks were downloaded from [26] (Table 4). Each network was composed of 13,523 proteins (nodes) and 134,223 PPIs (edges). The weight of an interaction relied on the expression of the interacting genes in the given tissue relative to their expression in other tissues [26]. Weights ranged between $[-1,1]$, where positive scores imply that the protein interaction was more likely to occur in that tissue relative to other tissues, and negative scores imply that it is less likely to occur.

Table 4. PPI Networks Datasets

Tissue	#Disease-related nodes (%)
Artery Aorta	23 (0.17)
Brain Cerebellum	65 (0.48)
Brain Cortex	77 (0.57)
Brain Spinal cord cervical c 1	48 (0.35)
Heart Atrial Appendage	133 (0.98)
Heart Left Ventricle	138 (1.02)
Liver	53 (0.39)
Lung	274 (2.03)
Muscle Skeletal	121 (0.89)
Nerve Tibial	77 (0.57)
Ovary	41 (0.3)
Pituitary	23 (0.17)
Skin Not Sun Exposed Suprapubic	125 (0.92)
Skin Sun Exposed Lower leg	125 (0.92)
Testis	101 (0.75)
Whole Blood	442 (3.3)
Whole Brain	564 (4.2)

Annotation of disease-related proteins and tissue-specific biological processes

Data on Mendelian diseases, disease-related proteins, and disease-affected tissues were obtained from ODiseA [20]. Nodes of a given tissue PPI network were labeled disease-related if the corresponding protein was associated with a Mendelian disease that affected that tissue. As shown in Table 4, up to 4.2% of the nodes per network were labeled disease-related. Data of proteins involved in additional tissue-selective diseases and phenotypes were obtained from PUBMED [27], GeneCards [28], and HPO [29] databases. Data of proteins participating in tissue-specific biological processes were obtained from Basha et al. [26]. Data on estimated activities of biological processes per protein and tissue were obtained from ProAct [30]. Data of tissue risk assessment per protein, reflecting the likelihood of being disease-related in a given tissue, was available for 11 tissues from the TRACE webtool [31]. The 11 tissues were mapped to 13 tissue PPI networks (e.g., we mapped skin tissue both to sun-exposed and unexposed skin tissue PPI networks).

Anomaly detection framework

Precisely defining what constitutes an anomaly is challenging [32], as definitions can vary across different domains and disciplines. Generally, anomalies are considered unusual observations that deviate significantly from the general population to the extent that they suggest a different mechanism may have created them [13]. In this study, we treated an anomaly as a node that behaves differently from most of the nodes in the network.

To formalize this idea, let us consider a weighted graph $G=(V,E,W)$, where V is the set of vertices, E is the set of edges, and W is the set of weights. Given a vertex v, we define its neighborhood $\Gamma(v)=\{(v,u) \mid (v,u)\in E\}$ as the set of vertices that are connected to v by an edge, and their weights as $W_v=\{W_{v,u} \mid u\in\Gamma(v)\}$, where W_a and W_n represent the edge weights between a suspected anomalous node a with its neighbors and a normal node n with its neighbors, respectively.

Extending our recent work [33], we hypothesized that W_a should deviate from W_n if a is truly anomalous. In other words, the average edge weights for a should differ from those of n. To test this hypothesis, we assumed that a general predictive function $P_f(v,u)$ exists, which can estimate the weight of an edge (v,u) with high accuracy. Given this assumption, we expect that $|\hat{W}_{a,v}-W_{a,v}| > |\hat{W}_{n,v}-W_{n,v}|$ if v is a neighbor of a and a is genuinely anomalous. This is because the predicted weight $\hat{W}_{a,v}$ for the edge (a,v) should deviate considerably from the actual weight $W_{a,v}$ if a is anomalous. In contrast, the predicted weight $\hat{W}_{n,v}$ for the edge (n,v) should be close to the actual weight $W_{n,v}$ if n is normal.

Since most nodes and their edges are normal, it should be possible to train a model that learns to predict the values of normal interactions. A model that will learn to generalize well should successfully predict the normal values and fail to predict abnormal values, resulting in higher errors for these cases.

Development of an Edge Weight Estimator

We trained a model that estimates edge weights to create the predictive function $P_f(v,u)$. We used node embedding to generate features representing nodes v and u to train $P_f(v,u)$. Node embedding is a vector representation of nodes in a graph designed to capture the relationships between nodes [34]. Unlike structure-based feature sets that may be specific to certain types of networks, node embedding is a more general solution that can produce near-state-of-the-art performance without requiring additional handcrafted features.

We treated the network as an unweighted graph to generate the embedding vectors.[1] To select the node embedding method, we evaluated five types of embeddings: node2vec [35], RandNE [18], GLEE [36], NodeSketch [37], and DeepWalk [38] [39] based on the implementation by Rozemberczki et al. [39].

Using the extracted embeddings, we then trained a weight estimator using three regressors: Random Forest [19], XG-

[1] Some node embedding algorithms, such as node2vec [35], use the edge weights when building the embedding. Using embedding created from a weighted graph to train an edge weight estimator creates a data leakage that will affect the whole system's performance.

Boost [40], and LightGBM [41]. We measured the performance of the weight estimators using MSE. Also, it is important to note that during the training of the weight estimator, it is crucial to avoid considerable overfit.[2] Suppose the model overfits and "memorizes" the edge values like an infinitely large decision tree. In that case, it will be unable to identify nodes that deviate from the norm, as there will be no deviations in the predictions.

Construction of anomaly detection features

Anomaly detection features are used to identify nodes in the network that behave differently from the norm. Features are constructed to represent a node by aggregating the difference between the estimated and the actual edge weight of its edges. An anomalous node is likelier to have a larger average error, indicating that it behaves differently. The accumulation suggests that the deviation is not a result of the model error but something systematic.

Deviation from the norm can be measured in a variety of ways. Similarly to Kagan et al. [33], we have generated anomaly detection features based on basic statistics such as standard deviation, mean, etc. To create the anomaly detection features, we defined the error of an edge (u,v) as $e_{u,v} = \hat{W}_{u,v} - W_{u,v}$ and the error of a node v as $e_v = \{e_{u,v} \mid u \in \Gamma(v)\}$. Based on these scores, we calculated ten meta-features for each node in the network:

1. **Mean Error** - The average error of the predicted weights of the interactions between node v and its neighbors: $ME(v) := \frac{\sum_{\{i \in \Gamma(v)\}} e_{v,i}}{|\Gamma(v)|}$.
2. **Error Standard Deviation** (Error SD) - The dispersion of the predicted weights of the interactions between node v and its neighbors: $\sigma(e_v)$.
3. **Median Error** - The median error of the predicted weights of the interactions between node v and its neighbors: $median(e_v)$.
4. **Sum of Errors** - The total sum of errors of the predicted weights of the interactions between node v and its neighbors: $\sum_{\{i \in \Gamma(v)\}} e_i$.
5. **Standard Error of the Mean (SEM)** - Measures the average amount of error variation of the predicted weights of the interactions between node v and its neighbors: $\frac{\sigma(e_v)}{\sqrt{n}}$.
6. **Mean Absolute Error** - The average absolute error of the predicted weights of the interactions between node v and its neighbors: $MAE(v) := \frac{\sum_{\{i \in \Gamma(v)\}} |e_{v,i}|}{|\Gamma(v)|}$.
7. **Absolute Error Standard Deviation** - The dispersion of the absolute errors of the predicted weights of the interactions between node v and its neighbors $\sigma(|e_v|)$.
8. **Median Absolute Error** - The absolute median error of the predicted weights of the interactions between node v and its neighbors: $median(|e_v|)$.
9. **Sum of Absolute Errors** - The sum of absolute errors of the predicted weights of the interactions between node v and its neighbors: $\sum_{i \in \Gamma(v)} |e_i|$.
10. **Standard Absolute Error of the Mean** - Measures the average amount of absolute error variation of the predicted weights of the interactions between node v and its neighbors: $\frac{\sigma(|e_v|)}{\sqrt{n}}$.

[2] We tuned the models' parameters by limiting each tree's max depth to reduce overfitting.

To inspect the potential of the proposed features in predicting anomalies, we extracted the meta-features of the differential PPI networks and treated them as a ten-dimension vector. Then, we used Principal Component Analysis (PCA) to reduce the meta-features into a two-dimensional vector. The PCA analysis revealed that the disease-related proteins are grouped together. This result indicates that the meta-features successfully identified proteins with major biological roles in a specific tissue and these proteins behave as anomalies (Fig. S3).

Anomalous nodes detection

The main approach to search for anomalies is by ranking the nodes according to a specific anomaly detection feature, where nodes with the highest values are considered potential anomalies. However, determining the most suitable feature among the presented anomaly detection features can be challenging. To address this, we propose four methods combining the generated anomaly detection features into one anomaly score.

1. **Feature's Mean** - uses a sigmoid function to normalize the values of each anomaly detection feature, then assigns each node with the mean normalized values of the features. This process produces a single value that can be used as the anomaly score.
2. **PCA** - reduces the dimensionality of the anomaly detection features. The resulting component value can be used as the anomaly score [42].
3. **Isolation Forest (IForest)** - uses anomaly detection features as the IForest [43] model input to generate an anomaly score.
4. **Ensemble** - combines the previous three methods. Here, the raw Feature's Mean, PCA values, and also their values after a sigmoid function were inserted into an IForest model to generate the anomaly score. Then, we select the max value from this new anomaly score and the scores produced from the first two methods.

Evaluation scheme

To assess the effectiveness of our method and evaluate anomaly detection performance, we performed a metrics-based evaluation. We measured the models' *AUC* [44], PR-AUC [45], and *P@K*, where *k* refers to the number of top-ranked records (nodes) to consider. *AUC* and PR-AUC are commonly used metrics to estimate the overall performance of a classifier [44, 45]. *P@K* is particularly useful in anomaly detection tasks, where the goal is to discover new items and recommend and prioritize a subset of predictions with the highest confidence scores. Based on these metrics, firstly, we compared the performance of five different node embedding models on weighted networks. Secondly, using the best-performing embedding model, we compared the performance of three edge weight estimator models, where their performance was also measured by *MSE*. Thirdly, we used the best-performing combination of node embedding and edge weight estimator models and evaluated the performance of four different anomaly detection methods.

To evaluate the performance of the proposed method, we used two baselines:

1. **Node2Vec + IForest** - Similarly to Lee et al. [12], we used node2vec combined with the IForest model. Here, we generated the embeddings based on the weights of the graph edges via node2vec. Then, we utilized these embeddings as the features for the IForest model.

2. **OddBall** - We used the OddBall algorithm [13] for anomaly detection in weighted graphs. To the best of our knowledge, OddBall is the only available algorithm with an implementation[3] that was specifically designed for anomaly detection in weighted graphs.

Evaluation of edge weight prediction by their ability to identify disease-related nodes

Per tissue, we constructed a tissue-specific PPI subnetwork that contained only edges with a high differences between the actual and the predicted values by the RandNE estimator. Specifically, we only kept edges where the difference between the actual and predicted values was in the 95 quantiles. We counted how many times each node appeared on a path between two disease-related nodes and considered the top-10 most frequent nodes per tissue PPI network. Next, we ranked nodes by their tissue risk assessment score, as computed by the TRACE webtool [16]. We compared the rank of the top-10 nodes of a tissue to their median rank in other tissues using a paired Wilcoxon test. In addition, per tissue, we compared the ranks of the top-10 nodes to those of other nodes that were not included in the top-10 or were not disease-related using the Mann-Whitney test. Using the Benjamini-Hochberg procedure, p-values were adjusted for multiple hypothesis testing [46].

[3] https://github.com/glooooryyt/oddball_py3

Protein anomaly detection model

We tested whether the anomaly detection algorithm could be used to identify anomalous nodes with tissue-specific roles. We considered a protein to have a tissue-specific role if it was (i) designated as disease-related in that tissue or was involved in additional tissue-selective diseases and phenotypes, (ii) participated in a biological process that was specific to that tissue, or (iii) participated in a biological process that was more active in that tissue relative to other tissues. We applied WGAND to each tissue-specific PPI network and focused on the top-10 anomalous nodes per network. To test for (i), we compared the fraction of disease-related proteins among the top-10 anomalous nodes relative to that fraction among all other nodes. To test for (ii), we computed the fraction of top-10 proteins that participated in a tissue-specific biological process out of the total number of proteins participating in such processes in the given tissue and assessed for enrichment using the Fisher exact test. To test for (iii), we compared the estimated biological process activities of the top-10 anomalous proteins in the given tissue to their estimated activities in all other tissues using the Mann-Whitney test.

Comparison of the WGAND anomaly detection method to other methods

We compared the performance of WGAND in revealing disease-related proteins that affect a specific tissue to three other tissue-specific ranking schemes. Firstly, we compared WGAND with a ranking of proteins according to the differential PPI networks that were initially used to build the anomaly detection method [21]. Basha et al. [21] revealed that subnetworks with the highest 1% differential interaction are enriched with disease-related proteins that affect the corresponding tissue. Hence, for each protein within a tissue network, we computed the median differential score of its interactions. Next, per network, we considered the top-ten proteins with the highest median differential score. Secondly, we compared WGAND with a ranking of proteins according to the activities of biological processes per tissue, as estimated by the ProAct method developed by Sharon et al. [30]. Sharon et al. [30] revealed that 21% of disease-related proteins participate in a process that have high activity score in the disease-related tissue. Hence, for each protein per tissue, we computed the median activity score of the processes it participates in. Then, per tissue, we considered the top-ten proteins with the highest median activity score. Thirdly, we compared WGAND with the top-10 tissue-relevant protiens accroding to FUGUE [7]. We downloaded FUGUE scores from [7]. Scores were limited to proteins and tissues, which WGAND also scored. Next, for each of the 10 overlapping tissues, we considered the top-10 tissue-relevant protiens. Per the method, we computed the fraction of proteins among the top-10 proteins of each tissue associated with Mendelian diseases that manifest in the corresponding tissue, as described in the preceding subsection. For FUGUE, we also tested whether the top-10 tissue-relevant proteins per tissue participated in a biological process specific

to that tissue, as described in the preceding subsection.

Availability of Source Code and Requirements

**Project name**	WGAND
**Project homepage**	`https://github.com/data4goodlab/wgand`
**Operating system(s)**	Platform independent
**Programming language**	Python 3.6 or higher
**Other requirements**	pandas, combo, tqdm, pyod, scikit-learn, numpy, networkx, karateclub
**License**	GPL 3.0

Abbreviations

WGAND: Weighted Graph Anomalous Node Detection;
PPI: protein-protein interaction;
AUC: area under curve;
P@K: precious at k;
MSE: mean squared error;
SD: standard deviation;
SEM: Standard error of the mean;
MAE: mean absolute error;
PCA: Principal Component Analysis;
IForest: Isolation Foresrt;

Acknowledgements

J.J. wishes to thank the Baroness Ariane de Rothchild Women Doctoral Program. Additionally, during the drafting of this article, we utilized ChatGPT and Grammarly for editing purposes, ensuring the text's grammar, spelling, and clarity were thoroughly checked and improved as necessary.

Funding

This study was funded by the Israeli Council for Higher Education (CHE) via the Data Science Research Center, Ben-Gurion University of the Negev, Israel [to M.F. and E.Y.-L], and by the Israel Science Foundation [401/22 to E.Y.-L.].

Authors' contributions

D.K., J.J., E.Y.-L., and M.F. conceived the study; D.K. developed the code framework; J. J. and D. K. analyzed the data; E.Y.-L. and M.F. supervised the study; and all authors wrote the paper.

Competing Interests

The authors declare that they have no competing interests.

References

[1] Lihua Jiang, Meng Wang, Shin Lin, Ruiqi Jian, Xiao Li, Joanne Chan, Guanlan Dong, Huaying Fang, Aaron E Robinson, François Aguet, et al. A quantitative proteome map of the human body. *Cell*, 183(1):269–283, 2020.

[2] GTEx Consortium. The gtex consortium atlas of genetic regulatory effects across human tissues. *Science*, 369(6509):1318–1330, 2020.

[3] Marc Vidal, Michael E Cusick, and Albert-László Barabási. Interactome networks and human disease. *Cell*, 144(6):986–998, 2011.

[4] Katja Luck, Dae-Kyum Kim, Luke Lambourne, Kerstin Spirohn, Bridget E Begg, Wenting Bian, Ruth Brignall, Tiziana Cafarelli, Francisco J Campos-Laborie, Benoit Charloteaux, et al. A reference map of the human binary protein interactome. *Nature*, 580(7803):402–408, 2020.

[5] Esti Yeger-Lotem and Roded Sharan. Human protein interaction networks across tissues and diseases. *Frontiers in genetics*, 6:257, 2015.

[6] Casey S Greene, Arjun Krishnan, Aaron K Wong, Emanuela Ricciotti, Rene A Zelaya, Daniel S Himmelstein, Ran Zhang, Boris M Hartmann, Elena Zaslavsky, Stuart C Sealfon, et al. Understanding multicellular function and disease with human tissue-specific networks. *Nature genetics*, 47(6):569–576, 2015.

[7] Gowthami Somepalli, Sarthak Sahoo, Arashdeep Singh, and Sridhar Hannenhalli. Prioritizing and characterizing functionally relevant genes across human tissues. *PLoS Computational Biology*, 17(7):e1009194, 2021.

[8] Frank E Grubbs. Procedures for detecting outlying observations in samples. *Technometrics*, 11(1):1–21, 1969.

[9] Ali Bou Nassif, Manar Abu Talib, Qassim Nasir, and Fatima Mohamad Dakalbab. Machine learning for anomaly detection: A systematic review. *Ieee Access*, 9:78658–78700, 2021.

[10] Leman Akoglu, Hanghang Tong, and Danai Koutra. Graph based anomaly detection and description: a survey. *Data mining and knowledge discovery*, 29:626–688, 2015.

[11] Tomer Michael-Pitschaze, Niv Cohen, Dan Ofer, Yedid Hoshen, and Michal Linial. Detecting anomalous proteins using deep representations. *NAR Genomics and Bioinformatics*, 6(1):lqae021, 2024.

[12] Meng-Chieh Lee, Hung T Nguyen, Dimitris Berberidis, Vincent S Tseng, and Leman Akoglu. Gawd: graph anomaly detection in weighted directed graph databases. In *Proceedings of the 2021 IEEE/ACM International Conference on Advances in Social Networks Analysis and Mining*, pages 143–150, 2021.

[13] Leman Akoglu, Mary McGlohon, and Christos Faloutsos. Oddball: Spotting anomalies in weighted graphs. In *Advances in Knowledge Discovery and Data Mining*, pages 410–421. Springer, 2010.

[14] Michael Davis, Weiru Liu, Paul Miller, and George Redpath. Detecting anomalies in graphs with numeric labels. In *Proceedings of the 20th ACM international conference on Information and knowledge management*, pages 1197–1202, 2011.

[15] Kasper Lage, Niclas Tue Hansen, E Olof Karlberg, Aron C Eklund, Francisco S Roque, Patricia K Donahoe, Zoltan Szallasi, Thomas Skøt Jensen, and Søren Brunak. A large-scale analysis of tissue-specific pathology and gene expression of human disease genes and complexes. *Proceedings of the National Academy of Sciences*, 105(52):20870–20875, 2008.

[16] Eyal Simonovsky, Moran Sharon, Maya Ziv, Omry Mauer, Idan Hekselman, Juman Jubran, Ekaterina Vinogradov, Chanan M Argov, Omer Basha, Lior Kerber, et al. Predicting molecular mechanisms of hereditary diseases by using their tissue-selective manifestation. *Molecular Systems Biology*, 19.8: e11407, 2023.

[17] Nino Arsov and Georgina Mirceva. Network embedding: An overview. *arXiv preprint arXiv:1911.11726*, 2019.

[18] Ziwei Zhang, Peng Cui, Haoyang Li, Xiao Wang, and Wenwu Zhu. Billion-scale network embedding with iterative random projection. In *2018 IEEE International Conference on Data Mining (ICDM)*, pages 787–796. IEEE, 2018.

[19] Leo Breiman. Random forests. *Machine learning*, 45:5–32, 2001.

[20] Idan Hekselman, Lior Kerber, Maya Ziv, Gil Gruber, and Esti Yeger-Lotem. The organ-disease annotations (odisea) database of hereditary diseases and inflicted tissues. *Journal of Molecular Biology*, 434(11):167619, 2022.

[21] Omer Basha, Rotem Shpringer, Chanan M Argov, and Esti Yeger-Lotem. The differentialnet database of differential protein–protein interactions in human tissues. *Nucleic acids research*, 46(D1):D522–D526, 2018.

[22] Moran Sharon, Gil Gruber, Chanan M Argov, Miri Volozhinsky, and Esti Yeger-Lotem. Proact: quantifying the differential activity of biological processes in tissues, cells, and user-defined contexts. *Nucleic Acids Research*, 51(W1):W478–W483, 2023.

[23] Takuya Akiba, Shotaro Sano, Toshihiko Yanase, Takeru Ohta, and Masanori Koyama. Optuna: A next-generation hyperparameter optimization framework. In *Proceedings of the 25th ACM SIGKDD international conference on knowledge discovery & data mining*, pages 2623–2631, 2019.

[24] Ashis Saha, Yungil Kim, Ariel DH Gewirtz, Brian Jo, Chuan Gao, Ian C McDowell, Barbara E Engelhardt, Alexis Battle, François Aguet, Kristin G Ardlie, et al. Co-expression networks reveal the tissue-specific regulation of transcription and splicing. *Genome research*, 27(11):1843–1858, 2017.

[25] The Tabula Sapiens Consortium*, Robert C Jones, Jim Karkanias, Mark A Krasnow, Angela Oliveira Pisco, Stephen R Quake, Julia Salzman, Nir Yosef, Bryan Bulthaup, Phillip Brown, et al. The tabula sapiens: A multiple-organ, single-cell transcriptomic atlas of humans. *Science*, 376(6594):eabl4896, 2022.

[26] Omer Basha, Chanan M Argov, Raviv Artzy, Yazeed Zoabi, Idan Hekselman, Liad Alfandari, Vered Chalifa-Caspi, and Esti Yeger-Lotem. Differential network analysis of multiple human tissue interactomes highlights tissue-selective processes and genetic disorder genes. *Bioinformatics*, 36(9):2821–2828, 2020.

[27] Jacob White. Pubmed 2.0. *Medical reference services quarterly*, 39(4):382–387, 2020.

[28] Gil Stelzer, Naomi Rosen, Inbar Plaschkes, Shahar Zimmerman, Michal Twik, Simon Fishilevich, Tsippi Iny Stein, Ron Nudel, Iris Lieder, Yaron Mazor, et al. The genecards suite: from gene data mining to disease genome sequence analyses. *Current protocols in bioinformatics*, 54(1):1–30, 2016.

[29] Sebastian Köhler, Michael Gargano, Nicolas Matentzoglu, Leigh C Carmody, David Lewis-Smith, Nicole A Vasilevsky, Daniel Danis, Ganna Balagura, Gareth Baynam, Amy M Brower, et al. The human phenotype ontology in 2021. *Nucleic acids research*, 49(D1):D1207–D1217, 2021.

[30] Moran Sharon, Ekaterina Vinogradov, Chanan M Argov, Or Lazarescu, Yazeed Zoabi, Idan Hekselman, and Esti Yeger-Lotem. The differential activity of biological processes in tissues and cell subsets can illuminate disease-related processes and cell-type identities. *Bioinformatics*, 38(6):1584–1592, 2022.

[31] Eyal Simonovsky, Moran Sharon, Maya Ziv, Omry Mauer, Idan Hekselman, Juman Jubran, Ekaterina Vinogradov, Chanan M Argov, Omer Basha, Lior Kerber, et al. A tissue-aware machine learning framework enhances the mechanistic understanding and genetic diagnosis of mendelian and rare diseases (in revision). *bioRxiv*, 2021.

[32] Caleb C Noble and Diane J Cook. Graph-based anomaly detection. In *Proceedings of the ninth ACM SIGKDD international conference on Knowledge discovery and data mining*, pages 631–636. ACM, 2003.

[33] Dima Kagan, Yuval Elovichi, and Michael Fire. Generic anomalous vertices detection utilizing a link prediction

algorithm. *Social Network Analysis and Mining*, 8(1):1–13, 2018.

[34] Martin Grohe. word2vec, node2vec, graph2vec, x2vec: Towards a theory of vector embeddings of structured data. In *Proceedings of the 39th ACM SIGMOD-SIGACT-SIGAI Symposium on Principles of Database Systems*, pages 1–16, 2020.

[35] Aditya Grover and Jure Leskovec. node2vec: Scalable feature learning for networks. In *Proceedings of the 22nd ACM SIGKDD international conference on Knowledge discovery and data mining*, pages 855–864, 2016.

[36] Leo Torres, Kevin S Chan, and Tina Eliassi-Rad. Glee: Geometric laplacian eigenmap embedding. *Journal of Complex Networks*, 8(2):cnaa007, 2020.

[37] Dingqi Yang, Paolo Rosso, Bin Li, and Philippe Cudre-Mauroux. Nodesketch: Highly-efficient graph embeddings via recursive sketching. In *Proceedings of the 25th ACM SIGKDD International Conference on Knowledge Discovery & Data Mining*, pages 1162–1172, 2019.

[38] Bryan Perozzi, Rami Al-Rfou, and Steven Skiena. Deepwalk: Online learning of social representations. In *Proceedings of the 20th ACM SIGKDD international conference on Knowledge discovery and data mining*, pages 701–710, 2014.

[39] Benedek Rozemberczki, Oliver Kiss, and Rik Sarkar. Karate Club: An API Oriented Open-source Python Framework for Unsupervised Learning on Graphs. In *Proceedings of the 29th ACM International Conference on Information and Knowledge Management (CIKM '20)*, page 3125–3132. ACM, 2020.

[40] Tianqi Chen and Carlos Guestrin. Xgboost: A scalable tree boosting system. In *Proceedings of the 22nd acm sigkdd international conference on knowledge discovery and data mining*, pages 785–794, 2016.

[41] Guolin Ke, Qi Meng, Thomas Finley, Taifeng Wang, Wei Chen, Weidong Ma, Qiwei Ye, and Tie-Yan Liu. Lightgbm: A highly efficient gradient boosting decision tree. *Advances in neural information processing systems*, 30, 2017.

[42] Charu C Aggarwal. *Outlier Analysis*, volume 1. Springer, 2015.

[43] Fei Tony Liu, Kai Ming Ting, and Zhi-Hua Zhou. Isolation forest. In *2008 eighth ieee international conference on data mining*, pages 413–422. IEEE, 2008.

[44] Tom Fawcett. An introduction to roc analysis. *Pattern recognition letters*, 27(8):861–874, 2006.

[45] Kendrick Boyd, Kevin H Eng, and C David Page. Area under the precision-recall curve: point estimates and confidence intervals. In *Machine Learning and Knowledge Discovery in Databases: European Conference, ECML PKDD 2013, Prague, Czech Republic, September 23-27, 2013, Proceedings, Part III 13*, pages 451–466. Springer, 2013.

[46] Yoav Benjamini and Yosef Hochberg. Controlling the false discovery rate: a practical and powerful approach to multiple testing. *Journal of the Royal statistical society: series B (Methodological)*, 57(1):289–300, 1995.

Supplementary Materials

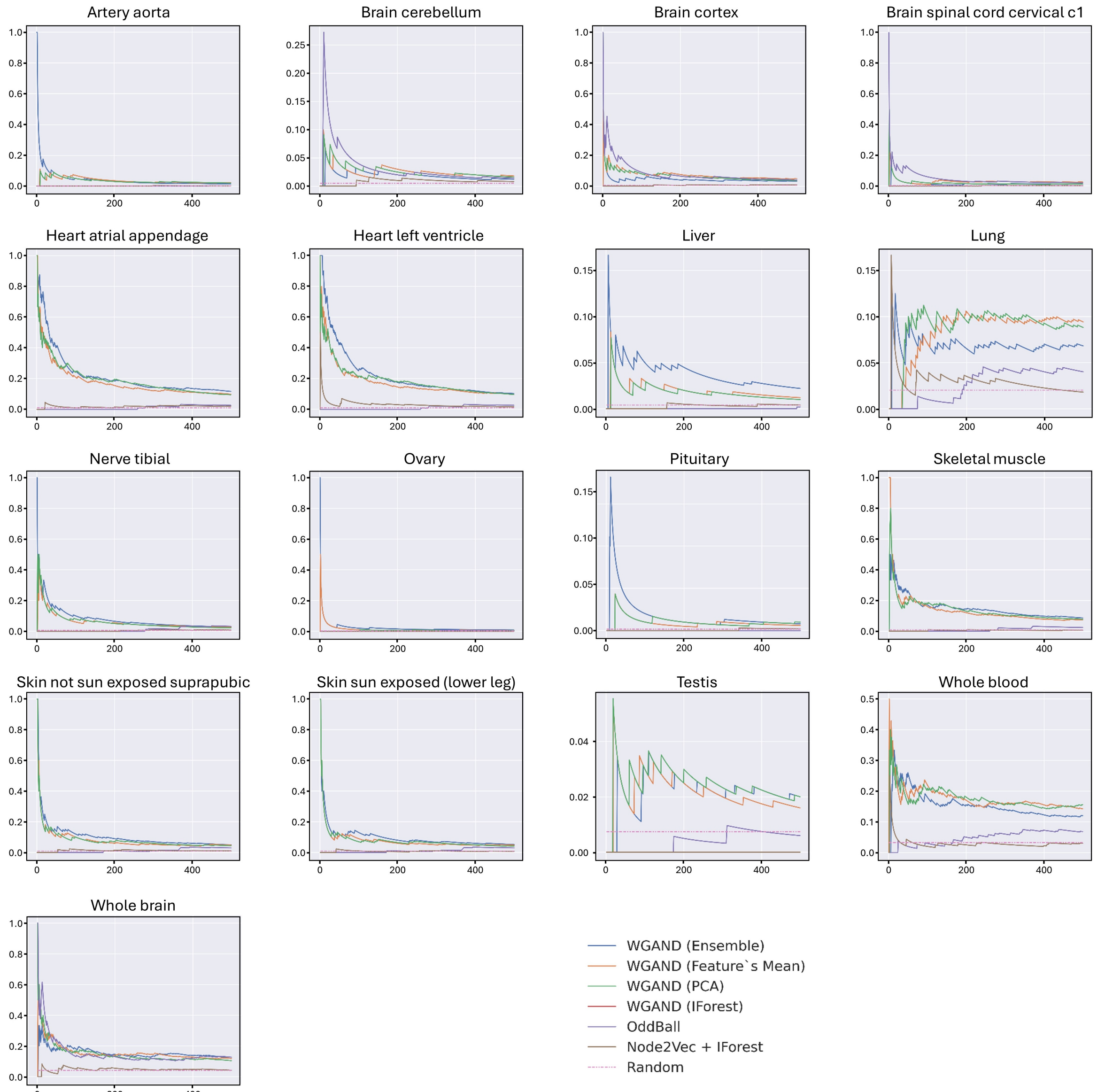

Figure S1. P@K of different classifiers per each PPI tissue-specific network.

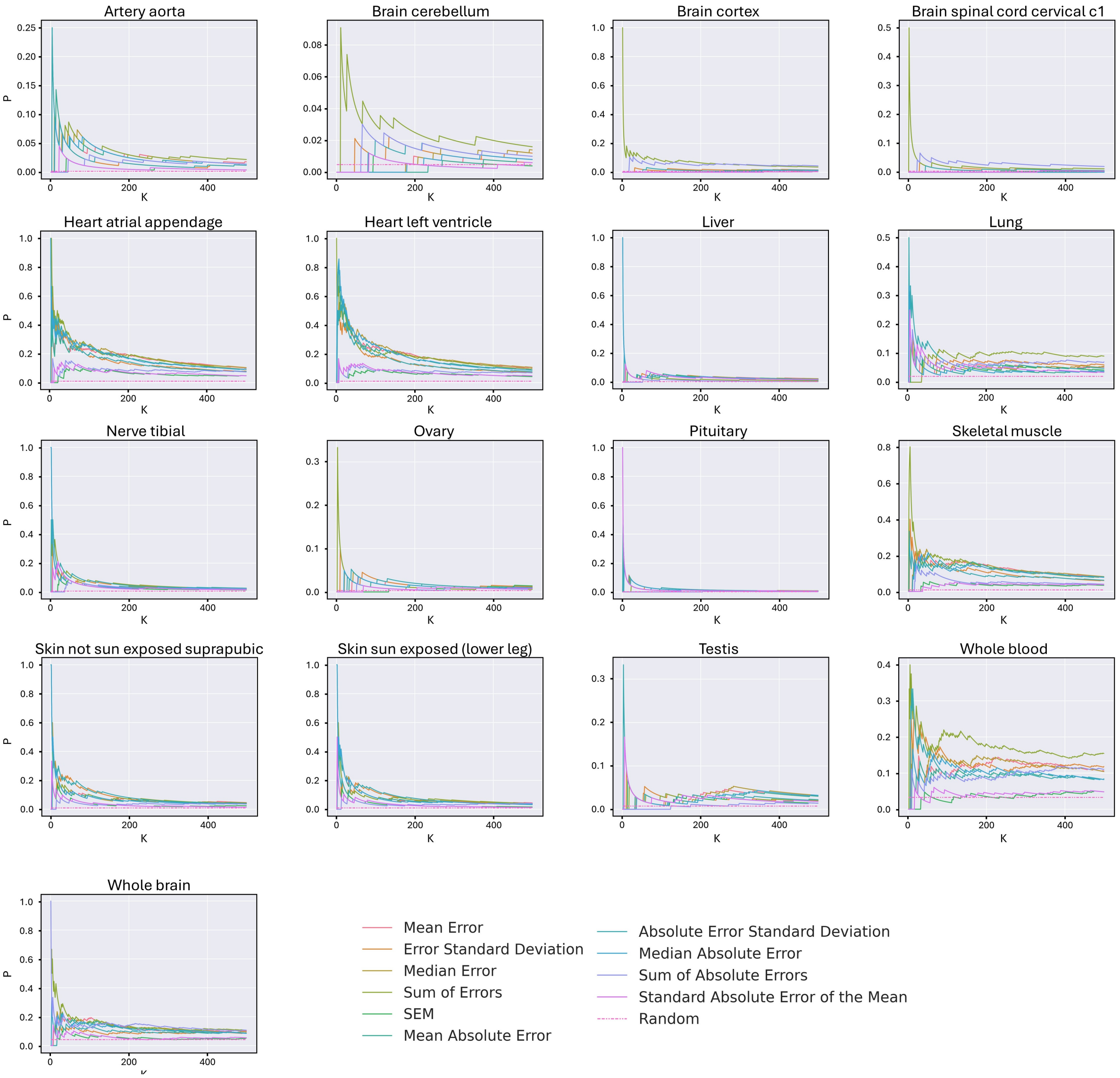

Figure S2. P@K of WGAND 'ensemble' method trained with each feature separately on PPI tissue-specific networks.

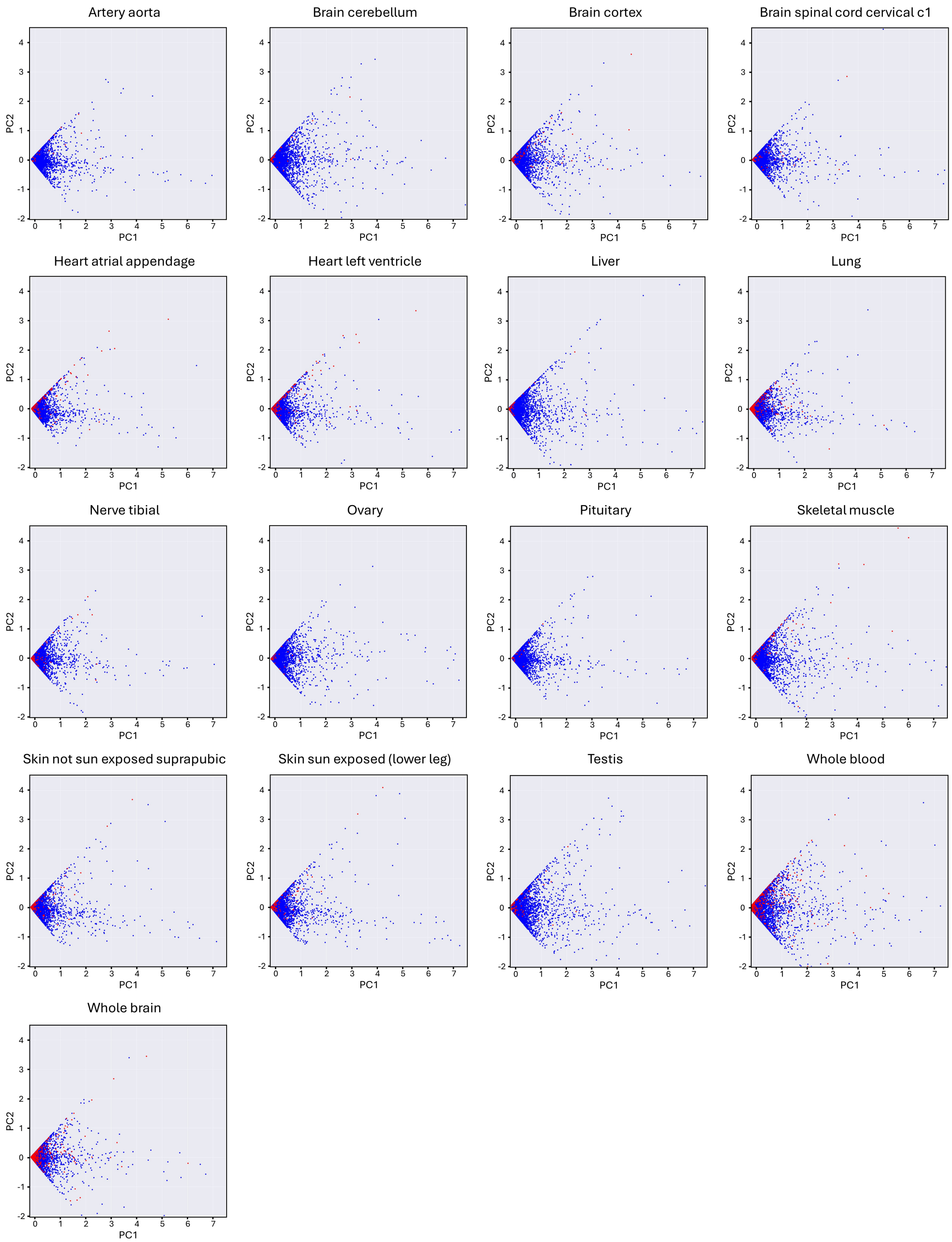

Figure S3. **PCA representation of the anomaly detection features.** The red dots represent disease-related proteins; the blue dots represent all other proteins.

Table S1. Evaluation WGAND versus baselines on PPI dataset

Tissue Network	WGAND (Ensemble)						Node2Vec + Iforest						Oddball					
	AUC	PR-AUC	P@1	P@3	P@10	P@20	AUC	PR-AUC	P@1	P@3	P@10	P@20	AUC	PR-AUC	P@1	P@3	P@10	P@20
Artery Aorta	**0.83**	**0.11**	**1.00**	**0.67**	**0.20**	**0.15**	0.46	0.00	0.00	0.00	0.00	0.00	0.70	0.00	0.00	0.00	0.00	0.00
Brain Cerebellum	**0.56**	**0.01**	0.00	0.00	0.00	0.05	0.52	**0.01**	0.00	0.00	0.00	0.00	0.53	**0.01**	0.00	0.00	**0.20**	**0.15**
Brain Cortex	**0.72**	**0.02**	0.00	**0.33**	0.10	0.05	0.53	0.01	0.00	0.00	0.00	0.00	0.68	**0.05**	**1.00**	**0.33**	**0.40**	**0.25**
Brain Spinal cord cervical c 1	**0.71**	**0.01**	0.00	0.00	0.10	0.05	0.52	0.00	0.00	0.00	0.00	0.05	0.70	**0.04**	**1.00**	**0.33**	**0.20**	**0.10**
Heart Atrial Appendage	**0.78**	**0.18**	**1.00**	**1.00**	**0.70**	**0.65**	0.56	0.01	0.00	0.00	0.00	0.00	0.63	0.03	0.00	0.00	0.00	0.00
Heart Left Ventricle	**0.76**	**0.18**	**1.00**	**1.00**	**0.90**	**0.80**	0.54	0.02	0.00	0.00	0.00	0.00	0.62	0.03	0.00	0.00	0.00	0.00
Liver	**0.55**	**0.01**	0.00	0.00	0.00	**0.10**	0.42	0.00	0.00	0.00	0.00	0.00	0.41	0.00	0.00	0.00	0.00	0.00
Lung	**0.69**	**0.05**	**0.00**	**0.00**	**0.10**	**0.10**	0.52	0.02	0.00	0.00	0.10	0.05	0.60	0.03	0.00	0.00	0.00	0.00
Muscle Skeletal	**0.73**	**0.09**	**1.00**	**0.67**	**0.50**	**0.40**	0.54	0.01	0.00	0.00	0.00	0.00	0.56	0.02	0.00	0.00	0.00	0.00
Nerve Tibial	**0.63**	**0.05**	**1.00**	**0.33**	**0.30**	**0.30**	0.51	0.01	0.00	0.00	0.00	0.00	0.59	0.01	0.00	0.00	0.00	0.00
Ovary	**0.52**	**0.03**	**1.00**	**0.33**	**0.10**	**0.05**	0.51	0.00	0.00	0.00	0.00	0.00	0.52	0.00	0.00	0.00	0.00	0.00
Pituitary	**0.63**	**0.01**	**0.00**	**0.00**	**0.20**	**0.10**	0.52	0.00	0.00	0.00	0.00	0.00	0.53	0.00	0.00	0.00	0.00	0.00
Skin Not Sun Exposed Suprapubic	**0.69**	**0.06**	**1.00**	**0.67**	**0.30**	**0.20**	0.54	0.01	0.00	0.00	0.00	0.00	0.62	0.02	0.00	0.00	0.00	0.00
Skin Sun Exposed Lower leg	**0.67**	**0.05**	**1.00**	**0.67**	**0.30**	**0.20**	0.52	0.01	0.00	0.00	0.00	0.00	0.62	0.02	0.00	0.00	0.00	0.00
Testis	**0.63**	**0.01**	**0.00**	**0.00**	**0.00**	**0.00**	0.51	**0.01**	0.00	0.00	0.00	0.00	0.57	**0.01**	0.00	0.00	0.00	0.00
Whole Blood	**0.69**	**0.08**	**1.00**	**0.33**	**0.20**	**0.25**	0.54	0.04	0.00	0.00	0.10	0.15	0.61	0.06	0.00	0.00	0.00	0.00
Whole Brain	**0.61**	0.07	0.00	0.33	0.30	0.20	0.53	0.04	0.00	0.00	0.00	0.00	0.56	**0.08**	**1.00**	**0.67**	**0.50**	**0.40**
Average	**0.67**	**0.06**	**0.53**	**0.37**	**0.25**	**0.21**	0.52	0.01	0.00	0.00	0.01	0.01	0.59	0.02	0.18	0.08	0.08	0.05

Table S2. P@K of WGAND 'ensemble' method constructed per feature.

Tissue Network	Mean Error				Error Standard Deviation				Median Error				Sum of Errors			
	P@1	P@3	P@10	P@20	P@1	P@3	P@10	P@20	P@1	P@3	P@10	P@20	P@1	P@3	P@10	P@20
Artery Aorta	0.00	0.00	**0.10**	**0.10**	0.00	0.00	**0.10**	0.05	0.00	0.00	0.00	0.05	0.00	0.00	**0.10**	0.05
Brain Cerebellum	0.00	0.00	0.00	0.00	0.00	0.00	0.00	0.00	0.00	0.00	0.00	0.00	0.00	0.00	0.00	**0.05**
Brain Cortex	0.00	0.00	0.00	0.00	0.00	0.00	0.00	0.00	0.00	0.00	0.00	0.00	**1.00**	**0.33**	**0.10**	**0.15**
Brain Spinal cord cervical c 1	0.00	0.00	0.00	0.00	0.00	0.00	0.00	0.00	0.00	0.00	0.00	0.00	0.00	**0.33**	**0.10**	**0.05**
Heart Atrial Appendage	**1.00**	0.33	0.20	0.35	0.00	0.33	0.40	0.40	**1.00**	0.67	0.40	0.35	**1.00**	**1.00**	**0.50**	**0.45**
Heart Left Ventricle	0.00	**0.67**	0.60	0.50	0.00	0.33	0.40	0.40	0.00	**0.67**	0.70	**0.55**	**1.00**	**0.67**	0.50	0.50
Liver	**1.00**	**0.33**	**0.10**	**0.05**	0.00	0.00	0.10	**0.05**	**1.00**	**0.33**	**0.10**	0.05	0.00	0.00	0.00	**0.05**
Lung	0.00	**0.33**	0.10	0.05	0.00	**0.33**	0.10	0.10	0.00	**0.33**	0.10	0.05	0.00	0.00	0.00	0.00
Muscle Skeletal	0.00	0.00	0.00	0.15	0.00	0.00	0.30	0.20	0.00	0.00	0.00	0.15	0.00	**0.67**	**0.40**	**0.25**
Nerve Tibial	**1.00**	**0.67**	0.20	0.10	0.00	0.00	0.20	0.15	**1.00**	**0.67**	**0.20**	0.10	0.00	0.33	**0.30**	**0.20**
Ovary	0.00	0.00	0.00	0.00	0.00	0.00	**0.10**	**0.05**	0.00	0.00	0.00	0.00	0.00	**0.33**	**0.10**	**0.05**
Pituitary	0.00	0.00	**0.10**	**0.10**	0.00	**0.33**	**0.10**	0.05	0.00	0.00	**0.10**	**0.10**	0.00	0.00	0.00	0.00
Skin Not Sun Exposed Suprapubic	**1.00**	**0.67**	**0.30**	0.20	0.00	0.00	0.10	0.20	**1.00**	**0.67**	**0.30**	0.20	**1.00**	**0.67**	**0.30**	0.15
Skin Sun Exposed Lower leg	**1.00**	**0.67**	**0.40**	**0.25**	0.00	0.33	0.10	0.15	**1.00**	**0.67**	**0.40**	**0.25**	**1.00**	**0.67**	0.30	0.15
Testis	0.00	0.00	0.00	0.00	0.00	0.00	0.00	**0.05**	0.00	0.00	0.00	0.00	0.00	0.00	0.00	**0.05**
Whole Blood	0.00	0.00	0.10	0.05	0.00	0.00	0.20	0.20	0.00	0.00	0.10	0.10	0.00	**0.33**	**0.30**	**0.25**
Whole Brain	0.00	0.33	0.10	0.15	0.00	0.00	0.10	0.10	0.00	0.33	0.10	0.20	**1.00**	**0.67**	**0.40**	**0.30**
Average	0.29	0.24	0.14	0.12	0.00	0.10	0.14	0.13	0.29	0.25	0.15	0.13	**0.35**	**0.35**	**0.20**	**0.16**

Tissue Network	SEM				Mean Absolute Error				Absolute Error Standard Deviation				Median Absolute Error				Sum of Absolute Errors				Standard Absolute Error of the Mean			
	P@1	P@3	P@10	P@20	P@1	P@3	P@10	P@20	P@1	P@3	P@10	P@20	P@1	P@3	P@10	P@20	P@1	P@3	P@10	P@20	P@1	P@3	P@10	P@20
Artery Aorta	0.00	0.00	0.00	0.00	0.00	0.00	**0.10**	**0.10**	0.00	0.00	**0.10**	0.05	0.00	0.00	0.00	0.05	0.00	0.00	0.00	0.00	0.00	0.00	0.00	0.00
Brain Cerebellum	0.00	0.00	0.00	0.00	0.00	0.00	0.00	0.00	0.00	0.00	0.00	0.00	0.00	0.00	0.00	0.00	0.00	0.00	0.00	0.00	0.00	0.00	0.00	0.00
Brain Cortex	0.00	0.00	0.00	0.00	0.00	0.00	0.00	0.00	0.00	0.00	0.00	0.00	0.00	0.00	0.00	0.00	0.00	0.00	0.00	0.10	0.00	0.00	0.00	0.00
Brain Spinal cord cervical c 1	0.00	0.00	0.00	0.00	0.00	0.00	0.00	0.00	0.00	0.00	0.00	0.00	0.00	0.00	0.00	0.00	0.00	0.00	0.00	0.00	0.00	0.00	0.00	0.00
Heart Atrial Appendage	0.00	0.00	0.00	0.05	**1.00**	0.33	0.20	0.35	0.00	0.33	0.40	**0.45**	**1.00**	0.67	0.40	0.35	0.00	0.00	0.10	0.05	0.00	0.00	0.10	0.10
Heart Left Ventricle	0.00	0.00	0.10	0.05	0.00	**0.67**	0.60	0.50	0.00	0.33	0.50	0.40	0.00	**0.67**	**0.70**	**0.55**	0.00	0.00	0.10	0.05	0.00	0.00	0.10	0.10
Liver	0.00	0.00	0.00	**0.05**	**1.00**	**0.33**	**0.10**	**0.05**	0.00	0.00	**0.10**	**0.05**	**1.00**	**0.33**	**0.10**	0.05	0.00	0.00	0.00	0.00	0.00	0.00	**0.10**	**0.05**
Lung	0.00	0.00	0.10	0.10	0.00	**0.33**	0.10	0.05	0.00	**0.33**	**0.30**	**0.15**	0.00	**0.33**	0.10	0.05	0.00	0.00	0.10	0.10	0.00	0.00	0.10	0.10
Muscle Skeletal	0.00	0.00	0.00	0.00	0.00	0.00	0.00	0.15	0.00	0.33	0.20	0.15	0.00	0.00	0.00	0.15	0.00	0.00	0.00	0.10	0.00	0.00	0.00	0.00
Nerve Tibial	0.00	0.00	0.00	0.05	**1.00**	**0.67**	0.20	0.15	0.00	0.33	**0.30**	**0.20**	**1.00**	**0.67**	0.20	0.10	0.00	0.00	0.00	0.00	0.00	0.00	0.10	**0.20**
Ovary	0.00	0.00	0.00	0.00	0.00	0.00	0.00	0.00	0.00	0.00	0.00	0.00	0.00	0.00	0.00	**0.05**	0.00	0.00	0.00	0.00	0.00	0.00	0.00	0.00
Pituitary	**1.00**	**0.33**	**0.10**	0.05	0.00	0.00	**0.10**	**0.10**	0.00	**0.33**	**0.10**	0.05	0.00	0.00	**0.10**	0.05	0.00	0.00	0.00	0.00	**1.00**	**0.33**	**0.10**	0.05
Skin Not Sun Exposed Suprapubic	0.00	0.00	0.10	0.15	**1.00**	**0.67**	**0.30**	0.20	0.00	0.00	**0.30**	**0.30**	**1.00**	**0.67**	**0.30**	0.20	0.00	0.00	0.00	0.05	0.00	0.33	0.10	0.10
Skin Sun Exposed Lower leg	0.00	0.33	0.10	0.10	**1.00**	**0.67**	**0.40**	**0.25**	0.00	0.33	0.20	0.15	**1.00**	**0.67**	**0.40**	**0.25**	0.00	0.00	0.00	0.05	0.00	0.33	0.10	0.10
Testis	0.00	0.00	0.00	0.05	0.00	0.00	0.00	0.00	0.00	**0.33**	**0.10**	**0.05**	0.00	0.00	0.00	0.00	0.00	0.00	0.00	0.00	0.00	0.00	**0.10**	**0.05**
Whole Blood	0.00	0.00	0.00	0.00	0.00	0.00	0.10	0.05	0.00	0.00	**0.30**	0.20	0.00	0.00	0.10	0.10	0.00	0.00	0.10	0.05	0.00	0.00	0.00	0.05
Whole Brain	0.00	0.00	0.00	0.05	0.00	0.33	0.10	0.15	0.00	0.00	0.00	0.10	0.00	0.33	0.10	0.20	**1.00**	0.33	0.20	0.15	0.00	0.00	0.10	0.10
Average	0.06	0.04	0.03	0.04	0.29	0.24	0.14	0.12	0.00	0.16	0.17	0.14	0.29	0.25	0.15	0.13	0.06	0.02	0.04	0.04	0.06	0.06	0.06	0.06

Table S3. The top-ten genes predicted by the ensemble methods and the diseases that these genes were related to.

Protein	Tissue Network	Disease-Related Protein	Anomalous Protein	Diseases
ENSG00000061455	Artery Aorta	1	1	Patent Ductus Arteriosus 3
ENSG00000107796	Artery Aorta	1	1	Aortic Aneurysm, Familial Thoracic 6
ENSG00000182492	Artery Aorta	0	0	-
ENSG00000115414	Artery Aorta	0	0	-
ENSG00000159251	Artery Aorta	0	0	-
ENSG00000136999	Artery Aorta	0	0	-
ENSG00000077943	Artery Aorta	0	0	-
ENSG00000135324	Artery Aorta	0	0	-
ENSG00000154553	Artery Aorta	0	0	-
ENSG00000133026	Artery Aorta	0	0	-
ENSG00000131095	Brain Cerebellum	0	1	Alexander Disease
ENSG00000104833	Brain Cerebellum	0	1	Leukodystrophy, Hypomyelinating, 6, Dystonia 4, Torsion, Autosomal Dominant
ENSG00000089199	Brain Cerebellum	0	0	-
ENSG00000154127	Brain Cerebellum	0	0	-
ENSG00000105613	Brain Cerebellum	0	1	Mega-Corpus-Callosum Syndrome With Cerebellar Hypoplasia And Cortical Malformations, Cerebellar Hypoplasia
ENSG00000101210	Brain Cerebellum	0	0	-
ENSG00000171885	Brain Cerebellum	0	0	-
ENSG00000188827	Brain Cerebellum	0	0	-
ENSG00000130540	Brain Cerebellum	0	0	-
ENSG00000132639	Brain Cerebellum	0	0	-
ENSG00000131095	Brain Cortex	0	1	Alexander Disease
ENSG00000156475	Brain Cortex	1	0	Spinocerebellar Ataxia 12, Autosomal Dominant Cerebellar Ataxia
ENSG00000130540	Brain Cortex	0	0	-
ENSG00000171885	Brain Cortex	0	1	Brain Edema
ENSG00000129990	Brain Cortex	0	0	-
ENSG00000104833	Brain Cortex	0	0	-
ENSG00000075340	Brain Cortex	0	0	-
ENSG00000127585	Brain Cortex	0	0	-
ENSG00000064787	Brain Cortex	0	0	-
ENSG00000089169	Brain Cortex	0	0	-
ENSG00000064787	Brain Spinal cord cervical c 1	0	0	-
ENSG00000131095	Brain Spinal cord cervical c 1	0	1	Alexander Disease
ENSG00000123560	Brain Spinal cord cervical c 1	0	1	Pelizaeus-Merzbacher Disease, Spastic Paraplegia 2, X-Linked
ENSG00000197971	Brain Spinal cord cervical c 1	0	1	Secondary Progressive Multiple Sclerosis, Demyelinating Disease

Continued on next page

Table S3 – *Continued from previous page*

Protein	Tissue Network	Disease-Related Protein	Anomalous Protein	Diseases
ENSG00000171885	Brain Spinal cord cervical c 1	0	1	Neuromyelitis Optica
ENSG00000105695	Brain Spinal cord cervical c 1	0	1	Polyneuropathy
ENSG00000156475	Brain Spinal cord cervical c 1	1	1	Spinocerebellar Ataxia 12, Autosomal Dominant Cerebellar Ataxia
ENSG00000104833	Brain Spinal cord cervical c 1	0	0	-
ENSG00000160307	Brain Spinal cord cervical c 1	0	1	Syringoma, Neurofibroma
ENSG00000112280	Brain Spinal cord cervical c 1	0	0	-
ENSG00000134571	Heart Atrial Appendage	1	1	Cardiomyopathy, Familial Hypertrophic, 4
ENSG00000159251	Heart Atrial Appendage	1	1	Atrial Septal Defect 5, Cardiomyopathy, Familial Hypertrophic, 11
ENSG00000175206	Heart Atrial Appendage	1	1	Atrial Standstill 2, Atrial Fibrillation, Familial, 6
ENSG00000120937	Heart Atrial Appendage	0	0	-
ENSG00000077522	Heart Atrial Appendage	1	1	Cardiomyopathy, Dilated, 1Aa, With Or Without Left Ventricular Noncompaction, Myopathy, Distal, 6, Adult-Onset, Autosomal Dominant
ENSG00000155657	Heart Atrial Appendage	1	1	Myopathy, Myofibrillar, 9, With Early Respiratory Failure, Congenital Myopathy 5 With Cardiomyopathy
ENSG00000118194	Heart Atrial Appendage	1	1	Cardiomyopathy, Dilated, 1D, Cardiomyopathy, Familial Hypertrophic, 2
ENSG00000173991	Heart Atrial Appendage	1	1	Cardiomyopathy, Familial Hypertrophic, 25, Muscular Dystrophy, Limb-Girdle, Autosomal Recessive 7
ENSG00000104879	Heart Atrial Appendage	0	0	-
ENSG00000198523	Heart Atrial Appendage	1	1	Cardiomyopathy, Dilated, 1P, Cardiomyopathy, Familial Hypertrophic, 18
ENSG00000134571	Heart Left Ventricle	1	1	Cardiomyopathy, Familial Hypertrophic, 4, Left Ventricular Noncompaction 10
ENSG00000159251	Heart Left Ventricle	1	1	Atrial Septal Defect 5, Cardiomyopathy, Familial Hypertrophic, 11
ENSG00000092054	Heart Left Ventricle	1	1	Myopathy, Distal, 1, Congenital Myopathy 7A, Myosin Storage, Autosomal Dominant
ENSG00000077522	Heart Left Ventricle	1	1	Cardiomyopathy, Dilated, 1Aa, With Or Without Left Ventricular Noncompaction, Myopathy, Distal, 6, Adult-Onset, Autosomal Dominant

Continued on next page

Table S3 – *Continued from previous page*

Protein	Tissue Network	Disease-Related Protein	Anomalous Protein	Diseases
ENSG00000155657	Heart Left Ventricle	1	1	Myopathy, Myofibrillar, 9, With Early Respiratory Failure, Congenital Myopathy 5 With Cardiomyopathy
ENSG00000118194	Heart Left Ventricle	1	1	Cardiomyopathy, Dilated, 1D, Cardiomyopathy, Familial Hypertrophic, 2
ENSG00000129991	Heart Left Ventricle	1	1	Cardiomyopathy, Dilated, 2A, Cardiomyopathy, Familial Hypertrophic, 7
ENSG00000143632	Heart Left Ventricle	0	0	-
ENSG00000186439	Heart Left Ventricle	1	1	Cardiac Arrhythmia Syndrome, With Or Without Skeletal Muscle Weakness, Catecholaminergic Polymorphic Ventricular Tachycardia 5
ENSG00000114854	Heart Left Ventricle	1	1	Cardiomyopathy, Familial Hypertrophic, 13, Cardiomyopathy, Dilated, 1Z
ENSG00000171557	Liver	0	1	†Afibrinogenemia, Congenital
ENSG00000171564	Liver	0	1	†Afibrinogenemia, Congenital
ENSG00000163631	Liver	0	1	Analbuminemia, Hyperthyroxinemia, Familial Dysalbuminemic
ENSG00000118137	Liver	0	1	Hypoalphalipoproteinemia, Primary, 2, Hypoalphalipoproteinemia, Primary, 2, Intermediate
ENSG00000124253	Liver	0	1	Phosphoenolpyruvate Carboxykinase Deficiency, Cytosolic, Pepck 1 Deficiency
ENSG00000198650	Liver	1	1	Tyrosinemia, Type Ii, Tyrosinemia
ENSG00000145321	Liver	0	1	Hepatic Encephalopathy
ENSG00000171759	Liver	0	1	Phenylketonuria, Hyperphenylalaninemia
ENSG00000257017	Liver	0	1	Anhaptoglobinemia
ENSG00000197249	Liver	0	1	Alpha-1-Antitrypsin Deficiency
ENSG00000187908	Lung	0	0	-
ENSG00000182010	Lung	0	0	-
ENSG00000171885	Lung	0	0	-
ENSG00000175899	Lung	0	0	-
ENSG00000171345	Lung	0	0	-
ENSG00000133661	Lung	0	1	Extrinsic Allergic Alveolitis, Pulmonary Alveolar Proteinosis
ENSG00000197249	Lung	0	1	Alpha-1-Antitrypsin Deficiency, Hemorrhagic Disease Due To Alpha-1-Antitrypsin Pittsburgh Mutation
ENSG00000168484	Lung	1	1	

Continued on next page

Table S3 – *Continued from previous page*

Protein	Tissue Network	Disease-Related Protein	Anomalous Protein	Diseases
ENSG00000211896	Lung	0	0	-
ENSG00000165140	Lung	0	0	-
ENSG00000130595	Muscle Skeletal	0	1	Arthrogryposis, Distal, Type 2B2, Arthrogryposis, Distal, Type 1A
ENSG00000183091	Muscle Skeletal	1	1	Nemaline Myopathy 2, Arthrogryposis Multiplex Congenita 6
ENSG00000130957	Muscle Skeletal	0	0	-
ENSG00000105048	Muscle Skeletal	1	1	Nemaline Myopathy 5, Nemaline Myopathy
ENSG00000197893	Muscle Skeletal	0	1	Myopathy, Myofibrillar, 5, Myopathy, Myofibrillar, 4
ENSG00000069869	Muscle Skeletal	0	0	-
ENSG00000143632	Muscle Skeletal	1	1	Myopathy, Scapulohumeroperoneal, Congenital Myopathy 2A, Typical, Autosomal Dominant
ENSG00000155657	Muscle Skeletal	1	1	Myopathy, Myofibrillar, 9, With Early Respiratory Failure, Congenital Myopathy 5 With Cardiomyopathy
ENSG00000086967	Muscle Skeletal	0	1	Lethal Congenital Contracture Syndrome 4, Nemaline Myopathy 9
ENSG00000186439	Muscle Skeletal	0	0	-
ENSG00000105227	Nerve Tibial	1	1	Charcot-Marie-Tooth Disease, Demyelinating, Type 4F, Hypertrophic Neuropathy Of Dejerine-Sottas
ENSG00000102385	Nerve Tibial	0	1	Charcot-Marie-Tooth Disease, Charcot-Marie-Tooth Disease, Demyelinating, Type 4F
ENSG00000064787	Nerve Tibial	0	0	-
ENSG00000158887	Nerve Tibial	1	1	Hypertrophic Neuropathy Of Dejerine-Sottas, Charcot-Marie-Tooth Disease, Demyelinating, Type 1B
ENSG00000181092	Nerve Tibial	0	0	-
ENSG00000109099	Nerve Tibial	1	1	Charcot-Marie-Tooth Disease And Deafness, Charcot-Marie-Tooth Disease, Demyelinating, Type 1A
ENSG00000171345	Nerve Tibial	0	0	-
ENSG00000064300	Nerve Tibial	0	0	-
ENSG00000160307	Nerve Tibial	0	1	Neurofibroma
ENSG00000197971	Nerve Tibial	0	0	-
ENSG00000136931	Ovary	1	1	46,Xx Sex Reversal 4, Premature Ovarian Failure 7

Continued on next page

Table S3 – *Continued from previous page*

Protein	Tissue Network	Disease-Related Protein	Anomalous Protein	Diseases
ENSG00000180447	Ovary	0	0	-
ENSG00000156475	Ovary	0	0	-
ENSG00000185070	Ovary	0	0	-
ENSG00000107317	Ovary	0	0	-
ENSG00000198300	Ovary	0	0	-
ENSG00000143768	Ovary	0	1	Infertility
ENSG00000125398	Ovary	0	0	-
ENSG00000185559	Ovary	0	0	-
ENSG00000117425	Ovary	0	0	-
ENSG00000089199	Pituitary	0	1	Pheochromocytoma
ENSG00000069011	Pituitary	0	0	-
ENSG00000135346	Pituitary	0	0	-
ENSG00000136931	Pituitary	0	0	-
ENSG00000124253	Pituitary	0	0	-
ENSG00000129990	Pituitary	0	0	-
ENSG00000170421	Pituitary	0	0	-
ENSG00000149295	Pituitary	0	0	-
ENSG00000064835	Pituitary	1	1	Pituitary Hormone Deficiency, Combined Or Isolated, 1, Isolated Growth Hormone Deficiency, Type Ii
ENSG00000105894	Pituitary	0	0	-
ENSG00000167768	Skin Not Sun Exposed Suprapubic	1	1	Palmoplantar Keratoderma, Nonepidermolytic, Ichthyosis Hystrix, Curth-Macklin Type
ENSG00000186081	Skin Not Sun Exposed Suprapubic	1	1	Epidermolysis Bullosa Simplex 2F, With Mottled Pigmentation, Epidermolysis Bullosa Simplex 2E, With Migratory Circinate Erythema
ENSG00000186847	Skin Not Sun Exposed Suprapubic	1	1	Dermatopathia Pigmentosa Reticularis, Naegeli-Franceschetti-Jadassohn Syndrome
ENSG00000096696	Skin Not Sun Exposed Suprapubic	0	1	Skin Fragility-Woolly Hair Syndrome, Epidermolysis Bullosa, Lethal Acantholytic
ENSG00000172867	Skin Not Sun Exposed Suprapubic	0	1	Ichthyosis Bullosa Of Siemens, Epidermolytic Hyperkeratosis
ENSG00000171346	Skin Not Sun Exposed Suprapubic	0	1	Morpheaform Basal Cell Carcinoma, Infiltrative Basal Cell Carcinoma
ENSG00000161634	Skin Not Sun Exposed Suprapubic	0	0	-
ENSG00000163207	Skin Not Sun Exposed Suprapubic	0	1	Cholesteatoma Of Middle Ear, Porokeratosis

Continued on next page

Table S3 – *Continued from previous page*

Protein	Tissue Network	Disease-Related Protein	Anomalous Protein	Diseases
ENSG00000081277	Skin Not Sun Exposed Suprapubic	0	1	Ectodermal Dysplasia/Skin Fragility Syndrome, Ectodermal Dysplasia
ENSG00000178372	Skin Not Sun Exposed Suprapubic	0	0	-
ENSG00000167768	Skin Sun Exposed Lower leg	1	1	Palmoplantar Keratoderma, Nonepidermolytic, Ichthyosis Hystrix, Curth-Macklin Type
ENSG00000186081	Skin Sun Exposed Lower leg	1	1	Epidermolysis Bullosa Simplex 2F, With Mottled Pigmentation, Epidermolysis Bullosa Simplex 2E, With Migratory Circinate Erythema
ENSG00000096696	Skin Sun Exposed Lower leg	0	1	Skin Fragility-Woolly Hair Syndrome, Epidermolysis Bullosa, Lethal Acantholytic
ENSG00000172867	Skin Sun Exposed Lower leg	0	1	Ichthyosis Bullosa Of Siemens, Epidermolytic Hyperkeratosis
ENSG00000161634	Skin Sun Exposed Lower leg	0	0	-
ENSG00000186847	Skin Sun Exposed Lower leg	1	1	Dermatopathia Pigmentosa Reticularis, Naegeli-Franceschetti-Jadassohn Syndrome
ENSG00000163207	Skin Sun Exposed Lower leg	0	0	-
ENSG00000171346	Skin Sun Exposed Lower leg	0	1	Morpheaform Basal Cell Carcinoma, Infiltrative Basal Cell Carcinoma
ENSG00000081277	Skin Sun Exposed Lower leg	0	1	Ectodermal Dysplasia/Skin Fragility Syndrome, Ectodermal Dysplasia
ENSG00000149418	Skin Sun Exposed Lower leg	1	1	Ichthyosis, Congenital, Autosomal Recessive 11, Ichthyosis
ENSG00000118137	Testis	0	0	-
ENSG00000131747	Testis	0	0	-
ENSG00000198033	Testis	0	0	-
ENSG00000174015	Testis	0	0	-
ENSG00000170777	Testis	0	0	-
ENSG00000168454	Testis	0	0	-
ENSG00000183207	Testis	0	0	-
ENSG00000166851	Testis	0	0	-
ENSG00000133101	Testis	0	1	Testicular Cancer
ENSG00000117399	Testis	0	0	-
ENSG00000148346	Whole Blood	0	0	-
ENSG00000104918	Whole Blood	0	0	-
ENSG00000124731	Whole Blood	0	0	-
ENSG00000132965	Whole Blood	0	0	-
ENSG00000143546	Whole Blood	0	0	-

Continued on next page

Table S3 – *Continued from previous page*

Protein	Tissue Network	Disease-Related Protein	Anomalous Protein	Diseases
ENSG00000141480	Whole Blood	0	0	-
ENSG00000188536	Whole Blood	1	1	Hemoglobin H Disease, Alpha-Thalassemia
ENSG00000100985	Whole Blood	0	0	-
ENSG00000140368	Whole Blood	1	1	Pyogenic Sterile Arthritis, Pyoderma Gangrenosum, And Acne
ENSG00000066336	Whole Blood	0	0	-
ENSG00000131095	Whole Brain	0	1	Alexander Disease
ENSG00000171885	Whole Brain	0	1	Neuromyelitis Optica
ENSG00000156475	Whole Brain	1	1	Spinocerebellar Ataxia 12, Autosomal Dominant Cerebellar Ataxia
ENSG00000064787	Whole Brain	0	0	-
ENSG00000130540	Whole Brain	0	0	-
ENSG00000104833	Whole Brain	1	1	Leukodystrophy, Hypomyelinating, 6, Dystonia 4, Torsion, Autosomal Dominant
ENSG00000129990	Whole Brain	0	0	-
ENSG00000089169	Whole Brain	0	0	-
ENSG00000167971	Whole Brain	0	0	-
ENSG00000155980	Whole Brain	1	1	Spastic Paraplegia 10, Autosomal Dominant, Myoclonus, Intractable, Neonatal

Click here to access/download
Supplementary Material
Response Letter GigaScience.pdf

Cover Letter
Click here to access/download;Personal Cover;GigaScience - Cover letter resubmission.pdf

Dear GigaScience Editorial Team,

I am writing to resubmit our revised manuscript titled Network-based anomaly detection algorithm reveals proteins with major roles in human tissues" for consideration in GigaScience. Following the valuable feedback provided by the reviewers on our initial submission (GIGA-D-23-00401), and in our correspondence with Dr. Scott Edmunds, we have made substantial revisions to enhance the clarity, scope, and impact of our work.

We have undertaken the following major revisions:

- **Manuscript Focus:** We have restructured the manuscript to align more closely with GigaScience's biomedical focus. This involved refining the bioinformatics aspects and emphasizing the relevance of our work to the biomedical community.

- **Comparative Analysis:** We compared our algorithm, WGAND, against various state-of-the-art solutions. The obtained results, detailed in the revised manuscript, demonstrate that WGAND significantly outperforms existing methods in detecting anomalous nodes within weighted PPI networks, as evidenced by superior AUC, PR-AUC, and Precision at K (P@K) metrics.

- **Graphics and Visualization:** To improve the visual presentation of our manuscript, we edited the figures throughout the manuscript. Moreover, we hired a professional graphic designer to create an infographic that illustrates our algorithm (see Figure 1 in the revised manuscript). We believe these revisions will make our findings more accessible and compelling to a broad audience.

- **Response to Reviewers:** We have prepared a detailed response to each reviewer's comments, outlining the changes made to address their concerns. We included the response letter with our resubmission.

For ease of review, we highlighted all modifications in the revised manuscript in blue. These changes have significantly strengthened the manuscript, addressing all concerns raised during the initial review process.

Thank you for considering our revised manuscript. We look forward to your feedback and are happy to provide any additional information that may be required.

Sincerely,,
Dima Kagan, Juman Jubran, Esti Yeger-Lotem, and Michael Fire

Infographic Click here to access/download;Graphical Abstract;infographic.jpg

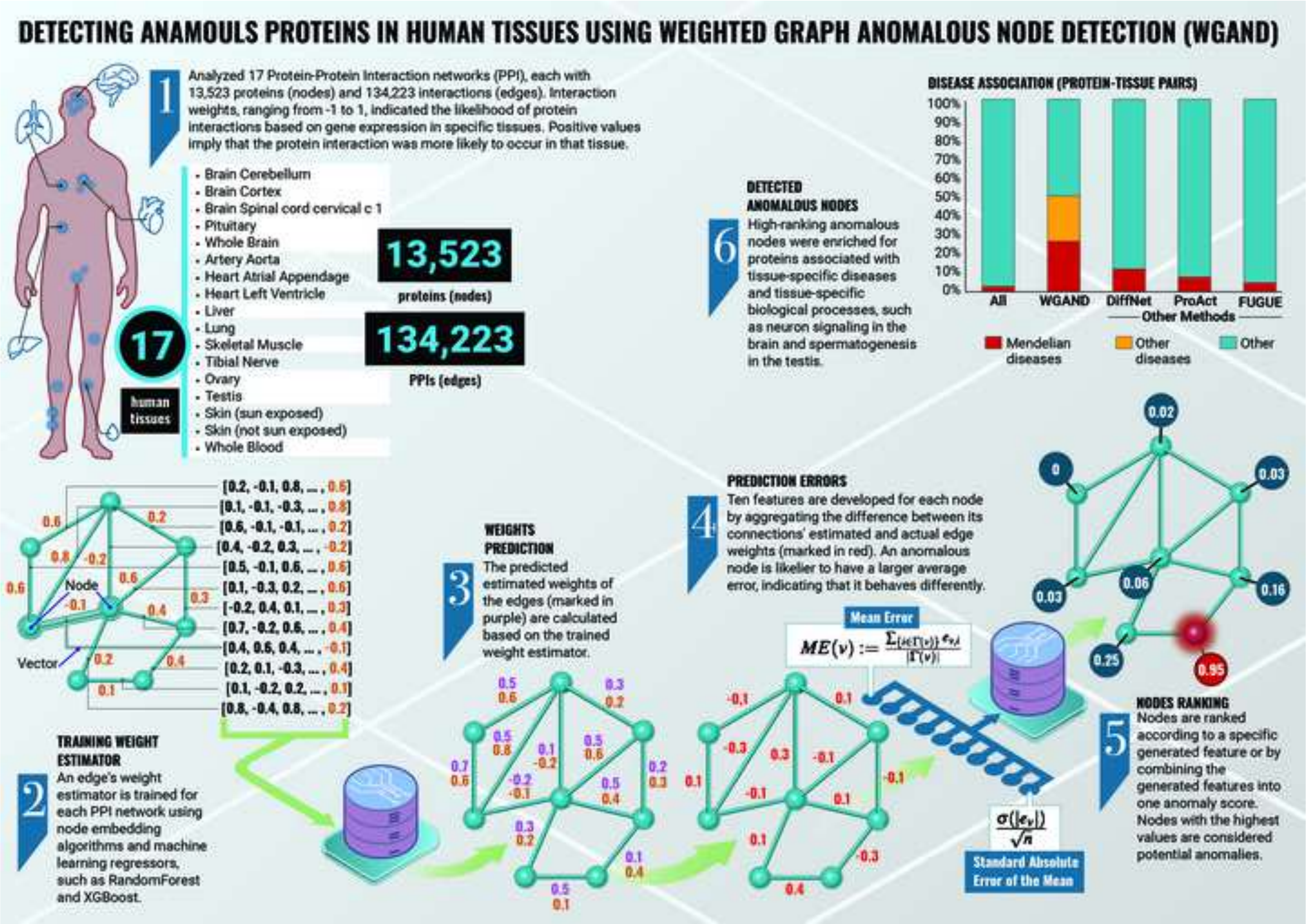

Supplement: giaf034_GIGA-D-24-00363_Original_Submission [file giaf034_giga-d-24-00363_original_submission.pdf]
