# Supplementary material for: Network-based anomaly detection algorithm reveals proteins with major roles in human tissues
Source: Gigascience. 2025 Apr 8;14:giaf034. doi: 10.1093/gigascience/giaf034 (PMC11976396; doi:10.1093/gigascience/giaf034)
Supplement: giaf034_GIGA-D-24-00363_Revision_2 [file giaf034_giga-d-24-00363_revision_2.pdf]

GigaScience

Network-based anomaly detection algorithm reveals proteins with major roles in human tissues

--Manuscript Draft--

**Manuscript Number:**	GIGA-D-24-00363R2
**Full Title:**	Network-based anomaly detection algorithm reveals proteins with major roles in human tissues
**Article Type:**	Research
**Funding Information:**	Council for Higher Education — Prof. Esti Yeger-Lotem, Dr. Michael Fire<br>Israel Science Foundation (401/22) — Prof. Esti Yeger-Lotem
**Abstract:**	Background<br>Proteins act through physical interactions with other molecules to maintain organismal health. Protein-protein interaction (PPI) networks proved to be a powerful framework for obtaining insight into protein functions, cellular organization, response to signals, and disease states. In multicellular organisms, protein content varies between tissues, influencing tissue morphology and function. Weighted PPI networks, reflecting the likelihood of interactions in specific tissues, offer insights into tissue-specific processes and disease mechanisms. We hypothesized that detecting anomalous nodes in these networks could reveal proteins with key tissue-specific functions.<br><br>Results<br>Here, we introduce Weighted Graph Anomalous Node Detection (WGAND), a novel machine-learning algorithm to identify anomalous nodes in weighted graphs. WGAND estimates expected edge weights and uses deviations to generate anomaly detection features, which are then used to score network nodes. We applied WGAND to weighted PPI networks of 17 human tissues. High-ranking anomalous nodes were enriched for proteins associated with tissue-specific diseases and tissue-specific biological processes, such as neuron signaling in the brain and spermatogenesis in the testis. WGAND outperformed other methods in terms of Area Under the ROC Curve (AUC) and Precision at K (P@K), highlighting its effectiveness in uncovering biologically meaningful anomalies.<br><br>Conclusion<br>Our findings demonstrate WGAND's potential as a powerful tool for detecting anomalous proteins with significant biological roles. By identifying proteins involved in critical tissue-specific processes and diseases, WGAND offers valuable insights for discovering novel biomarkers and therapeutic targets. Its versatile algorithm is suitable for any weighted graph and is broadly applicable across various fields. The WGAND algorithm is available as an open-source Python library at https://github.com/data4goodlab/wgand.
**Corresponding Author:**	Michael Fire, Ph.D<br>Ben-Gurion University of the Negev<br>Beer-Sheva, Israel ISRAEL
**Corresponding Author Secondary Information:**	
**Corresponding Author's Institution:**	Ben-Gurion University of the Negev
**Corresponding Author's Secondary Institution:**	
**First Author:**	Dima Kagan
**First Author Secondary Information:**	
**Order of Authors:**	Dima Kagan

Powered by Editorial Manager® and ProduXion Manager® from Aries Systems Corporation

	Juman Jubran
	Esti Yeger-Lotem
	Michael Fire, Ph.D
**Order of Authors Secondary Information:**	
**Response to Reviewers:**	See attached letter
**Additional Information:**	
**Question**	**Response**
Are you submitting this manuscript to a special series or article collection?	No
**Experimental design and statistics**<br><br>Full details of the experimental design and statistical methods used should be given in the Methods section, as detailed in our Minimum Standards Reporting Checklist. Information essential to interpreting the data presented should be made available in the figure legends.<br><br>Have you included all the information requested in your manuscript?	Yes
**Resources**<br><br>A description of all resources used, including antibodies, cell lines, animals and software tools, with enough information to allow them to be uniquely identified, should be included in the Methods section. Authors are strongly encouraged to cite Research Resource Identifiers (RRIDs) for antibodies, model organisms and tools, where possible.<br><br>Have you included the information requested as detailed in our Minimum Standards Reporting Checklist?	Yes
**Availability of data and materials**<br><br>All datasets and code on which the conclusions of the paper rely must be either included in your submission or	Yes

deposited in publicly available repositories (where available and ethically appropriate), referencing such data using a unique identifier in the references and in the "Availability of Data and Materials" section of your manuscript.<br><br>Have you have met the above requirement as detailed in our Minimum Standards Reporting Checklist?	

tex

Click here to access/download;LaTeX - Main Document (TeX file);article_3.tex

```
This is pdfTeX, Version 3.141592653-2.6-1.40.26 (TeX Live 2024)
(preloaded format=pdflatex 2024.8.2)  5 MAR 2025 00:05
entering extended mode
 restricted \write18 enabled.
 %&-line parsing enabled.
**article_3.tex
(./article_3.tex
LaTeX2e <2024-06-01> patch level 2
L3 programming layer <2024-05-27>
(./SelfArx.cls
Document Class: SelfArx 25/01/2012, v1.0
(c:/texlive/2024/texmf-dist/tex/latex/base/ifthen.sty
Package: ifthen 2024/03/16 v1.1e Standard LaTeX ifthen package (DPC)
) (c:/texlive/2024/texmf-dist/tex/latex/tools/calc.sty
Package: calc 2023/07/08 v4.3 Infix arithmetic (KKT,FJ)
\calc@Acount=\count194
\calc@Bcount=\count195
\calc@Adimen=\dimen141
\calc@Bdimen=\dimen142
\calc@Askip=\skip49
\calc@Bskip=\skip50
LaTeX Info: Redefining \setlength on input line 80.
LaTeX Info: Redefining \addtolength on input line 81.
\calc@Ccount=\count196
\calc@Cskip=\skip51
) (c:/texlive/2024/texmf-dist/tex/latex/base/article.cls
Document Class: article 2024/02/08 v1.4n Standard LaTeX document class
(c:/texlive/2024/texmf-dist/tex/latex/base/fleqn.clo
File: fleqn.clo 2016/12/29 v1.2b Standard LaTeX option (flush left
equations)
\mathindent=\skip52
Applying: [2015/01/01] Make \[ robust on input line 50.
LaTeX Info: Redefining \[ on input line 51.
Already applied: [0000/00/00] Make \[ robust on input line 62.
Applying: [2015/01/01] Make \] robust on input line 74.
LaTeX Info: Redefining \] on input line 75.
Already applied: [0000/00/00] Make \] robust on input line 83.
) (c:/texlive/2024/texmf-dist/tex/latex/base/size10.clo
File: size10.clo 2024/02/08 v1.4n Standard LaTeX file (size option)
)
\c@part=\count197
\c@section=\count198
\c@subsection=\count199
\c@subsubsection=\count266
\c@paragraph=\count267
\c@subparagraph=\count268
\c@figure=\count269
\c@table=\count270
\abovecaptionskip=\skip53
\belowcaptionskip=\skip54
\bibindent=\dimen143
) (c:/texlive/2024/texmf-dist/tex/generic/iftex/ifpdf.sty
Package: ifpdf 2019/10/25 v3.4 ifpdf legacy package. Use iftex instead.
(c:/texlive/2024/texmf-dist/tex/generic/iftex/iftex.sty
```

```
Package: iftex 2022/02/03 v1.0f TeX engine tests
)) (c:/texlive/2024/texmf-dist/tex/latex/psnfss/times.sty
Package: times 2020/03/25 PSNFSS-v9.3 (SPQR)
) (c:/texlive/2024/texmf-dist/tex/latex/psnfss/mathptmx.sty
Package: mathptmx 2020/03/25 PSNFSS-v9.3 Times w/ Math, improved (SPQR,
WaS)
LaTeX Font Info:    Redeclaring symbol font `operators' on input line 28.
LaTeX Font Info:    Overwriting symbol font `operators' in version
`normal'
(Font)                  OT1/cmr/m/n --> OT1/ztmcm/m/n on input line 28.
LaTeX Font Info:    Overwriting symbol font `operators' in version `bold'
(Font)                  OT1/cmr/bx/n --> OT1/ztmcm/m/n on input line 28.
LaTeX Font Info:    Redeclaring symbol font `letters' on input line 29.
LaTeX Font Info:    Overwriting symbol font `letters' in version `normal'
(Font)                  OML/cmm/m/it --> OML/ztmcm/m/it on input line 29.
LaTeX Font Info:    Overwriting symbol font `letters' in version `bold'
(Font)                  OML/cmm/b/it --> OML/ztmcm/m/it on input line 29.
LaTeX Font Info:    Redeclaring symbol font `symbols' on input line 30.
LaTeX Font Info:    Overwriting symbol font `symbols' in version `normal'
(Font)                  OMS/cmsy/m/n --> OMS/ztmcm/m/n on input line 30.
LaTeX Font Info:    Overwriting symbol font `symbols' in version `bold'
(Font)                  OMS/cmsy/b/n --> OMS/ztmcm/m/n on input line 30.
LaTeX Font Info:    Redeclaring symbol font `largesymbols' on input line
31.
LaTeX Font Info:    Overwriting symbol font `largesymbols' in version
`normal'
(Font)                  OMX/cmex/m/n --> OMX/ztmcm/m/n on input line 31.
LaTeX Font Info:    Overwriting symbol font `largesymbols' in version
`bold'
(Font)                  OMX/cmex/m/n --> OMX/ztmcm/m/n on input line 31.
\symbold=\mathgroup4
\symitalic=\mathgroup5
LaTeX Font Info:    Redeclaring math alphabet \mathbf on input line 34.
LaTeX Font Info:    Overwriting math alphabet `\mathbf' in version
`normal'
(Font)                  OT1/cmr/bx/n --> OT1/ptm/bx/n on input line 34.
LaTeX Font Info:    Overwriting math alphabet `\mathbf' in version `bold'
(Font)                  OT1/cmr/bx/n --> OT1/ptm/bx/n on input line 34.
LaTeX Font Info:    Redeclaring math alphabet \mathit on input line 35.
LaTeX Font Info:    Overwriting math alphabet `\mathit' in version
`normal'
(Font)                  OT1/cmr/m/it --> OT1/ptm/m/it on input line 35.
LaTeX Font Info:    Overwriting math alphabet `\mathit' in version `bold'
(Font)                  OT1/cmr/bx/it --> OT1/ptm/m/it on input line 35.
LaTeX Info: Redefining \hbar on input line 50.
) (c:/texlive/2024/texmf-dist/tex/latex/base/inputenc.sty
Package: inputenc 2024/02/08 v1.3d Input encoding file
\inpenc@prehook=\toks17
\inpenc@posthook=\toks18
) (c:/texlive/2024/texmf-dist/tex/latex/amsmath/amsmath.sty
Package: amsmath 2024/05/23 v2.17q AMS math features
\@mathmargin=\skip55
For additional information on amsmath, use the `?' option.
(c:/texlive/2024/texmf-dist/tex/latex/amsmath/amstext.sty
```

```
Package: amstext 2021/08/26 v2.01 AMS text
(c:/texlive/2024/texmf-dist/tex/latex/amsmath/amsgen.sty
File: amsgen.sty 1999/11/30 v2.0 generic functions
\@emptytoks=\toks19
\ex@=\dimen144
)) (c:/texlive/2024/texmf-dist/tex/latex/amsmath/amsbsy.sty
Package: amsbsy 1999/11/29 v1.2d Bold Symbols
\pmbraise@=\dimen145
) (c:/texlive/2024/texmf-dist/tex/latex/amsmath/amsopn.sty
Package: amsopn 2022/04/08 v2.04 operator names
)
\inf@bad=\count271
LaTeX Info: Redefining \frac on input line 233.
\uproot@=\count272
\leftroot@=\count273
LaTeX Info: Redefining \overline on input line 398.
LaTeX Info: Redefining \colon on input line 409.
\classnum@=\count274
\DOTSCASE@=\count275
LaTeX Info: Redefining \ldots on input line 495.
LaTeX Info: Redefining \dots on input line 498.
LaTeX Info: Redefining \cdots on input line 619.
\Mathstrutbox@=\box52
\strutbox@=\box53
LaTeX Info: Redefining \big on input line 721.
LaTeX Info: Redefining \Big on input line 722.
LaTeX Info: Redefining \bigg on input line 723.
LaTeX Info: Redefining \Bigg on input line 724.
\big@size=\dimen146
LaTeX Font Info:    Redeclaring font encoding OML on input line 742.
LaTeX Font Info:    Redeclaring font encoding OMS on input line 743.
\macc@depth=\count276
LaTeX Info: Redefining \bmod on input line 904.
LaTeX Info: Redefining \pmod on input line 909.
LaTeX Info: Redefining \smash on input line 939.
LaTeX Info: Redefining \relbar on input line 969.
LaTeX Info: Redefining \Relbar on input line 970.
\c@MaxMatrixCols=\count277
\dotsspace@=\muskip17
\c@parentequation=\count278
\dspbrk@lvl=\count279
\tag@help=\toks20
\row@=\count280
\column@=\count281
\maxfields@=\count282
\andhelp@=\toks21
\eqnshift@=\dimen147
\alignsep@=\dimen148
\tagshift@=\dimen149
\tagwidth@=\dimen150
\totwidth@=\dimen151
\lineht@=\dimen152
\@envbody=\toks22
\multlinegap=\skip56
```

```
\multlinetaggap=\skip57
\mathdisplay@stack=\toks23
LaTeX Info: Redefining \[ on input line 2953.
LaTeX Info: Redefining \] on input line 2954.
) (c:/texlive/2024/texmf-dist/tex/latex/amsfonts/amsfonts.sty
Package: amsfonts 2013/01/14 v3.01 Basic AMSFonts support
\symAMSa=\mathgroup6
\symAMSb=\mathgroup7
LaTeX Font Info:    Overwriting math alphabet `\mathfrak' in version
`bold'
(Font)                  U/euf/m/n --> U/euf/b/n on input line 106.
) (c:/texlive/2024/texmf-dist/tex/latex/amsfonts/amssymb.sty
Package: amssymb 2013/01/14 v3.01 AMS font symbols
) (c:/texlive/2024/texmf-dist/tex/latex/graphics/graphicx.sty
Package: graphicx 2021/09/16 v1.2d Enhanced LaTeX Graphics (DPC,SPQR)
(c:/texlive/2024/texmf-dist/tex/latex/graphics/keyval.sty
Package: keyval 2022/05/29 v1.15 key=value parser (DPC)
\KV@toks@=\toks24
) (c:/texlive/2024/texmf-dist/tex/latex/graphics/graphics.sty
Package: graphics 2024/05/23 v1.4g Standard LaTeX Graphics (DPC,SPQR)
(c:/texlive/2024/texmf-dist/tex/latex/graphics/trig.sty
Package: trig 2023/12/02 v1.11 sin cos tan (DPC)
) (c:/texlive/2024/texmf-dist/tex/latex/graphics-cfg/graphics.cfg
File: graphics.cfg 2016/06/04 v1.11 sample graphics configuration
)
Package graphics Info: Driver file: pdftex.def on input line 106.
(c:/texlive/2024/texmf-dist/tex/latex/graphics-def/pdftex.def
File: pdftex.def 2024/04/13 v1.2c Graphics/color driver for pdftex
))
\Gin@req@height=\dimen153
\Gin@req@width=\dimen154
) (c:/texlive/2024/texmf-dist/tex/latex/xcolor/xcolor.sty
Package: xcolor 2023/11/15 v3.01 LaTeX color extensions (UK)
(c:/texlive/2024/texmf-dist/tex/latex/graphics-cfg/color.cfg
File: color.cfg 2016/01/02 v1.6 sample color configuration
)
Package xcolor Info: Driver file: pdftex.def on input line 274.
(c:/texlive/2024/texmf-dist/tex/latex/graphics/mathcolor.ltx)
Package xcolor Info: Model `cmy' substituted by `cmy0' on input line
1350.
Package xcolor Info: Model `hsb' substituted by `rgb' on input line 1354.
Package xcolor Info: Model `RGB' extended on input line 1366.
Package xcolor Info: Model `HTML' substituted by `rgb' on input line
1368.
Package xcolor Info: Model `Hsb' substituted by `hsb' on input line 1369.
Package xcolor Info: Model `tHsb' substituted by `hsb' on input line
1370.
Package xcolor Info: Model `HSB' substituted by `hsb' on input line 1371.
Package xcolor Info: Model `Gray' substituted by `gray' on input line
1372.
Package xcolor Info: Model `wave' substituted by `hsb' on input line
1373.
) (c:/texlive/2024/texmf-dist/tex/generic/babel/babel.sty
Package: babel 2024/06/26 v24.7 The Babel package
```

```
\babel@savecnt=\count283
\U@D=\dimen155
\l@unhyphenated=\language89
(c:/texlive/2024/texmf-dist/tex/generic/babel/txtbabel.def)
\bbl@readstream=\read2
\bbl@dirlevel=\count284
(c:/texlive/2024/texmf-dist/tex/generic/babel-english/english.ldf
Language: english 2017/06/06 v3.3r English support from the babel system
Package babel Info: Hyphen rules for 'canadian' set to \l@english
(babel)             (\language0). Reported on input line 102.
Package babel Info: Hyphen rules for 'australian' set to \l@ukenglish
(babel)             (\language23). Reported on input line 105.
Package babel Info: Hyphen rules for 'newzealand' set to \l@ukenglish
(babel)             (\language23). Reported on input line 108.
)) (c:/texlive/2024/texmf-dist/tex/generic/babel/locale/en/babel-
english.tex
Package babel Info: Importing font and identification data for english
(babel)             from babel-en.ini. Reported on input line 11.
) (c:/texlive/2024/texmf-dist/tex/latex/booktabs/booktabs.sty
Package: booktabs 2020/01/12 v1.61803398 Publication quality tables
\heavyrulewidth=\dimen156
\lightrulewidth=\dimen157
\cmidrulewidth=\dimen158
\belowrulesep=\dimen159
\belowbottomsep=\dimen160
\aboverulesep=\dimen161
\abovetopsep=\dimen162
\cmidrulesep=\dimen163
\cmidrulekern=\dimen164
\defaultaddspace=\dimen165
\@cmidla=\count285
\@cmidlb=\count286
\@aboverulesep=\dimen166
\@belowrulesep=\dimen167
\@thisruleclass=\count287
\@lastruleclass=\count288
\@thisrulewidth=\dimen168
) (c:/texlive/2024/texmf-dist/tex/latex/geometry/geometry.sty
Package: geometry 2020/01/02 v5.9 Page Geometry
(c:/texlive/2024/texmf-dist/tex/generic/iftex/ifvtex.sty
Package: ifvtex 2019/10/25 v1.7 ifvtex legacy package. Use iftex instead.
)
\Gm@cnth=\count289
\Gm@cntv=\count290
\c@Gm@tempcnt=\count291
\Gm@bindingoffset=\dimen169
\Gm@wd@mp=\dimen170
\Gm@odd@mp=\dimen171
\Gm@even@mp=\dimen172
\Gm@layoutwidth=\dimen173
\Gm@layoutheight=\dimen174
\Gm@layouthoffset=\dimen175
\Gm@layoutvoffset=\dimen176
\Gm@dimlist=\toks25
```

```
) (c:/texlive/2024/texmf-dist/tex/latex/caption/caption.sty
Package: caption 2023/08/05 v3.6o Customizing captions (AR)
(c:/texlive/2024/texmf-dist/tex/latex/caption/caption3.sty
Package: caption3 2023/07/31 v2.4d caption3 kernel (AR)
\caption@tempdima=\dimen177
\captionmargin=\dimen178
\caption@leftmargin=\dimen179
\caption@rightmargin=\dimen180
\caption@width=\dimen181
\caption@indent=\dimen182
\caption@parindent=\dimen183
\caption@hangindent=\dimen184
Package caption Info: Standard document class detected.
)
\c@caption@flags=\count292
\c@continuedfloat=\count293
) (c:/texlive/2024/texmf-dist/tex/latex/fancyhdr/fancyhdr.sty
Package: fancyhdr 2024/07/23 v4.3.1 Extensive control of page headers and
foote
rs
\f@nch@headwidth=\skip58
\f@nch@O@elh=\skip59
\f@nch@O@erh=\skip60
\f@nch@O@olh=\skip61
\f@nch@O@orh=\skip62
\f@nch@O@elf=\skip63
\f@nch@O@erf=\skip64
\f@nch@O@olf=\skip65
\f@nch@O@orf=\skip66
) (c:/texlive/2024/texmf-dist/tex/latex/lastpage/lastpage.sty
Package: lastpage 2024/07/07 v2.1c lastpage: 2.09 or 2e? (HMM)
(c:/texlive/2024/texmf-dist/tex/latex/lastpage/lastpage2e.sty
Package: lastpage2e 2024/07/07 v2.1c Decide which 2e lastpage version to
use (H
MM)
(c:/texlive/2024/texmf-dist/tex/latex/lastpage/lastpagemodern.sty
Package: lastpagemodern 2024-07-07 v2.1c Refers to last page's name (HMM;
JPG)
\c@lastpagecount=\count294
)
)) (c:/texlive/2024/texmf-dist/tex/latex/titlesec/titlesec.sty
Package: titlesec 2023/10/27 v2.16 Sectioning titles
\ttl@box=\box54
\beforetitleunit=\skip67
\aftertitleunit=\skip68
\ttl@plus=\dimen185
\ttl@minus=\dimen186
\ttl@toksa=\toks26
\titlewidth=\dimen187
\titlewidthlast=\dimen188
\titlewidthfirst=\dimen189
)
\tocsep=\skip69
(c:/texlive/2024/texmf-dist/tex/latex/titlesec/titletoc.sty
```

```
Package: titletoc 2023/10/27 v2.16 TOC entries
\ttl@leftsep=\dimen190
)
\@authcnt=\count295
\@tmpcnt=\count296
(c:/texlive/2024/texmf-dist/tex/latex/enumitem/enumitem.sty
Package: enumitem 2019/06/20 v3.9 Customized lists
\labelindent=\skip70
\enit@outerparindent=\dimen191
\enit@toks=\toks27
\enit@inbox=\box55
\enit@count@id=\count297
\enitdp@description=\count298
)) (c:/texlive/2024/texmf-dist/tex/latex/microtype/microtype.sty
Package: microtype 2024/03/29 v3.1b Micro-typographical refinements (RS)
(c:/texlive/2024/texmf-dist/tex/latex/etoolbox/etoolbox.sty
Package: etoolbox 2020/10/05 v2.5k e-TeX tools for LaTeX (JAW)
\etb@tempcnta=\count299
)
\MT@toks=\toks28
\MT@tempbox=\box56
\MT@count=\count300
LaTeX Info: Redefining \noprotrusionifhmode on input line 1061.
LaTeX Info: Redefining \leftprotrusion on input line 1062.
\MT@prot@toks=\toks29
LaTeX Info: Redefining \rightprotrusion on input line 1081.
LaTeX Info: Redefining \textls on input line 1392.
\MT@outer@kern=\dimen192
LaTeX Info: Redefining \textmicrotypecontext on input line 2013.
\MT@listname@count=\count301
(c:/texlive/2024/texmf-dist/tex/latex/microtype/microtype-pdftex.def
File: microtype-pdftex.def 2024/03/29 v3.1b Definitions specific to
pdftex (RS)

LaTeX Info: Redefining \lsstyle on input line 902.
LaTeX Info: Redefining \lslig on input line 902.
\MT@outer@space=\skip71
)
Package microtype Info: Loading configuration file microtype.cfg.
(c:/texlive/2024/texmf-dist/tex/latex/microtype/microtype.cfg
File: microtype.cfg 2024/03/29 v3.1b microtype main configuration file
(RS)
)) (c:/texlive/2024/texmf-dist/tex/latex/natbib/natbib.sty
Package: natbib 2010/09/13 8.31b (PWD, AO)
\bibhang=\skip72
\bibsep=\skip73
LaTeX Info: Redefining \cite on input line 694.
\c@NAT@ctr=\count302
) (c:/texlive/2024/texmf-dist/tex/latex/caption/subcaption.sty
Package: subcaption 2023/07/28 v1.6b Sub-captions (AR)
Package caption Info: New subtype `subfigure' on input line 238.
\c@subfigure=\count303
Package caption Info: New subtype `subtable' on input line 238.
\c@subtable=\count304
```

```
) (c:/texlive/2024/texmf-dist/tex/latex/pdflscape/pdflscape.sty
Package: pdflscape 2022-10-27 v0.13 Display of landscape pages in PDF
(c:/texlive/2024/texmf-dist/tex/latex/pdflscape/pdflscape-nometadata.sty
Package: pdflscape-nometadata 2022-10-28 v0.13 Display of landscape pages
in PD
F (HO)
(c:/texlive/2024/texmf-dist/tex/latex/graphics/lscape.sty
Package: lscape 2020/05/28 v3.02 Landscape Pages (DPC)
)
Package pdflscape Info: Auto-detected driver: pdftex on input line 81.
)) (c:/texlive/2024/texmf-dist/tex/latex/multirow/multirow.sty
Package: multirow 2021/03/15 v2.8 Span multiple rows of a table
\multirow@colwidth=\skip74
\multirow@cntb=\count305
\multirow@dima=\skip75
\bigstrutjot=\dimen193
) (c:/texlive/2024/texmf-dist/tex/latex/tools/longtable.sty
Package: longtable 2024-04-26 v4.20 Multi-page Table package (DPC)
\LTleft=\skip76
\LTright=\skip77
\LTpre=\skip78
\LTpost=\skip79
\LTchunksize=\count306
\LTcapwidth=\dimen194
\LT@head=\box57
\LT@firsthead=\box58
\LT@foot=\box59
\LT@lastfoot=\box60
\LT@gbox=\box61
\LT@cols=\count307
\LT@rows=\count308
\c@LT@tables=\count309
\c@LT@chunks=\count310
\LT@p@ftn=\toks30
) (c:/texlive/2024/texmf-dist/tex/latex/tabu/tabu.sty
Package: tabu 2019/01/11 v2.9 - flexible LaTeX tabulars (FC+tabu-fixed)
(c:/texlive/2024/texmf-dist/tex/latex/tools/array.sty
Package: array 2024/06/14 v2.6d Tabular extension package (FMi)
\col@sep=\dimen195
\ar@mcellbox=\box62
\extrarowheight=\dimen196
\NC@list=\toks31
\extratabsurround=\skip80
\backup@length=\skip81
\ar@cellbox=\box63
) (c:/texlive/2024/texmf-dist/tex/latex/varwidth/varwidth.sty
Package: varwidth 2009/03/30 ver 0.92;  Variable-width minipages
\@vwid@box=\box64
\sift@deathcycles=\count311
\@vwid@loff=\dimen197
\@vwid@roff=\dimen198
)
\c@taburow=\count312
\tabu@nbcols=\count313
```

```
\tabu@cnt=\count314
\tabu@Xcol=\count315
\tabu@alloc=\count316
\tabu@nested=\count317
\tabu@target=\dimen199
\tabu@spreadtarget=\dimen256
\tabu@naturalX=\dimen257
\tabucolX=\dimen258
\tabu@Xsum=\dimen259
\extrarowdepth=\dimen260
\abovetabulinesep=\dimen261
\belowtabulinesep=\dimen262
\tabustrutrule=\dimen263
\tabu@thebody=\toks32
\tabu@footnotes=\toks33
\tabu@box=\box65
\tabu@arstrutbox=\box66
\tabu@hleads=\box67
\tabu@vleads=\box68
\tabu@cellskip=\skip82
) (c:/texlive/2024/texmf-dist/tex/latex/float/float.sty
Package: float 2001/11/08 v1.3d Float enhancements (AL)
\c@float@type=\count318
\float@exts=\toks34
\float@box=\box69
\@float@everytoks=\toks35
\@floatcapt=\box70
) (c:/texlive/2024/texmf-dist/tex/latex/graphics/rotating.sty
Package: rotating 2016/08/11 v2.16d rotated objects in LaTeX
\c@r@tfl@t=\count319
\rotFPtop=\skip83
\rotFPbot=\skip84
\rot@float@box=\box71
\rot@mess@toks=\toks36
) (c:/texlive/2024/texmf-dist/tex/latex/hyperref/hyperref.sty
Package: hyperref 2024-07-10 v7.01j Hypertext links for LaTeX
(c:/texlive/2024/texmf-dist/tex/latex/kvsetkeys/kvsetkeys.sty
Package: kvsetkeys 2022-10-05 v1.19 Key value parser (HO)
) (c:/texlive/2024/texmf-dist/tex/generic/kvdefinekeys/kvdefinekeys.sty
Package: kvdefinekeys 2019-12-19 v1.6 Define keys (HO)
) (c:/texlive/2024/texmf-dist/tex/generic/pdfescape/pdfescape.sty
Package: pdfescape 2019/12/09 v1.15 Implements pdfTeX's escape features
(HO)
(c:/texlive/2024/texmf-dist/tex/generic/ltxcmds/ltxcmds.sty
Package: ltxcmds 2023-12-04 v1.26 LaTeX kernel commands for general use
(HO)
) (c:/texlive/2024/texmf-dist/tex/generic/pdftexcmds/pdftexcmds.sty
Package: pdftexcmds 2020-06-27 v0.33 Utility functions of pdfTeX for
LuaTeX (HO
)
(c:/texlive/2024/texmf-dist/tex/generic/infwarerr/infwarerr.sty
Package: infwarerr 2019/12/03 v1.5 Providing info/warning/error messages
(HO)
)
```

```
Package pdftexcmds Info: \pdf@primitive is available.
Package pdftexcmds Info: \pdf@ifprimitive is available.
Package pdftexcmds Info: \pdfdraftmode found.
)) (c:/texlive/2024/texmf-dist/tex/latex/hycolor/hycolor.sty
Package: hycolor 2020-01-27 v1.10 Color options for hyperref/bookmark
(HO)
) (c:/texlive/2024/texmf-dist/tex/latex/hyperref/nameref.sty
Package: nameref 2023-11-26 v2.56 Cross-referencing by name of section
(c:/texlive/2024/texmf-dist/tex/latex/refcount/refcount.sty
Package: refcount 2019/12/15 v3.6 Data extraction from label references
(HO)
) (c:/texlive/2024/texmf-
dist/tex/generic/gettitlestring/gettitlestring.sty
Package: gettitlestring 2019/12/15 v1.6 Cleanup title references (HO)
(c:/texlive/2024/texmf-dist/tex/latex/kvoptions/kvoptions.sty
Package: kvoptions 2022-06-15 v3.15 Key value format for package options
(HO)
))
\c@section@level=\count320
) (c:/texlive/2024/texmf-dist/tex/generic/stringenc/stringenc.sty
Package: stringenc 2019/11/29 v1.12 Convert strings between diff.
encodings (HO
)
)
\@linkdim=\dimen264
\Hy@linkcounter=\count321
\Hy@pagecounter=\count322
(c:/texlive/2024/texmf-dist/tex/latex/hyperref/pd1enc.def
File: pd1enc.def 2024-07-10 v7.01j Hyperref: PDFDocEncoding definition
(HO)
Now handling font encoding PD1 ...
... no UTF-8 mapping file for font encoding PD1
) (c:/texlive/2024/texmf-dist/tex/generic/intcalc/intcalc.sty
Package: intcalc 2019/12/15 v1.3 Expandable calculations with integers
(HO)
)
\Hy@SavedSpaceFactor=\count323
(c:/texlive/2024/texmf-dist/tex/latex/hyperref/puenc.def
File: puenc.def 2024-07-10 v7.01j Hyperref: PDF Unicode definition (HO)
Now handling font encoding PU ...
... no UTF-8 mapping file for font encoding PU
)
Package hyperref Info: Hyper figures OFF on input line 4157.
Package hyperref Info: Link nesting OFF on input line 4162.
Package hyperref Info: Hyper index ON on input line 4165.
Package hyperref Info: Plain pages OFF on input line 4172.
Package hyperref Info: Backreferencing OFF on input line 4177.
Package hyperref Info: Implicit mode ON; LaTeX internals redefined.
Package hyperref Info: Bookmarks ON on input line 4424.
\c@Hy@tempcnt=\count324
(c:/texlive/2024/texmf-dist/tex/latex/url/url.sty
\Urlmuskip=\muskip18
Package: url 2013/09/16  ver 3.4  Verb mode for urls, etc.
)
```

```
LaTeX Info: Redefining \url on input line 4763.
\XeTeXLinkMargin=\dimen265
(c:/texlive/2024/texmf-dist/tex/generic/bitset/bitset.sty
Package: bitset 2019/12/09 v1.3 Handle bit-vector datatype (HO)
(c:/texlive/2024/texmf-dist/tex/generic/bigintcalc/bigintcalc.sty
Package: bigintcalc 2019/12/15 v1.5 Expandable calculations on big
integers (HO
)
))
\Fld@menulength=\count325
\Field@Width=\dimen266
\Fld@charsize=\dimen267
Package hyperref Info: Hyper figures OFF on input line 6042.
Package hyperref Info: Link nesting OFF on input line 6047.
Package hyperref Info: Hyper index ON on input line 6050.
Package hyperref Info: backreferencing OFF on input line 6057.
Package hyperref Info: Link coloring OFF on input line 6062.
Package hyperref Info: Link coloring with OCG OFF on input line 6067.
Package hyperref Info: PDF/A mode OFF on input line 6072.
(c:/texlive/2024/texmf-dist/tex/latex/base/atbegshi-ltx.sty
Package: atbegshi-ltx 2021/01/10 v1.0c Emulation of the original atbegshi
package with kernel methods
)
\Hy@abspage=\count326
\c@Item=\count327
\c@Hfootnote=\count328
)
Package hyperref Info: Driver (autodetected): hpdftex.
(c:/texlive/2024/texmf-dist/tex/latex/hyperref/hpdftex.def
File: hpdftex.def 2024-07-10 v7.01j Hyperref driver for pdfTeX
(c:/texlive/2024/texmf-dist/tex/latex/base/atveryend-ltx.sty
Package: atveryend-ltx 2020/08/19 v1.0a Emulation of the original
atveryend pac
kage
with kernel methods
)
\Fld@listcount=\count329
\c@bookmark@seq@number=\count330
(c:/texlive/2024/texmf-dist/tex/latex/rerunfilecheck/rerunfilecheck.sty
Package: rerunfilecheck 2022-07-10 v1.10 Rerun checks for auxiliary files
(HO)
(c:/texlive/2024/texmf-dist/tex/generic/uniquecounter/uniquecounter.sty
Package: uniquecounter 2019/12/15 v1.4 Provide unlimited unique counter
(HO)
)
Package uniquecounter Info: New unique counter `rerunfilecheck' on input
line 2
85.
)
\Hy@SectionHShift=\skip85
)
Package hyperref Info: Option `colorlinks' set `true' on input line 48.
Package hyperref Info: Option `breaklinks' set `true' on input line 48.
```

```
Package hyperref Info: Option `bookmarksopen' set `false' on input line
48.
LaTeX Font Info:    Trying to load font information for OT1+ptm on input
line 1
01.
(c:/texlive/2024/texmf-dist/tex/latex/psnfss/ot1ptm.fd
File: ot1ptm.fd 2001/06/04 font definitions for OT1/ptm.
) (c:/texlive/2024/texmf-dist/tex/latex/l3backend/l3backend-pdftex.def
File: l3backend-pdftex.def 2024-05-08 L3 backend support: PDF output
(pdfTeX)
\l__color_backend_stack_int=\count331
\l__pdf_internal_box=\box72
) (./article_3.aux)
\openout1 = `article_3.aux'.

LaTeX Font Info:    Checking defaults for OML/cmm/m/it on input line 101.
LaTeX Font Info:    ... okay on input line 101.
LaTeX Font Info:    Checking defaults for OMS/cmsy/m/n on input line 101.
LaTeX Font Info:    ... okay on input line 101.
LaTeX Font Info:    Checking defaults for OT1/cmr/m/n on input line 101.
LaTeX Font Info:    ... okay on input line 101.
LaTeX Font Info:    Checking defaults for T1/cmr/m/n on input line 101.
LaTeX Font Info:    ... okay on input line 101.
LaTeX Font Info:    Checking defaults for TS1/cmr/m/n on input line 101.
LaTeX Font Info:    ... okay on input line 101.
LaTeX Font Info:    Checking defaults for OMX/cmex/m/n on input line 101.
LaTeX Font Info:    ... okay on input line 101.
LaTeX Font Info:    Checking defaults for U/cmr/m/n on input line 101.
LaTeX Font Info:    ... okay on input line 101.
LaTeX Font Info:    Checking defaults for PD1/pdf/m/n on input line 101.
LaTeX Font Info:    ... okay on input line 101.
LaTeX Font Info:    Checking defaults for PU/pdf/m/n on input line 101.
LaTeX Font Info:    ... okay on input line 101.
(c:/texlive/2024/texmf-dist/tex/context/base/mkii/supp-pdf.mkii
[Loading MPS to PDF converter (version 2006.09.02).]
\scratchcounter=\count332
\scratchdimen=\dimen268
\scratchbox=\box73
\nofMPsegments=\count333
\nofMParguments=\count334
\everyMPshowfont=\toks37
\MPscratchCnt=\count335
\MPscratchDim=\dimen269
\MPnumerator=\count336
\makeMPintoPDFobject=\count337
\everyMPtoPDFconversion=\toks38
) (c:/texlive/2024/texmf-dist/tex/latex/epstopdf-pkg/epstopdf-base.sty
Package: epstopdf-base 2020-01-24 v2.11 Base part for package epstopdf
Package epstopdf-base Info: Redefining graphics rule for `.eps' on input
line 4
85.
(c:/texlive/2024/texmf-dist/tex/latex/latexconfig/epstopdf-sys.cfg
File: epstopdf-sys.cfg 2010/07/13 v1.3 Configuration of (r)epstopdf for
TeX Liv
```

```
e
))
*geometry* driver: auto-detecting
*geometry* detected driver: pdftex
*geometry* verbose mode - [ preamble ] result:
* driver: pdftex
* paper: letterpaper
* layout: <same size as paper>
* layoutoffset:(h,v)=(0.0pt,0.0pt)
* modes:
* h-part:(L,W,R)=(56.9055pt, 500.484pt, 56.9055pt)
* v-part:(T,H,B)=(64.01869pt, 666.9326pt, 64.01869pt)
* \paperwidth=614.295pt
* \paperheight=794.96999pt
* \textwidth=500.484pt
* \textheight=666.9326pt
* \oddsidemargin=-15.36449pt
* \evensidemargin=-15.36449pt
* \topmargin=-44.2513pt
* \headheight=11.0pt
* \headsep=25.0pt
* \topskip=10.0pt
* \footskip=30.0pt
* \marginparwidth=65.0pt
* \marginparsep=11.0pt
* \columnsep=15.6491pt
* \skip\footins=9.0pt plus 4.0pt minus 2.0pt
* \hoffset=0.0pt
* \voffset=0.0pt
* \mag=1000
* \@twocolumnfalse
* \@twosidefalse
* \@mparswitchfalse
* \@reversemarginfalse
* (1in=72.27pt=25.4mm, 1cm=28.453pt)

Package caption Info: Begin \AtBeginDocument code.
Package caption Info: float package is loaded.
Package caption Info: hyperref package is loaded.
Package caption Info: longtable package is loaded.
(c:/texlive/2024/texmf-dist/tex/latex/caption/ltcaption.sty
Package: ltcaption 2021/01/08 v1.4c longtable captions (AR)
)
Package caption Info: rotating package is loaded.
Package caption Info: End \AtBeginDocument code.
LaTeX Info: Redefining \microtypecontext on input line 101.
Package microtype Info: Applying patch `item' on input line 101.
Package microtype Info: Applying patch `toc' on input line 101.
Package microtype Info: Applying patch `eqnum' on input line 101.
Package microtype Info: Applying patch `footnote' on input line 101.
Package microtype Info: Applying patch `verbatim' on input line 101.
Package microtype Info: Generating PDF output.
Package microtype Info: Character protrusion enabled (level 2).
Package microtype Info: Using default protrusion set `alltext'.
```

```
Package microtype Info: Automatic font expansion enabled (level 2),
(microtype)                stretch: 20, shrink: 20, step: 1, non-selected.
Package microtype Info: Using default expansion set `alltext-nott'.
LaTeX Info: Redefining \showhyphens on input line 101.
Package microtype Info: No adjustment of tracking.
Package microtype Info: No adjustment of interword spacing.
Package microtype Info: No adjustment of character kerning.
(c:/texlive/2024/texmf-dist/tex/latex/microtype/mt-ptm.cfg
File: mt-ptm.cfg 2006/04/20 v1.7 microtype config. file: Times (RS)
)
Package hyperref Info: Link coloring ON on input line 101.
(./article_3.out) (./article_3.out)
\@outlinefile=\write3
\openout3 = `article_3.out'.

LaTeX Font Info:    Trying to load font information for OT1+phv on input
line 1
07.
(c:/texlive/2024/texmf-dist/tex/latex/psnfss/ot1phv.fd
File: ot1phv.fd 2020/03/25 scalable font definitions for OT1/phv.
)
Package microtype Info: Loading generic protrusion settings for font
family
(microtype)                `phv' (encoding: OT1).
(microtype)                For optimal results, create family-specific
settings.
(microtype)                See the microtype manual for details.
LaTeX Font Info:    Trying to load font information for OT1+ztmcm on
input line
 107.
(c:/texlive/2024/texmf-dist/tex/latex/psnfss/ot1ztmcm.fd
File: ot1ztmcm.fd 2000/01/03 Fontinst v1.801 font definitions for
OT1/ztmcm.
)
Package microtype Info: Loading generic protrusion settings for font
family
(microtype)                `ztmcm' (encoding: OT1).
(microtype)                For optimal results, create family-specific
settings.
(microtype)                See the microtype manual for details.
LaTeX Font Info:    Trying to load font information for OML+ztmcm on
input line
 107.
(c:/texlive/2024/texmf-dist/tex/latex/psnfss/omlztmcm.fd
File: omlztmcm.fd 2000/01/03 Fontinst v1.801 font definitions for
OML/ztmcm.
)
LaTeX Font Info:    Trying to load font information for OMS+ztmcm on
input line
 107.
(c:/texlive/2024/texmf-dist/tex/latex/psnfss/omsztmcm.fd
File: omsztmcm.fd 2000/01/03 Fontinst v1.801 font definitions for
OMS/ztmcm.
)
```

```
LaTeX Font Info:    Trying to load font information for OMX+ztmcm on
input line
 107.
(c:/texlive/2024/texmf-dist/tex/latex/psnfss/omxztmcm.fd
File: omxztmcm.fd 2000/01/03 Fontinst v1.801 font definitions for
OMX/ztmcm.
)
LaTeX Font Info:    Font shape `OT1/ptm/bx/n' in size <12> not available
(Font)              Font shape `OT1/ptm/b/n' tried instead on input line
107.
LaTeX Font Info:    Font shape `OT1/ptm/bx/n' in size <9> not available
(Font)              Font shape `OT1/ptm/b/n' tried instead on input line
107.
LaTeX Font Info:    Font shape `OT1/ptm/bx/n' in size <7> not available
(Font)              Font shape `OT1/ptm/b/n' tried instead on input line
107.
(c:/texlive/2024/texmf-dist/tex/latex/microtype/mt-msa.cfg
File: mt-msa.cfg 2006/02/04 v1.1 microtype config. file: AMS symbols (a)
(RS)
) (c:/texlive/2024/texmf-dist/tex/latex/microtype/mt-msb.cfg
File: mt-msb.cfg 2005/06/01 v1.0 microtype config. file: AMS symbols (b)
(RS)
)
LaTeX Font Info:    Trying to load font information for TS1+phv on input
line 1
07.
(c:/texlive/2024/texmf-dist/tex/latex/psnfss/ts1phv.fd
File: ts1phv.fd 2020/03/25 scalable font definitions for TS1/phv.
)
Package microtype Info: Loading generic protrusion settings for font
family
(microtype)             `phv' (encoding: TS1).
(microtype)             For optimal results, create family-specific
settings.
(microtype)             See the microtype manual for details.
LaTeX Font Info:    Font shape `OT1/ptm/bx/n' in size <10> not available
(Font)              Font shape `OT1/ptm/b/n' tried instead on input line
107.
LaTeX Font Info:    Font shape `OT1/ptm/bx/n' in size <7.4> not available
(Font)              Font shape `OT1/ptm/b/n' tried instead on input line
107.
LaTeX Font Info:    Font shape `OT1/ptm/bx/n' in size <6> not available
(Font)              Font shape `OT1/ptm/b/n' tried instead on input line
107.
LaTeX Font Info:    Trying to load font information for OT1+pcr on input
line 1
07.
(c:/texlive/2024/texmf-dist/tex/latex/psnfss/ot1pcr.fd
File: ot1pcr.fd 2001/06/04 font definitions for OT1/pcr.
)
Package microtype Info: Loading generic protrusion settings for font
family
(microtype)             `pcr' (encoding: OT1).
```

```
(microtype)             For optimal results, create family-specific
settings.
(microtype)             See the microtype manual for details.
LaTeX Font Info:    Font shape `OT1/ptm/bx/n' in size <8> not available
(Font)              Font shape `OT1/ptm/b/n' tried instead on input line
107.
LaTeX Font Info:    Font shape `OT1/ptm/bx/n' in size <5> not available
(Font)              Font shape `OT1/ptm/b/n' tried instead on input line
107.
LaTeX Font Info:    Font shape `OT1/phv/m/it' in size <8> not available
(Font)              Font shape `OT1/phv/m/sl' tried instead on input line
107.

Package natbib Warning: Citation `jiang2020quantitative' on page 1
undefined on
 input line 118.


Package natbib Warning: Citation `gtex2020gtex' on page 1 undefined on
input li
ne 118.


Package natbib Warning: Citation `vidal2011interactome' on page 1
undefined on
input line 118.


Package natbib Warning: Citation `luck2020reference' on page 1 undefined
on inp
ut line 118.


Package natbib Warning: Citation `yeger2015human' on page 1 undefined on
input
line 118.


Package natbib Warning: Citation `greene2015understanding' on page 1
undefined
on input line 118.


Package natbib Warning: Citation `somepalli2021prioritizing' on page 1
undefine
d on input line 118.


Underfull \vbox (badness 10000) has occurred while \output is active []
```

```
Package natbib Warning: Citation `grubbs1969procedures' on page 1
undefined on
input line 120.


Package natbib Warning: Citation `nassif2021machine' on page 1 undefined
on inp
ut line 120.



[1{c:/texlive/2024/texmf-
var/fonts/map/pdftex/updmap/pdftex.map}{c:/texlive/202
4/texmf-dist/fonts/enc/dvips/base/8r.enc}


]
<infographic.jpg, id=17, 3521.155pt x 2489.3pt>
File: infographic.jpg Graphic file (type jpg)
<use infographic.jpg>
Package pdftex.def Info: infographic.jpg  used on input line 127.
(pdftex.def)             Requested size: 704.21854pt x 497.85118pt.

Overfull \hbox (37.28593pt too wide) in paragraph at lines 127--128
 [][]
 []


LaTeX Warning: Float too large for page by 9.36719pt on input line 130.





Overfull \hbox (833.38121pt too wide) has occurred while \output is
active
[] []|[] []
 []

[2


 <./infographic.jpg>]

Package natbib Warning: Citation `nassif2021machine' on page 3 undefined
on inp
ut line 134.


Package natbib Warning: Citation `akoglu2015graph' on page 3 undefined on
input
 line 134.
```

```
Package natbib Warning: Citation `michael2024detecting' on page 3
undefined on
input line 134.


Package natbib Warning: Citation `akoglu2015graph' on page 3 undefined on
input
 line 136.


Package natbib Warning: Citation `akoglu2015graph' on page 3 undefined on
input
 line 136.


Package natbib Warning: Citation `lee2021gawd' on page 3 undefined on
input lin
e 137.


Package natbib Warning: Citation `akoglu2010oddball' on page 3 undefined
on inp
ut line 138.


Package natbib Warning: Citation `davis2011detecting' on page 3 undefined
on in
put line 139.


Package natbib Warning: Citation `kagan2018generic' on page 3 undefined
on inpu
t line 139.


Package natbib Warning: Citation `lee2021gawd' on page 3 undefined on
input lin
e 140.


Package natbib Warning: Citation `lage2008large' on page 3 undefined on
input l
ine 145.


Package natbib Warning: Citation `greene2015understanding' on page 3
undefined
on input line 145.


Package natbib Warning: Citation `somepalli2021prioritizing' on page 3
undefine
d on input line 145.
```

```
Package natbib Warning: Citation `simonovsky2023predicting' on page 3
undefined
 on input line 145.


Package natbib Warning: Citation `basha2018differentialnet' on page 3
undefined
 on input line 145.


Package natbib Warning: Citation `sharon2023proact' on page 3 undefined
on inpu
t line 145.


Package natbib Warning: Citation `sharon2022differential' on page 3
undefined o
n input line 145.


Package natbib Warning: Citation `somepalli2021prioritizing' on page 3
undefine
d on input line 145.


Package natbib Warning: Citation `somepalli2021prioritizing' on page 3
undefine
d on input line 145.


Underfull \vbox (badness 10000) has occurred while \output is active []




Package natbib Warning: Citation `wgand_2025' on page 3 undefined on
input line
 146.


Overfull \hbox (11.19746pt too wide) in paragraph at lines 145--147
\OT1/ptm/m/n/10 (-20) vel-oped Weighted Graph Anoma-lous Node De-tec-tion
(WGAN
D),
 []


Package natbib Warning: Citation `basha2018differentialnet' on page 3
undefined
 on input line 152.
```

```
[3

]

Package natbib Warning: Citation `arsov2019network' on page 4 undefined
on inpu
t line 164.


Package natbib Warning: Citation `zhang2018billion' on page 4 undefined
on inpu
t line 164.


Package natbib Warning: Citation `breiman2001random' on page 4 undefined
on inp
ut line 166.


Overfull \hbox (3.83278pt too wide) in paragraph at lines 166--167
\OT1/ptm/m/n/10 (-20) GBM pre-sented the best per-for-mance in terms of
Mean Sq
uared
 []


Package natbib Warning: Citation `simonovsky2023predicting' on page 4
undefined
 on input line 172.

<Figure 2_new.jpg, id=45, 1357.7124pt x 1037.5563pt>
File: Figure 2_new.jpg Graphic file (type jpg)
<use Figure 2_new.jpg>
Package pdftex.def Info: Figure 2_new.jpg  used on input line 176.
(pdftex.def)             Requested size: 450.43253pt x 344.2153pt.

Underfull \vbox (badness 1112) has occurred while \output is active []




Overfull \hbox (0.42pt too wide) in paragraph at lines 183--183
\OT1/phv/b/n/10 (-20) forms Other Meth-ods in De-tect-ing Anoma-lous
Nodes|
 []


Overfull \hbox (2.42996pt too wide) in paragraph at lines 184--185
\OT1/ptm/m/n/10 (-20) Next, we aimed to con-struct an un-su-per-vised
machine-l
earning-
 []
```

```
<Figure 3.jpg, id=54, 963.6pt x 542.025pt>
File: Figure 3.jpg Graphic file (type jpg)
<use Figure 3.jpg>
Package pdftex.def Info: Figure 3.jpg  used on input line 192.
(pdftex.def)             Requested size: 475.45825pt x 267.43892pt.
<Figure 4.jpg, id=55, 963.6pt x 542.025pt>
File: Figure 4.jpg Graphic file (type jpg)
<use Figure 4.jpg>
Package pdftex.def Info: Figure 4.jpg  used on input line 201.
(pdftex.def)             Requested size: 475.45825pt x 267.43892pt.

Package natbib Warning: Citation `hekselman2022organ' on page 4 undefined
on in
put line 215.


Underfull \vbox (badness 2961) has occurred while \output is active []



[4]
Underfull \vbox (badness 10000) has occurred while \output is active []




Package natbib Warning: Citation `basha2018differentialnet' on page 5
undefined
 on input line 220.


Package natbib Warning: Citation `sharon2023proact' on page 5 undefined
on inpu
t line 220.


Package natbib Warning: Citation `somepalli2021prioritizing' on page 5
undefine
d on input line 220.


Package natbib Warning: Citation `sharon2023proact' on page 5 undefined
on inpu
t line 225.


Underfull \vbox (badness 3780) has occurred while \output is active []



[5 <./Figure 2_new.jpg>]

Package natbib Warning: Citation `Pham2023' on page 6 undefined on input
line 2
```

```
30.


Package natbib Warning: Citation `Rasmussen2021' on page 6 undefined on input l
ine 230.


Package natbib Warning: Citation `Sandaradura2018' on page 6 undefined on input
 line 230.


Package natbib Warning: Citation `Meester2017' on page 6 undefined on input lin
e 230.


Underfull \vbox (badness 10000) has occurred while \output is active []




LaTeX Warning: File `validation fig_ver3.png' not found on input line 235.


! Package pdftex.def Error: File `validation fig_ver3.png' not found: using dra
ft setting.

See the pdftex.def package documentation for explanation.
Type  H <return>  for immediate help.
 ...

l.235 ...dth=1\textwidth]{validation fig_ver3.png}

Try typing  <return>  to proceed.
If that doesn't work, type  X <return>  to quit.


Package natbib Warning: Citation `sharon2023proact' on page 6 undefined on inpu
t line 247.


Package natbib Warning: Citation `basha2018differentialnet' on page 6 undefined
 on input line 247.


Package natbib Warning: Citation `somepalli2021prioritizing' on page 6 undefine
d on input line 247.
```

```
Package natbib Warning: Citation `simonovsky2023predicting' on page 6
undefined
 on input line 247.


Package natbib Warning: Citation `zietz2024probability' on page 6
undefined on
input line 251.



[6 <./Figure 3.jpg>]
Underfull \vbox (badness 10000) has occurred while \output is active []




Package natbib Warning: Citation `akiba2019optuna' on page 7 undefined on
input
 line 257.


Underfull \vbox (badness 10000) has occurred while \output is active []



[7 <./Figure 4.jpg>] [8]

Package natbib Warning: Citation `saha2017co' on page 9 undefined on
input line
 282.


Package natbib Warning: Citation `the2022tabula' on page 9 undefined on
input l
ine 282.


Underfull \vbox (badness 2635) has occurred while \output is active []




Package natbib Warning: Citation `noble2003graph' on page 9 undefined on
input
line 289.


Package natbib Warning: Citation `akoglu2010oddball' on page 9 undefined
on inp
ut line 289.
```

```
Package natbib Warning: Citation `kagan2018generic' on page 9 undefined
on inpu
t line 296.



[9]

Package natbib Warning: Citation `grohe2020word2vec' on page 10 undefined
on in
put line 302.


Package natbib Warning: Citation `grover2016node2vec' on page 10
undefined on i
nput line 304.


Package natbib Warning: Citation `grover2016node2vec' on page 10
undefined on i
nput line 305.


Package natbib Warning: Citation `zhang2018billion' on page 10 undefined
on inp
ut line 305.


Package natbib Warning: Citation `torres2020glee' on page 10 undefined on
input
 line 305.


Package natbib Warning: Citation `yang2019nodesketch' on page 10
undefined on i
nput line 305.


Package natbib Warning: Citation `perozzi2014deepwalk' on page 10
undefined on
input line 305.


Package natbib Warning: Citation `karateclub' on page 10 undefined on
input lin
e 305.


Package natbib Warning: Citation `karateclub' on page 10 undefined on
input lin
e 305.
```

```
Package natbib Warning: Citation `breiman2001random' on page 10 undefined
on in
put line 307.


Package natbib Warning: Citation `chen2016xgboost' on page 10 undefined
on inpu
t line 307.


Package natbib Warning: Citation `ke2017lightgbm' on page 10 undefined on
input
 line 307.


Package natbib Warning: Citation `kagan2018generic' on page 10 undefined
on inp
ut line 313.



Overfull \hbox (2.69673pt too wide) in paragraph at lines 317--318
[]\OT1/ptm/b/n/10 (-20) Mean Er-ror \OT1/ptm/m/n/10 (-20) - []The av-er-
age[] e
r-ror of the pre-dicted weights
 []


Overfull \hbox (5.13174pt too wide) in paragraph at lines 325--326
[]\OT1/ptm/m/n/10 (-20) of the in-ter-ac-tions be-tween node
$\OML/ztmcm/m/it/1
0 v$ \OT1/ptm/m/n/10 (-20) and its neigh-bors:[]$[]$.
 []


Overfull \hbox (8.38164pt too wide) in paragraph at lines 384--392
 [][]
 []



[10]

Package natbib Warning: Citation `aggarwal2015data' on page 11 undefined
on inp
ut line 404.


Package natbib Warning: Citation `liu2008isolation' on page 11 undefined
on inp
ut line 406.
```

```
Package natbib Warning: Citation `fawcett2006introduction' on page 11
undefined
 on input line 413.


Package natbib Warning: Citation `boyd2013area' on page 11 undefined on
input l
ine 413.


Package natbib Warning: Citation `fawcett2006introduction' on page 11
undefined
 on input line 413.


Package natbib Warning: Citation `boyd2013area' on page 11 undefined on
input l
ine 413.



Package natbib Warning: Citation `lee2021gawd' on page 11 undefined on
input li
ne 422.


Package natbib Warning: Citation `akoglu2010oddball' on page 11 undefined
on in
put line 424.


Package natbib Warning: Citation `basha2020differential' on page 11
undefined o
n input line 428.


Package natbib Warning: Citation `basha2020differential' on page 11
undefined o
n input line 428.


[11]

Package natbib Warning: Citation `hekselman2022organ' on page 12
undefined on i
nput line 460.


Package natbib Warning: Citation `white2020pubmed' on page 12 undefined
on inpu
t line 460.
```

```
Package natbib Warning: Citation `stelzer2016genecards' on page 12 
undefined on
 input line 460.


Package natbib Warning: Citation `kohler2021human' on page 12 undefined 
on inpu
t line 460.


Package natbib Warning: Citation `basha2020differential' on page 12 
undefined o
n input line 460.


Package natbib Warning: Citation `hekselman2022organ' on page 12 
undefined on i
nput line 463.


Package natbib Warning: Citation `basha2020differential' on page 12 
undefined o
n input line 463.


Package natbib Warning: Citation `sharon2023proact' on page 12 undefined 
on inp
ut line 463.



Overfull \hbox (10.24992pt too wide) in paragraph at lines 465--465
|\OT1/phv/b/n/10 (-20) Comparison of the WGAND Anomaly De-tec-tion Method
 []


Package natbib Warning: Citation `basha2018differentialnet' on page 12 
undefine
d on input line 467.


Package natbib Warning: Citation `sharon2023proact' on page 12 undefined 
on inp
ut line 467.


Package natbib Warning: Citation `somepalli2021prioritizing' on page 12 
undefin
ed on input line 467.
```

```
Package natbib Warning: Citation `somepalli2021prioritizing' on page 12
undefin
ed on input line 467.


Package natbib Warning: Citation `basha2018differentialnet' on page 12
undefine
d on input line 477.


Package natbib Warning: Citation `sharon2023proact' on page 12 undefined
on inp
ut line 477.


Package natbib Warning: Citation `simonovsky2023predicting' on page 12
undefine
d on input line 477.


Package natbib Warning: Citation `hekselman2022organ' on page 12
undefined on i
nput line 477.


Package natbib Warning: Citation `somepalli2021prioritizing' on page 12
undefin
ed on input line 477.


Package natbib Warning: Citation `kagan2025supporting' on page 12
undefined on
input line 477.


Underfull \hbox (badness 10000) in paragraph at lines 484--484
[]|\OT1/ptm/m/n/10 (+20) WGAND
 []


Underfull \hbox (badness 10000) in paragraph at lines 485--485
[]|[][]$\OT1/pcr/m/n/10 https : / / github . com /
 []


Underfull \hbox (badness 7814) in paragraph at lines 490--490
\OT1/ptm/m/n/10 (+20) scikit-learn, numpy, net-
 []


Package natbib Warning: Citation `wgand_2025' on page 12 undefined on
input lin
e 492.
```

[12]
No file article_3.bbl.

[13

]
<Figure S1.jpg, id=171, 4585.13pt x 4521.89375pt>
File: Figure S1.jpg Graphic file (type jpg)
<use Figure S1.jpg>
Package pdftex.def Info: Figure S1.jpg used on input line 547.
(pdftex.def)   Requested size: 450.43253pt x 444.21205pt.
<Figure S2.jpg, id=172, 4709.595pt x 4508.845pt>
File: Figure S2.jpg Graphic file (type jpg)
<use Figure S2.jpg>
Package pdftex.def Info: Figure S2.jpg used on input line 555.
(pdftex.def)   Requested size: 450.43253pt x 431.2343pt.
<Figure S3.jpg, id=173, 1138.0116pt x 1422.7554pt>
File: Figure S3.jpg Graphic file (type jpg)
<use Figure S3.jpg>
Package pdftex.def Info: Figure S3.jpg used on input line 564.
(pdftex.def)   Requested size: 450.43253pt x 563.12198pt.

[14

 <./Figure S1.jpg>]

[15 <./Figure S2.jpg>]

[16 <./Figure S3.jpg>]

LaTeX Warning: File `supp fig.png' not found on input line 573.

! Package pdftex.def Error: File `supp fig.png' not found: using draft setting.

See the pdftex.def package documentation for explanation.
Type H <return> for immediate help.
 ...

l.573   \includegraphics[scale=0.3]{supp fig.png}

Try typing <return> to proceed.
If that doesn't work, type X <return> to quit.

```
[17

]

LaTeX Warning: No positions in optional float specifier.
               Default added (so using `tbp') on input line 583.


LaTeX Warning: No positions in optional float specifier.
               Default added (so using `tbp') on input line 616.



[18]
Underfull \hbox (badness 1540) in paragraph at lines 683--683
\OT1/ptm/m/n/9 (+20) Cere-bel-lar Hy-popla-sia And Cor-ti-cal
 []


Underfull \hbox (badness 2990) in paragraph at lines 715--715
[]|\OT1/ptm/m/n/9 (+20) Cardiomyopathy, Di-lated, 1D, Car-
 []


Underfull \hbox (badness 2990) in paragraph at lines 724--724
[]|\OT1/ptm/m/n/9 (+20) Cardiomyopathy, Di-lated, 1D, Car-
 []


Underfull \hbox (badness 2990) in paragraph at lines 725--725
[]|\OT1/ptm/m/n/9 (+20) Cardiomyopathy, Di-lated, 2A, Car-
 []

LaTeX Font Info:    Trying to load font information for TS1+ptm on input
line 7
29.
(c:/texlive/2024/texmf-dist/tex/latex/psnfss/ts1ptm.fd
File: ts1ptm.fd 2001/06/04 font definitions for TS1/ptm.
)
Underfull \hbox (badness 3884) in paragraph at lines 731--731
[]|\OT1/ptm/m/n/9 (+20) Analbuminemia, Hy-per-thy-rox-ine-mia,
 []


Underfull \hbox (badness 7540) in paragraph at lines 733--733
[]|\OT1/ptm/m/n/9 (+20) Phosphoenolpyruvate Car-boxyk-i-nase
 []


Underfull \hbox (badness 3514) in paragraph at lines 745--745
\OT1/ptm/m/n/9 (+20) or-rhagic Dis-ease Due To Alpha-1-
 []
```

```
Underfull \hbox (badness 6284) in paragraph at lines 749--749
[]|\OT1/ptm/m/n/9 (+20) Arthrogryposis, Dis-tal, Type 2B2,
 []


Underfull \hbox (badness 10000) in paragraph at lines 755--755
[]|\OT1/ptm/m/n/9 (+20) Myopathy, Scapu-lo-humeroper-oneal,
 []


Underfull \hbox (badness 5622) in paragraph at lines 755--755
\OT1/ptm/m/n/9 (+20) Con-gen-i-tal My-opa-thy 2A, Typ-i-cal,
 []


Underfull \hbox (badness 2990) in paragraph at lines 757--757
[]|\OT1/ptm/m/n/9 (+20) Lethal Con-gen-i-tal Con-trac-ture Syn-
 []


Underfull \hbox (badness 3302) in paragraph at lines 789--789
\OT1/ptm/m/n/9 (+20) molytic, Ichthyosis Hys-trix, Curth-
 []


Underfull \hbox (badness 1688) in paragraph at lines 791--791
\OT1/ptm/m/n/9 (+20) Naegeli-Franceschetti-Jadassohn Syn-
 []


Underfull \hbox (badness 3158) in paragraph at lines 797--797
[]|\OT1/ptm/m/n/9 (+20) Ectodermal Dys-pla-sia/Skin Fragility
 []


Underfull \hbox (badness 3302) in paragraph at lines 799--799
\OT1/ptm/m/n/9 (+20) molytic, Ichthyosis Hys-trix, Curth-
 []


Underfull \hbox (badness 1688) in paragraph at lines 804--804
\OT1/ptm/m/n/9 (+20) Naegeli-Franceschetti-Jadassohn Syn-
 []


Underfull \hbox (badness 3158) in paragraph at lines 807--807
[]|\OT1/ptm/m/n/9 (+20) Ectodermal Dys-pla-sia/Skin Fragility
 []


Underfull \hbox (badness 10000) in paragraph at lines 825--825
[]|\OT1/ptm/m/n/9 (+20) Hemoglobin H Dis-ease, Alpha-
 []
```

```
[19



] [20] [21] [22] [23] [24]

[25]

Package natbib Warning: There were undefined citations.


enddocument/afterlastpage: lastpage setting LastPage.
(./article_3.aux)
 ***********
LaTeX2e <2024-06-01> patch level 2
L3 programming layer <2024-05-27>
 ***********
Package rerunfilecheck Info: File `article_3.out' has not changed.
(rerunfilecheck)             Checksum:
D41D8CD98F00B204E9800998ECF8427E;0.
 )
Here is how much of TeX's memory you used:
 18964 strings out of 473583
 298664 string characters out of 5732343
 2112908 words of memory out of 5000000
 40935 multiletter control sequences out of 15000+600000
 646716 words of font info for 493 fonts, out of 8000000 for 9000
 1141 hyphenation exceptions out of 8191
 85i,14n,79p,1938b,770s stack positions out of
10000i,1000n,20000p,200000b,200000s
<c:/texlive/2024/texmf-
dist/fonts/type1/public/amsfonts/cm/cmmi10.pfb><c:/tex
live/2024/texmf-
dist/fonts/type1/public/amsfonts/cm/cmr10.pfb><c:/texlive/2024/
texmf-
dist/fonts/type1/public/amsfonts/cm/cmsy10.pfb><c:/texlive/2024/texmf-dis
t/fonts/type1/urw/courier/ucrr8a.pfb><c:/texlive/2024/texmf-
dist/fonts/type1/ur
w/helvetic/uhvb8a.pfb><c:/texlive/2024/texmf-
dist/fonts/type1/urw/helvetic/uhvr
8a.pfb><c:/texlive/2024/texmf-
dist/fonts/type1/urw/helvetic/uhvro8a.pfb><c:/tex
live/2024/texmf-
dist/fonts/type1/urw/symbol/usyr.pfb><c:/texlive/2024/texmf-dis
t/fonts/type1/urw/symbol/usyr.pfb><c:/texlive/2024/texmf-
dist/fonts/type1/urw/t
imes/utmb8a.pfb><c:/texlive/2024/texmf-
dist/fonts/type1/urw/times/utmr8a.pfb><c
:/texlive/2024/texmf-dist/fonts/type1/urw/times/utmri8a.pfb>
Output written on article_3.pdf (25 pages, 14388952 bytes).
PDF statistics:
 305 PDF objects out of 1000 (max. 8388607)
```

243 compressed objects within 3 object streams
65 named destinations out of 1000 (max. 500000)
123940 words of extra memory for PDF output out of 128383 (max. 10000000)

Manuscript

Click here to access/download;Manuscript;Stylish_article_template___Gene-

Network-based anomaly detection algorithm reveals proteins with major roles in human tissues

Dima Kagan[1*], Juman Jubran[2*], Esti Yeger-Lotem[2,3,§], and Michael Fire[1,§]

Abstract

Background

Proteins act through physical interactions with other molecules to maintain organismal health. Protein-protein interaction (PPI) networks proved to be a powerful framework for obtaining insight into protein functions, cellular organization, response to signals, and disease states. In multicellular organisms, protein content varies between tissues, influencing tissue morphology and function. Weighted PPI networks, reflecting the likelihood of interactions in specific tissues, offer insights into tissue-specific processes and disease mechanisms. We hypothesized that detecting anomalous nodes in these networks could reveal proteins with key tissue-specific functions.

Results

Here, we introduce Weighted Graph Anomalous Node Detection (WGAND), a novel machine-learning algorithm to identify anomalous nodes in weighted graphs. WGAND estimates expected edge weights and uses deviations to generate anomaly detection features, which are then used to score network nodes. We applied WGAND to weighted PPI networks of 17 human tissues. High-ranking anomalous nodes were enriched for proteins associated with tissue-specific diseases and tissue-specific biological processes, such as neuron signaling in the brain and spermatogenesis in the testis. WGAND outperformed other methods in terms of Area Under the ROC Curve (AUC) and Precision at K (P@K), highlighting its effectiveness in uncovering biologically meaningful anomalies.

Conclusion

Our findings demonstrate WGAND's potential as a powerful tool for detecting anomalous proteins with significant biological roles. By identifying proteins involved in critical tissue-specific processes and diseases, WGAND offers valuable insights for discovering novel biomarkers and therapeutic targets. Its versatile algorithm is suitable for any weighted graph and is broadly applicable across various fields. The WGAND algorithm is available as an open-source Python library at `https://github.com/data4goodlab/wgand`.

Keywords

Protein-Protein Interaction (PPI) Networks — Anomaly Detection — Weighted Graphs — Machine Learning

[1] *Department of Software and Information Systems Engineering, Ben-Gurion University of the Negev, Beer Sheva 84105, Israel*

[2] *Department of Clinical Biochemistry and Pharmacology, Ben-Gurion University of the Negev, Beer Sheva 84105, Israel*

[3] *The National Institute for Biotechnology in the Negev, Ben-Gurion University of the Negev, Beer Sheva 84105, Israel*

[*] Dima Kagan and Juman Jubran have contributed equally to this work.

[†] Corresponding authors.

Email: kagandi@post.bgu.ac.il, juman@post.bgu.ac.il, estiyl@bgu.ac.il, mickyfi@bgu.ac.il

Introduction

Proteins are the prime molecules in living cells, driving and mediating all biological processes through interactions with other molecules. In multi-tissue organisms, protein content differs between tissues, as proteins could either be absent from certain tissues or expressed at different levels [1]. These differences affect tissues' morphology and function and susceptibility to aberrations [2]. Protein-protein interaction (PPI) networks, representing the interactions between proteins within a cell, provide a valuable framework for studying protein functions and cellular information transfer [3]. Weighted PPI networks, where edge weight reflects the likelihood of an interaction, offer a refined view of context-specific interactions [4]. For example, weighted PPI networks representing different human tissues helped understand tissue-specific protein functions, biological processes, and differences in disease susceptibilities [5, 6, 7].

In studying such complex networks, detecting anomalies could offer a unique perspective. Detecting anomalies is a critical problem in diverse domains, from cybersecurity to social network analysis. Anomalies refer to elements or objects "that appear to deviate markedly from other members of the sample in which it occurs" [8]. The study of anomalies has fascinated scientists for centuries, as they often hold unique insights into the dynamics of complex systems [9].

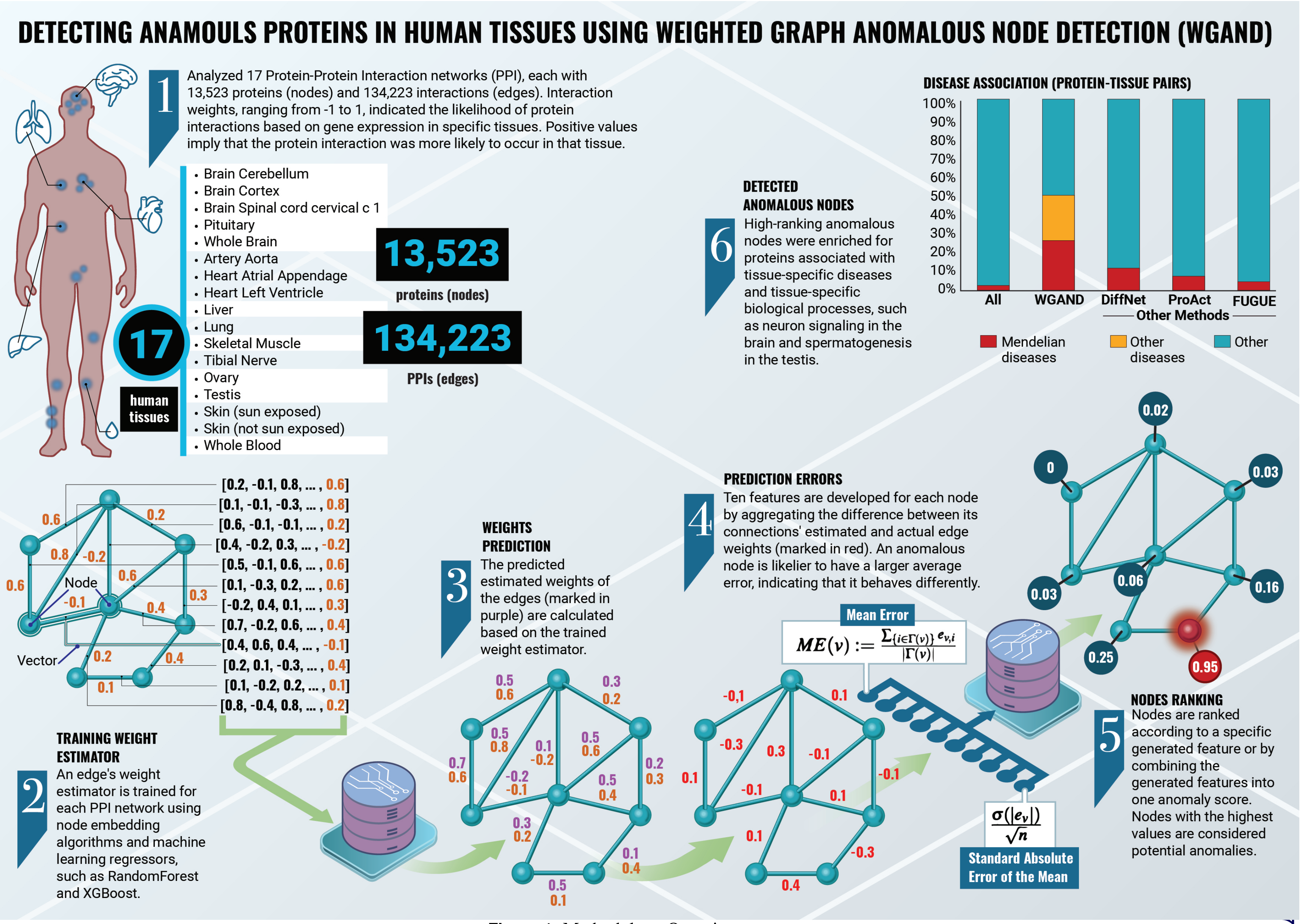

Figure 1. Methodology Overview.

Anomaly detection is an invaluable tool for various disciplines, from aviation to medicine, to uncover insights not easily gained through traditional methods [9]. For instance, in aviation, an anomaly found in airplane sensors may indicate a fault, while in medicine, it could signify a rare disease [10]. In biomedicine, many advances can be attributed to identifying unusual proteins and genes [11].

In recent years, anomaly detection in graphs (i.e., networks) has gained increasing attention due to its applicability in various domains such as cybersecurity, social networks, and biological networks [10]. The structure and interconnected nature of graphs can provide rich information for anomaly detection. However, the graph's complex nature, including its size, diversity, and noise, makes this task challenging [10]. Over the years, many studies have addressed anomaly detection, especially in graphs. However, only a few studies focused on anomaly detection in weighted graphs [12]. For example, Akoglu et al. [13] introduced OddBall, an algorithm that detects anomalous nodes in weighted graphs. OddbBall focuses on neighborhoods around each node and extracts features from these neighborhoods. Then, these features are transformed into a score to pinpoint outliers. The following year, Davis et al. [14] presented Yagada, an algorithm that searches for structural and numerical anomalies in labelled graphs. In 2018, Kagan et al. [15] introduced an unsupervised classifier based on network topology to detect anomalous nodes. Their algorithm operates on the assumption that nodes with many improbable links are more likely to be anomalous. They successfully applied this method to networks of varying scales, demonstrating its ability to identify abnormal nodes effectively. More recently, Lee et al. [12] introduced GAWD, a method for detecting outliers in weighted graphs. GAWD iterates through the graph, searching for the ”best” substructures, which generate the largest compression when replaced with a super-node. The anomaly score is generated based on how well it was compressed.

In this study, we hypothesized that proteins involved in key biological roles within a specific tissue would appear as anomalous nodes in the PPI network, representing that tissue. Among these proteins are those involved in main tissue processes, such as neuron signaling in the brain and spermatogenesis in the testis, and those that participate in tissue-specific diseases, such as BRCA1, whose aberrations increase the risk for breast and ovarian cancers. Previous methods aiming to reveal proteins with key tissue-specific roles have focused on the preferential expression of proteins in specific tissues [16], their network features [6], or both [7, 17]. For example, using the DiffNet method for scoring protein interactions in tissue contexts [18], it was shown that subnetworks composed of the top 1% differential interaction were enriched for tissue-associated disease proteins. Likewise, by using the ProAct method for scoring biological processes [19], it was shown that tissue-associated disease proteins participated in processes that were highly active in the respective tissue [20]. Lastly, FUGUE predicted tissue-relevant genes by applying supervised machine learning methods to transcriptional and network features of genes [7]. FUGUE distinguished tissue or cell type-specific genes better than conventional methods that used tissue-specific expression alone [7].

Here, we combined expression and network features with the anomaly concept. To identify anomalous proteins, we developed Weighted Graph Anomalous Node Detection (WGAND), a novel generic anomaly detection machine learning-based algorithm for weighted graphs. WGAND is constructed based on the assumption that edge weights of anomalous nodes would deviate from their expected norm. Thus, the model estimated the expected edge weight for all edges in the network and then used the difference between the actual and the expected weight to generate anomaly detection features. These features were then used to train a model that generates anomaly scores for all the network nodes. To test our hypothesis, we applied the WGAND algorithm to weighted PPI networks of 17 different human tissues, where each network was composed of 13,523 nodes (proteins) and 134,223 edges (PPIs) with the same topology but with different edge weights. When applied to identify disease tissue-associated proteins, we found that in most tissues, WGAND obtained a higher area under the ROC curve (AUC), a higher area under the precision-recall curve (PR-AUC), and a higher precision at K (P@K) than other methods. Moreover, high-ranking anomalous proteins were more likely to be associated with diseases than other proteins, were enriched for proteins involved in tissue-specific processes, and tended to be involved in processes that were preferentially active in the respective tissues. Notably, WGAND outperformed three other methods for identifying tissue-relevant or disease-associated proteins. Whereas our study used weighted PPI networks as a case study, WGAND is a generic method that could be applied to any weighted graph. WGAND is an open Python library [21].

Results

To create an anomaly detection model based on weighted graphs, we used 17 tissue-specific differential PPI networks (Methods, [18]). Each network consisted of 13,523 nodes (proteins) and 134,223 edges (PPIs). These networks had identical topologies and differed from each other only in their edge weights, ranging from -1 to 1 (Fig. 1 step 1).

The anomaly detection method assumes that the edge weights of anomalous nodes are likely to deviate from their expected norm. Hence, we constructed an anomaly detection model:

1. We trained an edge weight estimator to predict edge weights (Fig. 1, step 2).
2. We constructed meta-features based on the error of the edge weight prediction (Fig. 1, steps 3 and 4).
3. We trained a model on the meta-features to predict anomalous nodes (Fig. 1, steps 5 and 6).

Using Edge Weight Estimation Errors to Identify Anomalous Nodes

To build an edge weight estimator, we used node embedding methods [22] to generate features representing the nodes, allowing us to predict the edge weight. We tested and evaluated five different node embedding models and assessed their performance in detecting anomalous nodes using *AUC*, *PR-AUC*, *P@K*, and *runtime* (Table 1). On average, the RandNE [23] embedding method showed the highest performance by all metrics, including the fastest average runtimes in generating the embeddings (Table 1). Although its AUC is slightly higher than other models, its *P@K* and *PR-AUC* show considerable gains compared to the rest of the models.

Next, we used the node embedding features generated by RandNE and constructed three different edge weight estimators using different regression algorithms (Methods, Fig. 1 step 2). First, we evaluated their performances just on the *edge-weight estimation task*. The model created by LightGBM presented the best performance in terms of Mean Squared Error (MSE) (Table 2). Second, we evaluated the performance of the generated anomaly detection models (Table 3). We found that, on average, when using the Random Forest [24] as an edge weight estimator to generate the meta-features, the anomaly detection model achieved the highest score in all anomaly detection metrics. Therefore, we chose the combination of RandNE embedding and Random Forest edge weight estimator, which presented the best results for the anomaly detection model construction.

We turned to check whether the edge weight estimator could provide biological insights (Methods). Per tissue network, we created a PPI subnetwork that only contained edges with a high difference between predicted and actual values (Fig. 1 step 3). Within each subnetwork, we labeled the tissue-associated disease proteins, namely proteins associated with Mendelian diseases that manifest in that tissue. We hypothesized that proteins that appear on paths between tissue-associated disease proteins are likely to have central roles in the tissue. Hence, we counted how many times each protein appeared on a path connecting two tissue-associated disease proteins. We focused on the top-10 proteins per network that participated in the largest number of such paths.

We used the TRACE tool [17] to rank these proteins by their likelihood of being tissue-associated disease proteins in the given tissue and in other tissues. The top-10 proteins were ranked significantly higher in the given tissue relative to their median ranking in other tissues ($p-value = 7.57e-6$; Wilcoxon test; Fig. 2). In addition, except for brain cerebellum tissue, the top-10 proteins ranked significantly higher relative to other proteins of the same tissue (adjusted $p-value \leq 8.12e-3$, Mann-Whitney test, Benjamini-Hochberg correction; Fig. 2). Taken together, these results show that the top-10 proteins were not randomly picked but could potentially have tissue-specific roles. Overall, this analysis suggests that we could identify anomalous nodes based on the error in predicting their edge weight.

The Ensemble Anomaly Detection Method Outperforms Other Methods in Detecting Anomalous Nodes

Next, we aimed to construct an unsupervised machine-learning-based anomaly detector model to detect anomalous nodes. Per tissue PPI network, we assigned nodes with ten meta-features constructed based on the edge weight prediction error (Methods, Fig. 1 step 4). Then, using the edges' actual weights, we evaluated the performance of four suggested anomaly detector models and compared them to two baseline models (Methods). On average, all four anomaly detector models present superior performance over the baseline models in all metrics with respect to AUC and P@K (Fig. 3). In terms of AUC, the ensemble and the feature's mean presented the best performance with a marginal difference. In terms of P@K, we saw similar performance between the ensemble, PCA, and the feature's mean with a slight advantage for the ensemble. In addition, we evaluated the performance of the ensemble method against the baseline models of each tissue network separately. The ensemble method had a higher AUC than the baselines across all tissues, higher PR-AUC in 16 out of 17 tissues, and higher P@20 in 13 out of 17 (Table S1). Overall, the ensemble method – 'WGAND (ensemble)' - performed best. For 85% of the datasets, the proposed method achieved higher performance than the baselines in terms of AUC and P@K. Figures 4 and S1 show significant variance between the different methods in most tissues. Furthermore, we observed some tissues, such as the Brain Spinal cord cervical c 1, where the anomaly detector achieves high AUC but relatively lower P@K values (Table S1).

Next, to inspect which feature could explain anomaly best and may be used as an indicator in unlabeled networks, we used the "WGAND (ensemble)" method and constructed a model per feature. The models were evaluated based on their P@K values (Fig. S2). Most features had P@K higher than random in all the tissue networks (Table S2). The features with the best performance on average were the Sum of Errors, Error Standard Deviation, Mean Error, and Mean Absolute Error.

WGAND Predicts Anomalous Proteins with Major Biological Roles

We hypothesized that tissue-associated anomalous proteins would play major biological roles in the respective tissue. Examples of major biological roles include involvement in a tissue-associated disease (e.g., Duchenne muscular dystrophy in skeletal muscle) or in a biological process that is highly active specifically in that tissue (e.g., neuron signaling in brain tissue). Notably, we do not assume that an anomalous node necessarily causes a tissue-associated disease or a certain biological process, only that it is associated with them and thus appears different in the networks. We tested this hypothesis for the top-10 most likely anomalous proteins per tissue network according to the best-performing combination "WGAND (ensemble)."

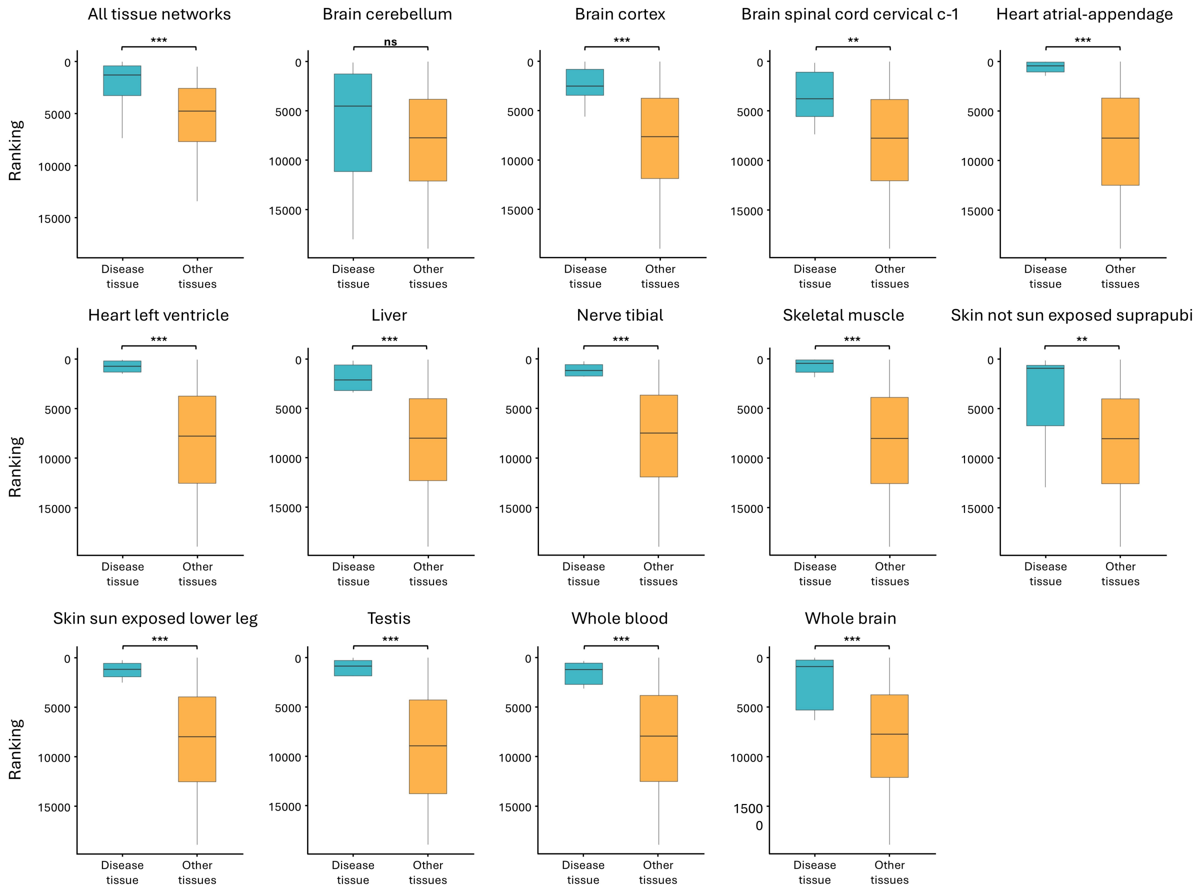

Figure 2. Using edge weight estimation errors to identify tissue-associated disease proteins. The 10 proteins that were most frequently on paths connecting tissue-associated disease proteins were significantly more likely to be tissue-associated disease proteins in that tissue (blue) than in other tissues (orange; $p-value = 7.57e-6$, Wilcoxon test). Top-10 proteins were collated from 13 tissue networks. Adjusted p-values for cerebellum, cortex, spinal cord, heart atrial appendage, heart left ventricle, liver, nerve tibial, skeletal muscle, skin not sun-exposed, skin sun-exposed, testis, whole blood and whole brain in respective order: $0.13, 8.14e-4, 8.12e-3, 2.75e-6, 2.46e-6, 9.21e-4, 3.85e-5, 3.03e-5, 1.85e-3, 9.83e-5, 3.11e-5, 1.16-4$, and $8.6e-4$ (one-sided Mann-Whitney test, Benjamini-Hochberg correction).

To test for involvement in tissue-associated diseases, we first assessed the association of the top-10 anomalous proteins with tissue-selective Mendelian diseases affecting the relevant tissue [25] (Methods). We found that 26% of the top-10 anomalous proteins per tissue (a total of 170 proteins) were indeed tissue-associated diseases proteins (Fig. 5A). To assess whether this percentage is expected by chance, we assessed it for all proteins in our networks. We found that only 1.5% of all proteins were tissue-associated disease proteins, a 17-fold decrease relative to the top-10 anomalous proteins. To go beyond Mendelian diseases, we manually mined the literature and disease-related databases for additional associations between the top-10 proteins per tissue and non-Mendelian diseases and phenotypes affecting the same tissue. This revealed that an additional 24% of the top-10 proteins were associated with diseases and phenotypes of that tissue (Fig. 5A and Table S3). Hence, top-10 anomalous proteins per tissue were highly enriched for proteins involved in diseases and phenotypes affecting the respective anomaly-relevant tissue.

Next, we compared WGAND to three other methods, including DiffNet [18], ProACT [19], and FUGUE [7] (Methods). All three methods were tested previously for their ability to detect tissue-associated disease proteins. Hence, per tissue, we ranked proteins according to each method. Then, we checked how many of the top-10 highly-ranked proteins were tissue-associated disease proteins. According to DiffNet, ProAct, and FUGUE, only 11%, 6%, and 4%, respectively, were tissue-associated diseases proteins in the respective tissue, compared with 24% for WGAND (Fig. 5A). Hence, WGAND outperformed all three other methods.

To test the involvement of anomalous proteins in tissue-associated biological processes, we checked how many of the top-10 anomalous proteins were annotated as participating in a biological process that was specific to that tissue (Meth-

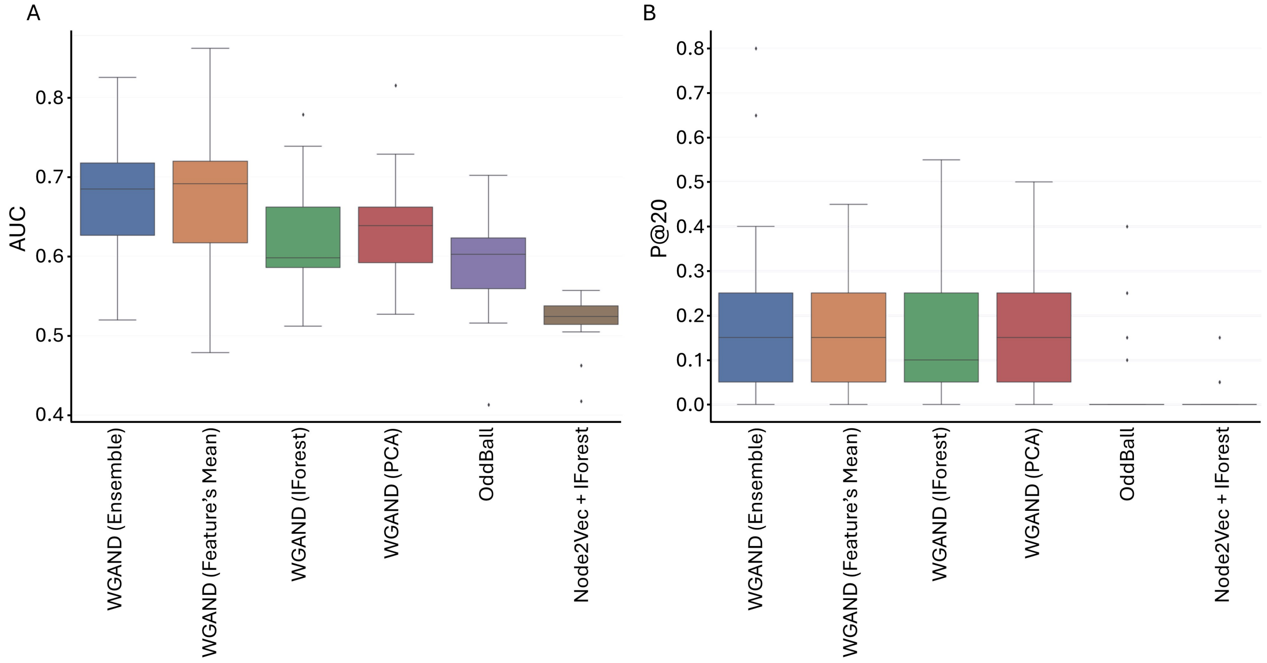

Figure 3. Evaluation of different classifiers using AUC (A) and P@20 (B) measures to predict anomalous nodes based on the meta-features.

ods). Yet, WGAND had a larger and more significant overlap. We extended this analysis to include proteins that were involved in processes that were not necessarily tissue-specific but rather preferentially active in the respective tissue by using ProAct scores [19]. Notably, the top-10 anomalous proteins, according to WGAND were also more likely involved in such processes relative to their score in other tissues (Fig 5C; $p-value = 2.5e-16$, Mann-Whitney test). Lastly, we compared WGAND ensemble to three other WGAND methods, including feature's mean, IForest, and PCA. WGAND ensemble performed best in all tests (Fig. 5D). We also checked whether the results were specific to the top-10 proteins by checking for the top-5, top-15, and top-20 proteins. In all cases, WGAND performed better that all other methods (Fig. S4).

To demonstrate the value of our method, we describe tissue-relevant proteins that were indeed ranked as anomalous by "WGAND (ensemble)" in the respective tissue, supporting our hypothesis. Notably, these proteins were not part of the positive set used to train WGAND. TNNT2 and TNNT3 are members of the troponin protein complex that initiates the process of muscle contraction. Both proteins encode the troponin T subunit of the complex. TNNT2 encodes the cardiac type of troponin T and was indeed ranked by WGAND as the 7th and 6th most anomalous node in the heart atrial appendage and left ventricle tissues, respectively. TNNT3 encodes the fast skeletal muscle type of troponin T and was indeed ranked by WGAND as the topmost anomalous node in skeletal muscle. Additionally, each of these proteins is associated with a disease affecting the anomaly-relevant tissue: TNNT2 is involved in cardiomyopathy (OMIM #191045, [26]), whereas TNNT3 is involved in the Sheldon-Hall syndrome. This disorder affects muscle and skeletal development before birth [27, 28]. Another example is the BGN protein, ranked third by WGAND in artery aorta tissue. BGN is associated with a rare genetic disorder called Meester-Loeys syndrome that is characterized by early-onset aortic aneurysm and dissection, leading to compromised structural integrity of the aorta and other large arteries [29]. Thus, the predicted anomalous state could indicate proteins involved in diseases and biological processes affecting the respective tissues.

Discussion

We presented a novel machine learning method for detecting abnormal nodes in weighted networks with positive and negative edge weights, which has important implications for various domains. Here, we demonstrated the effectiveness of our approach by applying it to PPI networks of different human tissues to identify anomalous proteins. By anomalous proteins, we refer not only to proteins associated with human diseases that manifest in specific tissues but also to proteins with major tissue-specific biological roles. Knowledge of such proteins could illuminate the molecular basis of human diseases, tissue phenotypes, and tissue-specific physiological processes. These goals were partially met by previous methods that relied on tissue transcriptomes [19], tissue-relevant networks [18], or methods that combine both, often using machine learning [7, 17]. Yet, the concept of network anomaly

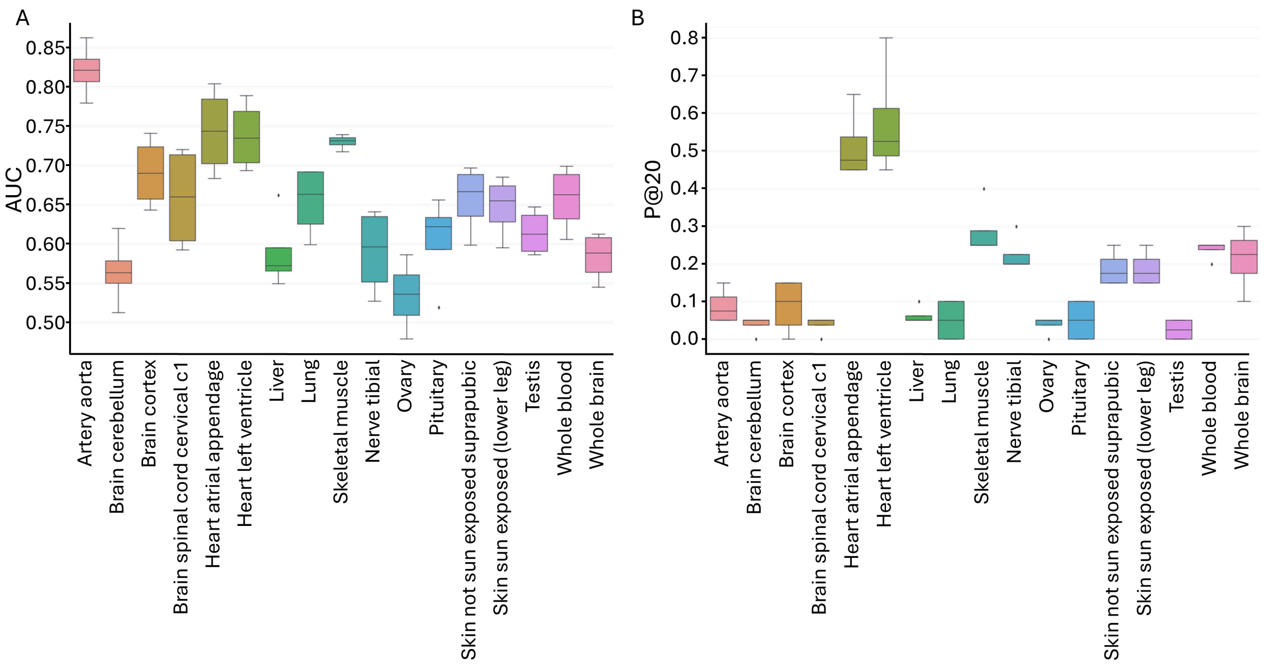

Figure 4. Evaluation of the variance of the four methods for predicting anomalous nodes for each tissue. We can observe that most tissues exhibit high variance in performance between different methods.

has not been used for this purpose.

We first demonstrated the relevance of network anomaly by showing that estimation errors of edge weights could be used to identify tissue-associated disease proteins (Fig. 2). Next, we took this approach further by devising a machine-learning method, WGAND, which was trained on features derived from estimation errors to predict anomalous proteins per tissue. Lastly, we used the predicted anomaly scores to rank anomalous proteins. We showed that the top-10 anomalous proteins overlapped significantly with proteins associated with diseases or phenotypes that affect the anomaly-relevant tissues, or with proteins with major tissue-specific roles. Moreover, WGAND outperformed other methods in detecting tissue-associated disease proteins and tissue-relevant proteins (Fig. 5). One caveat in our analyses is that training data of disease- or tissue-associated proteins is incomplete. This implies that performance could be improved once more training data is available. It also implies that anomaly prediction scores could be used to pinpoint promising candidates for further analysis. Another potential bias in our analysis is that disease-associated proteins were more likely to be extensively studied than other proteins. Hence, their node degrees in our networks tend to be higher, contributing to their anomaly scores [30]. We controlled for this bias in two ways. Firstly, all tissue networks had the same topology and differed only in edge weights derived from unbiased transcriptomic data. Since anomaly scores were tissue-specific, they were not simply affected by differences in protein degrees between disease-associated proteins and other proteins. Secondly, we showed that proteins with major tissue-specific roles beyond disease-association were more likely to be an anomaly in the relevant tissue network. Specifically, we applied WGAND to predict tissue-relevant proteins participating in tissue-specific processes. These were derived either via manual curation or via an unbiased analysis of processes that were preferentially active in specific tissues based on transcriptomic data. Anomaly scores were assessed for each category. We found that high-ranking anomalous proteins were enriched not only for disease-associated proteins (Fig. 5A), but also for proteins involved in tissue-specific processes (Fig. 5B) and tissue preferentially active processes (Fig. 5C). Finally, we compared our results to FUGUE, which had the same goal as WGAND of predicting tissue-relevant proteins going beyond disease-associated proteins. We found that WGAND performed better than FUGUE (Fig. 5A,B).

We evaluated WGAND on weighted networks where edge weights can be positive and negative. WGAND can also be applied to other types of weighted networks, such as those with only positive or negative values. Moreover, WGAND can also be used for unweighted networks by converting the networks' weights into binary values, where the existing edge will have a weight of 1 and the non-existing edge will have a weight of 0. WGAND could generate anomaly scores for all of these cases. We hope to evaluate this in future studies by analyzing additional types of networks.

We evaluate WGAND's performance using AUC, P@K, and PR-AUC. When dealing with imbalanced data, P@K is critical, as a model with higher P@K can help experts narrow

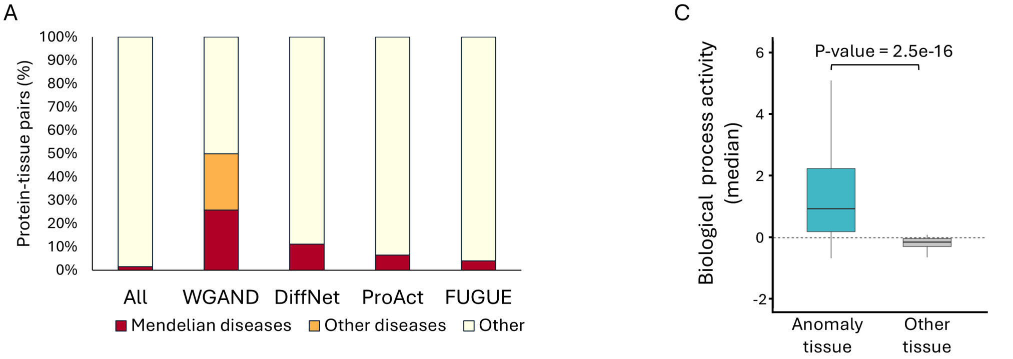

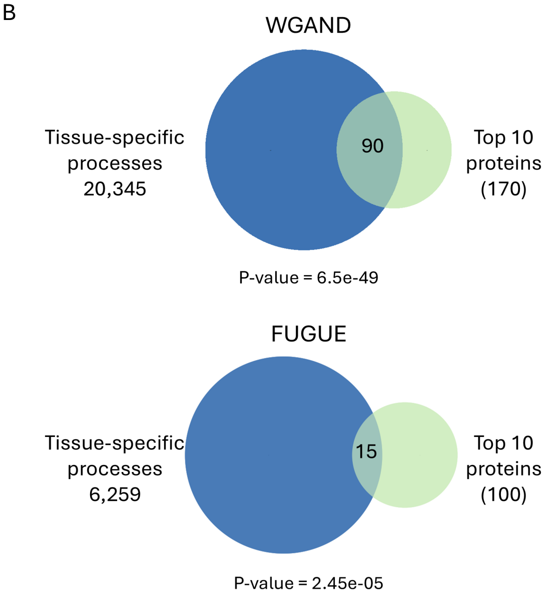

D

Method	Disease-related pairs (%)	Tissue-associated disease proteins (#)	Tissue-specific process' proteins (p-value)	Biological process activity (p-value)
Features' mean	22%	81	9.95e-40	3.65e-10
iForest	19%	87	9.2e-46	2.66e-14
PCA	19%	77	6.5e-36	1.03e-09

Figure 5. Top-10 anomalous proteins were enriched for tissue-specific diseases and cellular processes. (a) The percentage of protein-tissue pairs where the protein was associated with a Mendelian disease affecting that tissue (red). The percentage of such pairs out of all possible pairs was 1.5% (All), which was lower than their percentage among the top-10 pairs ranked by WGAND (ensemble) (26%), DiffNet (11%), ProAct (6%), and FUGUE (4%). The top-10 pairs ranked by WGAND were also enriched for proteins involved in other tissue-selective diseases and phenotypes (24%, orange). (b) The overlap between the top-10 protein-tissue pairs and pairs where the protein was associated with a biological process specific to that tissue. The number of such pairs appears in parentheses. Pairs ranked by WGAND were more significantly enriched than pairs ranked by FUGUE (Fisher exact test). (c) Top-10 proteins per tissue were more likely associated with cellular processes that were preferentially active in that tissue (blue) relative to other tissues (grey; p-value = $2.5e-16$, Mann-Whitney). (d) Analyses in panels (a-c) were applied to the top-10 anomalous proteins detected by three other WGAND methods, including features' mean, IForest, and PCA. The results were significant yet weaker than the ensemble method.

down the search space when inspecting anomalies. While the differences between various node embedding models are not highly significant, it is possible that the performance differences could be more pronounced in detecting anomalous nodes in different types of networks, such as social networks or computer networks. Additionally, the performance of the method may be improved by using a more systematic way to select the best node embedding algorithm for a given task. Moreover, using hyperparameter optimization tuning and using frameworks like Optuna [31] can also improve the algorithm's performance. We plan to investigate this further in future studies by analyzing additional node embedding models and networks.

We found that the WGAND ensemble performs significantly better than the baseline models in most tissues. OddBall outperformed WGAND in only four brain-related networks in terms of P@K and one network in terms of PR-AUC, but performed worse in terms of AUC (Table S1). These results suggest that biological differences between brain tissues and other tissues may be a contributing factor to the observed performance differences. In future studies, one could explore the commonalities and differences between brain-related tissues further. We also noted that Node2Vec with IForest could not achieve good results with this data in all the tissues, suggesting they was not suited for the task.

We showed that a single meta-feature can be a good measure for node anomaly detection. On average, the Sum of Errors is the best predictive single feature across all metrics. Surprisingly, the Absolute Sum of Errors is the worst feature across all metrics, despite our expectation that the error direction should not matter and that a higher total error should be a good indication of an anomaly. This result suggests that error direction is an important factor in node anomaly detection and that the difference between regular and absolute meta-features is inconsistent. In a future study, we would like to evaluate the WGAND on different use cases, such as malicious user detection in online social networks. We would like to explore if the same type of anomaly detection features would continue to give the best performance in different domains.

Lastly, we investigated different methods for generating anomaly scores and extracting anomalies from the generated anomaly features and found that some methods have similar performance. Among the four proposed methods, the IForest-based solution performs the worst in terms of AUC, while Feature's Mean has the best AUC. In terms of P@K, the differences are smaller, but different methods have advantages in different tissue networks. For example, the ensemble-based method has a significant advantage in the liver network, while the PCA-based and Feature's Mean methods have a significant advantage in the lung network. Also, some tissues, such as Skin Not Sun Exposed Suprapubic, Heart Left Ventricle, and Nerve Tibial had higher P@K when learning from a single feature than all of the features. While it is difficult to conclude which method is the best, a good rule of thumb would be first to try using the ensemble-based WGAND, which should combine the advantages of all other methods.

Currently, our search for anomalous nodes is limited to proteins with known PPIs. Future applications could extend the search for other types of genes, for example, by using coexpression networks [32] and to additional contexts, such as cell types [33].

Methods and Experiments

In this section, we present the components of our generic anomaly detection framework and detail how its performance was tested and evaluated. We then describe the dataset of weighted, tissue-specific protein-protein interaction (PPI) networks utilized in this study. Following this, we explain the process of annotating tissue-associated disease proteins and tissue-specific biological processes. Next, we outline how the framework was employed to evaluate edge weight predictions based on their ability to identify tissue-associated disease proteins and how it was applied to detect anomalous nodes with tissue-specific roles. Lastly, we conclude the section by comparing the (RRID:SCR_026406) in identifying tissue-associated disease proteins proteins with tissue-specific impacts to other tissue-specific ranking methods.

Anomaly Detection Generic Framework

Precisely defining what constitutes an anomaly is challenging [34], as definitions can vary across different domains and disciplines. Generally, anomalies are considered unusual observations that deviate significantly from the general population to the extent that they suggest a different mechanism may have created them [13]. In this study, we treated an anomaly as a node that behaves differently from most of the nodes in the network and interacts differently with its neighbors.

To formalize this idea, let us consider a weighted graph $G = (V, E, W)$, where V is the set of vertices, E is the set of edges, and W is the set of weights. Given a vertex v, we define its neighborhood $\Gamma(v) = \{(v,u) \mid (v,u) \in E\}$ as the set of vertices that are connected to v by an edge, and their weights as $W_v = \{W_{v,u} \mid u \in \Gamma(v)\}$, where W_a and W_n represent the edge weights between a suspected anomalous node a with its neighbors and a normal node n with its neighbors, respectively.

Extending our recent work [15], we hypothesized that W_a should deviate from W_n if a is truly anomalous. In other words, the average edge weights for a should differ from those of n. To test this hypothesis, we assumed that a general predictive function $P_f(v,u)$ exists, which can estimate the weight of an edge (v,u) with high accuracy. Given this assumption, we expect that $|\hat{W}_{a,v} - W_{a,v}| > |\hat{W}_{n,v} - W_{n,v}|$ if v is a neighbor of a and a is genuinely anomalous. This is because the predicted weight $\hat{W}_{a,v}$ for the edge (a,v) should deviate considerably from the actual weight $W_{a,v}$ if a is anomalous. In contrast, the predicted weight $\hat{W}_{n,v}$ for the edge (n,v) should be close to the actual weight $W_{n,v}$ if n is normal.

Since most nodes and their edges are normal, it should be possible to train a model that learns to predict the values

of normal interactions. A model that will learn to generalize well should successfully predict the normal values and fail to predict abnormal values, resulting in higher errors for these cases.

Development of an Edge Weight Estimator

We trained a model that estimates edge weights to create the predictive function $P_f(v,u)$. We used node embedding to generate features representing nodes v and u to train $P_f(v,u)$. Node embedding is a vector representation of nodes in a graph designed to capture the relationships between nodes [35]. Unlike structure-based feature sets that may be specific to certain types of networks, node embedding is a more general solution that can produce near-state-of-the-art performance without requiring additional handcrafted features.

We treated the network as an unweighted graph to generate the embedding vectors.[1] In this study, to select the node embedding method, we evaluated five types of embeddings: node2vec [36], RandNE [23], GLEE [37], NodeSketch [38], and DeepWalk [39, 40] based on the implementation by Rozemberczki et al. [40].

Using the extracted embeddings, we can train a weight estimator by using various regression algorithms and measure their performances using a variety of metrics, such as Mean Absolute Error (MAE), Root Mean Square Error (RMSE), and MSE. In the study, we utilized three regressors: Random Forest [24], XGBoost (RRID:SCR_021361) [41], and LightGBM (RRID:SCR_021697) [42]. We measured the performance of the weight estimators using MSE. Also, it is essential to note that during the training of the weight estimator, it is crucial to avoid considerable overfit. In this study, to detect anomalous proteins, we tuned the models' parameters by limiting each tree's max depth to reduce overfitting. Suppose the model overfits and "memorizes" the edge values like an infinitely large decision tree. In that case, it will be unable to identify nodes that deviate from the norm, as there will be no deviations in the predictions.

Construction of Anomaly Detection Features

Anomaly detection features are used to identify nodes in the network that behave differently from the norm. Features are constructed to represent a node by aggregating the difference between the estimated and the actual edge weight of its edges. An anomalous node is likelier to have a larger average error, indicating that it behaves differently. The accumulation suggests that the deviation is not a result of the model error but something systematic.

Deviation from the norm can be measured in a variety of ways. Similarly to Kagan et al. [15], we have generated anomaly detection features based on basic statistics such as standard deviation, mean, etc. To create the anomaly detection features, we defined the error of an edge (u,v) as $e_{u,v} = \hat{W}_{u,v} - W_{u,v}$ and the error of a node v as $e_v = \{e_{u,v} \mid u \in \Gamma(v)\}$. Based on these scores, we calculated ten meta-features for each node in the network:

1. **Mean Error** - The average error of the predicted weights of the interactions between node v and its neighbors: $ME(v) := \frac{\sum_{\{i\in\Gamma(v)\}} e_{v,i}}{|\Gamma(v)|}$.
2. **Error Standard Deviation** (Error SD) - The dispersion of the predicted weights of the interactions between node v and its neighbors: $\sigma(e_v)$.
3. **Median Error** - The median error of the predicted weights of the interactions between node v and its neighbors: $median(e_v)$.
4. **Sum of Errors** - The total sum of errors of the predicted weights of the interactions between node v and its neighbors: $\sum_{\{i\in\Gamma(v)\}} e_i$.
5. **Standard Error of the Mean (SEM)** - Measures the average amount of error variation of the predicted weights of the interactions between node v and its neighbors: $\frac{\sigma(e_v)}{\sqrt{n}}$.
6. **Mean Absolute Error** - The average absolute error of the predicted weights of the interactions between node v and its neighbors: $MAE(v) := \frac{\sum_{\{i\in\Gamma(v)\}} |e_{v,i}|}{|\Gamma(v)|}$.
7. **Absolute Error Standard Deviation** - The dispersion of the absolute errors of the predicted weights of the interactions between node v and its neighbors $\sigma(\mid e_v \mid)$.
8. **Median Absolute Error** - The absolute median error of the predicted weights of the interactions between node v and its neighbors: $median(\mid e_v \mid)$.
9. **Sum of Absolute Errors** - The sum of absolute errors of the predicted weights of the interactions between node v and its neighbors: $\sum_{i\in\Gamma(v)} \mid e_i \mid$.
10. **Standard Absolute Error of the Mean** - Measures the average amount of absolute error variation of the predicted weights of the interactions between node v and its neighbors: $\frac{\sigma(|e_v|)}{\sqrt{n}}$.

Using these features, we generate a ten-dimension vector for each node. In this study, to inspect the potential of the proposed features in predicting anomalies in PPI networks, we extracted the meta-features of the differential networks and treated them as a ten-dimension vector. Then, we used Principal Component Analysis (PCA) to reduce the meta-features into a two-dimensional vector. The PCA analysis revealed that the tissue-associated disease proteins are grouped together. This result indicates that the meta-features successfully identified proteins with major biological roles in a specific tissue and these proteins behave as anomalies (Fig. S3).

[1] Some node embedding algorithms, such as node2vec [36], use the edge weights when building the embedding. Using embedding created from a weighted graph to train an edge weight estimator creates a data leakage that will affect the whole system's performance.

Table 1. Evaluation of different node embedding algorithms.

Embedding	AUC	PR-AUC	P@1	P@3	P@10	P@20	Embedding Runtime (Sec)
DeepWalk	0.6629	0.0528	0.4118	0.2353	0.1941	0.1588	96
GLEE	0.6699	0.0417	0.3529	0.2549	0.1765	0.1412	4
Node2Vec	0.6658	0.0565	0.4118	0.2745	0.2412	0.1824	2912
NodeSketch	0.6700	0.0569	0.4118	0.3137	0.2471	0.1941	229
RandNE	**0.6701**	**0.06155**	**0.5294**	**0.3725**	**0.2529**	**0.2147**	**1.6**

Table 2. Edge weight estimator performance based on different models.

Weight Estimator	MSE
LightGBM	**0.0016**
RandomForest	0.0029
XGBoost	0.0020

Table 3. Average metrics for different weight estimators base models when used to detect anomalous nodes.

Weight Estimator	AUC	PR-AUC	P@1	P@3	P@10	P@20
LightGBM	0.64	0.03	0.18	0.14	0.12	0.08
RandomForest	**0.67**	**0.06**	**0.53**	**0.37**	**0.25**	**0.21**
XGBoost	0.60	0.01	0.00	0.00	0.01	0.02

Anomalous Nodes Detection

The main approach to search for anomalies is by ranking the nodes according to a specific anomaly detection feature, where nodes with the highest values are considered potential anomalies. However, determining the most suitable feature among the presented anomaly detection features can be challenging. To address this, we propose a variety of methods can be four methods combining the generated anomaly detection features into one anomaly score.

1. **Feature's Mean** - uses a sigmoid function to normalize the values of each anomaly detection feature, then assigns each node with the mean normalized values of the features. This process produces a single value that can be used as the anomaly score.

2. **PCA** - reduces the dimensionality of the anomaly detection features. The resulting component value can be used as the anomaly score [43].

3. **Isolation Forest (IForest)** - uses anomaly detection features as the IForest [44] model input to generate an anomaly score.

4. **Ensemble** - combines the previous three methods. Here, the raw Feature's Mean, PCA values, and also their values after a sigmoid function were inserted into an IForest model to generate the anomaly score. Then, we select the max value from this new anomaly score and the scores produced from the first two methods.

Evaluation Scheme

To assess the effectiveness of our proposed method and evaluate anomaly detection performance, a variety of metrics can be used, such as the models' AUC [45], $PR-AUC$ [46], and $P@K$, where k refers to the number of top-ranked records (nodes) to consider. AUC and PR-AUC are commonly used metrics to estimate the overall performance of a classifier [45, 46]. $P@K$ is particularly useful in anomaly detection tasks, where the goal is to discover new items and recommend and prioritize a subset of predictions with the highest confidence scores.

To evaluate our method in identifying abnormal nodes in PPI networks, in this study, we utilized the AUC, $PR-AUC$, and $P@K$ metrics. Based on these metrics, we first compared the performance of five different node embedding models on weighted networks. Secondly, using the best-performing embedding model, we compared the performance of three edge weight estimator models, where their performance was also measured by MSE. Thirdly, we used the best-performing combination of node embedding and edge weight estimator models and evaluated the performance of four different anomaly detection methods.

To evaluate the performance of the proposed method on the PPI networks, and compare it to other node anomaly detection methods, we used two baseline methods:

1. **Node2Vec + IForest** - Similarly to Lee et al. [12], we used node2vec combined with the IForest model. Here, we generated the embeddings based on the weights of the graph edges via node2vec. Then, we utilized these embeddings as the features for the IForest model.

2. **OddBall** - We used the OddBall algorithm [13] for anomaly detection in weighted graphs. To the best of our knowledge, OddBall is the only available algorithm with an implementation[2] that was specifically designed for anomaly detection in weighted graphs.

Dataset of Weighted Tissue-Specific PPI Networks

In this study, we tested and evaluated our method on data of 17 PPIs networks were downloaded from [47] (Table 4). Each network was composed of 13,523 proteins (nodes) and 134,223 PPIs (edges). The weight of an interaction relied on the expression of the interacting genes in the given tissue relative to their expression in other tissues [47]. Weights

[2] https://github.com/gloooryyt/oddball_py3

ranged between $[-1,1]$, where positive scores imply that the protein interaction was more likely to occur in that tissue relative to other tissues, and negative scores imply that it is less likely to occur.

Table 4. PPI Networks Datasets

Tissue	#Tissue-Associated Disease-Proteins nodes (%)
Artery Aorta	23 (0.17)
Brain Cerebellum	65 (0.48)
Brain Cortex	77 (0.57)
Brain Spinal cord cervical c 1	48 (0.35)
Heart Atrial Appendage	133 (0.98)
Heart Left Ventricle	138 (1.02)
Liver	53 (0.39)
Lung	274 (2.03)
Muscle Skeletal	121 (0.89)
Nerve Tibial	77 (0.57)
Ovary	41 (0.3)
Pituitary	23 (0.17)
Skin Not Sun Exposed Suprapubic	125 (0.92)
Skin Sun Exposed Lower leg	125 (0.92)
Testis	101 (0.75)
Whole Blood	442 (3.3)
Whole Brain	564 (4.2)

Annotation of Tissue-Associated Disease Proteins and Tissue-specific Biological Processes

Data of Mendelian diseases, disease proteins, and disease-affected tissues were obtained from ODiseA [25]. Nodes of a given tissue PPI network were labeled as tissue-associated disease proteins if the corresponding protein was associated with a Mendelian disease that affected that tissue. As shown in Table 4, up to 4.2% of the nodes per network were labeled as tissue-associated disease proteins. Data of proteins involved in additional tissue-selective diseases and phenotypes were obtained from PUB-MED [48], GeneCards [49], and HPO [50] databases via manual curation. Data of proteins participating in tissue-specific biological processes were obtained from Basha et al. [47].

Protein Anomaly Detection Model

We tested whether the anomaly detection algorithm could be used to identify proteins with major biological roles in the tissue where they appear anomalous. We considered a protein to have major biological roles in the tissue if it was (i) designated as tissue-associated disease protein in that tissue according to ODiseA [25]; (ii) was involved in an additional tissue-selective disease or phenotype, according to manual search; (iii) participated in a biological process that was specific to that tissue according to [47]; or (iv) participated in a biological process that was more active in that tissue relative to other tissues, as estimated by ProAct [19]. We applied WGAND to each tissue-specific PPI network and focused on the top-10 anomalous nodes per network. To test for (i), we compared the fraction of tissue-associated disease proteins among the top-10 anomalous nodes relative to that fraction among all other nodes. To test for (ii), we performed a manual search. To test for (iii), we computed the fraction of top-10 proteins that participated in a tissue-specific biological process out of the total number of proteins participating in such processes in the given tissue and assessed for enrichment using the Fisher exact test. To test for (iv), we compared the estimated biological process activities of the top-10 anomalous proteins in the given tissue to their estimated activities in all other tissues using the Mann-Whitney test.

Comparison of the WGAND Anomaly Detection Method to Other Methods

We compared the performance of WGAND in revealing tissue-associated disease proteins that affect a specific tissue to three other tissue-specific ranking schemes. Firstly, we compared WGAND with a ranking of proteins by DiffNet [18]. Specifically, per tissue network, we associated each protein with the median differential score of its interactions and then considered the top-10 proteins with the highest score. Secondly, we compared WGAND with a ranking of proteins by ProAct [19]. Specifically, per tissue, we associated each protein with median ProAct score of the processes it participates in, and then considered the top-10 proteins with the highest score. Thirdly, we compared WGAND with the top-10 tissue-relevant proteins according to FUGUE [7]. We downloaded FUGUE scores from [7]. Scores were limited to proteins and tissues, that WGAND also scored, resulting in ten tissues that were common to both methods. Per method, we computed per tissue the fraction of tissue-associated disease proteins out of the top-10 proteins that were associated with a Mendelian disease that affected that tissue. For FUGUE, we also tested whether the top-10 tissue-relevant proteins per tissue participated in a biological process specific to that tissue, as described in the preceding subsection. All tests were repeated for the top 5,15, and 20 proteins per tissue (Fig. S4).

Data Availability

The following datasets and algorithms were utilized in this study: DiffNet [18], ProAct [19], TRACE [17], ODiseA [25], and FUGUE [7]. Supporting data for this study can be found in the GigaScience GigaDB [51].

Availability of Source Code and Requirements

**Project name**	WGAND (RRID:SCR_026406)
**Project homepage**	`https://github.com/data4goodlab/wgand`
**Operating system(s)**	Platform independent
**Programming language**	Python 3.6 or higher
**Other requirements**	pandas, combo, tqdm, pyod, scikit-learn, numpy, networkx, karateclub
**License**	GPL 3.0
**Reference**	Version 1 [21]

Abbreviations

WGAND: Weighted Graph Anomalous Node Detection;
PPI: protein-protein interaction;
AUC: area under curve;
P@K: precious at k;
MSE: mean squared error;
SD: standard deviation;
SEM: Standard error of the mean;
MAE: mean absolute error;
PCA: Principal Component Analysis;
IForest: Isolation Foresrt;

Acknowledgements

J.J. wishes to thank the Baroness Ariane de Rothchild Women Doctoral Program. Additionally, during the drafting of this article, we utilized ChatGPT and Grammarly for editing purposes, ensuring the text's grammar, spelling, and clarity were thoroughly checked and improved as necessary.

Funding

This study was funded by the Israeli Council for Higher Education (CHE) via the Data Science Research Center, Ben-Gurion University of the Negev, Israel [to M.F. and E.Y.-L], and by the Israel Science Foundation [401/22 to E.Y.-L.].

Authors' contributions

D.K., J.J., E.Y.-L., and M.F. conceived the study; D.K. developed the code framework; J. J. and D. K. analyzed the data; E.Y.-L. and M.F. supervised the study; and all authors wrote the paper.

Competing Interests

The authors declare that they have no competing interests.

References

[1] Lihua Jiang, Meng Wang, Shin Lin, Ruiqi Jian, Xiao Li, Joanne Chan, Guanlan Dong, Huaying Fang, Aaron E Robinson, François Aguet, et al. A quantitative proteome map of the human body. *Cell*, 183(1):269–283, 2020.

[2] GTEx Consortium. The gtex consortium atlas of genetic regulatory effects across human tissues. *Science*, 369(6509):1318–1330, 2020.

[3] Marc Vidal, Michael E Cusick, and Albert-László Barabási. Interactome networks and human disease. *Cell*, 144(6):986–998, 2011.

[4] Katja Luck, Dae-Kyum Kim, Luke Lambourne, Kerstin Spirohn, Bridget E Begg, Wenting Bian, Ruth Brignall, Tiziana Cafarelli, Francisco J Campos-Laborie, Benoit Charloteaux, et al. A reference map of the human binary protein interactome. *Nature*, 580(7803):402–408, 2020.

[5] Esti Yeger-Lotem and Roded Sharan. Human protein interaction networks across tissues and diseases. *Frontiers in genetics*, 6:257, 2015.

[6] Casey S Greene, Arjun Krishnan, Aaron K Wong, Emanuela Ricciotti, Rene A Zelaya, Daniel S Himmelstein, Ran Zhang, Boris M Hartmann, Elena Zaslavsky, Stuart C Sealfon, et al. Understanding multicellular function and disease with human tissue-specific networks. *Nature genetics*, 47(6):569–576, 2015.

[7] Gowthami Somepalli, Sarthak Sahoo, Arashdeep Singh, and Sridhar Hannenhalli. Prioritizing and characterizing functionally relevant genes across human tissues. *PLoS Computational Biology*, 17(7):e1009194, 2021.

[8] Frank E Grubbs. Procedures for detecting outlying observations in samples. *Technometrics*, 11(1):1–21, 1969.

[9] Ali Bou Nassif, Manar Abu Talib, Qassim Nasir, and Fatima Mohamad Dakalbab. Machine learning for anomaly detection: A systematic review. *Ieee Access*, 9:78658–78700, 2021.

[10] Leman Akoglu, Hanghang Tong, and Danai Koutra. Graph based anomaly detection and description: a survey. *Data mining and knowledge discovery*, 29:626–688, 2015.

[11] Tomer Michael-Pitschaze, Niv Cohen, Dan Ofer, Yedid Hoshen, and Michal Linial. Detecting anomalous proteins using deep representations. *NAR Genomics and Bioinformatics*, 6(1):lqae021, 2024.

[12] Meng-Chieh Lee, Hung T Nguyen, Dimitris Berberidis, Vincent S Tseng, and Leman Akoglu. Gawd: graph anomaly detection in weighted directed graph databases. In *Proceedings of the 2021 IEEE/ACM International Conference on Advances in Social Networks Analysis and Mining*, pages 143–150, 2021.

[13] Leman Akoglu, Mary McGlohon, and Christos Faloutsos. Oddball: Spotting anomalies in weighted graphs. In *Advances in Knowledge Discovery and Data Mining*, pages 410–421. Springer, 2010.

[14] Michael Davis, Weiru Liu, Paul Miller, and George Redpath. Detecting anomalies in graphs with numeric labels. In *Proceedings of the 20th ACM international conference on Information and knowledge management*, pages 1197–1202, 2011.

[15] Dima Kagan, Yuval Elovichi, and Michael Fire. Generic anomalous vertices detection utilizing a link prediction algorithm. *Social Network Analysis and Mining*, 8(1):1–13, 2018.

[16] Kasper Lage, Niclas Tue Hansen, E Olof Karlberg, Aron C Eklund, Francisco S Roque, Patricia K Donahoe, Zoltan Szallasi, Thomas Skøt Jensen, and Søren

Brunak. A large-scale analysis of tissue-specific pathology and gene expression of human disease genes and complexes. *Proceedings of the National Academy of Sciences*, 105(52):20870–20875, 2008.

[17] Eyal Simonovsky, Moran Sharon, Maya Ziv, Omry Mauer, Idan Hekselman, Juman Jubran, Ekaterina Vinogradov, Chanan M Argov, Omer Basha, Lior Kerber, et al. Predicting molecular mechanisms of hereditary diseases by using their tissue-selective manifestation. *Molecular Systems Biology*, 19.8: e11407, 2023.

[18] Omer Basha, Rotem Shpringer, Chanan M Argov, and Esti Yeger-Lotem. The DifferentialNet database of differential protein–protein interactions in human tissues. *Nucleic acids research*, 46(D1):D522–D526, 2018.

[19] Moran Sharon, Gil Gruber, Chanan M Argov, Miri Volozhinsky, and Esti Yeger-Lotem. Proact: quantifying the differential activity of biological processes in tissues, cells, and user-defined contexts. *Nucleic Acids Research*, 51(W1):W478–W483, 2023.

[20] Moran Sharon, Ekaterina Vinogradov, Chanan M Argov, Or Lazarescu, Yazeed Zoabi, Idan Hekselman, and Esti Yeger-Lotem. The differential activity of biological processes in tissues and cell subsets can illuminate disease-related processes and cell-type identities. *Bioinformatics*, 38(6):1584–1592, 2022.

[21] Dima Kagan, Juman Jubran, Esti Yeger-Lotem, and Michael Fire. WGAND - Weighted Graph Anomalous Node Detection (Version 1). `https://archive.softwareheritage.org/swh:1:snp:8b101aad101c594b30c1e194a0868e789d744cde`, 2025. [Computer software], Accessed: 2025-02-21.

[22] Nino Arsov and Georgina Mirceva. Network embedding: An overview. *arXiv preprint arXiv:1911.11726*, 2019.

[23] Ziwei Zhang, Peng Cui, Haoyang Li, Xiao Wang, and Wenwu Zhu. Billion-scale network embedding with iterative random projection. In *2018 IEEE International Conference on Data Mining (ICDM)*, pages 787–796. IEEE, 2018.

[24] Leo Breiman. Random forests. *Machine learning*, 45:5–32, 2001.

[25] Idan Hekselman, Lior Kerber, Maya Ziv, Gil Gruber, and Esti Yeger-Lotem. The organ-disease annotations (odisea) database of hereditary diseases and inflicted tissues. *Journal of Molecular Biology*, 434(11):167619, 2022.

[26] Justin H. Pham, John R. Giudicessi, Marysia S. Tweet, Lauren Boucher, D. Brian Newman, and Jeffrey B. Geske. Tale of two hearts: a tnnt2 hypertrophic cardiomyopathy case report. *Frontiers in Cardiovascular Medicine*, 10:1167256, 2023.

[27] Monica Rasmussen and Jian-Ping Jin. Troponin variants as markers of skeletal muscle health and diseases. *Frontiers in Physiology*, 12:747214, 2021.

[28] Sarah A. Sandaradura, Adam Bournazos, Amali Mallawaarachchi, Beryl B. Cummings, Leigh B. Waddell, Kristi J. Jones, Christopher Troedson, Annapurna Sudarsanam, Benjamin M. Nash, Gregory B. Peters, Elizabeth M. Algar, Daniel G. MacArthur, Kathryn N. North, Susan Brammah, Amanda Charlton, Nigel G. Laing, Meredith J. Wilson, Mark R. Davis, and Sandra T. Cooper. Nemaline myopathy and distal arthrogryposis associated with an autosomal recessive tnnt3 splice variant. *Human Mutation*, 39(3):383–388, 2018.

[29] Josephina A. N. Meester, Geert Vandeweyer, Isabel Pintelon, Martin Lammens, Lana Van Hoorick, Simon De Belder, Kathryn Waitzman, Luciana Young, Larry W. Markham, Julie Vogt, Julie Richer, Luc M. Beauchesne, Sheila Unger, Andrea Superti-Furga, Milan Prsa, Rami Dhillon, Edwin Reyniers, Harry C. Dietz, Wim Wuyts, Geert Mortier, Aline Verstraeten, Lut Van Laer, and Bart L. Loeys. Loss-of-function mutations in the x-linked biglycan gene cause a severe syndromic form of thoracic aortic aneurysms and dissections. *Genetics in Medicine*, 19(4):386–395, 2017.

[30] Michael Zietz, Daniel S Himmelstein, Kyle Kloster, Christopher Williams, Michael W Nagle, and Casey S Greene. The probability of edge existence due to node degree: a baseline for network-based predictions. *GigaScience*, 13:giae001, 2024.

[31] Takuya Akiba, Shotaro Sano, Toshihiko Yanase, Takeru Ohta, and Masanori Koyama. Optuna: A next-generation hyperparameter optimization framework. In *Proceedings of the 25th ACM SIGKDD international conference on knowledge discovery & data mining*, pages 2623–2631, 2019.

[32] Ashis Saha, Yungil Kim, Ariel DH Gewirtz, Brian Jo, Chuan Gao, Ian C McDowell, Barbara E Engelhardt, Alexis Battle, François Aguet, Kristin G Ardlie, et al. Co-expression networks reveal the tissue-specific regulation of transcription and splicing. *Genome research*, 27(11):1843–1858, 2017.

[33] The Tabula Sapiens Consortium*, Robert C Jones, Jim Karkanias, Mark A Krasnow, Angela Oliveira Pisco, Stephen R Quake, Julia Salzman, Nir Yosef, Bryan Bulthaup, Phillip Brown, et al. The tabula sapiens: A multiple-organ, single-cell transcriptomic atlas of humans. *Science*, 376(6594):eabl4896, 2022.

[34] Caleb C Noble and Diane J Cook. Graph-based anomaly detection. In *Proceedings of the ninth ACM SIGKDD international conference on Knowledge discovery and data mining*, pages 631–636. ACM, 2003.

[35] Martin Grohe. word2vec, node2vec, graph2vec, x2vec: Towards a theory of vector embeddings of structured data. In *Proceedings of the 39th ACM SIGMOD-SIGACT-SIGAI Symposium on Principles of Database Systems*, pages 1–16, 2020.

[36] Aditya Grover and Jure Leskovec. node2vec: Scalable feature learning for networks. In *Proceedings of the 22nd ACM SIGKDD international conference on Knowledge discovery and data mining*, pages 855–864, 2016.

[37] Leo Torres, Kevin S Chan, and Tina Eliassi-Rad. Glee: Geometric laplacian eigenmap embedding. *Journal of Complex Networks*, 8(2):cnaa007, 2020.

[38] Dingqi Yang, Paolo Rosso, Bin Li, and Philippe Cudre-Mauroux. Nodesketch: Highly-efficient graph embeddings via recursive sketching. In *Proceedings of the 25th ACM SIGKDD International Conference on Knowledge Discovery & Data Mining*, pages 1162–1172, 2019.

[39] Bryan Perozzi, Rami Al-Rfou, and Steven Skiena. Deepwalk: Online learning of social representations. In *Proceedings of the 20th ACM SIGKDD international conference on Knowledge discovery and data mining*, pages 701–710, 2014.

[40] Benedek Rozemberczki, Oliver Kiss, and Rik Sarkar. Karate Club: An API Oriented Open-source Python Framework for Unsupervised Learning on Graphs. In *Proceedings of the 29th ACM International Conference on Information and Knowledge Management (CIKM '20)*, page 3125–3132. ACM, 2020.

[41] Tianqi Chen and Carlos Guestrin. Xgboost: A scalable tree boosting system. In *Proceedings of the 22nd acm sigkdd international conference on knowledge discovery and data mining*, pages 785–794, 2016.

[42] Guolin Ke, Qi Meng, Thomas Finley, Taifeng Wang, Wei Chen, Weidong Ma, Qiwei Ye, and Tie-Yan Liu. Lightgbm: A highly efficient gradient boosting decision tree. *Advances in neural information processing systems*, 30, 2017.

[43] Charu C Aggarwal. *Outlier Analysis*, volume 1. Springer, 2015.

[44] Fei Tony Liu, Kai Ming Ting, and Zhi-Hua Zhou. Isolation forest. In *2008 eighth ieee international conference on data mining*, pages 413–422. IEEE, 2008.

[45] Tom Fawcett. An introduction to roc analysis. *Pattern recognition letters*, 27(8):861–874, 2006.

[46] Kendrick Boyd, Kevin H Eng, and C David Page. Area under the precision-recall curve: point estimates and confidence intervals. In *Machine Learning and Knowledge Discovery in Databases: European Conference, ECML PKDD 2013, Prague, Czech Republic, September 23-27, 2013, Proceedings, Part III 13*, pages 451–466. Springer, 2013.

[47] Omer Basha, Chanan M Argov, Raviv Artzy, Yazeed Zoabi, Idan Hekselman, Liad Alfandari, Vered Chalifa-Caspi, and Esti Yeger-Lotem. Differential network analysis of multiple human tissue interactomes highlights tissue-selective processes and genetic disorder genes. *Bioinformatics*, 36(9):2821–2828, 2020.

[48] Jacob White. Pubmed 2.0. *Medical reference services quarterly*, 39(4):382–387, 2020.

[49] Gil Stelzer, Naomi Rosen, Inbar Plaschkes, Shahar Zimmerman, Michal Twik, Simon Fishilevich, Tsippi Iny Stein, Ron Nudel, Iris Lieder, Yaron Mazor, et al. The genecards suite: from gene data mining to disease genome sequence analyses. *Current protocols in bioinformatics*, 54(1):1–30, 2016.

[50] Sebastian Köhler, Michael Gargano, Nicolas Matentzoglu, Leigh C Carmody, David Lewis-Smith, Nicole A Vasilevsky, Daniel Danis, Ganna Balagura, Gareth Baynam, Amy M Brower, et al. The human phenotype ontology in 2021. *Nucleic acids research*, 49(D1):D1207–D1217, 2021.

[51] Dima Kagan, Juman Jubran, Esti Yeger-Lotem, and Michael Fire. Supporting data for "Network-based anomaly detection algorithm reveals proteins with major roles in human tissues". `https://doi.org/10.5524/102669`, 2025. `[Data set], Accessed: 2025-02-21`.

Supplementary Materials

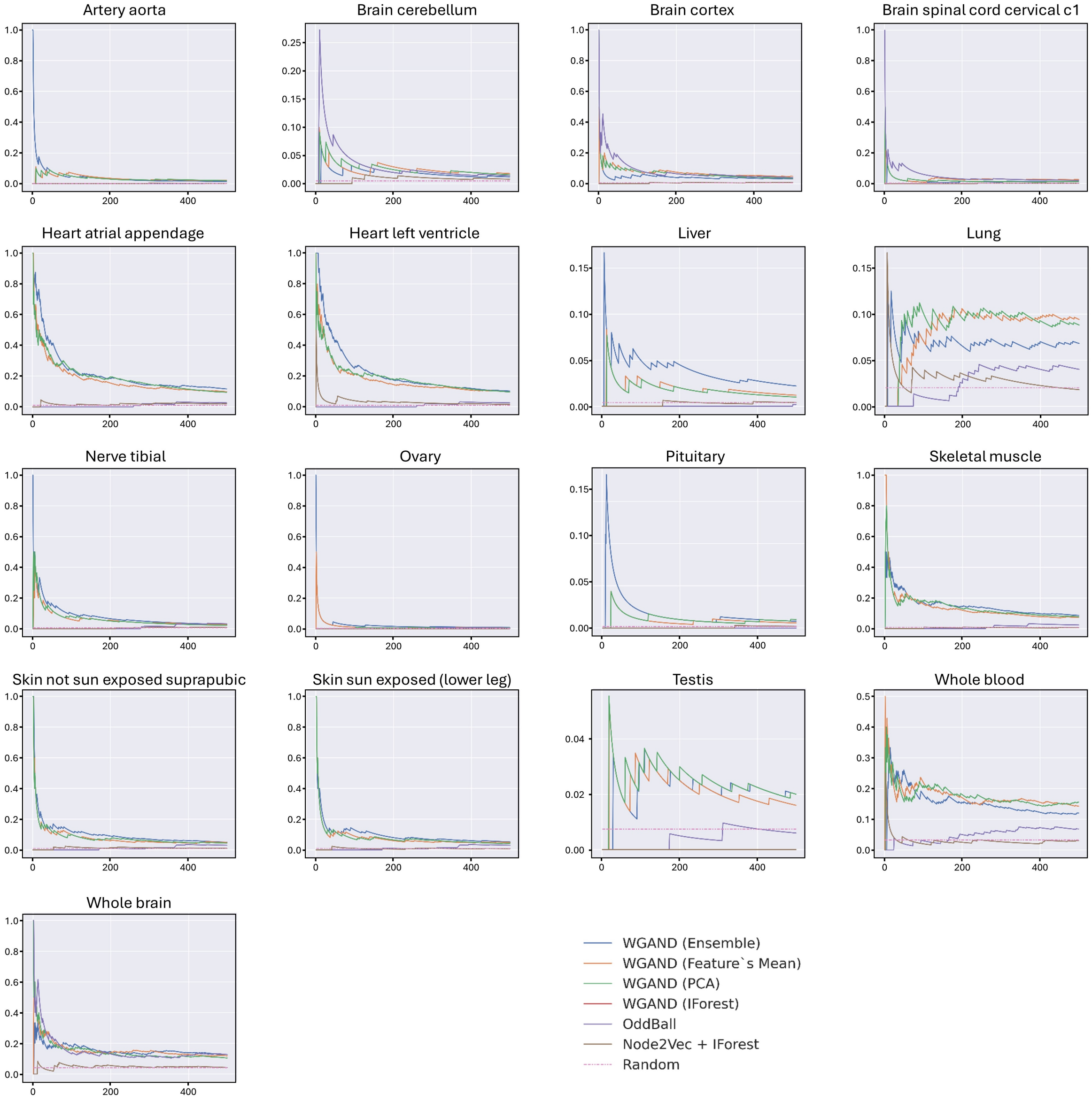

Figure S1. P@K of different classifiers per each PPI tissue-specific network.

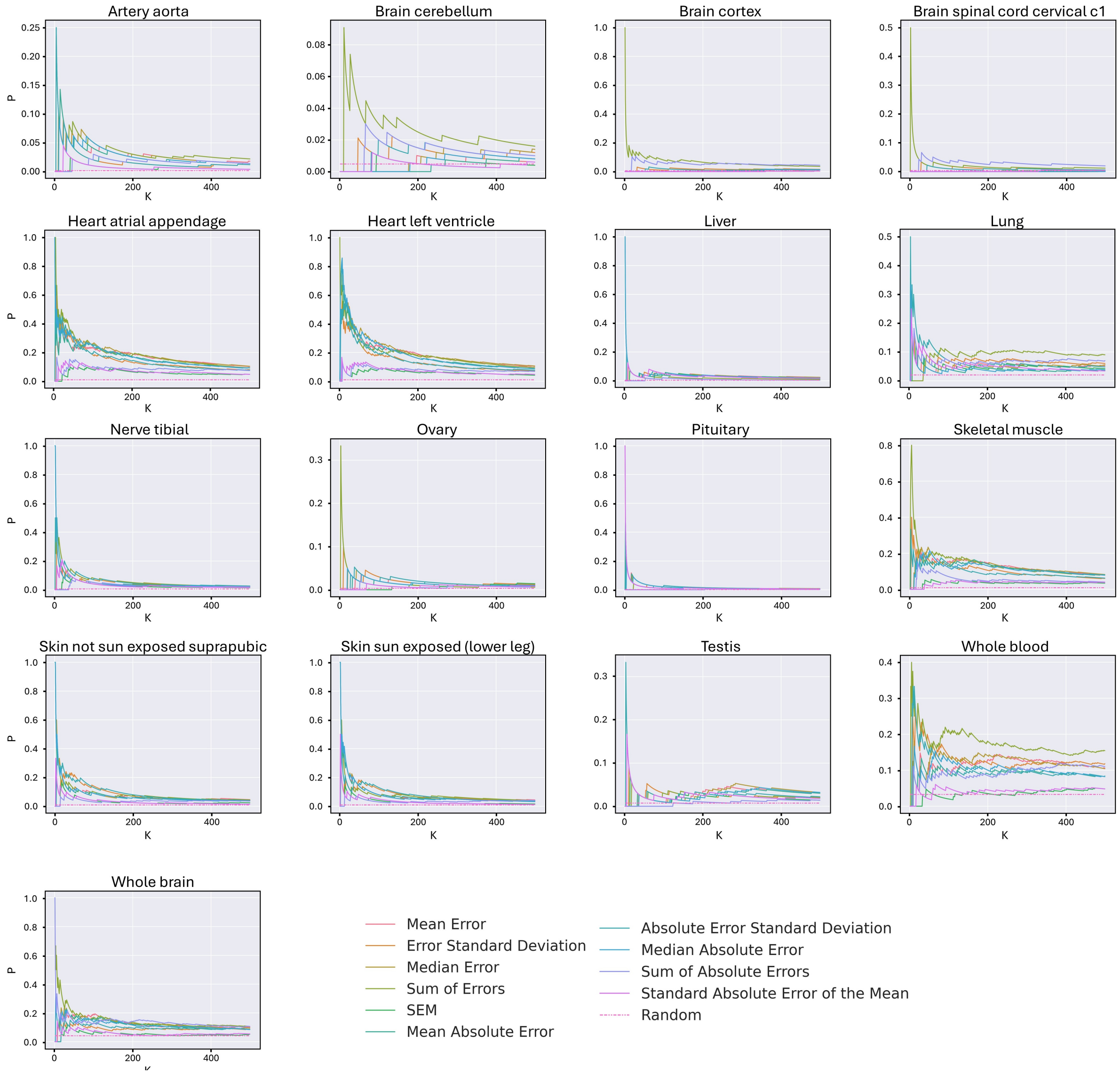

Figure S2. P@K of WGAND 'ensemble' method trained with each feature separately on PPI tissue-specific networks.

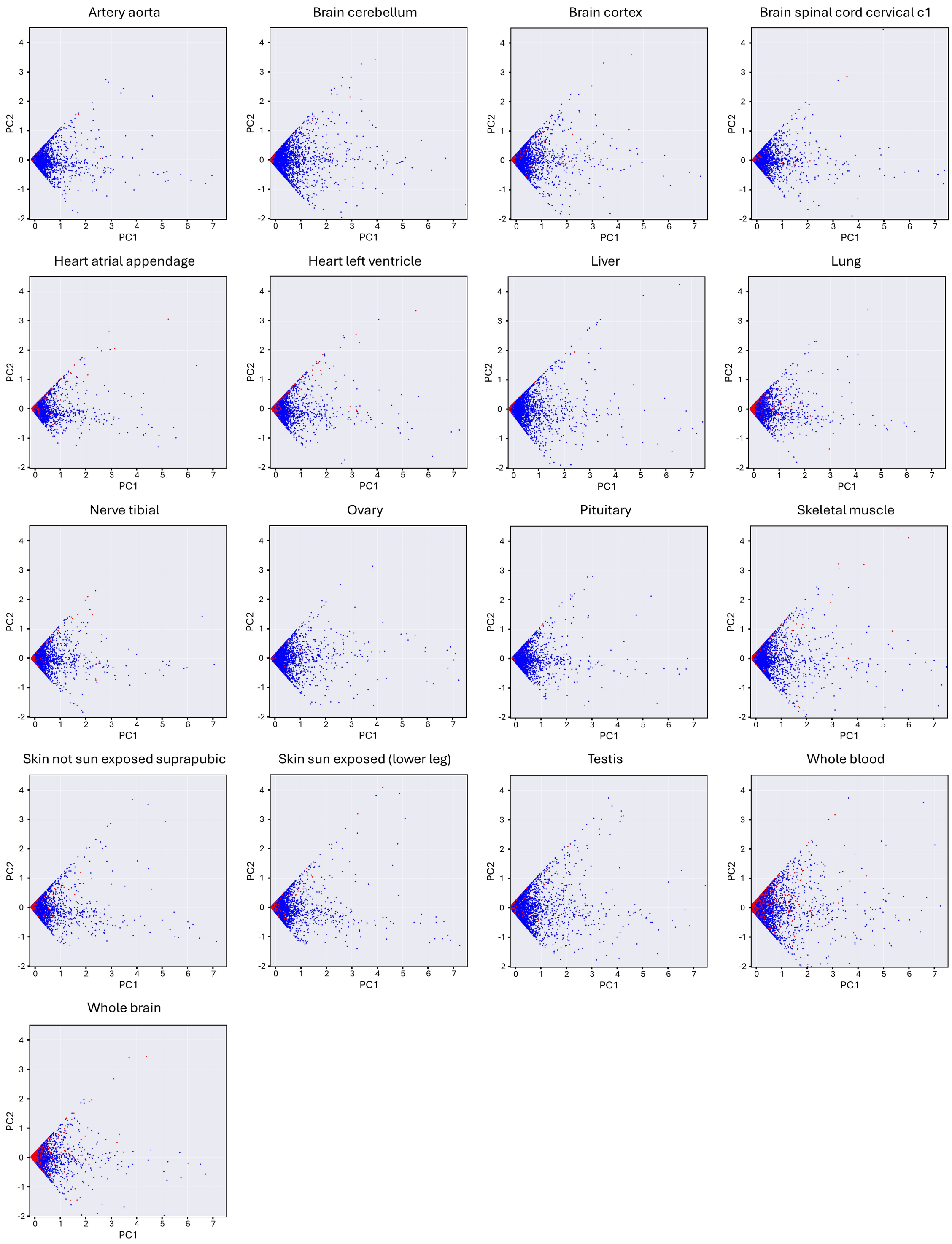

Figure S3. PCA representation of the anomaly detection features. The red dots represent tissue-associated disease proteins; the blue dots represent all other proteins.

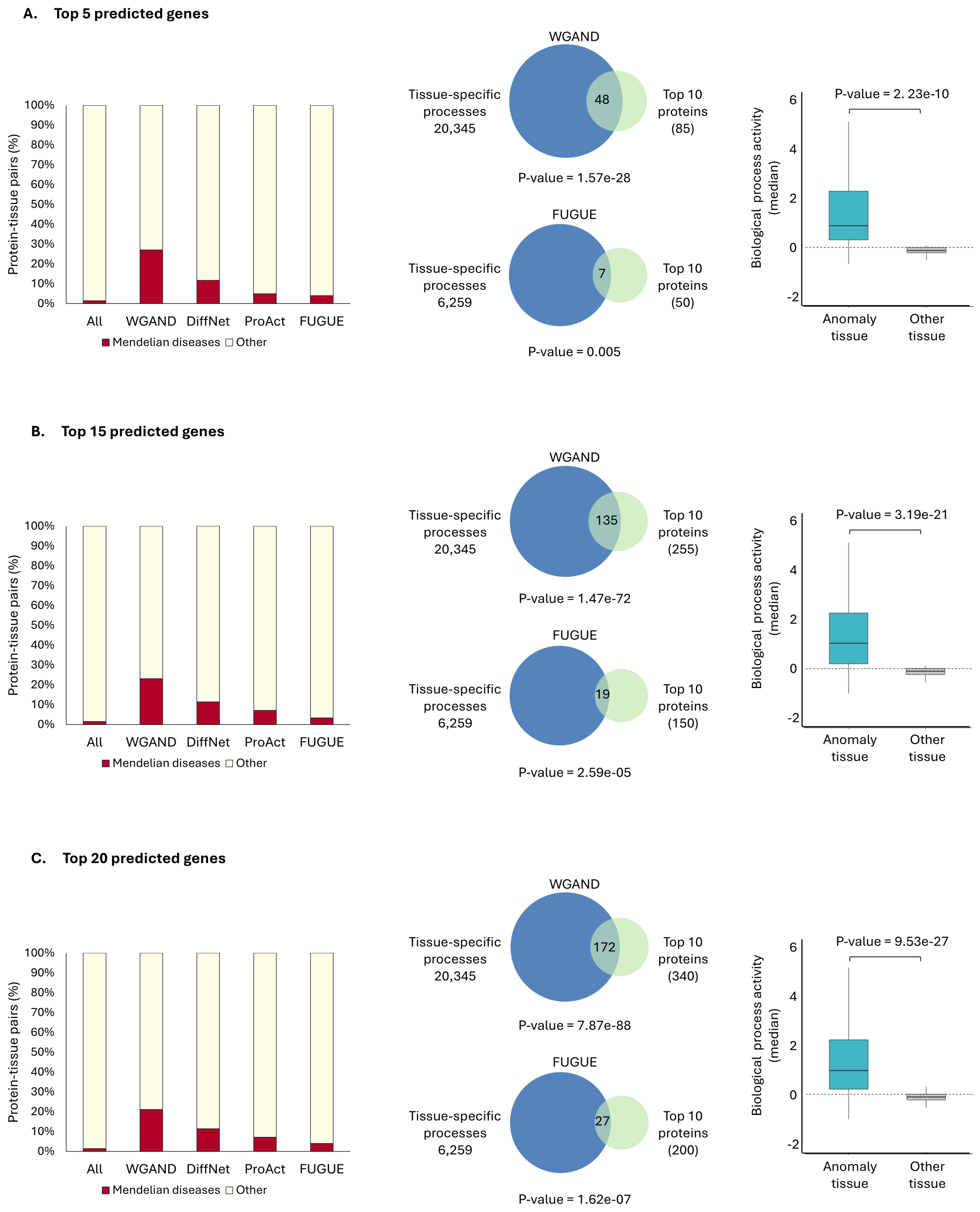

Method	Disease-related pairs (%)	Tissue-associated disease proteins (#)	Tissue-specific process' proteins (p-value)	Biological process activity (p-value)
Features' mean	31%	46	2.56e-26	6.53e-07
iForest	22%	44	3.47e-24	3.12e-8
PCA	28%	44	3.47e-24	5.08e-08

Method	Disease-related pairs (%)	Tissue-associated disease proteins (#)	Tissue-specific process' proteins (p-value)	Biological process activity (p-value)
Features' mean	19%	117	1.87e-54	2.42e-13
iForest	17%	124	3.12e-61	1.52e-20
PCA	17%	114	1.9e-51	4.05e-12

Method	Disease-related pairs (%)	Tissue-associated disease proteins (#)	Tissue-specific process' proteins (p-value)	Biological process activity (p-value)
Features' mean	15%	150	1.77e-66	1.62e-14
iForest	16%	162	8.11e-78	6.34e-26
PCA	16%	144	4.35e-61	1.11e-16

Figure S4. WGAND shows robust performance for the top-k proteins for different values of K. Top anomalous proteins show consistent enrichment for tissue-aware disease proteins, tissue-specific biological processes, and biological process activity in the anomaly-relevant tissue, as described in Fig. 5 of the main text for top-10 proteins. (a) Top-5 proteins. (b) Top-15 proteins, and (c) Top-20 proteins.

Table S1. Evaluation WGAND versus baselines on PPI dataset

Tissue Network	WGAND (Ensemble)						Node2Vec + Iforest						Oddball					
	AUC	PR-AUC	P@1	P@3	P@10	P@20	AUC	PR-AUC	P@1	P@3	P@10	P@20	AUC	PR-AUC	P@1	P@3	P@10	P@20
Artery Aorta	**0.83**	**0.11**	**1.00**	**0.67**	**0.20**	**0.15**	0.46	0.00	0.00	0.00	0.00	0.00	0.70	0.00	0.00	0.00	0.00	0.00
Brain Cerebellum	**0.56**	**0.01**	0.00	0.00	0.00	0.05	0.52	**0.01**	0.00	0.00	0.00	0.00	0.53	**0.01**	0.00	0.00	**0.20**	**0.15**
Brain Cortex	**0.72**	**0.02**	0.00	**0.33**	0.10	0.05	0.53	0.01	0.00	0.00	0.00	0.00	0.68	**0.05**	**1.00**	**0.33**	**0.40**	**0.25**
Brain Spinal cord cervical c 1	**0.71**	**0.01**	0.00	0.00	0.10	0.05	0.52	0.00	0.00	0.00	0.00	0.05	0.70	**0.04**	**1.00**	**0.33**	**0.20**	**0.10**
Heart Atrial Appendage	**0.78**	**0.18**	**1.00**	**1.00**	**0.70**	**0.65**	0.56	0.01	0.00	0.00	0.00	0.00	0.63	0.03	0.00	0.00	0.00	0.00
Heart Left Ventricle	**0.76**	**0.18**	**1.00**	**1.00**	**0.90**	**0.80**	0.54	0.02	0.00	0.00	0.00	0.00	0.62	0.03	0.00	0.00	0.00	0.00
Liver	**0.55**	**0.01**	0.00	0.00	0.00	**0.10**	0.42	0.00	0.00	0.00	0.00	0.00	0.41	0.00	0.00	0.00	0.00	0.00
Lung	**0.69**	**0.05**	**0.00**	**0.00**	**0.10**	**0.10**	0.52	0.02	0.00	0.00	0.10	0.05	0.60	0.03	0.00	0.00	0.00	0.00
Muscle Skeletal	**0.73**	**0.09**	**1.00**	**0.67**	**0.50**	**0.40**	0.54	0.01	0.00	0.00	0.00	0.00	0.56	0.02	0.00	0.00	0.00	0.00
Nerve Tibial	**0.63**	**0.05**	**1.00**	**0.33**	**0.30**	**0.30**	0.51	0.01	0.00	0.00	0.00	0.00	0.59	0.01	0.00	0.00	0.00	0.00
Ovary	**0.52**	**0.03**	**1.00**	**0.33**	**0.10**	**0.05**	0.51	0.00	0.00	0.00	0.00	0.00	0.52	0.00	0.00	0.00	0.00	0.00
Pituitary	**0.63**	**0.01**	**0.00**	**0.00**	**0.20**	**0.10**	0.52	0.00	0.00	0.00	0.00	0.00	0.53	0.00	0.00	0.00	0.00	0.00
Skin Not Sun Exposed Suprapubic	**0.69**	**0.06**	**1.00**	**0.67**	**0.30**	**0.20**	0.54	0.01	0.00	0.00	0.00	0.00	0.62	0.02	0.00	0.00	0.00	0.00
Skin Sun Exposed Lower leg	**0.67**	**0.05**	**1.00**	**0.67**	**0.30**	**0.20**	0.52	0.01	0.00	0.00	0.00	0.00	0.62	0.02	0.00	0.00	0.00	0.00
Testis	**0.63**	**0.01**	**0.00**	**0.00**	**0.00**	**0.00**	0.51	**0.01**	0.00	0.00	0.00	0.00	0.57	**0.01**	0.00	0.00	0.00	0.00
Whole Blood	**0.69**	**0.08**	**1.00**	**0.33**	**0.20**	**0.25**	0.54	0.04	0.00	0.00	0.10	0.15	0.61	0.06	0.00	0.00	0.00	0.00
Whole Brain	**0.61**	0.07	0.00	0.33	0.30	0.20	0.53	0.04	0.00	0.00	0.00	0.00	0.56	**0.08**	**1.00**	**0.67**	**0.50**	**0.40**
Average	**0.67**	**0.06**	**0.53**	**0.37**	**0.25**	**0.21**	0.52	0.01	0.00	0.00	0.01	0.01	0.59	0.02	0.18	0.08	0.08	0.05

Table S2. P@K of WGAND 'ensemble' method constructed per feature.

Tissue Network	Mean Error				Error Standard Deviation				Median Error				Sum of Errors			
	P@1	P@3	P@10	P@20	P@1	P@3	P@10	P@20	P@1	P@3	P@10	P@20	P@1	P@3	P@10	P@20
Artery Aorta	0.00	0.00	**0.10**	**0.10**	0.00	0.00	**0.10**	0.05	0.00	0.00	0.00	0.05	0.00	0.00	**0.10**	0.05
Brain Cerebellum	0.00	0.00	0.00	0.00	0.00	0.00	0.00	0.00	0.00	0.00	0.00	0.00	0.00	0.00	0.00	**0.05**
Brain Cortex	0.00	0.00	0.00	0.00	0.00	0.00	0.00	0.00	0.00	0.00	0.00	0.00	**1.00**	**0.33**	**0.10**	**0.15**
Brain Spinal cord cervical c 1	0.00	0.00	0.00	0.00	0.00	0.00	0.00	0.00	0.00	0.00	0.00	0.00	0.00	**0.33**	**0.10**	**0.05**
Heart Atrial Appendage	**1.00**	0.33	0.20	0.35	0.00	0.33	0.40	0.40	**1.00**	0.67	0.40	0.35	**1.00**	**1.00**	**0.50**	**0.45**
Heart Left Ventricle	0.00	**0.67**	0.60	0.50	0.00	0.33	0.40	0.40	0.00	**0.67**	0.70	**0.55**	**1.00**	**0.67**	0.50	0.50
Liver	**1.00**	**0.33**	**0.10**	**0.05**	0.00	0.00	0.10	**0.05**	**1.00**	**0.33**	**0.10**	0.05	0.00	0.00	0.00	**0.05**
Lung	0.00	**0.33**	0.10	0.05	0.00	**0.33**	0.10	0.10	0.00	**0.33**	0.10	0.05	0.00	0.00	0.00	0.00
Muscle Skeletal	0.00	0.00	0.00	0.15	0.00	0.00	0.30	0.20	0.00	0.00	0.00	0.15	0.00	**0.67**	**0.40**	**0.25**
Nerve Tibial	**1.00**	**0.67**	0.20	0.10	0.00	0.00	0.20	0.15	**1.00**	**0.67**	**0.20**	0.10	0.00	0.33	**0.30**	**0.20**
Ovary	0.00	0.00	0.00	0.00	0.00	0.00	**0.10**	**0.05**	0.00	0.00	0.00	0.00	0.00	**0.33**	**0.10**	**0.05**
Pituitary	0.00	0.00	**0.10**	**0.10**	0.00	**0.33**	**0.10**	0.05	0.00	0.00	**0.10**	**0.10**	0.00	0.00	0.00	0.00
Skin Not Sun Exposed Suprapubic	**1.00**	**0.67**	**0.30**	0.20	0.00	0.00	0.10	0.20	**1.00**	**0.67**	**0.30**	0.20	**1.00**	**0.67**	**0.30**	0.15
Skin Sun Exposed Lower leg	**1.00**	**0.67**	**0.40**	**0.25**	0.00	0.33	0.10	0.15	**1.00**	**0.67**	**0.40**	**0.25**	**1.00**	**0.67**	0.30	0.15
Testis	0.00	0.00	0.00	0.00	0.00	0.00	0.00	**0.05**	0.00	0.00	0.00	0.00	0.00	0.00	0.00	**0.05**
Whole Blood	0.00	0.00	0.10	0.05	0.00	0.00	0.20	0.20	0.00	0.00	0.10	0.10	0.00	**0.33**	**0.30**	**0.25**
Whole Brain	0.00	0.33	0.10	0.15	0.00	0.00	0.10	0.10	0.00	0.33	0.10	0.20	**1.00**	**0.67**	**0.40**	**0.30**
Average	0.29	0.24	0.14	0.12	0.00	0.10	0.14	0.13	0.29	0.25	0.15	0.13	**0.35**	**0.35**	**0.20**	**0.16**

Tissue Network	SEM				Mean Absolute Error				Absolute Error Standard Deviation				Median Absolute Error				Sum of Absolute Errors				Standard Absolute Error of the Mean			
	P@1	P@3	P@10	P@20	P@1	P@3	P@10	P@20	P@1	P@3	P@10	P@20	P@1	P@3	P@10	P@20	P@1	P@3	P@10	P@20	P@1	P@3	P@10	P@20
Artery Aorta	0.00	0.00	0.00	0.00	0.00	0.00	**0.10**	**0.10**	0.00	0.00	**0.10**	0.05	0.00	0.00	0.00	0.05	0.00	0.00	0.00	0.00	0.00	0.00	0.00	0.00
Brain Cerebellum	0.00	0.00	0.00	0.00	0.00	0.00	0.00	0.00	0.00	0.00	0.00	0.00	0.00	0.00	0.00	0.00	0.00	0.00	0.00	0.00	0.00	0.00	0.00	0.00
Brain Cortex	0.00	0.00	0.00	0.00	0.00	0.00	0.00	0.00	0.00	0.00	0.00	0.00	0.00	0.00	0.00	0.00	0.00	0.00	0.00	0.10	0.00	0.00	0.00	0.00
Brain Spinal cord cervical c 1	0.00	0.00	0.00	0.00	0.00	0.00	0.00	0.00	0.00	0.00	0.00	0.00	0.00	0.00	0.00	0.00	0.00	0.00	0.00	0.00	0.00	0.00	0.00	0.00
Heart Atrial Appendage	0.00	0.00	0.00	0.05	**1.00**	0.33	0.20	0.35	0.00	0.33	0.40	**0.45**	**1.00**	0.67	0.40	0.35	0.00	0.00	0.10	0.05	0.00	0.00	0.10	0.10
Heart Left Ventricle	0.00	0.00	0.10	0.05	0.00	**0.67**	0.60	0.50	0.00	0.33	0.50	0.40	0.00	**0.67**	**0.70**	**0.55**	0.00	0.00	0.10	0.05	0.00	0.00	0.10	0.10
Liver	0.00	0.00	0.00	**0.05**	**1.00**	**0.33**	**0.10**	**0.05**	0.00	0.00	**0.10**	**0.05**	**1.00**	**0.33**	**0.10**	0.05	0.00	0.00	0.00	0.00	0.00	0.00	**0.10**	**0.05**
Lung	0.00	0.00	0.10	0.10	0.00	**0.33**	0.10	0.05	0.00	**0.33**	**0.30**	**0.15**	0.00	**0.33**	0.10	0.05	0.00	0.00	0.10	0.10	0.00	0.00	0.10	0.10
Muscle Skeletal	0.00	0.00	0.00	0.00	0.00	0.00	0.00	0.15	0.00	0.33	0.20	0.15	0.00	0.00	0.00	0.15	0.00	0.00	0.00	0.10	0.00	0.00	0.00	0.00
Nerve Tibial	0.00	0.00	0.00	0.05	**1.00**	**0.67**	0.20	0.15	0.00	0.33	**0.30**	**0.20**	**1.00**	**0.67**	0.20	0.10	0.00	0.00	0.00	0.00	0.00	0.00	0.10	**0.20**
Ovary	0.00	0.00	0.00	0.00	0.00	0.00	0.00	0.00	0.00	0.00	0.00	0.00	0.00	0.00	0.00	**0.05**	0.00	0.00	0.00	0.00	0.00	0.00	0.00	0.00
Pituitary	**1.00**	**0.33**	**0.10**	0.05	0.00	0.00	**0.10**	**0.10**	0.00	**0.33**	**0.10**	0.05	0.00	0.00	**0.10**	0.05	0.00	0.00	0.00	0.00	**1.00**	**0.33**	**0.10**	0.05
Skin Not Sun Exposed Suprapubic	0.00	0.00	0.10	0.15	**1.00**	**0.67**	**0.30**	0.20	0.00	0.00	**0.30**	**0.30**	**1.00**	**0.67**	**0.30**	0.20	0.00	0.00	0.00	0.05	0.00	0.33	0.10	0.10
Skin Sun Exposed Lower leg	0.00	0.33	0.10	0.10	**1.00**	**0.67**	**0.40**	**0.25**	0.00	0.33	0.20	0.15	**1.00**	**0.67**	**0.40**	**0.25**	0.00	0.00	0.00	0.05	0.00	0.33	0.10	0.10
Testis	0.00	0.00	0.00	0.05	0.00	0.00	0.00	0.00	0.00	**0.33**	**0.10**	**0.05**	0.00	0.00	0.00	0.00	0.00	0.00	0.00	0.00	0.00	0.00	**0.10**	**0.05**
Whole Blood	0.00	0.00	0.00	0.00	0.00	0.00	0.10	0.05	0.00	0.00	**0.30**	0.20	0.00	0.00	0.10	0.10	0.00	0.00	0.10	0.05	0.00	0.00	0.00	0.05
Whole Brain	0.00	0.00	0.00	0.05	0.00	0.33	0.10	0.15	0.00	0.00	0.00	0.10	0.00	0.33	0.10	0.20	**1.00**	0.33	0.20	0.15	0.00	0.00	0.10	0.10
Average	0.06	0.04	0.03	0.04	0.29	0.24	0.14	0.12	0.00	0.16	0.17	0.14	0.29	0.25	0.15	0.13	0.06	0.02	0.04	0.04	0.06	0.06	0.06	0.06

Table S3. The top-ten genes predicted by the ensemble methods and the diseases that these genes were related to.

Protein	Tissue Network	Tissue-associated Disease Protein	Anomalous Protein	Diseases
ENSG00000061455	Artery Aorta	1	1	Patent Ductus Arteriosus 3
ENSG00000107796	Artery Aorta	1	1	Aortic Aneurysm, Familial Thoracic 6
ENSG00000182492	Artery Aorta	0	0	-
ENSG00000115414	Artery Aorta	0	0	-
ENSG00000159251	Artery Aorta	0	0	-
ENSG00000136999	Artery Aorta	0	0	-
ENSG00000077943	Artery Aorta	0	0	-
ENSG00000135324	Artery Aorta	0	0	-
ENSG00000154553	Artery Aorta	0	0	-
ENSG00000133026	Artery Aorta	0	0	-
ENSG00000131095	Brain Cerebellum	0	1	Alexander Disease
ENSG00000104833	Brain Cerebellum	0	1	Leukodystrophy, Hypomyelinating, 6, Dystonia 4, Torsion, Autosomal Dominant
ENSG00000089199	Brain Cerebellum	0	0	-
ENSG00000154127	Brain Cerebellum	0	0	-
ENSG00000105613	Brain Cerebellum	0	1	Mega-Corpus-Callosum Syndrome With Cerebellar Hypoplasia And Cortical Malformations, Cerebellar Hypoplasia
ENSG00000101210	Brain Cerebellum	0	0	-
ENSG00000171885	Brain Cerebellum	0	0	-
ENSG00000188827	Brain Cerebellum	0	0	-
ENSG00000130540	Brain Cerebellum	0	0	-
ENSG00000132639	Brain Cerebellum	0	0	-
ENSG00000131095	Brain Cortex	0	1	Alexander Disease
ENSG00000156475	Brain Cortex	1	0	Spinocerebellar Ataxia 12, Autosomal Dominant Cerebellar Ataxia
ENSG00000130540	Brain Cortex	0	0	-
ENSG00000171885	Brain Cortex	0	1	Brain Edema
ENSG00000129990	Brain Cortex	0	0	-
ENSG00000104833	Brain Cortex	0	0	-
ENSG00000075340	Brain Cortex	0	0	-
ENSG00000127585	Brain Cortex	0	0	-
ENSG00000064787	Brain Cortex	0	0	-
ENSG00000089169	Brain Cortex	0	0	-
ENSG00000064787	Brain Spinal cord cervical c 1	0	0	-
ENSG00000131095	Brain Spinal cord cervical c 1	0	1	Alexander Disease
ENSG00000123560	Brain Spinal cord cervical c 1	0	1	Pelizaeus-Merzbacher Disease, Spastic Paraplegia 2, X-Linked
ENSG00000197971	Brain Spinal cord cervical c 1	0	1	Secondary Progressive Multiple Sclerosis, Demyelinating Disease

Continued on next page

Table S3 – *Continued from previous page*

Protein	Tissue Network	Tissue-associated Disease Protein	Anomalous Protein	Diseases
ENSG00000171885	Brain Spinal cord cervical c 1	0	1	Neuromyelitis Optica
ENSG00000105695	Brain Spinal cord cervical c 1	0	1	Polyneuropathy
ENSG00000156475	Brain Spinal cord cervical c 1	1	1	Spinocerebellar Ataxia 12, Autosomal Dominant Cerebellar Ataxia
ENSG00000104833	Brain Spinal cord cervical c 1	0	0	-
ENSG00000160307	Brain Spinal cord cervical c 1	0	1	Syringoma, Neurofibroma
ENSG00000112280	Brain Spinal cord cervical c 1	0	0	-
ENSG00000134571	Heart Atrial Appendage	1	1	Cardiomyopathy, Familial Hypertrophic, 4
ENSG00000159251	Heart Atrial Appendage	1	1	Atrial Septal Defect 5, Cardiomyopathy, Familial Hypertrophic, 11
ENSG00000175206	Heart Atrial Appendage	1	1	Atrial Standstill 2, Atrial Fibrillation, Familial, 6
ENSG00000120937	Heart Atrial Appendage	0	0	-
ENSG00000077522	Heart Atrial Appendage	1	1	Cardiomyopathy, Dilated, 1Aa, With Or Without Left Ventricular Noncompaction, Myopathy, Distal, 6, Adult-Onset, Autosomal Dominant
ENSG00000155657	Heart Atrial Appendage	1	1	Myopathy, Myofibrillar, 9, With Early Respiratory Failure, Congenital Myopathy 5 With Cardiomyopathy
ENSG00000118194	Heart Atrial Appendage	1	1	Cardiomyopathy, Dilated, 1D, Cardiomyopathy, Familial Hypertrophic, 2
ENSG00000173991	Heart Atrial Appendage	1	1	Cardiomyopathy, Familial Hypertrophic, 25, Muscular Dystrophy, Limb-Girdle, Autosomal Recessive 7
ENSG00000104879	Heart Atrial Appendage	0	0	-
ENSG00000198523	Heart Atrial Appendage	1	1	Cardiomyopathy, Dilated, 1P, Cardiomyopathy, Familial Hypertrophic, 18
ENSG00000134571	Heart Left Ventricle	1	1	Cardiomyopathy, Familial Hypertrophic, 4, Left Ventricular Noncompaction 10
ENSG00000159251	Heart Left Ventricle	1	1	Atrial Septal Defect 5, Cardiomyopathy, Familial Hypertrophic, 11
ENSG00000092054	Heart Left Ventricle	1	1	Myopathy, Distal, 1, Congenital Myopathy 7A, Myosin Storage, Autosomal Dominant
ENSG00000077522	Heart Left Ventricle	1	1	Cardiomyopathy, Dilated, 1Aa, With Or Without Left Ventricular Noncompaction, Myopathy, Distal, 6, Adult-Onset, Autosomal Dominant

Continued on next page

Table S3 – *Continued from previous page*

Protein	Tissue Network	Tissue-associated Disease Protein	Anomalous Protein	Diseases
ENSG00000155657	Heart Left Ventricle	1	1	Myopathy, Myofibrillar, 9, With Early Respiratory Failure, Congenital Myopathy 5 With Cardiomyopathy
ENSG00000118194	Heart Left Ventricle	1	1	Cardiomyopathy, Dilated, 1D, Cardiomyopathy, Familial Hypertrophic, 2
ENSG00000129991	Heart Left Ventricle	1	1	Cardiomyopathy, Dilated, 2A, Cardiomyopathy, Familial Hypertrophic, 7
ENSG00000143632	Heart Left Ventricle	0	0	-
ENSG00000186439	Heart Left Ventricle	1	1	Cardiac Arrhythmia Syndrome, With Or Without Skeletal Muscle Weakness, Catecholaminergic Polymorphic Ventricular Tachycardia 5
ENSG00000114854	Heart Left Ventricle	1	1	Cardiomyopathy, Familial Hypertrophic, 13, Cardiomyopathy, Dilated, 1Z
ENSG00000171557	Liver	0	1	†Afibrinogenemia, Congenital
ENSG00000171564	Liver	0	1	†Afibrinogenemia, Congenital
ENSG00000163631	Liver	0	1	Analbuminemia, Hyperthyroxinemia, Familial Dysalbuminemic
ENSG00000118137	Liver	0	1	Hypoalphalipoproteinemia, Primary, 2, Hypoalphalipoproteinemia, Primary, 2, Intermediate
ENSG00000124253	Liver	0	1	Phosphoenolpyruvate Carboxykinase Deficiency, Cytosolic, Pepck 1 Deficiency
ENSG00000198650	Liver	1	1	Tyrosinemia, Type Ii, Tyrosinemia
ENSG00000145321	Liver	0	1	Hepatic Encephalopathy
ENSG00000171759	Liver	0	1	Phenylketonuria, Hyperphenylalaninemia
ENSG00000257017	Liver	0	1	Anhaptoglobinemia
ENSG00000197249	Liver	0	1	Alpha-1-Antitrypsin Deficiency
ENSG00000187908	Lung	0	0	-
ENSG00000182010	Lung	0	0	-
ENSG00000171885	Lung	0	0	-
ENSG00000175899	Lung	0	0	-
ENSG00000171345	Lung	0	0	-
ENSG00000133661	Lung	0	1	Extrinsic Allergic Alveolitis, Pulmonary Alveolar Proteinosis
ENSG00000197249	Lung	0	1	Alpha-1-Antitrypsin Deficiency, Hemorrhagic Disease Due To Alpha-1-Antitrypsin Pittsburgh Mutation
ENSG00000168484	Lung	1	1	

Continued on next page

Table S3 – *Continued from previous page*

Protein	Tissue Network	Tissue-associated Disease Protein	Anomalous Protein	Diseases
ENSG00000211896	Lung	0	0	-
ENSG00000165140	Lung	0	0	-
ENSG00000130595	Muscle Skeletal	0	1	Arthrogryposis, Distal, Type 2B2, Arthrogryposis, Distal, Type 1A
ENSG00000183091	Muscle Skeletal	1	1	Nemaline Myopathy 2, Arthrogryposis Multiplex Congenita 6
ENSG00000130957	Muscle Skeletal	0	0	-
ENSG00000105048	Muscle Skeletal	1	1	Nemaline Myopathy 5, Nemaline Myopathy
ENSG00000197893	Muscle Skeletal	0	1	Myopathy, Myofibrillar, 5, Myopathy, Myofibrillar, 4
ENSG00000069869	Muscle Skeletal	0	0	-
ENSG00000143632	Muscle Skeletal	1	1	Myopathy, Scapulohumeroperoneal, Congenital Myopathy 2A, Typical, Autosomal Dominant
ENSG00000155657	Muscle Skeletal	1	1	Myopathy, Myofibrillar, 9, With Early Respiratory Failure, Congenital Myopathy 5 With Cardiomyopathy
ENSG00000086967	Muscle Skeletal	0	1	Lethal Congenital Contracture Syndrome 4, Nemaline Myopathy 9
ENSG00000186439	Muscle Skeletal	0	0	-
ENSG00000105227	Nerve Tibial	1	1	Charcot-Marie-Tooth Disease, Demyelinating, Type 4F, Hypertrophic Neuropathy Of Dejerine-Sottas
ENSG00000102385	Nerve Tibial	0	1	Charcot-Marie-Tooth Disease, Charcot-Marie-Tooth Disease, Demyelinating, Type 4F
ENSG00000064787	Nerve Tibial	0	0	-
ENSG00000158887	Nerve Tibial	1	1	Hypertrophic Neuropathy Of Dejerine-Sottas, Charcot-Marie-Tooth Disease, Demyelinating, Type 1B
ENSG00000181092	Nerve Tibial	0	0	-
ENSG00000109099	Nerve Tibial	1	1	Charcot-Marie-Tooth Disease And Deafness, Charcot-Marie-Tooth Disease, Demyelinating, Type 1A
ENSG00000171345	Nerve Tibial	0	0	-
ENSG00000064300	Nerve Tibial	0	0	-
ENSG00000160307	Nerve Tibial	0	1	Neurofibroma
ENSG00000197971	Nerve Tibial	0	0	-
ENSG00000136931	Ovary	1	1	46,Xx Sex Reversal 4, Premature Ovarian Failure 7

Continued on next page

Table S3 – *Continued from previous page*

Protein	Tissue Network	Tissue-associated Disease Protein	Anomalous Protein	Diseases
ENSG00000180447	Ovary	0	0	-
ENSG00000156475	Ovary	0	0	-
ENSG00000185070	Ovary	0	0	-
ENSG00000107317	Ovary	0	0	-
ENSG00000198300	Ovary	0	0	-
ENSG00000143768	Ovary	0	1	Infertility
ENSG00000125398	Ovary	0	0	-
ENSG00000185559	Ovary	0	0	-
ENSG00000117425	Ovary	0	0	-
ENSG00000089199	Pituitary	0	1	Pheochromocytoma
ENSG00000069011	Pituitary	0	0	-
ENSG00000135346	Pituitary	0	0	-
ENSG00000136931	Pituitary	0	0	-
ENSG00000124253	Pituitary	0	0	-
ENSG00000129990	Pituitary	0	0	-
ENSG00000170421	Pituitary	0	0	-
ENSG00000149295	Pituitary	0	0	-
ENSG00000064835	Pituitary	1	1	Pituitary Hormone Deficiency, Combined Or Isolated, 1, Isolated Growth Hormone Deficiency, Type Ii
ENSG00000105894	Pituitary	0	0	-
ENSG00000167768	Skin Not Sun Exposed Suprapubic	1	1	Palmoplantar Keratoderma, Nonepidermolytic, Ichthyosis Hystrix, Curth-Macklin Type
ENSG00000186081	Skin Not Sun Exposed Suprapubic	1	1	Epidermolysis Bullosa Simplex 2F, With Mottled Pigmentation, Epidermolysis Bullosa Simplex 2E, With Migratory Circinate Erythema
ENSG00000186847	Skin Not Sun Exposed Suprapubic	1	1	Dermatopathia Pigmentosa Reticularis, Naegeli-Franceschetti-Jadassohn Syndrome
ENSG00000096696	Skin Not Sun Exposed Suprapubic	0	1	Skin Fragility-Woolly Hair Syndrome, Epidermolysis Bullosa, Lethal Acantholytic
ENSG00000172867	Skin Not Sun Exposed Suprapubic	0	1	Ichthyosis Bullosa Of Siemens, Epidermolytic Hyperkeratosis
ENSG00000171346	Skin Not Sun Exposed Suprapubic	0	1	Morpheaform Basal Cell Carcinoma, Infiltrative Basal Cell Carcinoma
ENSG00000161634	Skin Not Sun Exposed Suprapubic	0	0	-
ENSG00000163207	Skin Not Sun Exposed Suprapubic	0	1	Cholesteatoma Of Middle Ear, Porokeratosis

Continued on next page

Table S3 – *Continued from previous page*

Protein	Tissue Network	Tissue-associated Disease Protein	Anomalous Protein	Diseases
ENSG00000081277	Skin Not Sun Exposed Suprapubic	0	1	Ectodermal Dysplasia/Skin Fragility Syndrome, Ectodermal Dysplasia
ENSG00000178372	Skin Not Sun Exposed Suprapubic	0	0	-
ENSG00000167768	Skin Sun Exposed Lower leg	1	1	Palmoplantar Keratoderma, Nonepidermolytic, Ichthyosis Hystrix, Curth-Macklin Type
ENSG00000186081	Skin Sun Exposed Lower leg	1	1	Epidermolysis Bullosa Simplex 2F, With Mottled Pigmentation, Epidermolysis Bullosa Simplex 2E, With Migratory Circinate Erythema
ENSG00000096696	Skin Sun Exposed Lower leg	0	1	Skin Fragility-Woolly Hair Syndrome, Epidermolysis Bullosa, Lethal Acantholytic
ENSG00000172867	Skin Sun Exposed Lower leg	0	1	Ichthyosis Bullosa Of Siemens, Epidermolytic Hyperkeratosis
ENSG00000161634	Skin Sun Exposed Lower leg	0	0	-
ENSG00000186847	Skin Sun Exposed Lower leg	1	1	Dermatopathia Pigmentosa Reticularis, Naegeli-Franceschetti-Jadassohn Syndrome
ENSG00000163207	Skin Sun Exposed Lower leg	0	0	-
ENSG00000171346	Skin Sun Exposed Lower leg	0	1	Morpheaform Basal Cell Carcinoma, Infiltrative Basal Cell Carcinoma
ENSG00000081277	Skin Sun Exposed Lower leg	0	1	Ectodermal Dysplasia/Skin Fragility Syndrome, Ectodermal Dysplasia
ENSG00000149418	Skin Sun Exposed Lower leg	1	1	Ichthyosis, Congenital, Autosomal Recessive 11, Ichthyosis
ENSG00000118137	Testis	0	0	-
ENSG00000131747	Testis	0	0	-
ENSG00000198033	Testis	0	0	-
ENSG00000174015	Testis	0	0	-
ENSG00000170777	Testis	0	0	-
ENSG00000168454	Testis	0	0	-
ENSG00000183207	Testis	0	0	-
ENSG00000166851	Testis	0	0	-
ENSG00000133101	Testis	0	1	Testicular Cancer
ENSG00000117399	Testis	0	0	-
ENSG00000148346	Whole Blood	0	0	-
ENSG00000104918	Whole Blood	0	0	-
ENSG00000124731	Whole Blood	0	0	-
ENSG00000132965	Whole Blood	0	0	-
ENSG00000143546	Whole Blood	0	0	-

Continued on next page

Table S3 – *Continued from previous page*

Protein	Tissue Network	Tissue-associated Disease Protein	Anomalous Protein	Diseases
ENSG00000141480	Whole Blood	0	0	-
ENSG00000188536	Whole Blood	1	1	Hemoglobin H Disease, Alpha-Thalassemia
ENSG00000100985	Whole Blood	0	0	-
ENSG00000140368	Whole Blood	1	1	Pyogenic Sterile Arthritis, Pyoderma Gangrenosum, And Acne
ENSG00000066336	Whole Blood	0	0	-
ENSG00000131095	Whole Brain	0	1	Alexander Disease
ENSG00000171885	Whole Brain	0	1	Neuromyelitis Optica
ENSG00000156475	Whole Brain	1	1	Spinocerebellar Ataxia 12, Autosomal Dominant Cerebellar Ataxia
ENSG00000064787	Whole Brain	0	0	-
ENSG00000130540	Whole Brain	0	0	-
ENSG00000104833	Whole Brain	1	1	Leukodystrophy, Hypomyelinating, 6, Dystonia 4, Torsion, Autosomal Dominant
ENSG00000129990	Whole Brain	0	0	-
ENSG00000089169	Whole Brain	0	0	-
ENSG00000167971	Whole Brain	0	0	-
ENSG00000155980	Whole Brain	1	1	Spastic Paraplegia 10, Autosomal Dominant, Myoclonus, Intractable, Neonatal

Fig1

Click here to access/download;Figure;Figure 1 -infographic.jpg

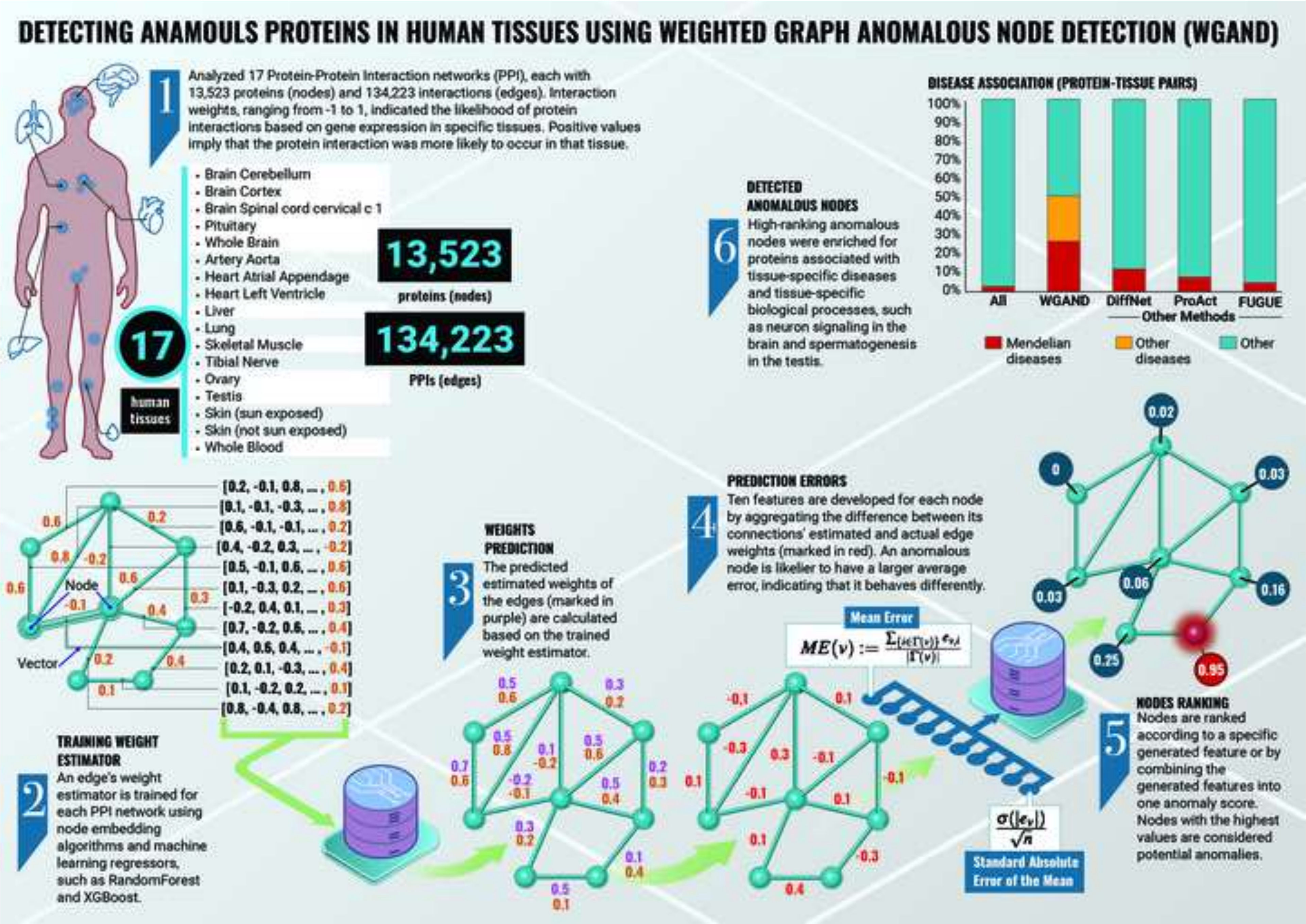

Click here to access/download;Figure;Figure 5.jpg

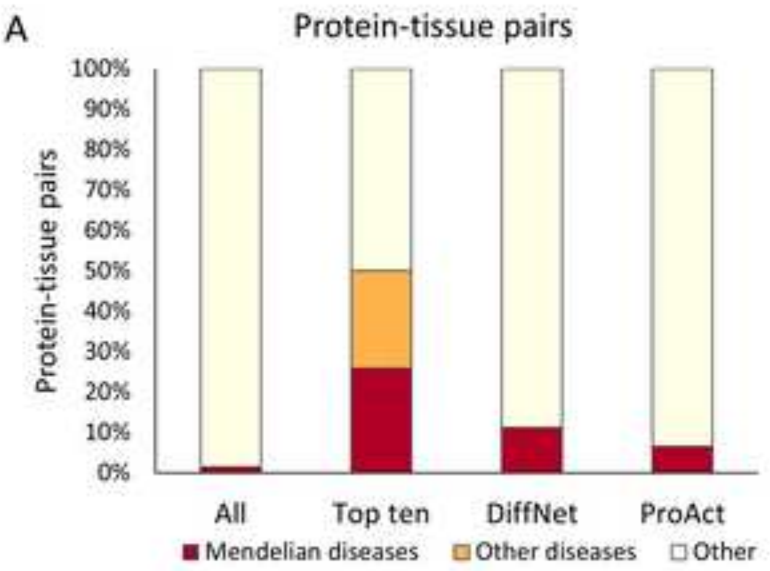

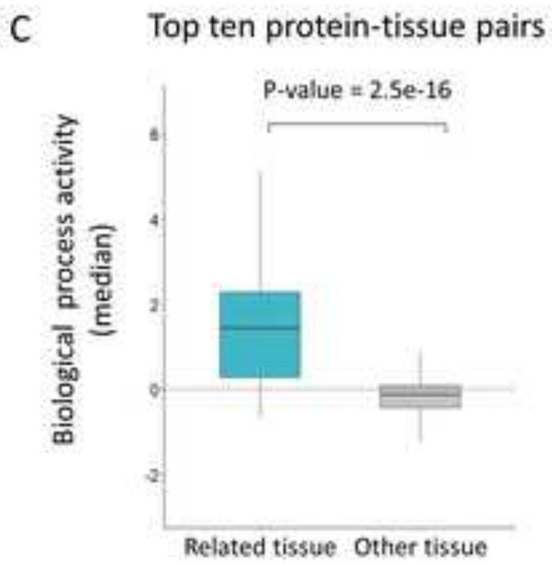

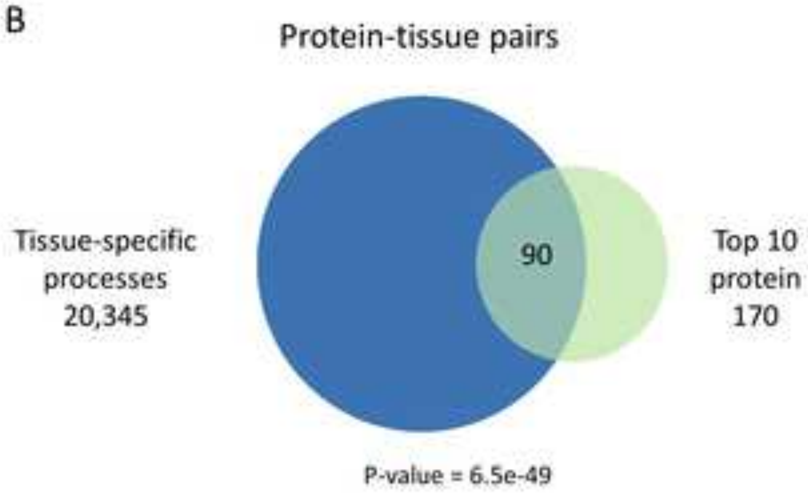

D

Method	Disease-related pairs (%)	Tissue-specific processes (#)	Tissue-specific enrichment (p-value)	Biological process activity test (p-value)
Features' mean	22%	81	9.95e-40	3.65e-10
iForest	19%	87	9.2e-46	2.66e-14
PCA	19%	77	6.5e-36	1.18e-07

 Click here to access/download;Personal Cover;Response Letter - Minor Revision.pdf

Network-based anomaly detection algorithm reveals proteins with major roles in human tissues - Response Letter

Dear Dr. Zhou,

We would like to thank you, Dr. Casey Greene, and the reviewers for your valuable time and feedback. We appreciate the opportunity to refine our manuscript further and have carefully considered the comments provided. Below, we outline our responses and the corresponding changes we have made to improve our work's clarity and scientific rigor. As requested, we have registered WGAND to SciCrunch.org (RRID:SCR_026406) and bio.tools (weighted_graph_anomalous_node_detection_wgand) databases. The revised manuscript highlights new modifications in blue.

Response to Dr. Casey Greene

Reviewer's Concern:

"The authors assume that a node being an anomaly in a PPI causes the disease status. However, a node might be an anomaly simply because it has a disease association and has thus been studied more extensively. Unless the authors can exclude this possibility, they should be careful with their interpretation of their findings."

Response:

We appreciate this important point and agree that care must be taken when interpreting anomaly scores in the context of disease. In the previous version, we clarified that our study does not claim a direct causal relationship between anomaly detection and disease status. Instead, our approach is designed to identify proteins with unusual interaction patterns that may reflect important tissue-specific biological roles, including but not limited to disease association. To address this concern explicitly, we have revised the Discussion section to acknowledge the possibility that disease-associated proteins are more studied and may, therefore, exhibit different network properties. Specifically, we now state (Page 7):

> *"One caveat in our analyses is that training data of disease- or tissue-associated proteins is incomplete. This implies that performance could be improved once more training data is available. It also implies that anomaly prediction scores could be used to pinpoint promising candidates for further analysis. Another potential bias in our analysis is that disease-associated proteins were more likely to be extensively studied than other proteins. Hence, their node degrees in our networks tend to be higher, contributing to their anomaly scores [1]. We controlled for this bias in two ways. Firstly, all tissue networks had the same topology and differed only in edge weights derived from unbiased transcriptomic data. Since anomaly scores were*

tissue-specific, they were not simply affected by differences in protein degrees between disease-associated proteins and other proteins. Secondly, we showed that proteins with major tissue-specific roles beyond disease-association were more likely to be an anomaly in the relevant tissue network. Specifically, we applied WGAND to predict tissue-relevant proteins participating in tissue-specific processes. These were derived either via manual curation or via an unbiased analysis of processes that were preferentially active in specific tissues based on transcriptomic data. Anomaly scores were assessed for each category. We found that high-ranking anomalous proteins were enriched not only for disease-associated proteins (Fig. 5A), but also for proteins involved in tissue-specific processes (Fig. 5B) and tissue preferentially active processes (Fig. 5C). Finally, we compared our results to FUGUE, which had the same goal as WGAND of predicting tissue-relevant proteins going beyond disease-associated proteins. We found that WGAND performed better than FUGUE (Fig. 5A,B)."

We believe these revisions address the reviewer's core concern while maintaining the integrity of our analytical approach. Additionally, we would like to highlight that Figure 5, panels B-D provide further evidence that our anomaly detection method captures biologically meaningful signals beyond simple disease annotation. This figure shows that the Top-10 anomalous proteins are enriched for tissue-specific cellular processes, reinforcing that anomalous behavior is not solely an artifact of prior disease knowledge.

References

[1] Zietz, Michael, et al. "The probability of edge existence due to node degree: a baseline for network-based predictions." *GigaScience* 13 (2024): giae001.
