# Supplementary material for: Network-based anomaly detection algorithm reveals proteins with major roles in human tissues
Source: Gigascience. 2025 Apr 8;14:giaf034. doi: 10.1093/gigascience/giaf034 (PMC11976396; doi:10.1093/gigascience/giaf034)
Supplement: giaf034_Response_to_Reviewer_Comments_Revision_1 [file giaf034_response_to_reviewer_comments_revision_1.pdf]

# Author's Response To Reviewer Comments

See attached letter
